# Fighting addiction's death row: British Columbia Supreme Court Justice Ian Pitfield shows a measure of legal courage

**DOI:** 10.1186/1477-7517-5-31

**Published:** 2008-10-28

**Authors:** Dan Small

**Affiliations:** 1 Department of Medicine, University of British Columbia, Vancouver, Canada; 2 Department of Anthropology, University of British Columbia, Vancouver, Canada; 3 PHS Community Services Society, Vancouver, Canada

## Abstract

The art in law, like medicine, is in its humanity. Nowhere is the humanity in law more poignant than in BC Supreme Court Justice Ian Pitfield's recent judgment in the legal case aimed at protecting North America's only supervised injection facility (SIF) as a healthcare program: PHS Community Services Society versus the Attorney General of Canada. In order to protect the SIF from politicization, the PHS Community Services Society, the community organization that established and operates the program, along with two people living with addiction and three lawyers working for free, *pro bono publico*, took the federal government of Canada to court. The courtroom struggle that ensued was akin to a battle between David and Goliath. The judge in the case, Justice Pitfield, ruled in favour of the PHS and gave the Government of Canada one year to bring the Controlled Drugs and Substances Act (CDSA) into compliance with the country's Charter of Rights and Freedoms. If parliament fails to do so, then the CDSA will evaporate from enforceability and law in June of 2009. Despite the fact that there are roughly twelve million intravenous drug addiction users in the world today, politics andprejudice oards harm reduction are still a barrier to the widespread application of the "best medicine" available for serious addicts. Nowhere is this clearer than in the opposition by conservative Prime Minister Stephen Harper and his faithful servant, federal health minister Tony Clement, towards Vancouver's SIF ("Insite"). The continued angry politicization of addiction will only lead to the tragic loss of life, as addicts are condemned to death from infectious diseases (HIV & hepatitis) and preventable overdoses. In light of the established facts in science, medicine and now law, political opposition to life-saving population health programs (including SIFs) to address the effects of addiction is a kind of implicit capital punishment for the addicted. This commentary examines the socio-political context of the legal case and the major figures that contributed to it. It reviews Justice Pitfield's ruling, a judgment that has brought Canada one step closer to putting a stop to addiction's death row where intravenous drug users are needlessly, for political and ideological reasons alone, forced to face increased risks of death due to AIDS, hepatitis and overdose.

## 

"I am pleading for the future; I am pleading for a time when hatred and cruelty will not control the hearts of men. When we can learn by reason and judgment and understanding and faith that all life is worth saving, and that mercy is the highest attribute of man."

Clarence Darrow[1]

## Introduction: a measure of legal courage

All heroic figures, and in fact all human beings, are flawed. Perhaps it is this self-evident frailty in all humanity, readily apparent for all to see in addiction, that scares us most about injection drug use. Addiction unlocks a window that glimpses into our own imperfections with blunt truthfulness. The quote opening this commentary is from famed American lawyer and orator Clarence Darrow who provided no exception to the certainty of humanity in his character. Early in his career at the turn of the 20^th ^century, a faint shadow was cast over him by the suspicions that he may have displayed poor judgment in a case representing labour leaders. As a result, he left corporate and labour law to take up the pursuit of criminal law as a defence attorney. He went on to become one of the greatest orators in legal history with some of his most famous closing arguments extending to twelve hours in length while he reviewed law, philosophy and the essence of humanity. He had a life long hatred of capital punishment that he saw as a kind of cool and calculated murder by the state. [2] The politicization of responses to addiction, is often led by anger, hatred and fear rather than science, medicine and compassion. With what modern science has shown us about harm reduction initiatives like supervised injection facilities (SIF) and syringe distribution programs, it is becoming increasingly clear that attempts to politically block these measures, based on mistaken moral judgment, is to condemn addicts to a kind of addiction's death row. Justice Pitfield's decision in the matter of PHS Community Services Society versus Attorney General of Canada has further shown us that all life is worth saving [see Additional file 1]. [3]

This commentary focuses on a legal case aimed at protecting the fundamental right to life, liberty and security of the person for people living with addictions by protecting their access to North America's only SIF. The SIF, known as *Insite *in the community, is a health program located in Vancouver, British Columbia aimed at reaching a difficult group of people living with active intravenous addictions in a healthcare setting in order to help reduce HIV/AIDS and Hepatitis by curbing syringe sharing and to prevent fatal drug overdoses with clinical supervision. To date, over **1,000,000 **injections have been supervised at the SIF, injections that might otherwise have occurred in public spaces in unsupervised and dangerous circumstances where overdoses could have occurred without emergency interventions and dangerous injection practices could have taken place. [4] There have been hundreds of overdose events at the facility, many of which, had they occurred in unsafe and unsupervised settings would have surely resulted in death. While the precise number of deaths averted by Insite can never be known, as it would be an unethical and forbidden experiment, it appears that the facility has prevented as many as 12 overdose deaths per year since it opened. [5]

Thesecalculationspoint to the possibility that over fiftyfatal overdoseshave been prevented by Vancouver's SIF since the opening of program. These estimates, of course, do not include the lives that would have been saved by preventing infectious diseasesincluding HIV and HCV. Regardless of the exact number, if even one death could have been prevented, it would be enough.

While Canada had shown strong political leadership in opening the SIF as a health program in September of 2003, the program became the subject of political intrusion in February of 2006 when a minority conservative government came into power under Prime Minister Stephen Harper. [4] Of course, the issue of a comprehensive approach to addiction, that includes harm reduction, doesn't have to be a partisan political issue. Several mayors, of different parties in Vancouver, have supported and support Vancouver's SIF including, Gordon Campbell, Mike Harcourt, Philip Owen, Larry Campbell and Sam Sullivan. Medical and scientific evidence demonstrating the efficacy of Insite has been collected through an independent review by a team of physicians and scientists. The results of their evaluation have been published in over thirty peer reviewed research papers published in internationally recognized academic journals.

The results of this independent evaluation indicate that the program has reduced unsafe injection practices, public disorder, overdose deaths and HIV/Hepatitis while increasing uptake of addiction services and detox and keeping people with extremely compromised health alive to, perhaps, be on the threshold of a successful life one day. [4].

In the face of increasing danger that Prime Minister Stephen Harper and federal Health Minister Tony Clement would not extend a permit for Insite under the Controlled Drugs and Substances Act for Insite past 30 June 2008, the community organization that operates the program, the PHS Community Services Society (PHS), felt compelled to try to protect this life-saving program through the courts. As a result, legal case was brought forward by a community organization, two people living with active addictions and three lawyers working for free (pro bono publico).

Essentially, the case against the Government of Canada followed two streams of argument. The first related to inter-jurisdictional issues:

(1) In the Constitution of Canada, there is a clear division of powers between the Federal and Provincial Government. The PHS made the argument that regulating the SIF operates within the jurisdiction of the Province of BC and that, as such, interference from the Federal Government is inappropriate.

The second pertained to the first part of Canada's Constitution, the Canadian Charter of Rights and Freedoms (the Charter). The critical area of the Charter for Insite is found in section 7:

(2) Section 7 of the Charter states that: "Everyone has the right to life, liberty and security of the person and the right not to be deprived thereof except in accordance with the principles of fundamental justice.". [6] (p. 4) The PHS argued that if the Health Minister were to use the Controlled Drugs and Substances Act (CDSA) to close Insite, then this action would wrongly jeopardize the life chances of people with addictions by denying them access to critical healthcare.

How, then, do we measure heroism in poignant historical moments? Surely, the flaws and frailties of humanity do not turn strong social conscience into fiction? Nowhere is this more evident than in the courageous decision of British Columbia Chief Justice Ian Pitfield on the matter of Insite. In his landmark decision, Judge Pitfield showed a measure of legal courage that is certain to shape Canada in terms of our understanding of addiction as a healthcare issue in the years to come.

## Government of Canada

The Attorney General of Canada hired a formidable legal adversary, John Hunter, Q.C. of Hunter Litigation Chambers as their lead counsel. At the time of his appointment as lead counsel, he was the president of the Law Society of British Columbia. [7] He has represented the Attorney General on numerous occasions:

*"K.L.B. v. v. British Columbia*, (Supreme Court of Canada; 2003) client: Attorney General of British Columbia issue: Crown liability under principles of vicarious liability or non-delegable duty of care for foster parent abuse.

*Tremblay v. Attorney General of British Columbia*, (British Columbia Court of Appeal; 2002) client: Attorney General of British Columbia issue: Whether a Cabinet order dismissing the board of the Legal Services Society was valid.

*Soowahlie Band v. Canada*, (Federal Court of Appeal; 2001) client: Attorney General of Canada issue: Whether Canada should be enjoined from transferring land claimed by the Sto:lo Nation to third parties.

*Human Rights Institute of Canada et al v. Canada (Attorney General)*, (British Columbia Supreme Court and Federal Court Trial Division; 1999) client: Attorney General of Canada issue: Whether an injunction should be granted to restrain the completion of an expropriation of land by the Federal government.

*Luuxhon v. Canada*, (British Columbia Supreme Court; 1998) client: Attorney General of Canada issue: Whether Canada has a legally enforceable obligation to conduct treaty negotiations with First Nations in good faith."[8]

Mr. Hunter specializes in aboriginal law and has represented government clients in opposition to various aboriginal groups (e.g. Musqueam Indian Band, Haida Nation, Soowahlie Band and Luuxhon First Nation). [8] He also specializes in forestry litigation. He has represented private sector forestry clients including companies Weyerhaeuser Company Limited and MacMillan Bloedel.

Mr. Hunter made a significant acknowledgment early in the case. He rose, during a presentation by one of the PHS lawyers, Mr. Arvay, to make the point that the Government of Canada agrees that addiction is an illness. This recognition proved to be a crucial entry into the legal record.

## Heroes figures in the legal establishment of addiction as a healthcare matter

There were many important figures in this legal case that helped to further establish addiction as a matter for the Chief of Medicine rather than the Chief of Police. All of them showed courage and took social risks by participating in this legal case. There was tremendous courage in the three lawyers, who took on the cause of Insite. There was courage shown by provincial and municipal bureaucrats who entered their testimony into the record. The federal bureaucracy, sadly, testified on behalf of the Attorney General of Canada and, as such, defended the position of the Prime Minister and Health Minister, and stood against the provincial bureaucrats from the Vancouver Coastal Health Authority (VCH) and the City of Vancouver. The federal bureaucracy also dispatched legal and administrative staff to assist with, observe and report back on the case. During the trial, a staff lawyer for the Department of Justice assisted Hunter Litigation Chambers by using her personal data assistant to look up and then communicate key facts to Mr. Hunter during the proceedings. There was courage shown from the scientists who evaluate Insite, in providing scientific evidence about the role of Insite as a comprehensive response to addition. There was courage shown from the community organization that established Insite. But most of all, there was courage shown by two people living with addiction, wounded witnesses, who opened up their lives and shared their stories of suffering with the court. The stories of these important contributors to the case will be examined in turn.

## Vancouver Coastal Health authority

Representing the VCH, and the Province of British Columbia (the Province), Ms. Heather Hay provided testimony that enshrined the responsibility of the local health authority as the institution responsible for addressing the public problem of addiction and its epidemiological aftermath. Not all problems, of course, are "public problems". A public problem is one for which a public institution formally takes responsibility for addressing and for which public resources are dedicated. [9] When an issue, such as an epidemic of addiction, is socially transformed into a public problem, then it also becomes the responsibility of public institutions, such as the VCH, to discover and implement a solution. Some social phenomena are transformed into public problems requiring institutional action and resources while others are not. For instance, universal healthcare, homelessness, psychiatric disorders, road racing, childhood poverty, the environment and drunken driving have not in the past been considered public problems, whereas today, in Canada, they are expected to be the focus of government officials and publicly funded bodies.

The construction of addiction as a public problem demanding a public health response began as a result of three key factors in the late 1990s: rising overdose deaths, and the gradual shift in community organizations to attempt to reach increasingly vulnerable populations including injection drug users and a pandemic of addiction accounts for one-third of the HIV infections outside the sub-Saharan world. [10] These factors provided the healthcare context for the establishment of the SIF. Addiction was further transformed into a public problem through the establishment of the Vancouver Agreement in 2000 where all three levels of government officially took on the responsibility to address injection drug use and its consequences. [11]

Ms. Hay's written testimony and submissions brought together a number of important documents and facts pertaining to the epidemic of addiction in Vancouver. The documents in her submission included the momentous 1994 *Report of the of the Task Force into Illicit Narcotic Overdose Deaths in British Columbia *[see Additional file 2] [12] chaired by Chief Coroner Vince Cain, the influential 1996 report *Health Impact of Injection Drug Use and HIV in Vancouver *[see Additional file 3] [13] by Dr. Elizabeth Whynot by Vancouver's Chief Medical Health Officer Dr. John Blatherwick and the landmark 1998 report *HIV, Hepatitis, and Injection Drug Use in British Columbia: Pay Now or Pay Later *[see Additional file 4] [14] by Provincial Health Officer Dr. John S. Millar outlining the need for harm reduction approaches. Ms. Hay also entered into the record the recognition by Vancouver Richmond Health Board (predecessor to Vancouver Coastal Health) in the 1997 that injection drug use and its consequences (spread of infectious disease and overdose deaths) had become an epidemic. This evidence indicated the early identification of addiction as an epidemic, by Dr. John Blatherwick, the Chief Medical Health Officer of the Vancouver Richmond Health Board (predecessor to the VCH), and adopted as a Board Resolution in September 1997 [15] provided substantiation of the planning that went into the establishment of harm reduction initiatives in the community.

Originally trained as a nurse before pursuing graduate studies, Ms. Hay worked in the acute care sector before becoming the Director for Addictions, HIV/AIDS and Aboriginal Health Services for the VCH. Ms. Hay has always maintained a connection to the front-line during her vocational life as indicated by the fact that during her visits to Insite people from the community that rely on the facility warmly greet her. Ms. Hay's testimony crystallized the official view that the VCH recognizes the SIF as an important part of its fundamental responsibility to provide and lead healthcare delivery. As her signature dried and her affidavit was sworn in, she had made a sacred commitment, on behalf of the Province of BC, to a vulnerable group of citizens: those living with active addictions and their families.

## Medical expert for the Vancouver Coastal Health

Dr. David Marsh, the physician lead for addiction medicine at the VCH, also provided evidence on behalf of Insite. He is medical supervisor of the program. He also serves as the VCH Medical Director for Addiction, HIV/AIDS and Aboriginal Health Services. He is the Division Head of Addiction Medicine in the Department of Family and Community Medicine at Providence Health Care (St. Paul's Hospital) and the Leader of Addiction Research at the Centre for Health Evaluation and Outcome Sciences (CHEOS).

Dr. Marsh holds specialist certificates from the Canadian, American and International Societies of Addiction Medicine. He is a Clinical Associate Professor, jointly appointed, in the the Department of Health Care and Epidemiology in the Faculty of Medicine at the University of British Columbia where he teaches addiction medicine and conducts research into innovative addiction treatments including medically managed heroin treatment. At the time of his testimony, he was the immediate past President of the Canadian Society for Addiction Medicine, having served as President between October 2003 and October 2006.

Dr. Marsh reviewed the standard definitions of addiction as a chronic disease according to the Canadian Society of Addiction Medicine and American Psychiatric Association as delineated in the Diagnostic and Statistical Manual. His evidence outlined the usage characteristics at Insite including the fact that over 1,000,000 supervised injection had occurred in the facility and that roughly 60% of the injections were opioids and 40% were stimulants. He also provided an overview of the bio-chemical effects of heroin, cocaine and methamphetamine as well as inherited, psychological and social variables influencing addiction. He also presented a description of drug overdose and intoxication along with the appropriate interventions.

## The City of Vancouver

The City of Vancouver was represented by testimony from Donald MacPherson, Drug Policy Coordinator. His roots reach back to the Downtown Eastside, where Insite is located. Before he became the first and present Drug Policy Coordinator, Mr. MacPherson had been the Director of the local community centre and had served on the board of directors of the PHS Community Services Society (the community organization that initiated and operates the SIF).

MacPherson (2001) is the author of the influential policy document: *Framework for Action: A Four-Pillar Approach to Drug Problems in Vancouver *[see Additional file 5] [16]. This document was drafted in the late 1990's, adopted by the City of Vancouver Council in 2001 under the leadership of Mayor Philip Owen and provides an analytical tool for bringing diverse approaches together to work towards common goals. The Framework incorporates four broad streams of understanding and action with respect to addiction: Prevention, Treatment, Enforcement and Harm Reduction.

Of course, as this is an analytical framework for increasing dialogue and cooperation, the four pillars overlap and converge with one another. There is, by example, harm reduction within policing such as the Vancouver Police Department's Policy 11.04 that provides the possibility for police to avoid attending illicit drug overdoses in order to reduce fatal overdoses that might occur due to fear of prosecution. [17-20] Similarly, state police officers in the districts of Espanola and Santa Fe in New Mexico also employ harm reduction and are trained to administer naloxone (trade named Narcan) in order to save lives by reversing opiate overdoses. [21] Moreover, harm reduction measures such as syringe distribution and supervised injection facilities play a prevention role with respect to HCV and HIV. Further, some prevention programs contain elements of harm reduction by providing practical advise about a spectrum of drug use ranging from active addiction to safer, managed use and abstinence. [22]

The Framework for Action brought different actors together and engendered a spirit of cooperation that helped Insite to commence with the support of a broad base of support. While many traditional drug policy documents contain only three elements: prevention, treatment and enforcement, a kind of "three-legged dog", the City of Vancouver's policy framework was a proud departure amongst cities in North America. As the author of this document, MacPherson put his pen to paper for another important cause with regard to the societal treatment of addiction. He entered evidence on behalf of the City of Vancouver and, in so doing, made a further commitment from the City and the municipal level of government to the core principle that addition is a healthcare matter and a public problem requiring healthcare innovations such as Insite.

## The scientific community

The Centre of Excellence in HIV/AIDS (CFE) provided evidence regarding the scientific evaluation of the SIF. When the SIF was initiated, the CFE was chosen to evaluate the project. Four scientists and clinicians led the evaluation team: Dr. Julio Montaner, Dr. Thomas Kerr, Dr. Evan Wood and Dr. Mark Tyndall. Drs. Montaner, Kerr and Wood provided expert evidence in the case.

There have been a small number of detractors that have attacked the CFE's role in evaluating Insite. These detractors have, as a rule, been associated with or paid by national police organizations. In their condemnation of Insite, they have tried to imply that the reporting of positive scientific results associated with Insite by the evaluation scientists along with their support for the preservation of Insite indicates a loss of objectivity. For example, Canada's national police force, the Royal Canadian Mounted Police, has stated publicly that they are "yet to see an arms-length report of the evaluation of the facility" and that they have not seen "research that we can have confidence in"[23]. The force has remarked that "until such time as we can have arms-length report by an independent person or group to show us how well or how effective that site is, then we're not in a position to support it-period". [23]

The RCMP also appeared to engage consultants to perform additional reviews of SIFS and hired academics with known bias against harm reduction approaches to addiction to provide public criticism of Insite. These attempts, by the national police force, to publicly and covertly undermine a healthcare program and the work of a community agency were met with extensive criticism from the community and the media. [24,25] The possibility that the national police force may have clandestinely funded anti-Insite research is especially concerning. [26] Ultimately, these activities led to a letter of apology from the Deputy Commissioner of the national police force. [27] An internal RCMP review of the circumstances surrounding this research activity is underway. Hopefully, this review signals a new direction for Canada's national police force; one that will lead to them being a partner in a comprehensive approach to addiction that embraces evidenced based medicine and a comprehensive approach to addiction. We live in hope that the RCMP will be a partner rather than an opponent.

Of course, the notion that the CFE research is not "arms-length" is farcical. The CFE has published the results of their evaluation in peer-reviewed journals including some of the most respected scientific and medical journals in the world. To date, they have published thirty peer-reviewed papers on the SIF. [28-57]

The peer review stream was chosen precisely in order to provide the uppermost standard for "arm's length" evaluation to ensure the highest quality and objectivity in reviewing the outcomes of the program. Furthermore, to imply a loss of objectivity by the CFE would also require that nearly the entire medical and scientific community had also lost objectivity. In 2007, 130 leading scientists, physicians and healthcare professionals in Canada endorsed a commentary published in a national medical journal publicly stating that the research evaluation on Vancouver's SIF indicated that the healthcare program had reduced harms associated injection drug use and that no adverse consequences had resulted. [58] Likewise, the Canadian Medical Association (CMA) has come out strongly in favour of harm reduction and Insite. In a letter to Canada's largest newspaper, Dr. Brian Day, President of the CMA states:

"In this matter, the science is clear: Harm reduction is a proven and effective tool. Marginalizing an already vulnerable population and leaving them at even greater risk of disease and death is bad medicine and, as the polls show, even worse politics. And with the B.C. government's plans to intervene on behalf of Insite, Canadians should rightly wonder why their tax dollars are going to be financing both sides of this argument. They also should wonder why the federal government seems to be opposed to safe injection sites in British Columbia, but is willing to consider them in Quebec. Clement's public hedging on Quebec's proposal [for an SIF] is further proof that his decision appears to be based on political science and not the real thing. When it comes to safe injection sites, Conservatives need to consider the health of all Canadians, not just those who agree with the government's ideological bias against drug-addicted patients.". [59]

In fact, to oppose the scientific data on the subject would itself appear to be driven by ideology rather than objectivity.

If it were a healthcare issue other than addiction, then clinicians and researchers calling for the best medicine wouldn't have their objectivity called into question. If, for example, a group of researchers and physicians were advocating for the clinical application of an effective cancer treatment, then surely they wouldn't be accused of somehow crossing a line of objectivity?

In fact, I would like to carry this argument one step further. It is the duty of clinicians performing healthcare research to be concerned about clinical application and public policy that improves the health in the community. [60] The glorious days of pursuing knowledge just for knowledge sake in healthcare, like examining theoretical extraction of rainwater from zucchinis, are gone. In my view, part of the responsibility of scientists and clinicians performing healthcare research is to employ what they have learned from their research in order to improve patient lives. And that is exactly what the Centre for Excellence in HIV/AIDS has done through their research, public statements and participation in this legal case. If it closes, people will die from preventable overdoses and HIV infections. It's that simple.

## Government of Canada and PHS witnesses

The Government of Canada relied on three main witnesses: a federal bureaucrat, a retired pharmacist and an addiction physician with what appeared to be little or no experience working with the vulnerable and multiply barried population of injection drug users served by the SIF. The addiction physician engaged by Canada was "more closely associated with healthcare professionals and airline pilots, a significantly different group from injection drug uses in the DTES". [3] (p. 24). In preparation for his testimony, the physician made a visit to the Downtown Eastside in order to obtain a tour of Insite on 19 March 2008. The retired professor of pharmacology "did not depose to any personal knowledge regarding Insite, or to involvement in any aspect of its operations" (p. 30). The employee of Health Canada provided more general information about drug policy in Canada. Neither of the two witnesses on behalf of Canada "deposed any specific observations about Insite or their individual assessment of its efficacy". [3] (p. 30). The expert witnesses testifying on behalf of the PHS, had significant knowledge of the efficacy, evaluation and operation of Insite. They also all had extensive experience working with marginalized injection drug users with multiple barriers to their medical and social tenure. They had all also made noteworthy contributions to the research and treatment of addiction and its consequences.

Dr. Julio Montaner provided evidence from the CFE. He is a practising physician who treats people living with additions and HIV. He is Professor of Medicine at the University of British Columbia, Chair of AIDS research at St. Paul's Hospital, Director of the BC Centre in HIV/AIDS, Director of the SPH Immunodeficiency Clinic, National Co-Director of the Canadian HIV Trials Network and President-Elect of the International AIDS Society. He has pioneered therapies in the treatment of AIDS and received over two-dozen awards for teaching, research and public service including the Pasteur Prize and the Clinical Infectious Diseases Award. He is the editor or co-editor of a dozen scholarly journals. He has written 350 peer reviewed articles. He provided testimony outlining that the methods chosen for evaluating the SIF were at the highest level of scientific enquiry. He also affirmed that the program demonstrated clear public health and community benefit by reaching an under-served population.

Dr. Evan Wood is a physician and researcher. He holds aPhD and MD. He has published over 170 peer-reviewed scientific articles and has been the lead author of articles on the SIF published in leading medical journals including the Lancet, Canadian Medical Association Journal, Journal of the American Medical Association and New England Journal of Medicine. He is a clinical assistant professor with appointments in the Department of Medicine and Epidemiology at the University of British Columbia. He provided evidence outlining the first three years of the evaluation that generated 22 peer-reviewed publications on the outcomes of the SIF. He testified that the first three years of study revealed a number of key benefits associated with the SIF including: reduced dangerous injection practices, reduced public injection and increased uptake of treatment. Moreover, he revealed that the studies examined potential harms associated with the healthcare project but no evidence of deleterious impacts was discovered. He reviewed a number of studies for which he was the principal author in his evidence. [43-45,47-56]

Dr. Thomas Kerr first began his work with the injection drug using population began at the Dr. Peter Centre for people living with AIDS. He holds a PhD in psychology and behavioural science. He is a co-principal investigator of the Scientific Evaluation Supervised of Supervised Injecting (SEOSI) study that focuses on Insite. He has published over 150 peer-reviewed scientific articles and has written articles on the SIF published in leading medical journals including the Lancet, Canadian Medical Association Journal, Journal of the American Medical Association and New England Journal of Medicine. He is a clinical assistant professor with appointments in the Department of Medicine and Epidemiology at the University of British Columbia. He reviewed five key studies, of which he was the principal author, as part of his evidence. [30-34]

## International physician specialist in the treatment of injection drug use

From Canada, Australia looms large on the horizon of healthcare as a kind of sister country with regard to innovations in addiction treatment. Australia opened a Medically Supervised Injecting Centre (MSIC) in May of 2001, two years before Vancouver opened the first such facility in North America. A number of Australians have extended their social conscience to assist Canada in developing the best addiction medicine. In 2000, Tony Trimmingham, a father who tragically lost his son to an overdose, travelled to Vancouver to share his story and help lay the groundwork for the public understanding of addiction as a healthcare matter. Dr. Alex Wodak, a practising physician in the realm of addiction medicine, has visited Canada, both before and after the establishment of Insite, numerous times in order to acquaint himself with the public problem of addiction in Vancouver. He graciously agreed to provide extensive expert evidence in the case *pro bono publico*. There are only two supervised injection facilities outside of Europe (in Australia and Canada) and Dr. Wodak's testimony further strengthened the special bond between our two countries in addressing the pandemic of addiction using humane and evidenced based initiatives.

Dr. Wodak is a physician and specialist in internal medicine who has specialized in the treatment of alcohol and drug addiction for over 30 years. He has been the Director of the Alcohol and Drug Service at St. Vincent's Hospital in Darlinghurst, Australia since 1982. He has published 239 peer-reviewed papers examining the health risks and treatment of injection drug use. His testimony outlined three deadly health conditions associated with injection drug use: overdoses, local infections (bacterial abscesses, endocarditus, brain abscess) and infectious disease (HIV, hepatitis C, B, bacterial, fungal and parasitic infections). He provided an opinion on the scientific research concerning harm reduction measures. He also reviewed the scientific literature on the outcomes associated with SIFs and Insite in particular. After review of the studies on Insite in his affidavit, he provided the expert opinion that the research conducted was in keeping with existing research indicating beneficence without significant negative consequences. He also stated under oath that the research performed by the CFE had set the highest standard, in fact, a benchmark, for evaluation and scientific rigour of supervised injection facilities.

## The kind country doctor in the inner city

Reaching vulnerable populations with medicine in the inner city, with multiple barriers to their healthcare tenure, demands an inversion of medical practice. Rather than expecting autonomous patients to attend healthcare facilities, seek out services and advocate for themselves as their own personal case managers, barriers need to be removed, healthcare has to be brought to the population. In essence, what is required is a return to the "kind country doctor" of the past that performed "house calls". However, rather than visiting the country homes and farms of the patient, the doctor has to visit 100 square foot single room occupancy hotels in the inner city. The most challenging population to reach with healthcare, housing and services are those with **active **addictions, histories of non-compliance, conflict with the law, multiple health conditions (e.g. HIV, HCV) and untreated psychiatric illness (primarily personality related disorders). This population will not, as a rule, travel great distances to obtain healthcare. They do not have automobiles or telephones. For them, travelling from Vancouver's DTES to the main hospital is like travelling from London to Edinburgh. Further, many have severe health problems that limit their mobility.

We cannot expect this population to come to healthcare; healthcare has to go to them.

Providing medical care to this population, the social lepers of today [60,61], is not like fighting for market share between multinational corporations. There is, in contrast, little competition to provide healthcare to this vulnerable group. It requires a special commitment and a special physician. Dr. Gabor Mate is one of these special physicians and he has been treating this population of social lepers from within the Portland Hotel in Vancouver's Downtown Eastside for a decade. He provided evidence as a practising physician, working with the most difficult to treat patient group imaginable, often neglected, turned away and forgotten by mainstream physicians, in the inner city. A large portion of this group is dually diagnosed: suffering from active addiction and personality related psychiatric illness and, as a result, are sadly not eligible for mainstream mental health services. His testimony provided an illustration, based on extensive "on the ground" medical experience, of how innovative healthcare has to be fitted to this patient population rather than expecting this patient population to fit to pre-existing notions of healthcare.

## A community organization: PHS community services society

Many thousands of low-income residents in the Downtown Eastside (DTES) of Vancouver typically live in 80 to 140 square foot hotel rooms where they share a single bathroom and kitchen with dozens of other tenants. The community organization that developed and operates Insite is the PHS Community Services Society (formerly the Portland Hotel Society; PHS). The organization began in an old "single room" hotel (SRO) in the DTES 1993 called the Portland Hotel. The philosophy and practice of the organization traces its roots back to that early and ongoing experience in providing supported housing to people with multiple barriers to their social and medical tenure (many of whom were active injection drug users).

Much of the constituency of the downtown eastside hotels has changed in the last twenty years. As of June 2007, there were 4,992 private SRO units in the Downtown Eastside and surrounding communities of Chinatown, Gastown and Strathcona representing 83 per cent of the 5,985 private SROs throughout the entire downtown core of Vancouver. [62] Including private SROs, non-profit housing, there are a total of 11,131 housing units in the area. This population is no longer simply reflected by an image of unemployed or low-income individuals on a fixed income. Rather, today, many of the individuals who inhabit this often-demonized district of Vancouver have are more aptly described in terms of the challenges they face as the "hard to house", "hard to treat", "hard to reach" or "housing first" population. They live with multiple health and social barriers such as:

• Serious and persistent **active **drug use

• Poverty

• Survival street involvement (e.g. survival sex trade)

• Malnutrition

• Chronic medical problems

• A history of non-compliance

• Untreated psychiatric illness (including personality related disorders)

• HIV and AIDS related illness

• Increased incidence of Hepatitis A, B, C

• Conflict with the law

• Lower levels of education

• High incidence of childhood trauma and adverse life events

• High degree of multiple diagnoses (e.g. active addiction, mental illness, hepatitis and HIV/AIDS)

• Traumatic residential school experiences

• Stigmatization

• Denial of housing

• Denial of healthcare services

• Denial of support services

The PHS has learned, from experience, that the challenges encountered with this group are amenable to intervention if services are offered in a low-threshold (without barriers) and tenant-centred manner. In addition, the needs of this group have to be addressed by an adequate level of resources that respond to the following challenges:

• Many do not have a family doctor (healthcare exclusion)

• Many individuals do not have personal identification (ID is an important symbol of personhood)

• Many require help with completing their taxes

• Many require help filling out forms

• Many report major components of their diet missing (malnutrition)

• Many require help with obtaining supported and affordable housing (multiple evictions and housing exclusion)

• Many do not have the basic necessities of life: clothing, bedding, furniture or cooking utensils

• Many do not have a bank account (financial exclusion)

• Many are not able to be compliant to excessive rules, policies and procedures

Insite fits into a range of PHS programs including: financial services, a Drug Users Resource Centre, adentalclinic, two medical clinics, an art gallery, a grocery store, a community based antibiotic program and a range of employment and social enterprises. The supported housing stock of the PHS encompasses approximately 1000 units including operational projectsas well as those under development. Through its services, the PHS reaches approximately 10,000 vulnerable individuals who are homeless or at risk of homelessness each year and comes into contact with almost **every **person who lives in an SRO in the DTES community. It is precisely the "hardest to reach", "hardest to treat" and "hardest to house" group that the PHS aims to reach with low-threshold programs like Insite: vulnerable individuals who have limited or no other healthcare options. The decision of the PHS to launch the legal case to protect the SIF was an attempt at preventing this group from being further neglected, forgotten and pushed into the shadows of society.

## Attorneys for the vulnerable and forgotten

Monique Pongrecic-Speier, a partner in the firm Schroeder Speier, has been the lawyer of record for the PHS for a number of years. She is an award winning lawyer [63] and has been involved in a number of socially conscious legal realms including the protection of workers'. [64] and human rights. [65,66] throughout her career. Early on, as political events threatened the fate of Insite unfolded, she was quick to make the commitment to defend this important part of BC's healthcare system pro bono publico. She compiled and reviewed the majority of the initial evidence for the case, in the form of interviews, affidavits, official documents, comprised of thousands of pages, which she prepared for the legal team. She argued the inter-jurisdictional component of the case.

F. Andrew (Drew) Schroeder, also partner in the firm Schroeder Speier, is a former Rhodes scholar who has been involved in many high profile cases including a breakthrough victory in the BC Supreme Court for injured workers. [67,68]. He also represented 49 descendents of Doukhobors who were separated, as children, from their families for years at a time. [69] He is considered to be one of the best lawyers representing workers rights in Canada. [70] In his role in the case, he argued the early part of the case and carried the team through the initial administrative sections of the case with regard to whether the case could be heard as a summary trial (relying on written documents) or as a full trial (relying on live witness testimony).

Joseph Arvay, Q.C., is an award winning lawyer, highly recognized for his social conscience, who has, according to the Canadian Bar Association, has "litigated many ground-breaking constitutional law cases" in Canada. [71] Mr. Arvay has been described by the President of the International Commission of Jurists, Madam Justice Michele Rivet, as "one of Canada's most tireless civil rights and human rights lawyers". [72] He has acted on behalf of gays and lesbians, BC Civil Liberty Association, First Nations, women involved in the sex trade, the disabled, laid-off mill workers and 400 Crown Prosecutors against the Province of British Columbia. [73] He has defended same sex marriage, academic freedom, Aboriginal fishing rights, and collective bargaining by unions as a right under the Canadian Constitution. He has fought against warrantless searches, high voltage power lines, affronts to freedom of speech and the privatization of healthcare. Some of his most famous cases include representation of the rights to free speech for a gay and lesbian bookstore, the protection of same sex-spousal benefits and the protection of the constitutional rights to collective bargaining for workers in government contracts, a case that he took all the way to the Supreme Court of Canada and won. He led the case on the Charter arguments regarding the rights to life, liberty and security of the person for people living with addictions who need Insite.

## Two people living with addiction

Addiction doesn't really happen in courtrooms; it happens in the lifeworld of everyday humans and their families. Knee-deep in personal and familial sorrow, people with addictions are often on the edge of psychosocial survival. To venture from the edge of existence in the inner city where Insite is located to the courtroom showed the greatest measure of personal courage in this legal case. When the lawyer finished each interview, told with painful honesty by wounded witnesses, an almost unbearable sadness blanketed each affidavit. The Government of Canada never contested the credibility or representivity of the two people with addictions that provided evidence about how they rely on Insite. What greater measure of courage than to share your personal experience with the healthcare issue of addiction, still deeply stigmatized, in the public realm? Many people in the community, especially those that rely on Insite for life-saving healthcare, are particularly grateful to Dean Wilson and Shelly Tomic for their tremendous social conscience and courage in sharing their stories for the betterment of others.

## The trial

On the first day and the last day of this legal case, people wept. The evidence in the case, as summarized in the Justice Pitfield's Reasons for Judgement, provided an depth history of the recognition of addiction as an epidemic in Vancouver and the government responses to it. [3]

During the trial, our legal team began to examine the notion of addiction as a healthcare matter. The lead lawyer for the Government of Canada rose in immediate response and stated for the record that Canada had no intention of disputing that addiction is an illness. The legal team for Canada had made a crucial concession: addiction is an illness. Nor could they have done otherwise, with any credibility, given that they had relied on evidence from selected experts in the field of addiction medicine.

The moment seemed historic when I attended the courtroom and looked into the eyes of Justice Pitfield. I wondered at the time, if he, too, felt the presence of an historical moment. Did Justice Pitfield know that he was on the verge of legal greatness? When the judgement was rendered, the answer was clear. Judge Pitfield was ever present in this case, he had heard every word, read every paper and he understood with clarity the truthfulness of this historical moment in law.

In his Reasons for Judgement, Justice Pitfield notes that the Government of Canada and the plaintiffs agreed on a crucial point: "drug addiction is an illness". [3] (p. 20). Furthermore, he concludes that all the evidence put forward three incontrovertible facts:

1. "Addiction is an illness. One aspect of the illness is the continuing need or craving to consume the substance to which the addiction relates.

2. Controlled substances such as heroin or cocaine that are introduced into the bloodstream by injection do not cause Hepatitis C or HIV/AIDS. Rather, the use of unsanitary equipment, techniques and procedures for injection permits the transmission of those infections, illnesses or diseases from one individual to another; and

3. The risk of morbidity and mortality associated with addiction and injection is ameliorated by injection in the presence of qualified health professionals."[3] (p. 33, para. 87)

Furthermore, Justice Pitfield concludes, on the basis of the evidence, that the SIF is a healthcare facility:

"While users do not use Insite directly to treat addiction, they receive services and assistance at Insite which reduce the risk of overdose that is a feature of their illness, they avoid risk of being infected or of infecting others by injection and they gain access to counselling and consultation that may lead to abstinence and rehabilitation. All of this is healthcare."[3](p. 51, para. 136)

He also addresses moral arguments, popular with detractors against harm reduction measures that are sometimes, mistakenly, believed to somehow condone addiction:

"Society cannot condone addiction, but in the face of its presence it cannot fail to manage it, hopefully with ultimate success reflected in the cure of the addicted individual and abstinence". [3](p. 54., para. 144).

He takes this notion further to examine the process of condemnation in addiction while drawing analogy to other, less stigmatized, conditions:

"Denial of access to Insite and safe injection for the reason by Canada, amounts to a condemnation of the consumption that lead to addiction in the first place, while ignoring the resulting illness. While there is nothing to be said in favour of the injection of controlled substances that leads to addiction, there is much to be said against denying addicts healthcare services that will ameliorate the effects of their condition. While those are not prohibited substances, society neither condemns the individual who chose to drink or smoke to excess, nor deprives that individual of a range of healthcare services. Management of the harm in those cases is accepted as a community responsibility. I cannot see any rational or logical reason why the approach should be different when dealing with narcotics, an aspect of which is that the substance that resulted in the addiction in the first place will invariably be ingested in the short-term, and possibly the long-term, because of the very nature of the illness. Simply stated, I cannot agree with Canada's submission that an addict must feed his addiction in an unsafe environment when a safe environment that may lead to rehabilitation is the alternative" (p. 155–156, para 146).

Justice Pitfield infers that the management of harm from addiction is a community responsibility. Addiction is, then, a public problem demanding public resources and responsibility. He goes on to conclude that failure to protect the staff of Insite from criminal prosecution through the Controlled Drugs and Substances Act (CDSA) for performing their duties in the healthcare program is contrary to the Charter of Rights and Freedoms of Canada (Charter) that protect, under section 7, their fundamental right to life, liberty and security of the person. Even more important than liberty, he asserts, is the threat to life and security of the person of people with active addictions if Insite were to be arbitrarily closed by the federal minister of health.

Moreover, Justice Pitfield notes that the CDSA, as it pertains to the SIF, is actually incongruent with the state's interest:

"In particular, it prohibits the management of addiction and its associated risks at Insite...Instead of being rationally connected to a reasonable apprehension of harm, the blanket prohibition contributes to the very harm it seeks to prevent. It is inconsistent with the state's interest in fostering individual and community health, and preventing death and disease." (page 56–57, para. 152)

In his reasons for judgment, he also provides critique of the unencumbered and blanket power over the program by the federal Minister of Health and his failure to examine the SIF in relation to the public interest:

"The unfettered nature of the discretion to exempt is apparent in this case. Following a detailed assessment of medical and social need, the Health Authority applied for an exemption that would permit Insite to operate. The heading under which the Minister granted the exemption was 'necessiity for a scientific purpose'. No reference was made to necessity for a medical purpose. No reference was made to necessity in the pubic interest, which, in the context of the DTES, was the over-riding concern." (p58, para 155).

He emphasizes that the CDSA cannot take precedence over the Charter rights of users who rely on Insite. As such, he pronounced that sections 4(1) and 5(1) CDSA as applied to Insite "are inconsistent with s. 7 of the Charter, and have no force and effect."[3] (p. 58, para. 158)

Furthermore, Justice Ian Pitfield makes the observation that the injection of drugs by marginalized people with multiple barriers to their social and medical tenure is not recreational:

"Residents of the DTES who are addicted to heroin, cocaine and other controlled substances are not engaged in recreation. Their addiction is an illness frequently, if not invariably, accompanied by serious infections and the real risk of overdose that compromise their physical health and health of other members of the public. I do not assign or apportion blame, but I conclude that their situation results from a complicated combination of personal, governmental and legal factors: a mixture of genetic, psychological, sociological and familial problems; the inability, despite serious and prolonged efforts, of municipal, provincial and federal governments, as well as numerous non-profit organizations, to provide meaningful and effective support and solutions; and the failure of the criminal law to prevent the trafficking of controlled substances in the DTES as evidenced by the continuing problem of addiction in the area."[3] (p. 33–34, para. 89)

His analysis reaches far beyond the simple process of blaming addicts for their condition towards a more complicated understanding of addiction and the factors that affect it. The Judgement of Justice Pitfield has shown the way to develop kindness in human civilization, as it should pertain to those struggling with addiction, a little further.

## The tables are turned

In the end, Justice Pitfield provided the Government of Canada with one-year, ending 30 June 2009, during which time the CDSA must be brought into compliance with the Charter otherwise the law will become constitutionally invalid. During that one year period, he granted the "users and staff at Insite, acting in conformity with the operating protocol now in effect, a constitutional exemption from the application of ss. 4(1) and 5(1) of the CDSA."[3] (p.59, para. 159)

Until the completion of this legal case, the PHS, the VCH and the Province of British Columbia had to apply for a Section 56 exemption, for scientific purposes, from the CDSA. As such, those that rely on the program and the staff who serve them had been constantly subject to the political whims of the Prime Minister and his health minister to determine the fate of this crucial healthcare program. With the Pitfield decision, instead of waiting with their hearts in their hands for the outcome of the cabinet discussions of Harper and Clement, it is now the federal government that has a tight timeline looming over its head. If the parliament does not bring the CDSA into compliance with the Charter by 30 June 2009, then the authority of act evaporates.

We are now standing on the legal shoulders of Justice Ian Pitfield. As it stands, then, the VCH and PHS has a permanent exemption to operate Insite. What is required now is a permanent removal of the SIF from the Controlled Drugs and Substances Actso that recurrent politicization of serious addiction through this federal act can be eliminated. That is, of course, unless the conservative federal government can set the clock back by having Mr. Pitfield's decision overturned by the BC Court of Appeal in April 2009.

## Back to Darrow

I opened this commentary with a reference to American lawyer Clarence Darrow, famous for legal fight for freedom of speech and his defence of the damned. His work is relevant to the present case in three respects. Firstly, his incredible dedication to defending the poor, the damned and the unpopular is particularly relevant here. Defending people with active addictions, who are still deeply stigmatized, is not an automatically popular legal cause. The decision to defend this group of citizens has to be, by social necessity, done out of principle. Monique Pongracic-Speier, Drew Schroeder and Joseph Arvay showed remarkable legal conscience in taking up the case of people with addictions, like modern day lepers. [60,61].

The second connection to Darrow is the fact that defending the vulnerable and socially damned is not financially rewarding. It seems that some of the most important legal struggles, by virtue of their stigmatization, must be done for free. It is estimated that between thirty and fifty per cent of the people represented by Clarence Darrow did not have the financial means to pay any fee whatsoever. [2] The lawyers defending Insite in this case did so without payment. In fact, all the lawyers and expert witnesses in **defence **of Insite gave their time and testimony for free. For that, we are in debt for the generous contribution that they all made to the public good.

However, it was Darrow's tireless work against capital punishment that I draw the most important connection. The final link to Darrow pertains to capital punishment, state sanctioned and committed killing, an anachronistic and murderous act by the nation. Ignoring the scientific and medical evidence with regard to harm reduction measures, such as SIFs and syringe distribution programs, which are designed to address key epidemiological aspects of the pandemic of addiction, is to allow the State to unreservedly condemn addicts to death by preventable causes. Fatal drug overdose is not like some healthcare challenges, like cancer, for which there are no known cures. There is a cure for fatal drug overdoses; they can be prevented when injections are medically supervised. Deadly HIV and hepatitis infections can be curbed by initiatives that reduce deadly syringe sharing. Closing the door on harm reduction measures is to condemn addicts to an epidemiological death row. This state condemnation, like capital punishment, is something that is even more calculated than murder:

"I have always hated capital punishment. To me, it seems a cruel, brutal, useless barbarism. The killing of an individual by another always shows real or fancied excuse or reason. The cause, however poor, was enough to induce the act. But the killing of an individual by the State is deliberate, and is done without personal grievance or feeling. It is the outcome of long pre-meditated hatred. It does not happen suddenly, without warning, without time for the emotions to cool and subside, but a day is fixed a long time ahead, and the victim is kept in continued prolonged torture up to the moment of execution". [2] (pp. 49–50)

Clarence Darrow was relentless in his pursuit of the immortal goal of kindness towards all persons, including the most vulnerable and weak in our midst. The Judgement by Justice Pitfield is reminiscent of that goal. With what we know in science, medicine and now law, state refusal to accept injection facilities and other harm reduction measures such as Insite as part of a comprehensive approach to addiction is, plainly, a form of implicit capital punishment of the addicted by means of fatal overdose, hepatitis and AIDS. Perhaps, with this Judgement of Justice Pitfield, we are at the beginning of the end of the deadly fervour that accounts for addiction's death row and drives political anger towards addicts.

## The hard-hearted appeal: where do we go from here?

Two days after the decision by Justice Pitfield on 27 May 2008, federal health minister Tony Clement announced that he would direct the federal justice minister to attempt to have Justice Pitfield's landmark decision overturned by the BC Court of Appeal. BC Supreme Court decision. [74] He then launched a full offensive attack on Insite. In the parliamentary committee on healthcare on 29 May 2008, he attempted to undermine the scientists who have evaluated Insite:

"On the question of science, let me assure you I've read many of the studies that have been published on Insite. These studies have the weight of publication, as well as some articulate proponents who insist their positions are the correct one. Many of the studies are by the same authors who, quite frankly, plow their ground with regularity and righteousness. Indeed, while in our free society scientists are at liberty to become advocates for their position, I've noticed that the line between scientific views and advocacy is sometimes hard to find as the issue on Insite is developed". [75] (pp. 30–31)

Clement declares to the Parliamentary Committee that one of the activities performed by Insite is to "facilitate injection drug use". [75] (p. 48). As someone that was intricately involved in the development and implementation of Insite, I can say with conviction that nothing could be further from the truth. I have known people, personally, to die of fatal overdoses or AIDS and neither I nor anyone who is involved in the operation of Insite promote, facilitate or glamorize injection drug use in any way conceivable. This program facilitates life in a healthcare facility as an alternative to a lonely death in an ugly alleyway. In fact, no one in their right mind, and certainly not people living with addictions or the people that love them, would want to facilitate drug use. Minister Clement then rejects the decision of Justice Pitfield and calls into the question the very notion of the SIF as healthcare:

"In my opinion, supervised injection is not medicine; it does not heal the person addicted to drugs. Each and every injection, along with the heroin and cocaine injected, harms the person. Injection not only causes physical harm, it also deepens and prolongs the addiction.". [75] (p. 36)

In a letter to the Globe and Mail on 5 June 2008, he attacked physician Gabor Mate, who testified in support of Insite, calling him hypocritical:

"A more apt analogy of what Insite, Vancouver's safe-injection facility, does would be a doctor holding a cigarette to make sure a smoker doesn't' burn his lips, or watching a woman with cardiac problems eat fatty French fries to ensure she swallows them properly. Given that doctors are ethically bound to do no harm, the idea of one doctor or a community of doctors advocating for activities that cause harm is disturbing. It is also hypocritical, given that a doctor suffering from drug addiction in Canada would automatically be referred to a treatment program based on abstinence; no addicted doctor would be referred to a supervised injection site and told: 'Keep injecting until you are ready for treatment"'. [76]

It seems impossible for someone to have made these comments that had actually read Justice Pitfield's judgement in its entirety and understood it. So, then, the federal health minister appears to draw similarity between saving the life of person from a fatal drug overdose in an SIF to watching a person with a cardiac condition eat French fries. Perhaps, if he had not had his power to close Insite taken away by Justice Pitfield, he could have closed the facility happily and then explained this parallel to a mother who has just lost her daughter to a preventable overdose? If there were, in fact, a hypothetical physician living in the systemic refugee camp of the DTES in Vancouver who was addicted to injecting heroin, then, in contrast to the Minister's lofty ruminations, we would definitely grant them access to Insite to keep them from dying in an alleyway. And, as a result, Insite would serve as a doorway into treatment and healthcare for the physician.

## The future

Within 48 hours of Justice Pitfield's decision, federal Health Minister Tony Clement reacted by announcing that Canada would attempt to have Mr. Justice Pitfield's decision overturned by the BC Court of Appeal. The legal team for Insite received official notice of the Canada's aim to have the decision on 3 June 2008. Accordingly, the PHS legal team provided notice of Cross Appeal on 12 June 2008. In taking only 48 hours to weigh up the complex decision of Justice Pitfield, what does this political decision reveal about the analysis performed byPrime Minister Harper and health minister Clement regarding the medical, scientific and, now, the legal wisdom regarding SIFs?

The federal conservatives appeared to be attempting to usedemagogy regarding addiction to garner political support when a pamphlet was mailed out in August of 2008 using free postage privileges for members of parliament. The pamphlet featured a picture of a needle in a playground with a swing and children playing in the background. The documentstated, "junkies and drug pushers don't belong near children and families. They should be in rehab or behind bars." [see Additional File 6]. A formal complaint from opposition Members of Parliament ensued on the grounds that rights to free postage for federal politicians in Canada does not allow requests for re-election. [77] The Conservative pamphlet asked recipients to fill out a ballot and send it back to Ottawa to the attention of a conservative Member of Parliament (who is Co-Chair of the Canada-United States Inter-Parliamentary Group) using free postage.

It appears from their decision to attempt to overturn the findings of the BC Supreme Court that the Prime Minister Harper and health minister are once again going to use the resources of the federal government to attempt to stand against science, medicine and law in their attempt to prevent the medical supervision of deadly injections. It is hard to understand what reasoning might account for such a cold decision.

In sharp contrast, the Premier of the province of British Columbia, Gordon Campbell has shown remarkable leadership, along with his health minister George Abbott and attorney general Wally Oppall (former BC Supreme Court Judge), in funding and protecting Insite as part of the continuum of healthcare for vulnerable populations in the province. Moreover, the Attorney General of British Columbia has now officially entered the next stage of the legal case by exercising the right of the Province of BC to be a party to the appeal. In fact, a second ministry of the Province, the Vancouver Coastal Health Authority, have also signalled their intention to enter the legal case to defend the SIF.

It is now time for the conservative government of Canada, under the leadership of Prime Minister Stephen Harper, to do the right thing and to bring the CDSA into compliance with the Charter. Justice Pitfield has given them one year to do so. Only a few months are left. While the clock ticks on the CDSA, Prime Minister Harper and federal health minister Clement are missing an important historic opportunity to rise to the challenge of Justice Pitfield. They could be political heroes in the story by showing leadership by joining scientists, physicians and population health experts in moving the country with a comprehensive approach to injection drug use. Prime Minister Harper and health minister Clementcould work together with the other parties in the parliament, in a non-partisan spirit, to remove SIFs from the CDSA. In so doing, they wouldrepresent the will of the vast majority of Canadians and demonstrate our country'shealthy respectfor scientific, medical, legal and humanistic approaches to the pandemic of addiction.

For now, we are, sadly, going to back to court in the spring of 2008. Once again, the PHS, the two people living with addiction and three lawyers have been thrust into a legal gale originating from our nation's capital. The federal government's determination to overturn the rights of Insite to operate appears to be driven by stubborn ideology. The community is forced, once again, to fight for life-saving healthcare for people with addictions and their families. But, at least, we are trimming our sails to the legal wind that has been provided by Justice Pitfield. Even as the Prime Minister and federal health ministeronce again dispatch the resources of the federal government against Vancouver's SIF, we still live in hope for a humane and evidence based approach to addiction for the children of tomorrow. We are hopeful, for the wounded addicts that rely on Insite, that we will one day reach a kinder and more humane destination where the rights to liberty, freedom and security of the person for people living with addiction are a part of fundamental justice. Thank you Justice Pitfield for giving us hope to see beyond addiction's death row:

"Who hopes for only for what they see before them?

*For hope that is seen is not hope at all"*.

Paul to the Romans 8

## Supplementary Material

Additional file 1 **The Honourable Mr. Justice Pitfield Reasons for Judgment: PHS Community Services Society v. Attorney General of Canada, 2008 BCSC 661.** The legal judgment pertaining to North America's only SIF. Click here for file

Additional file 2 **Report of the Task Force into illicit narcotic overdose deaths in British Columbia .** The results of a provincial task force examining fatal overdoses due to injection drug use. Click here for file

Additional file 3 **Health impact of injection drug use and HIV in Vancouver.** A report on the epidemic of injection drug use in the Vancouver area commissioned for the local health authority. Click here for file

Additional file 4 HIV, Hepatitis, and injection drug use in British Columbia: pay now or pay later? A report on the population health impacts of injection drug user by British Columbia's Chief Medical Health Officer. Click here for file

Additional file 5 **Framework for action.** This is the drug policy document for City of Vancouver. Click here for file

Additional file 6 **Junkies and drug pushers don't belong near children and families.** This is a pamphlet mailed out compliments of Rob Merrifield, Member of Parliament, in August of 2008. Click here for file
