# Supplementary material for: Fighting addiction's death row: British Columbia Supreme Court Justice Ian Pitfield shows a measure of legal courage
Source: Harm Reduct J. 2008 Oct 28;5:31. doi: 10.1186/1477-7517-5-31 (PMC2611970; doi:10.1186/1477-7517-5-31)
Supplement: Additional file 1 — The Honourable Mr. Justice Pitfield Reasons for Judgment: PHS Community Services Society v. Attorney General of Canada, 2008 BCSC 661. The legal judgment pertaining to North America's only SIF. [file 1477-7517-5-31-S1.pdf]

IN THE SUPREME COURT OF BRITISH COLUMBIA

Citation: ***PHS Community Services Society v. Attorney General of Canada,*** 2008 BCSC 661

Date: 20080527
Docket: S075547
Registry: Vancouver

Between:

PHS Community Services Society,
Dean Edward Wilson and Shelly Tomic

Plaintiffs

And:

Attorney General of Canada

Defendant

\- and -

Docket: S065587
Registry: Vancouver

Between:

Vancouver Area Network of Drug Users (VANDU)

Plaintiff

And:

Attorney General of Canada and Minister of Health for Canada

Defendants

And:

British Columbia Civil Liberties Association

Intervenor

Before: The Honourable Mr. Justice Pitfield

Reasons for Judgment

Counsel for the Plaintiff: PHS Community Services Society, Dean Edward Wilson and Shelly Tomic	J.J. Arvay, Q.C.<br>F.A. Schroeder<br>M. Pongracic-Speier
Counsel for the Plaintiff: VANDU	J.W. Conroy, Q.C.
Counsel for the Defendant: Attorney General of Canada	J.J.L. Hunter, Q.C.<br>K.M. Stephens

Counsel for the Intervenor: British Columbia Civil Liberties Association	D.A. Webster, Q.C.<br>R.D.W. Dalziel
Date and Place of Hearing:	April 28 - May 2,<br>May 5-7, 2008<br>Vancouver, B.C.

I. Introduction

[1] When within the confines of the Vancouver Safe Injection Site (“Insite”), drug users not liable to prosecution for possessing a controlled substance contrary to s. 4(1) of the *Controlled Drugs and Substances Act*, R.S.C. 1996, c. 19 (the “*CDSA*”), or staff, for trafficking contrary to s. 5(1). Users and staff have been afforded exemptions by the Federal Minister of Health under s. 56 of the *CDSA*:

> 56. The Minister may, on such terms and conditions as the Minister deems necessary, exempt any person or class of persons or any controlled substance or precursor or any class thereof from the application of all or any of the provisions of the Act or the regulations if, in the opinion of the Minister, the exemption is necessary for a medical or scientific purpose or is otherwise in the public interest.

[2] The initial exemptions, based on necessity for a scientific purpose, were granted for a term of three years commencing September 12, 2003. They were subsequently extended to December 31, 2007, and then to June 30, 2008. If the ability to operate is dependent upon the exemptions and no further extensions are forthcoming, Insite will close its doors on June 30th.

[3] For that reason, PHS Community Services Society (“PHS”) and its co-plaintiffs, Mr. Wilson and Ms. Tomic, and Vancouver Area Network of Drug Users (VANDU) have commenced separate actions seeking relief that will obviate the need for the exemptions.

[4] PHS is a non-profit organization and registered charity whose main purpose is to provide housing and support to individuals in the Downtown Eastside (“DTES”) of Vancouver, individuals which it describes collectively as the “hard to house, hard

to reach or hard to treat". PHS describes its constituents as those who are homeless or at risk of homelessness due to multiple barriers to stable housing associated with a combination of unemployment, addiction, chronic illness and mental health problems.

[5] PHS operates Insite under a contractual arrangement with the Vancouver Coastal Health Authority (the "Health Authority"). In its action, PHS claims that:

> 1. Insite is a health care undertaking, authority for the operation of which lies with the Province.
>
> 2. The federal constitutional power to legislate with respect to criminal law cannot interfere with the provincial constitutional power with respect to health care because of the doctrine of interjurisdictional immunity.
>
> 3. In the alternative, ss. 4(1) and 5(1) of the CDSA are unconstitutional and should be struck down because they deprive persons addicted to one or more controlled substances of access to health care at Insite and therefore violate the right conferred by s. 7 of the *Charter of Rights and Freedoms* (the "*Charter*") to life, liberty, and security of the person, and the right not to be deprived thereof except in accordance with the principles of fundamental justice.

[6] VANDU is a non-profit society whose primary purpose as an advocate on behalf of drug users is to increase the capacity of addicts to live healthy lives by promoting local, regional and national harm reduction, education, intervention and peer support. VANDU seeks declarations that:

> 1. The conduct of the staff in the ordinary course of business at Insite does not involve the commission of any offences at law;
>
> 2. The *CDSA* and the regulations do not apply to the medical treatment at Insite of persons addicted to a controlled substance;
>
> 3. The offence of the possession of all addictive drugs as set out in Schedules I, II and III of the *CDSA* violates s. 7 of the *Charter*; and

4. Section 56 of the *CDSA*, which vests an unfettered discretion in the Minister to grant an exemption from the provisions of the *CDSA*, is unconstitutional.

[7] The Attorney General of Canada ("Canada") opposes the granting of any of the relief claimed in either action on the basis that:

1. The impugned provisions of the *CDSA* are valid federal law and are not subject to “reading down” or other limitation as alleged by the plaintiffs.
2. The doctrine of interjurisdictional immunity has no application.
3. Insite only operates lawfully because of the ministerial exemptions granted under s. 56 of the *CDSA*;
4. No interest in life, liberty or security of the person under s. 7 of the *Charter* is engaged;
5. If a s.7 interest is engaged, the plaintiffs have not been deprived of any such interest contrary to the principles of fundamental justice because the application of valid criminal prohibitions in respect of the possession and trafficking of illicit drugs to the plaintiffs is not arbitrary, overly broad, grossly disproportionate, or otherwise constitutionally objectionable;
6. In the further alternative, if the plaintiffs’ s. 7 rights have been infringed, any such infringement is saved by s. 1 of the *Charter*.

[8] Because of the common issues raised by the actions, they were heard together.

II. Preliminary Objection to Summary Trial

[9] The plaintiffs set these actions for hearing by way of summary trial on affidavit evidence pursuant to the Rules of Court. At the commencement of the hearing, Canada applied for an order declaring that the actions were not appropriate for summary trial and disposition because the complexity of the evidence, the appearance of material conflicts in the numerous affidavits, and the importance of the issues required the in-court testimony of witnesses.

[10] After considering the submissions of all counsel, I concluded that I could not finally rule on Canada's objection with fairness to the parties without considering all of the affidavit evidence and hearing the submissions of counsel in relation to it. I adjourned Canada's application and invited counsel to renew it, should they wish to do so, in the course of final submissions. I also advised the parties that I was bound by Rule 18A(11) in any event, and would not dispose of the actions if I concluded that I was unable, on the whole of the evidence before the court on the summary trial applications, to find the facts necessary to decide the issues of fact or law.

[11] At the close of eight days of hearing, counsel for Canada submitted that the actions could and should be decided by means of this summary trial, provided that I did not find it necessary to make findings of fact in relation to various matters of science on which there are conflicts in the evidence.

[12] I have concluded that the affidavit evidence and the course of argument enable me to decide the relevant issues of fact and law that are essential to the disposition of the actions, and that it is appropriate to proceed by means of the summary trial process. It follows that Canada's application to dismiss the plaintiffs' summary trial applications on the basis that the issues are not suitable for disposition in that manner is dismissed.

III. Historical and Operating Context

A. The Downtown Eastside and the Origin of Insite

[13] The character of the DTES and the context in which Insite emerged are central to an understanding of the issues raised by these actions.

[14] PHS tendered the affidavits of Mr. Donald McPherson, Vancouver's Director of Drug Policy, and Ms. Heather Hay, Director of Addiction, HIV/AIDS and Aboriginal Health Services for the Health Authority. Their evidence describing the DTES and the origin of Insite was not challenged by Canada. I accept their evidence regarding the social context. The summary that follows is based on that evidence. However, I am not to be taken to have necessarily accepted the admissibility of any opinions contained in their affidavits. I will consider the admissibility of those opinions and their relevance to the issues before me when necessary or appropriate to do so.

[15] The DTES is generally regarded as the area bounded by the waterfront along Burrard Inlet on the north, Clark Drive on the east, Pender and Terminal Streets on the south, and Richards Street on the west. Ms. Hay described the DTES as "the home to the poorest postal code in Canada". The median household income in the area was approximately $12,900 in 1996 compared to the City average of approximately $48,000. The DTES is home to over 4,600 of the estimated 12,000 intravenous drug users in Vancouver. There is no dispute regarding the fact that numerous studies have noted the lack of adequate housing, the superabundance of single-room occupancy hotels, and the fact that the DTES is home to many

vulnerable people including urban aboriginals who live in poverty and transition, and individuals with serious mental illnesses.

[16] In its report of March 31, 2008, an Expert Advisory Committee (the "EAC"), constituted to report to the Federal Minister of Health, described the characteristics of approximately 1,000 users surveyed in the DTES as follows:

- have been injecting drugs for an average of 15 years;
- majority (51%) inject heroin and 32% cocaine;
- 87% are infected with Hepatitis C virus (HCV) and 17% with human immunodeficiency virus (HIV);
- 18% are aboriginal;
- 20% are homeless and many more live in single resident rooms;
- 80% have been incarcerated;
- 38% are involved in the sex trade;
- 21% are using methadone; and
- 59% reported a non-fatal overdose in their lifetime.

[17] The conditions that have prevailed in the DTES affecting the lives of many who live there have long been known to civic, provincial and federal authorities as well as police. Inestimable amounts of time, energy and money have been directed to the search for solutions. Despite the efforts and undoubtedly good intentions, the problems persist.

[18] Mr. Donald McPherson began his employment with the City of Vancouver in 1987 as the Education Programmer of the Carnegie Centre located in the heart of the DTES. He became director of the Centre in 1993. He moved to City Hall in

1997 to help develop a drug policy for the City. He became the City's Drug Policy Co-ordinator in 2000, his present position.

[19] Mr. McPherson described the circumstances that prevailed in the vicinity of the Carnegie Centre:

> …During my time at the Centre, I watched the public illicit drug use mushroom on the very steps of the Centre at the corner of Main and Hastings. By the time I left the Centre, the top priority at the Centre was to contribute to the development of a plan to address the public use of illicit drugs and the rise in overdose deaths and spread of HIV/AIDS and Hepatitis C.

[20] Mr. McPherson attributes the heightened interest in intravenous drug use in the DTES to a 1994 report prepared by Coroner J.V. Cain entitled *Report on the Task Force into Illicit Narcotic Overdose Deaths in British Columbia*. The coroner's report was prompted by his investigation of overdose deaths in Vancouver which had risen from 16 in 1987 to 200 in 1993.

[21] Ms. Hay, like Mr. McPherson, identifies the coroner's report as the warning that an epidemic of drug-related deaths had developed in British Columbia by 1995.

[22] In 1996, Dr. Whynot, then Vancouver's medical health officer, issued a report describing the major impacts of injection drug use on the health system in Vancouver. Dr. Whynot concluded that injection drug use was leading to an increased incidence and prevalence of symptomatic infectious disease including HIV/AIDS, Hepatitis A, B and C, and skin- and blood-borne infections; frequent drug overdoses resulting in significant morbidity and mortality; increased hospital and emergency service utilization such as treatment for HIV-related disease, septicaemia

and endocarditis; increased ambulance responses and emergency room visits in response to drug overdoses; fetal exposure to addictive substances with both short-term and long-term consequences; increased pressure on all community-level outreach nursing and medical services; an increased need for community-level hospice palliative care; and worsening consequences for associated conditions such as mental illness.

[23] In 1996, the British Columbia Centre for Excellence in HIV/AIDS based at St. Paul’s Hospital, Vancouver, reported an HIV/AIDS epidemic in the DTES. In 1997, the Health Authority’s predecessor reported that the spread of Hepatitis C in the DTES had reached epidemic proportions.

[24] By early 1997, researchers at the Center released a research report indicating that the prevalence rate of HIV/AIDS in the DTES was then approximately 27% among injection drug users. The researchers concluded that the HIV/AIDS prevalence rate in the DTES was indicative of epidemic levels of infection. The Health Authority's predecessor accepted the Centre's research as scientifically sound.

[25] In the spring of 1997, the Province of British Columbia announced that it would allocate $3 million to combat the spread of HIV in the DTES. In the summer of 1997, a report to the provincial Minister of Health indicated that the HIV/AIDS epidemic in the City of Vancouver was focused in the DTES and that the disease was spreading most rapidly among individuals who were, or were likely to become, street involved or involved with injection drug use. The report observed that those

engaged in injection drug use in the DTES were frequently afflicted by multiple health issues: HIV/AIDS, Hepatitis C, Tuberculosis, alcohol and drug addiction, and mental illness. In addition, the report noted that many individuals coped simultaneously with poverty, a lack of affordable housing, a lack of transportation, inter-generational abuse and violence, and poor access to services.

[26] In September 1997, the Chief Medical Health Officer of the Vancouver Health Board declared a public health emergency in the DTES in relation to the escalating rate of HIV infection. In October 1997, the Health Board adopted the *Vancouver Downtown Eastside HIV/AIDS Action Plan*, which introduced several harm reduction strategies including the development of VANDU, targeted prevention programs, expanded needle exchange, and drug overdose management. The goal of the action plan was to keep drug users alive, contain the communicable disease epidemics, and stop others from being infected.

[27] In 1998, the provincial Health Officer published a report entitled *HIV, Hepatitis and Injection Drug Use in British Columbia - Pay Now or Pay Later*. The report recommended local action including consideration of a harm reduction approach.

[28] In 1999, Ms. Hay was directed to head a team to investigate the broader health situation in the DTES. Ms. Hay described the findings in her affidavit as follows:

> After broad-based consultation with national and international experts, we found that several public policies combined with changes in available services and changes in the nature of available drugs, made this community [the DTES] the perfect storm for a continued public health crisis. Decentralization of mental health clients, over-representation of urban

> aboriginal clients, withdrawal and closure of the addiction/mental health treatment services, introduction of crack cocaine to the neighbourhood, limited access to housing, limited access to clean needles, and poor access to primary health care led to a severe burden of illness and disease. Combined with poor, if any, access to washrooms, hand washing and food security, these circumstances continued to fuel an already extremely critical situation. The combination of expanded drug distribution and use, unstable housing, poor access to treatment and vulnerable client populations called for a much larger intervention than what had been previously realized through the HIV/AIDS Action Plan.

[29] The team reached the following conclusions: HIV/AIDS was at epidemic levels in the DTES; there was an epidemic of transferable Tuberculosis attributable to the compromised health status of injection drug users and impoverished housing condition in the DTES; there was a serious syphilis outbreak among female sex-trade workers, the preponderance of whom were also drug addicted and engaged in survival prostitution to feed their drug habits; there were outbreaks of Hepatitis A and B in the DTES attributable to the compromised immune systems of injection drug users who occupied single-room occupancy hotel rooms, often on a shared basis, with extremely limited, if any, access to hand washing or toilet facilities, there often being only one washroom available to be shared among 80 to 90 residents; there was a Hepatitis C epidemic (90% infection rate) associated with needle sharing; and the drug overdose epidemic continued among injection drug users, producing fatal and non-fatal overdoses.

[30] Ms. Hays' team concluded that injection drug use ought to be a primary focus of the Health Authority's efforts in the DTES. They encouraged the development of an integrated health approach built on four pillars: the expansion of primary care services in the DTES; the development of creative interventions to address communicable diseases, including mass immunization and treatment; the

development of a continuum of alcohol and drug services including expanded innovative harm reduction strategies, targeted prevention strategies, and short-term and long-term residential health care treatment services to be developed within and outside the DTES; and improved access to stable housing for individuals living in the DTES.

[31] In March 2000, the City, the Province and the Federal Government, entered into *The Vancouver Agreement*, which provided for cooperation and funding among the three levels of government in order to assess the City's critical needs. The DTES was identified as the number one priority. With the benefit of funding derived through *The Vancouver Agreement*, and in accordance with the integrated health approach, the Health Authority began to implement core addiction services, particularly in the DTES.

[32] *The Vancouver Agreement* stated that the goal for the DTES was to create a healthy, safe and sustainable community. The strategy by which the goal would be pursued involved initiatives in community health and safety, economic and social development, and community capacity building. The community health and safety component was described in the following terms:

> Proposed Components of the Downtown Eastside Strategy
>
> The Strategy proposes three equal components: Community Health and Safety, Economic and Social Development, and Community Capacity Building.
>
> 1. Community Health and Safety
>
> *1a. Primary Health Care*

Residents in the Downtown Eastside should have access to coordinated, high quality, primary health care. Effective linkages between health care and social services programs will be promoted. Strategies which promote programming where outcome evidence demonstrates effectiveness will be developed in collaboration with the Vancouver/Richmond Health Board.

The following are some objectives:

- Improve the health status of residents in the Downtown Eastside.
- Reduce the need for emergency and crisis interventions.
- Improve access to hospital care.
- Reduce the spread of HIV/AIDS and other infectious diseases.
- Reduce preventable deaths.
- Increase service integration to improve continuity of care.
- Increase the proportion of residents receiving regular primary care.

1b. Comprehensive Substance Misuse Strategy

Substance misuse is a health and social issue which is best addressed through collaborative health and social programs that get at the root causes of substance misuse.

An effective substance misuse strategy consists of a continuum of services from prevention and education to treatment and rehabilitation. A key feature is to build a range of comprehensive addiction services including a continuum of innovative approaches to reducing harm. Links to primary health care, housing, education and training, and employment are critical to the success of this strategy.

The following are some objectives:

- Decrease preventable deaths related to substance misuse.
- Reduce incidence of communicable disease associated with injection drug use.
- Reduce preventable harm associated with alcohol and other substance misuse.
- Reduce overall costs to the economy related to substance misuse,
- Enhance integration of services to address the social causes of substance misuse.
- Enhance prevention initiatives for all age groups.
- Increase public awareness and education to reduce harm.
- Promote rehabilitation options.
- Reduce criminal activities associated with substance misuse.

[33] A document entitled *A Framework for Action - A Four Pillars Approach to Drug Problems in Vancouver: Prevention, Treatment, Enforcement and Harm Reduction*, was released in April 2001.

[34] The report, of which Mr. McPherson was the principal author, introduced and described the four pillars concept:

> The following briefly describes the four pillars:
>
> **Prevention** involves education about the dangers of drug use and builds awareness about why people misuse alcohol and drugs and what can be done to avoid addiction. *A Framework for Action* supports coordinated, evidence-based programs targeted to specific populations and age groups—programs that focus on the causes and nature of addiction as well as on prevention.
>
> **Treatment** consists of a continuum of interventions and support programs that enable individuals with addiction problems to make healthier decisions about their lives and move towards abstinence. These include detoxification, outpatient counselling and residential treatment, as well as housing, ongoing medical care, employment services, social programs, and life skills.
>
> **Enforcement** strategies are key to any drug strategy. In order to increase public order and to close the open drug scene in the Downtown Eastside, more effective enforcement strategies will include a redeployment of officers in the Downtown Eastside, increased efforts to target organized crime, drug houses and drug dealers, and improved coordination with health services and other agencies to link drug and alcohol users to available programs throughout Vancouver and the region.
>
> In order for *A Framework for Action* to increase public order, it requires the collaboration of various enforcement agencies such as the Vancouver Police Department, RCMP, the newly created Organized Crime Agency, probation services, and the courts with the other programs and agencies involved in each pillar.
>
> **Harm Reduction** is a pragmatic approach that focuses on decreasing the negative consequences of drug use for communities and individuals. It recognizes that abstinence-based approaches are limited in dealing with a street-entrenched open drug scene and that the protection of communities and individuals is the primary goal of programs to tackle substance misuse. *A Framework for Action* attempts to demonstrate the need for harm reduction by outlining, and drawing upon, other successful programs around the world that have significantly reduced both the negative health and societal impacts and the costs of drug addiction.

[35] In relation to harm reduction the report said the following:

> If we are going to implement a successful drug strategy in Vancouver, we must acknowledge the need for harm reduction programs and realize that accepting harm reduction as part of the strategy does not mean condoning the use of illicit drugs. It means accepting the fact that drug use does and will occur - and accepting the need to minimize the harm this has on communities and individuals. It means recognizing that abstinence-based strategies are often impractical and ineffective in dealing with the street entrenched drug scene.

[36] The report stated that harm reduction was a pragmatic approach with the overall goal of reducing harm to communities and individuals; harm reduction involved establishing a hierarchy of achievable goals which, taken one at a time, step by step, could lead to a fuller, healthier life for drug users and a safer, healthier community for everyone; harm reduction recognized that abstinence may not be a realistic or desirable goal for certain users, particularly in the short-term; and harm reduction must include a law enforcement strategy to remove addicts from streets and alleys and into health services.

[37] Mr. McPherson deposed to the investigation undertaken in the course of developing the plan:

> In developing the plan, I investigated experience elsewhere. National and provincial sources could not offer much help - other than ultimately providing funding. I needed pragmatic approaches to the local problem of inner city decay and drug addiction. Frankfurt, Germany, Amsterdam, Netherlands, and Bern, Switzerland had already addressed the same problem that the City was facing in the Downtown Eastside, that is, an urban neighbourhood where drug use had created health care and public health issues of epidemic proportions. The plan was developed based on the experience of these other cities. My key question posed to the representatives of these cities was: what works?

[38] In developing the plan, Mr. McPherson worked closely with the Health Authority, to whom he attributed responsibility for the individual health care and public health components of the plan, part of which recommended expansion and de-centralization of needle exchange facilities which have now been established in community health clinics throughout the City.

[39] Mr. McPherson worked in close consultation with the Vancouver Police Department ("VPD") in developing the plan. The report stated that the VPD supported the establishment of Insite. The VPD affirmed its continuing support in a letter to the Minister of Health on April 21, 2006. There is no evidence to suggest that the VPD's support for Insite has diminished or evaporated.

[40] On May 15, 2001, the City formally adopted the plan contained in *A Framework for Action*.

[41] In the period from March 2000 through May 2003, needle exchange programs were expanded, a downtown community health centre was opened in the DTES, methadone service was expanded, and access to anti-retroviral drugs was enhanced.

[42] Part of the overall plan involved the integration of health care services. Prior to 2002, the Health Authority and its predecessor had limited ability to provide targeted alcohol and drug services to the community. The provincial Ministry of Health retained sole responsibility for adult alcohol and drug treatment services throughout the Province. In April 2002, the Ministry of Health transferred responsibility for those services to the regional health authorities. Ms. Hay deposed

to the fact that the change in mandate paved the way for the Health Authority to more wholly integrate addiction treatment into the spectrum of health care services delivered by it in the DTES. The Health Authority began its delivery of adult addiction services in October 2002.

[43] In September 2002, the Health Authority proposed a plan which would provide a continuum of services, including harm reduction and the development of multiple access points for clients with substance abuse issues. Ms. Hay described the model as a movement away from the traditional abstinence-based model for addiction services. Her affidavit evidence is instructive:

> Although [the Health Authority's] goal is to support clients to live a life free from substance abuse, we acknowledge that for many individuals it takes support across a continuum of services including harm reduction to realize that goal. The plan was aimed at assisting individuals with active addictions to make numerous small steps to reduce risky behaviours, improve their own health and achieve stability. The plan was also to protect the public from the spread of communicable disease. The plan proposed a continuum of care for drug users. The continuum of care was based on the premise that addictions services needed to expand the range of addiction harm reduction, prevention, intervention and recovery and that [the Health Authority] should facilitate access to addiction services at all points on the continuum rather than focussing [*sic*] predominantly on prevention and recovery as had been done in the past. The continuum envisioned increased services to both assist individuals to end drug use *and* to better protect their health during the active drug use. The continuum of care envisioned included supervised drug consumption sites. [Ms. Hay's emphasis]

[44] Ms. Hay described the supervised injection site initiative in the following terms:

> [The Health Authority] elected to pursue a supervised injection site to complement the other health care services developed pursuant to the Addiction Plan. In June 2001, at the convening of the last [Vancouver Richmond Health Board] board meeting, the board of directors voted to support Supervised Injection Sites (SIS) as a vital and necessary part of the continuum of the health care system. [The Health Authority's] decision was

> the product of extensive community consultations and research which led us to conclude that there was public demand for a safe injection site in Vancouver. Such a site would assist [the Health Authority] to meet its healthcare mandate in providing appropriate and necessary health care to all populations it serves. More specifically, [the Health Authority's] research suggested that a safe injection site would facilitate contact with high-risk injection drug users, provide means to reduce users' risk of [Injection Drug Use]-related health complications and death, and assist users to access other health and social services.

[45] In March 2003, the Health Authority approved the proposal for a safe injection site and authorized an application to Health Canada for an exemption under s. 56 of the *CDSA* that would permit it to open a safe injection site in the DTES, and to study the outcomes associated with its operation. Ms. Hay describes the Health Authority's goals for the site as follows:

> (a) provide a hygienic facility for injection drug users to inject their drugs under the supervision of a health care professional;
>
> (b) reduce the risk of overdose and the number of overdose deaths in the DTES;
>
> (c) reduce the number of ambulance calls to the DTES for overdose;
>
> (d) reduce the transmission of blood-borne pathogens including HIV and Hepatitis C;
>
> (e) reduce the incidents of potentially serious infections leading to conditions such as endocarditis and osteomyelitis;
>
> (f) reduce the incidence of soft tissue injuries associated with [Injection Drug Use], including abscesses and cellulites;
>
> (g) provide access to needle exchange and sterile injecting equipment;
>
> (h) provide referrals to other health and social service providers in the area;
>
> (i) connect participants with peer support services and increase opportunities for health and social networking;
>
> (j) increase public order; and
>
> (k) increase safety and security for the community.

[46] The exemption requested by the Health Authority was granted for a three-year term commencing September 12, 2003, extended shortly before it was due to expire to December 31, 2007, and further extended on October 2, 2007, to expire June 30, 2008.

B. The Nature of Addiction

[47] The plaintiffs and Canada agree on one thing: drug addiction is an illness.

[48] The Canadian Society of Addiction Medicine defines addiction as follows:

> A primary, chronic disease, characterized by impaired control over the use of a psychoactive substance and/or behaviour. Clinically, the manifestations occur along biological, psychological, sociological and spiritual dimensions. Common features are change in mood, relief from negative emotions, provision of pleasure, pre-occupation with the use of substance(s) or ritualistic behaviour(s); and continued use of the substance(s) and/or engagement in behaviour(s) despite adverse physical, psychological and/or social consequences. Like other chronic diseases, it can be progressive, relapsing and fatal.

[49] The nature of the illness was described in the affidavit of Dr. David Marsh which was tendered by PHS. Dr. Marsh is a licensed medical practitioner who holds specialist certificates from the International, American and Canadian Societies of Addiction Medicine. In addition to teaching addiction medicine and conducting research into innovative addiction treatments, Dr. Marsh is the Physician Leader for Addiction Medicine for the Health Authority; the Medical Director for Addiction, HIV/AIDS and Aboriginal Health Services for the Vancouver Community area of the Health Authority; the Division Head, Addiction Medicine, in the Department of Family and Community Medicine at St. Paul’s Hospital, Vancouver; and the Leader of

Addiction Research at the Centre for Health Evaluation and Outcome Sciences. In addition he is the physician responsible for the medical supervision of Insite.

[50] Dr. Marsh deposed to his opinion that the bio-chemical effects of narcotics contributed to addiction. In the case of opioids, including heroin, morphine and hydromorphone, the intensity of symptoms was a function of the extent to which the dose administered exceeded an individual's tolerance level. He described the symptoms of opioid administration:

> …Acute symptoms of opioid administration include: analgesia, sedation, euphoria, slowed heart rate, lower blood pressure, warm flushed skin, constricted pupils, uncoordinated movement and slowed respiratory rate. In a small number of individuals opioid administration can also unmask or trigger tics, repetitive automatic movements or choreoathetosis. In amounts far in excess of tolerance, heroin and other opioids can produce respiratory arrest, cardiovascular collapse and death.

[51] Dr. Marsh characterized cocaine and methamphetamine as stimulants and described the symptoms associated with their use:

> …The symptoms of acute administration of stimulants include: agitation, increased energy, a feeling of powerfulness or omnipotence, decreased appetite, decreased ability to sustain concentration, decreased sleep and rarely psychotic symptoms such as hallucinations and delusions. In very high amounts, stimulants can lead to cardiovascular complications such as arrhythmias, myocardial infarction or stroke any of which can cause death.

[52] Dr. Marsh deposed to the fact that these addictive substances affect the release of dopamine in the cells of certain parts of the brain. He described dopamine as the compound responsible for the transmission of a signal from one nerve cell to another in certain parts of the brain. He deposed to his opinion that the repeated administration of the addictive substances caused the brain to undergo

structural and functional changes that tended to counteract the acute effect of the drugs. The process of chronic change leads to tolerance such that the person using drugs must take higher amounts to experience the same effects, and the associated lower baseline in certain brain systems required an addict to take drugs in order that the level of function in those systems could be altered to approximate those of a non-addicted individual.

[53] Dr. Marsh deposed to his opinion that while the neuro-chemical effects of addictive substances partially explained addiction, genetic factors influence the risk of addiction, as do psychological and social determinants: stress, trauma, sexual and physical abuse, parental neglect and the effects of behavioural conditioning.

[54] Dr. Marsh described the process of withdrawal and deposed to his opinion that the combination of biochemical, psychological and social stresses that typically affect habitual drug users experiencing withdrawal put long-term users and “hard core” addicts at high risk of relapse when they attempt abstinence.

[55] Finally, Dr. Marsh deposed to his opinion that when the accumulation of biological, psychological and social factors are taken together and applied to a population of addicts such as those in the DTES, the route to long-term abstinence would be a long and difficult process characterized by many treatment attempts and a high, life-long risk of return to drug use:

> Rather than focusing on long-term abstinence as the only indication of success, therefore, a range of health interventions can be implemented which each contribute to improving the health and social functioning of these individuals by preventing adverse physical complications of drug use (such as HIV infection). …

[56] Canada responded to Dr. Marsh’s evidence with the affidavit of Dr. Frank Evans, an addiction specialist certified by the American Society of Addiction Medicine. He practises addiction medicine on a full-time basis and has worked and trained at eleven addiction treatment facilities. He is currently the President of the Canadian Society of Addiction Medicine responsible for the certification and training of physicians in addiction medicine. His curriculum vitae portrays a wide range of practice and involvement with addiction medicine with a special interest in the treatment of addiction in professionals, including health professionals and airline pilots. Neither his affidavit nor his curriculum vitae identifies the extent of his experience with persons who possess the genetic, psychological and sociological characteristics prevalent in the DTES.

[57] Dr. Evans did not dispute much of Dr. Marsh’s opinion. He deposed to the fact that some addiction medicine specialists, of which he was one, viewed abstinence-based treatment as the best course, while other specialists believed that “harm reduction” was a more realistic and practical therapeutic approach because abstinence may not be achievable for very chronic and severe addictions. He disagreed with Dr. Marsh’s opinion on the effect of prolonged ingestion on the brain and the life-long risk of relapse:

> I agree … that changes in the brain take place with repeated use of opioids that lead to increasing tolerance and the need for higher dosages to achieve a similar affect [*sic*]. However, increased tolerance reverses to normal levels after the person has been abstinent for some period of time.
>
> … I do not agree with [Dr. Marsh’s] proposition that opioid-addicted patients will likely continue to relapse over their lifetimes due to other permanent, irreversible and chronic brain changes from narcotic (opioid) usage. This

> propensity to relapse from any addiction has not been proved to be the result of permanent brain changes or neurosystem deficits.

[58] Dr. Evans deposed to his primary concern that the harm reduction as opposed to the abstinence-based approach can actually or potentially cause harm by enabling continuation of an addictive disease, and by delaying a potential change to a safer behaviour, such as abstinence.

[59] I do not need to attempt to finally assess or decide upon the merits of the competing positions regarding harm reduction and abstinence-based programs for addicts. The differing opinions point to the fact that in medicine, as in other disciplines, there is room for a difference in view. I do observe, however, that Dr. Marsh's experience with persons of the kind resident in the DTES is markedly greater than that of Dr. Evans who, as I have noted, is most closely associated with health care professionals and airline pilots, a significantly different group from injection drug users in the DTES. The correlation between Dr. Marsh's opinion and reality is reflected in the circumstances of the plaintiffs, Mr. Wilson and Ms. Tomic.

C. Dean Edward Wilson and Shelly Tomic

[60] Mr. Wilson and Ms. Tomic are residents of the DTES. They are described as representative users of Insite. Their affidavit evidence sets forth their personal histories and describes their illnesses. Canada did not challenge the evidence of either individual.

[61] Mr. Wilson is 51 years of age and unemployed. He first began using heroin when he was 12 years old. He has been injecting heroin for 38 years and cocaine

for 35 years. He has lived in the DTES for the last ten years and has resided in a single room at the Sunrise Hotel, a residential hotel run by PHS, since 2000.

[62] Mr. Wilson has enrolled in a detoxification program at least once a year for the past 37 years. He has participated in approximately 25 treatment programs. He has been on and off a methadone program for the last ten years, and in the last three years has been able to go without using heroin for periods of up to 30 days. When on methadone, he has continued to inject cocaine.

[63] Dr. Gabor Maté, who supervises Mr. Wilson's methadone program, has assessed Mr. Wilson as both physically and psychologically addicted to heroin and cocaine.

[64] In 2006, Mr. Wilson was president of the B.C. Association of Persons on Methadone. He served as the president of VANDU from 2000 until 2003. He was re-elected to the Board in September 2007. He was the Co-Chair of the Harm Reduction Action Society from 2002 to 2003 and sat on the Ministerial Council for HIV/AIDS for Canada from 2001 to 2004.

[65] Ms. Tomic is 39 years of age. She was born and raised in the vicinity of Calgary, Alberta, addicted to speed because her mother had been addicted to the substance throughout pregnancy. Ms. Tomic’s first experience with illegal drugs occurred when she was seven years old. A relative injected her with speed which she continued to use throughout her childhood.

[66] Ms. Tomic first came to Vancouver when she was 13 or 14 years of age. She continued to use speed and a combination of Talwin and Ritalin throughout her teens. She began injecting cocaine at the age of 19 or 20 when she was living in the DTES. An acquaintance gave her the first injection. Ms. Tomic resorted to prostitution in order to obtain funds to buy cocaine.

[67] Ms. Tomic first experienced heroin when she was 26 or 27 years of age. She bought drugs from a dealer believing she was buying cocaine. Shortly after injection, she realized that she had been provided with heroin which has since been her drug of choice, although she admits to smoking crack cocaine since it became readily available in the DTES.

[68] Ms. Tomic describes herself as one who has been a regular heroin user for about 12 or 13 years. She started using methadone in the spring of 2007, a step she attributes to Insite. As of January 2008, Ms. Tomic was using heroin only when, for one reason or another, she had been unable to obtain her methadone dose “on time”. She says she gets "dope sick" if she has neither methadone nor heroin. She describes dope sickness as follows:

> …Dope sickness is like having pneumonia, the flu and food poisoning all at once. It makes you feel sick to your stomach, sometimes to the point where you throw up and you get fever, chills and severe achiness. I have injected heroin when I have been dope sick to get functional again.

[69] Ms. Tomic has never overdosed to the point of losing consciousness. She has suffered from abscesses and skin problems over the years as a result of needle use. She developed endocarditis, a deep tissue infection that affects the heart. She

attributes the infection to the injection process. She was paralyzed by the infection for a period of two years and unable to walk. She has been able to regain the use of her legs but suffers from disabling arthritis.

[70] Ms. Tomic is Hepatitis C-positive, a fact she has known for approximately 15 years. She does not have HIV or AIDS.

D. The Operation of Insite

[71] Insite is located on East Hastings Street between Carrall and Main Streets. It is open daily from 10:00 a.m. to 4:00 a.m. the following day. The facility is known to DTES residents. Police refer addicts to it. Insite operates under an extensive and detailed operating protocol approved by Health Canada. It is staffed by a combination of PHS, Health Authority and community workers.

[72] Users must be 16 years of age or over, must sign a user agreement, release and consent form, must agree to adhere to a code of conduct, and cannot be accompanied by children. Users must register at each visit to the site and each is asked to identify the substance that will be injected. No substances are provided by staff. It goes without saying that the substances brought to Insite by users have been obtained from a trafficker in an illegal transaction. Users are obviously in possession of their substance en route to Insite. Approximately 60% of the drugs injected are opioids, of which two-thirds are heroin and one-third morphine or hydromorphone. Approximately 40% of injected drugs are stimulants, approximately 90% of which are cocaine and 10%, methamphetamine.

[73] Insite has 12 injection bays. Users are provided with clean injection equipment which is the only equipment that can be used at the site. Users are monitored by staff during injection. Nurses and paramedical staff provide treatment in the event of overdose and contact a physician and the ambulance service as necessary. Overdoses vary in severity and treatment.

[74] The protocol permits pregnant women to use Insite. They are required to undergo a more intensive assessment than others before being allowed access to the injection room. Those women are also referred to a clinic and child daycare facilities directly managed by the Health Authority, which provides pre- and post-natal care to pregnant women who are actively using illegal substances.

[75] Users who have completed an injection are assessed by staff. They may be discharged to the "chill-out" lounge or treated by a nurse in the treatment room for injection-related conditions. Users requiring extensive or ongoing care are referred to the closest primary care facility, either the Downtown Community Health Centre or the Pender Clinic.

[76] Staff and support workers interact with users at Insite on a one-to-one basis. Users are provided with health care information, counselling and referrals to Health Authority and other service providers. Records indicate that in 2005, 2006 and 2007, staff made 2,270, 1,828, and 2,269 referrals, respectively, to community clinic, hospital emergency, outpatient medical mental health, emergency shelter and community services; and to addiction counselling, housing, withdrawal, methadone treatment, drug recovery, and miscellaneous other services.

[77] Since the fall of 2007, the staff has also been able to refer users to "Onsite", a detox centre located above Insite which permits Insite to provide detox on demand. Onsite is a drug free environment supported by physicians who are addiction specialists and general practitioners, nurses and peers. Users may also be referred to residential detox and additional treatment services.

E. The Assessment of Outcomes

[78] Ms. Hay deposes to the fact that more than one million injections have occurred at Insite. Staff have managed in excess of 1,000 overdoses without any resulting fatalities to date. A variety of statistics is available on the total number of visits, the average number of daily visits, and the number of overdose interventions, nursing interventions, and referrals to other services. As with most statistics, the conclusions to be drawn from them are debatable. I see nothing to be gained by reproducing any of the raw data which is generally available in any event.

[79] The plaintiffs tendered affidavits from a number of medical practitioners who have endeavoured to assess the efficacy of Insite:

1. Dr. Thomas Kerr, assistant professor cross-appointed to the Departments of Medicine and Healthcare and Epidemiology at the University of British Columbia, co-principal investigator of the Scientific Evaluation of Supervised Injecting ("SEOSI") study pertaining to Insite, and the author of 22 peer-reviewed research papers documenting the impact of Insite;

2. Dr. Julio Montaner, professor of Medicine at the University of British Columbia, the Director of the BC Centre for Excellence in HIV/AIDS (the "Centre") at St. Paul's Hospital, Vancouver which was selected by Health Canada to evaluate Insite over a three-year period from 2003 to 2006; and

3. Dr. Evan Wood, a clinical assistant professor cross-appointed to the Departments of Medicine and Healthcare and Epidemiology, at the University

> of British Columbia, holder of a PhD. in Epidemiology and a research scientist at the Centre engaged in the SEOSI study.

[80] In addition to deposing to and describing some of the objective evidence accumulated over the period during which Insite has operated, these physicians deposed generally to their opinions that Insite serves a beneficial purpose.

[81] Canada tendered affidavits from persons in response:

> 1. Dr. Harold Kalant, M.D., PhD., Professor Emeritus in Pharmacology at the University of Toronto, and Director Emeritus (Biobehavioral Studies) at the Centre for Addiction and Mental Health; and
>
> 2. Nathan Lockhart, Senior Policy Analyst in the Drug Strategy and Controlled Substances Healthy Environment and Consumer Safety Branch of Health Canada.

[82] Neither individual deposed to any specific observations about Insite or their individual assessment of its efficacy. Dr. Kalant did not depose to any personal knowledge regarding Insite, or to involvement in any aspect of its operations. Mr. Lockhart's evidence was confined primarily to an outline of the evolution of Canada's drug policy.

[83] I do not propose to embark upon a review of the opinions expressed by any of the professionals on behalf of the plaintiffs or Canada with a view to resolving differences in order to decide where the balance lies. I do not doubt that there is room for divergent opinions in the debate about the efficacy of safe injection sites generally and Insite in particular. I do harbour doubt on the question of whether any investigation, however thorough, can provide answers that will scientifically resolve or reconcile the differences.

[84] The report of the EAC created by the Federal Minister of Health is instructive. The Committee described its role in the following terms:

> …The task of the EAC was to solicit evidence-based scientific and ethical data to ensure that the information needs of the Health Minister's Office were met. The primary role of the EAC was to help guide the development and implementation of the [Safe Injection Site] research plan and then to distil and synthesize the key research findings into a final report for the Minister. The EAC had no decision-making authority and were [*sic*] not responsible for making formal recommendations.

[85] The EAC reported consensus among its members on a number of issues: over 8,000 people have visited Insite to inject; 18% of that total account for 86% of overall visits; the median number of visits was 8; Insite operated near capacity; approximately 80% of daily visits are for injecting and 20% for support services; Insite injections account for less than 5% of total injections in the DTES; Insite provides a clean, supervised environment for injection where the sharing of drugs and needles is not permitted; Insite provides nursing services; users rated the services as highly satisfactory and the staff as helpful, trustworthy and respectful; letters of support and surveys show that health professionals, local police, the local community and the general public have positive or neutral views on Insite and the majority wish to see the service continue; Insite encourages users to seek counselling, detoxification and treatment; the presence of Insite facilitated the immunization of injection drug users in the DTES during an outbreak of pneumococcal pneumonia in 2006; Insite had successfully intervened in over 336 overdose events since 2006 and no overdose deaths had occurred at the site; self-reports from Insite users indicate that needle sharing decreases with increased use of the site but mathematical modelling made it difficult to accurately estimate the

number of HIV cases that might have been prevented; observations in the period before and shortly after the opening of Insite indicated a reduction in the number of people injecting in public; there was no evidence of increases in drug-related loitering, drug dealing or petty crime in areas around Insite; the Chinese Business Association reported reductions in crime in the Chinese business district outside the DTES and police data showed no changes in rates of crime recorded by police for the DTES; there was no evidence that Insite increased the relapse rate among injection drug users; and the cost/benefit analysis was favourable.

[86] The EAC's criticisms of the research, which provided the basis for the consensus, were benign. They were stated in the following terms:

> Caution should be exercised in using mathematical modelling for assessing [*sic*] cost benefit/effectiveness [*sic*] of INSITE given that:
>
> - There was limited local data available regarding baseline frequency of injection, frequency of needle sharing and other key variables used in the analysis;
> - While some longitudinal studies have been conducted, the results have yet to be published and may never be published given the overlapping design of the cohorts;
> - No studies have compared INSITE with other methods that might be used to increase referrals to detoxification and treatment services, such as outreach, enhanced needle exchange service, or drug treatment courts
>
> Some user characteristics relevant to understanding their needs and monitoring change have not been reported including details of baseline treatment histories, frequency of injection and frequency of needle sharing.
>
> User characteristics and reported changes in injection practices are based on self-reports and have not been validated in other ways. More objective evidence of sustained changes in risk behaviours and a comparison or control group study would be needed to confidently state that INSITE and [safe injection sites] have a significant impact on needle sharing and other risk behaviours outside of the site where the vast majority of drug injections still take place.

> There are a number of issues where future research could inform policy decisions, including research on the social determinants of injection drug users.

[87] Whatever the shortcomings in the science surrounding the assessment of outcomes at Insite, and however the disputes may be resolved among those who engage in the assessment of the efficacy of safe injection sites generally, or Insite in particular, all of the evidence adduced by PHS, VANDU and Canada supports some incontrovertible conclusions:

1. Addiction is an illness. One aspect of the illness is the continuing need or craving to consume the substance to which the addiction relates.
2. Controlled substances such as heroin and cocaine that are introduced into the bloodstream by injection do not cause Hepatitis C or HIV/AIDS. Rather, the use of unsanitary equipment, techniques, and procedures for injection permits the transmission of those infections, illnesses or diseases from one individual to another; and
3. The risk of morbidity and mortality associated with addiction and injection is ameliorated by injection in the presence of qualified health professionals.

[88] What is less certain and more controversial are the root causes of addiction. The evidence adduced in these proceeding regarding the character of the DTES, many of its inhabitants, and the nature of addiction leads me to the following assessment.

[89] Residents of the DTES who are addicted to heroin, cocaine, and other controlled substances are not engaged in recreation. Their addiction is an illness frequently, if not invariably, accompanied by serious infections and the real risk of overdose that compromise their physical health and the health of other members of the public. I do not assign or apportion blame, but I conclude that their situation

results from a complicated combination of personal, governmental and legal factors: a mixture of genetic, psychological, sociological and familial problems; the inability, despite serious and prolonged efforts, of municipal, provincial and federal governments, as well as numerous non-profit organizations, to provide meaningful and effective support and solutions; and the failure of the criminal law to prevent the trafficking of controlled substances in the DTES as evidenced by the continuing prevalence of addiction in the area.

IV. Analysis

A. The VANDU Claim for Declarations

[90] VANDU seeks a declaration that the conduct of the staff in the ordinary course of business at Insite does not amount to or involve the commission of any offences at law, with the result that an exemption under s. 56 of the *CDSA* is not required or necessary. The argument proceeds on the premise that the activities of the staff at Insite do not constitute possession or trafficking for purposes of the *CDSA*.

[91] Section 4(1) of the *CDSA* provides as follows:

> 4(1) Except as authorized under the regulations, no person shall possess a substance included in Schedule I, II or III.

[92] For purposes of the *CDSA*, “possession” has the meaning assigned by s. 4(3) of the *Criminal Code*:

> 4(3) For the purposes of this Act,

(a) a person has anything in possession when he has it in his personal possession or knowingly

(i) has it in the actual possession or custody of another person, or

(ii) has it in any place, whether or not that place belongs to or is occupied by him, for the use or benefit of himself or of another person; and

(b) where one of two or more persons, with the knowledge and consent of the rest, has anything in his custody or possession, it shall be deemed to be in the custody and possession of each and all of them.

[93] Sections 5(1) and (2) of the *CDSA* provide:

5(1) No person shall traffic in a substance included in Schedule I, II, III or IV or in any substance represented or held out by that person to be such a substance.

(2) No person shall, for the purpose of trafficking, possess a substance included in Schedule I, II, III or IV.

[94] The word "traffic" is defined by s. 2(1) of the *CDSA*:

""traffic" means, in respect of a substance included in any of Schedules I to IV,

(a) to sell, administer, give, transfer, transport, send or deliver the substance,

(b) to sell an authorization to obtain the substance, or

(c) to offer to do anything mentioned in paragraph (a) or (b).

[95] The law pertaining to the possession and trafficking of controlled substances has been developed in the decided cases and is well established. If the provisions of s. 4(1) and s. 5(1) apply to the staff at Insite, the answer to the question of whether, in the course of what they do, they either possess or traffic in a controlled substance at any particular point in time must be answered by reference to the facts

as they are determined in relation to any specific action or conduct and the law as it has evolved. That being the case, declaratory relief cannot be considered.

[96] The Court of Appeal discussed the court's capacity to grant declaratory relief in *Lee v. Li*, 2002 BCCA 209, 113 A.C.W.S. (3d) 192 at para. 19:

> [19] An action for a declaration must be in relation to a right and must have some utility: see *Canada v. Solosky*, [1980] 1 S.C.R. 821, where at 832 Dickson J., for the majority, quotes with approval from a learned author on the relevant factors:
>
> > As Hudson suggests in his article, "Declaratory Judgments in Theoretical Cases: The Reality of the Dispute" (1977), 3 Dal.L.J. 706:
> >
> > > The declaratory action is discretionary and the two factors which will influence the court in the exercise of its discretion are the utility of the remedy, if granted, and whether, if it is granted, it will settle the questions at issue between the parties.

[97] Other statements by the Supreme Court of Canada in *Solosky v. The Queen*, [1980] 1 S.C.R. 821 at 830-832, 105 D.L.R. (3d) 745 are relevant:

> Declaratory relief is a remedy neither constrained by form nor bounded by substantive content, which avails persons sharing the legal relationship, in respect of which a 'real issue' concerning the relative interest of each has been raised and falls to be determined: …
>
> The question must be a real and not a theoretical question, the person raising it must have a real interest to raise it, he must be able to secure a proper contradictor, that is to say, someone presently existing who has a true interest to oppose the declaration sought: *Russian Commercial and Industrial Bank v. British Bank for Foreign Trade Ltd.*, [1921] 2 A.C. 438 at 448. …
>
> ...[I]f a substantial question exists which one person has a real interest to raise, and the other to oppose, then the court has a discretion to resolve it by a declaration, which it will exercise if there is good reason for so doing: *Pyx Granite Co. Ltd. v. Ministry of Housing and Local Government*, [1958] 1 Q.B. 554 (rev'd [1960] A.C. 260, on other grounds). …
>
> …[C]ourts will not grant declarations regarding the future.

[98] In present circumstances, where the legal principles that apply to possession and trafficking are settled, where the question of whether one possesses or is trafficking in controlled substances is fact-dependent, and where the ordinary course of business is not fixed with precision and may change in the future, the declaration is sought in respect of future events and would serve no useful purpose. Although the staff at Insite have a real interest in knowing whether what they do in any particular circumstances constitutes a criminal offence, judicial discretion cannot be used to answer the question one way or the other by way of a declaration "in the air" which would have no utility.

[99] The VANDU application for the declaration in relation to staff conduct is dismissed.

B. The PHS Claim Regarding Interjurisdictional Immunity

[100] PHS and VANDU, supported by the British Columbia Civil Liberties Association as an intervenor, claim that ss. 4(1) and 5(1) of the *CDSA* do not apply to users or staff at Insite because of the doctrine of interjurisdictional immunity. The claim is framed as follows:

> (a) By design and in effect Insite is a health care undertaking within the core of provincial constitutional jurisdiction over health care pursuant to s. 92(16) of the *Constitution Act, 1867*, or some combination of ss. 92(7), (13) and (16).
>
> (b) While ss. 4(1) and 5(1) of the *CDSA* are valid legislation under the federal power to enact criminal law pursuant to s. 91(27) of the *Constitution Act, 1867*, the application of those provisions to users and staff at Insite materially intrudes on a vital part of a validly constituted provincial undertaking, or the core of the Province's jurisdiction over health care.

(c) The doctrine of interjurisdictional immunity applies to protect Insite from this intrusion of the criminal law on provincial jurisdiction and renders ss. 4(1) and 5(1) of the *CDSA* constitutionally inapplicable to users and staff who, respectively, receive and deliver health care services.

(d) Sections 4(1) and 5(1) of the *CDSA* should be "read down" so that they do not apply to staff and users at Insite.

[101] Canada responds, saying:

1. The fact the Province is competent to legislate in respect of matters of health and health care does not preclude the application of federal law to such activities.

2. There is nothing inherently constitutionally offensive with an overlap between the federal criminal law power in the area of health, and provincial authority with respect to health care.

3. The double aspect doctrine and when necessary, the doctrine of federal paramountcy, permit the joint regulation of conduct throughout Canada.

(i) The Constitutional Framework

[102] In order to place the PHS claim in context, a brief and greatly over-simplified summary of the legal principles applying to constitutional questions of this kind is required.

[103] Section 91 of the *Constitution Act, 1867*, assigns to the federal Parliament the power to make laws for the peace, order and good government of Canada in relation to all matters not coming within the classes of subjects exclusively assigned by s. 92 to the legislatures of the Provinces. In addition to assigning this general residuary power, s. 91 enumerates certain classes of subjects over which Parliament has exclusive legislative authority. One such exclusive federal power is criminal law: s. 91(27).

[104] Included in the powers assigned exclusively to the provincial legislatures under s. 92 are the following:

> (7) The establishment, maintenance and management of hospitals, asylums, charities and eleemosynary [charitable] institutions in and for the Province, other than marine hospitals.
>
> (13) Property and civil rights of the Province.
>
> (16) Generally all matters of a merely local or private nature in the Province.

[105] The manner in which the division of powers should be construed and managed in a federal context has evolved through judgments of the Supreme Court of Canada since Confederation. The topic has been the subject of extensive academic commentary and debate. The governing principles were recently discussed by the Supreme Court of Canada in *Canadian Western Bank v. Alberta*, [2007] 2 S.C.R. 3, 2007 SCC 22, and *(British Columbia) Attorney General v. Lafarge Canada Inc.*, [2007] 2 S.C.R. 86, 2007 SCC 23, which were released concurrently.

[106] The Court discussed the concept of federalism and the balance between provincial and federal legislative power in *Canadian Western Bank* at paras. 22 to 24:

> 22 …[F]ederalism was the legal response of the framers of the Constitution to the political and cultural realities that existed at Confederation. It thus represented a legal recognition of the diversity of the original members. The division of powers, one of the basic components of federalism, was designed to uphold this diversity within a single nation. Broad powers were conferred on provincial legislatures, while at the same time Canada's unity was ensured by reserving to Parliament powers better exercised in relation to the country as a whole. Each head of power was assigned to the level of government best placed to exercise the power. The fundamental objectives of federalism were, and still are, to reconcile unity with diversity, promote democratic participation by reserving meaningful powers to the local

> or regional level and to foster co-operation among governments and legislatures for the common good.
>
> 23 To attain these objectives, a certain degree of predictability with regard to the division of powers between Parliament and the provincial legislatures is essential. For this reason, the powers of each of these levels of government were enumerated in ss. 91 and 92 of the *Constitution Act, 1867* or provided for elsewhere in that Act. As is true of any other part of our Constitution -- this "living tree" as it is described in the famous image from *Edwards v. Attorney-General for Canada*, [1930] A.C. 124 (P.C.), at p. 136 -- the interpretation of these powers and of how they interrelate must evolve and must be tailored to the changing political and cultural realities of Canadian society. It is also important to note that the fundamental principles of our constitutional order, which include federalism, continue to guide the definition and application of the powers as well as their interplay. Thus, the very functioning of Canada's federal system must continually be reassessed in light of the fundamental values it was designed to serve.
>
> 24 As the final arbiters of the division of powers, the courts have developed certain constitutional doctrines, which, like the interpretations of the powers to which they apply, are based on the guiding principles of our constitutional order. The constitutional doctrines permit an appropriate balance to be struck in the recognition and management of the inevitable overlaps in rules made at the two levels of legislative power, while recognizing the need to preserve sufficient predictability in the operation of the division of powers. The doctrines must also be designed to reconcile the legitimate diversity of regional experimentation with the need for national unity. Finally, they must include a recognition that the task of maintaining the balance of powers in practice falls primarily to governments, and constitutional doctrine must facilitate, not undermine what this Court has called "co-operative federalism" (*Husky Oil Operations Ltd. v. Minister of National Revenue*, [1995] 3 S.C.R. 453, at para. 162; *Reference re Employment Insurance Act (Can.), ss. 22 and 23*, [2005] 2 S.C.R. 669, 2005 SCC 56, at para. 10). …

[107] The resolution of disputes regarding the constitutionality of impugned legislation begins with the identification of the "pith and substance" of the legislation. Pith and substance is determined by reference to the purpose for which the legislative body acts and the legal effect of the law: *Canadian Western Bank*, para. 27.

[108] The need to identify pith and substance recognizes that legislation may touch upon matters that fall outside the exclusive legislative authority of the enacting body.

In such circumstances, the court must be determined to identify the "dominant purpose" of the legislation: *Canadian Western Bank* at para.28:

> ...[T]he "dominant purpose" of the legislation is still decisive. Its secondary objectives and effects have no impact on its constitutionality: "merely incidental effects will not disturb the constitutionality of an otherwise *intra vires* law" (*Global Securities Corp. v. British Columbia (Securities Commission)*, [2000] 1 S.C.R. 494, 2000 SCC 21, at para. 23). By "incidental" is meant effects that may be of significant practical importance but are collateral and secondary to the mandate of the enacting legislature: see *British Columbia v. Imperial Tobacco Canada Ltd.*, [2005] 2 S.C.R. 473, 2005 SCC 49, at para. 28. Such incidental intrusions into matters subject to the other level of government's authority are proper and to be expected: *General Motors of Canada Ltd. v. City National Leasing*, [1989] 1 S.C.R. 641, at p. 670.

[109] In some instances it is difficult to determine whether the subject matter of the legislation pertains to a topic assigned exclusively to the federal Parliament or provincial legislatures because the topic shares aspects of both. This fact has given rise to what is known as the "double aspect doctrine" described in *Canadian Western Bank* at para. 30:

> [30] The double aspect doctrine, as it is known, which applies in the course of a pith and substance analysis, ensures that the policies of the elected legislators of both levels of government are respected. A classic example is that of dangerous driving: Parliament may make laws in relation to the "public order" aspect, and provincial legislatures in relation to its "Property and Civil Rights in the Province" aspect (*O'Grady v. Sparling*, [1960] S.C.R. 804). The double aspect doctrine recognizes that both Parliament and the provincial legislatures can adopt valid legislation on a single subject depending on the perspective from which the legislation is considered, that is, depending on the various "aspects" of the "matter" in question.

[110] To avoid inappropriate encroachment by one legislative body upon the jurisdiction of another, various techniques including "reading down", "interjurisdictional immunity" and "paramountcy" come into play. The principles are discussed in *Canadian Western Bank* at paras. 31-32:

> 31 When problems resulting from incidental effects arise, it may often be possible to resolve them by a firm application of the pith and substance analysis. The scale of the alleged incidental effects may indeed put a law in a different light so as to place it in another constitutional head of power. The usual interpretation techniques of constitutional interpretation, such as reading down, may then play a useful role in determining on a case-by-case basis what falls exclusively to a given level of government. In this manner, the courts incrementally define the scope of the relevant heads of power. The flexible nature of the pith and substance analysis makes it perfectly suited to the modern views of federalism in our constitutional jurisprudence.
>
> 32 That being said, it must also be acknowledged that, in certain circumstances, the powers of one level of government must be protected against intrusions, even incidental ones, by the other level. For this purpose, the courts have developed two doctrines. The first, the doctrine of interjurisdictional immunity, recognizes that our Constitution is based on an allocation of exclusive powers to both levels of government, not concurrent powers, although these powers are bound to interact in the realities of the life of our Constitution. The second, the doctrine of federal paramountcy, recognizes that where laws of the federal and provincial levels come into conflict, there must be a rule to resolve the impasse. Under our system, the federal law prevails. …

[111] The origin of the doctrine of interjurisdictional immunity, its nature, and its extent were described in *Canadian Western Bank* at para. 33:

> 33 Interjurisdictional immunity is a doctrine of limited application, but its existence is supported both textually and by the principles of federalism. The leading modern formulation of the doctrine of interjurisdictional immunity is found in the judgment of this Court in *Bell Canada (1988)* where Beetz J. wrote that "classes of subject" in ss. 91 and 92 must be assured a "basic, minimum and unassailable content" immune from the application of legislation enacted by the other level of government.

(ii) The Constitutional Analysis

[112] In the context of this constitutional framework, PHS concedes that ss. 4(1) and 5(1) of the *CDSA* are concerned with suppressing the availability of drugs that have harmful effects on human health, and that the provisions have been lawfully enacted by Parliament as, in pith and substance, they represent a use of its criminal law power.

[113] The question then is whether the purpose and object of Insite are immune from the reach of criminal law because of interjurisdictional immunity, or whether, because the provincial policy conflicts with a federal power, the federal law will prevail because of the doctrine of paramountcy.

[114] PHS argues that Insite is a carefully considered and developed health care facility initiated by the Health Authority, in its capacity as a delegate of the provincial legislature, and in furtherance of the provincial power to govern matters of health in the Province. It says that Insite is the product of a detailed assessment of the personal and public health consequences of injection drug use in the DTES, and the best health care delivery technique to address those issues. As such, it is part of the core area of health care over which the Province has exclusive jurisdiction and which the federal Parliament cannot impair.

[115] Counsel on behalf of PHS frames its claim in this manner:

> …[T]he provincial interest in ensuring that the Health Authority can fulfill its fundamental mandate of providing effective and responsive health care to local populations is part of the "basic minimum and unassailable content" of the Province's legislative power over the delivery of health care services. This "core" competence must be preserved to make the power effective for the purpose for which it was conferred.

PHS says that permitting the *CDSA* to override the Province’s concern and responsibility for health care within a very local community by turning injection drug users away from the health care door intrudes upon the Province’s core responsibility and the incursion must be prevented by invoking the doctrine of interjurisdictional immunity.

[116] In response, Canada says that Parliament has a compelling state interest in prohibiting the injection of controlled substances, in part because of their adverse effects on individual and community health. Canada says that the reach of the *CDSA*, which has the effect of criminalizing injection, has but an incidental effect on the provincial domain of health care. Permitting Insite to continue its operations will create a safe haven from the criminal law and undermine its national objective and importance.

[117] The difficulty in this case results from the fact that the *CDSA* prohibition against possession indirectly controls injection, which is not proscribed by the criminal law, and in doing so, has an incidental effect upon a vital part of a provincial health care undertaking. As a result, the federal power to legislate in relation to criminal law, and the power of a provincial delegate to provide health care services meet head-to-head in conflict. This is a classic case of "double aspect". That being the case, the doctrine of interjurisdictional immunity cannot be applied.

[118] The Supreme Court of Canada has said that the doctrine should be used sparingly, and not used where the subject matter presents a double aspect: *British Columbia (Attorney General) v. Lafarge Canada Inc.*, [2007] 2 S.C.R. 86, 2007 SCC 23 at para. 4. While the Court has also said that the doctrine is reciprocal in that it can prevent legislation by the provincial government in relation to the essential and vital elements of a federal power, and vice versa, it has been most often, if not always, applied to ensure that provincial legislation does not encroach upon the core of a federal power or undertaking. As the Court stated in *Canadian Western Bank* at para.78, "in practice, the absence of prior case law favouring the application [of

interjurisdictional immunity] to the subject matter at hand will generally justify a court proceeding directly to the consideration of federal paramountcy."

[119] When confronted with a double aspect, the court must strive to give legitimacy to both legislative initiatives: *Canadian Western Bank* at para. 37. In this case, however, the operation of the provincial undertaking, which is concerned with health care, interferes with or directly confronts the operation of the criminal law by permitting the possession of controlled substances at Insite contrary to the *CDSA*, which prohibits possession in all circumstances. While Parliament has some capacity to affect the supply and delivery of health care, the Province has no capacity to override the criminal law by creating an environment in which individuals can conduct themselves free of its constraints.

[120] Because there is operational conflict between the Province's initiatives in health care and the criminal law which is directed in part to health, the conflict must be resolved by application of the doctrine of paramountcy. Absent *Charter* considerations, the criminal law must prevail.

[121] The PHS and VANDU applications for declarations that ss. 4(1) and 5(1) of the *CDSA* do not apply to Insite on the basis of interjurisdictional immunity are dismissed.

C. The Charter: Section 7

[122] The question to be addressed and answered may be stated as follows:

> Does the criminalization of the possession of controlled substances within the premises of the Vancouver Safe Injection Site violate s. 7 of the *Charter of Rights and Freedoms*?

[123] Section 7 provides as follows:

> 7. Everyone has the right to life, liberty and security of the person and the right not to be deprived thereof except in accordance with the principles of fundamental justice.

(i) The Competing Claims and the Legal Context

[124] The plaintiffs claim that because s. 4(1) of the *CDSA* imposes an absolute and unqualified prohibition on the possession of controlled substances, it prevents access to Insite, a health care facility that reduces or eliminates the risk of death from an overdose and the risk of infectious disease, by persons who are ill with an addiction, thereby violating the right to life and security of the person. They also say that because users of Insite risk incarceration while seeking health care services, the right to liberty is violated. They say that the violations do not accord with the principles of fundamental justice.

[125] Canada says that the federal Parliament is empowered to prohibit the possession of controlled substances without regard for the circumstances because of their dangerous nature and the state's compelling interest in controlling their use, an interest share by the world and formalized in international treaties:

> Simply put, there is no constitutional right to the non-medical injection of hard drugs. The CDSA does not in any event provide the plaintiffs with a stark choice between committing a crime and obtaining medical treatment, as the plaintiffs would suggest. The CDSA does not prevent medical treatment for addiction, and the unbridled injection of illegal rugs, the activity at the SIS

> which the CDSA does prohibit, is not a form of medical treatment for drug addiction.

[126] Canada says that if s. 4(1) contravenes s. 7, then it is saved by s. 1 of the *Charter* because the absolute prohibition is a reasonable limit prescribed by law on the right to life, liberty, and security of the person as can be demonstrably justified in a free and democratic society.

[127] Two decided cases are of particular importance in the present circumstances: *R. v. Parker* (2000), 49 O.R. (3d) 481, [2000] O.J. No. 2787 (Ont. C.A.), and *R. v. Malmo-Levine; R. v. Caine*, [2003] 3 S.C.R. 571, 2003 SCC 74.

[128] In *Parker*, the Ontario Court of Appeal held that the prohibition against the possession of marijuana for use as a medical antidote to epilepsy violated s. 7 of the *Charter* and was not saved by s. 1. *Parker* was not appealed to the Supreme Court of Canada. Rather, Parliament responded by promulgating the *Marihuana Medical Access Regulations*, SOR/2001-227.

[129] In *Malmo-Levine*, the Supreme Court of Canada considered a *Charter* challenge to the general prohibition imposed by the *CDSA* against the simple possession of marijuana for recreational purposes. The Court held that s. 4(1), in the context of that purpose, did not infringe s. 7.

[130] Several principles emerge from *Malmo-Levine*:

1. The prohibition against the use of marijuana for recreational purposes is supported under the criminal law power conferred upon Canada by s. 91(27) of the *Constitution Act, 1867*: para. 72.

2. The federal criminal law power has been broadly construed:

> A crime is an act which the law, with appropriate penal sanctions forbids; but as prohibitions are not enacted in a vacuum, we can properly look for some evil or injurious or undesirable effect upon the public against which the law is directed. That effect may be in relation to social, economic or political interests; and the legislature has had in mind to suppress the evil or to safeguard the interest threatened.
>
> *Malmo-Levine*, para. 73 citing *Reference re Validity of s. 5(a) of the Dairy Industry Act,* [1949] S.C.R. 1.

3. The harm attributed to the non-medical use of marijuana is the "evil or injurious or undesirable effect" to which the *CDSA* is directed and is a lawful and proper use of the legislative competence conferred under s. 91(27) of the *Constitution Act, 1867*: paras. 73 and 78.

4. The availability of imprisonment for the offence of simple possession is sufficient to trigger s. 7 scrutiny: para. 84.

5. Liberty is afforded individuals in respect of matters that "can properly be characterized as fundamentally or inherently personal such that, by their very nature, they implicate basic choices going to the core of what it means to enjoy individual dignity and independence": para. 85, citing *Godbout v. Longueuil (City)*, [1997] 3 S.C.R. 844 at para. 66.

6. The constitution cannot be stretched to afford protection to whatever activity an individual chooses to define as central to his or her lifestyle: para. 86.

7. The desire to build the lifestyle around the recreational use of marijuana does not attract *Charter* protection. There is no free-standing constitutional right to smoke "pot" for recreational purposes: para. 87.

8. The principles of fundamental justice embraced by s. 7 of the *Charter* include the following:

    (a) a criminal law that is arbitrary or irrational will infringe s. 7: para. 135, citing *R. v. Arkell*, [1990] 2 S.C.R. 695, at 704; and *R. v. Hamon* (1993), 85 C.C.C. (3d) 490 (Que. C. A.) at 492.

    (b) A criminal law that is grossly disproportionate, the proof of which rests with the claimant, will infringe s. 7: para. 143.

9. Disproportionality of penalty, adverse personal consequences such as a criminal record, or the ineffectiveness of the law do not contribute to a finding of gross disproportionality.

10. The balance of salutary and deleterious effects of the law is a factor to be considered in the context of s. 1 of the *Charter*, not s. 7.

[131] Canada says the PHS and VANDU actions should be decided by reference to the principles enunciated in *Malmo-Levine*:

> The [drugs used at Insite] are all more harmful than marijuana. Unlike marijuana, there have been many deaths reported as a result of overdose of opioids, central stimulants, and speedball. If, as the Supreme Court of Canada has already held, the physical and social harms of marijuana represent a compelling state interest to criminalize the possession of marijuana for non-medical use, *a fortiori* there is a compelling state interest to prohibit the use of the deadly and socially corrosive [Insite] drugs.

[132] Canada acknowledges that the Supreme Court left open the question of how the medical use of marijuana might be analyzed, but says that is not an issue here because permitting the injection of dangerous drugs with impunity is not medical treatment for drug addition.

[133] *Parker* was concerned with the impact of the criminal law upon an individual claiming a need for marijuana as a matter of medical necessity, not recreational use. The Crown appealed a trial judge's ruling that the prohibition against the cultivation and possession of marijuana by Parker, who required the product in order to control his epilepsy, infringed his s. 7 *Charter* right. The Ontario Court of Appeal upheld the trial decision, saying that forcing Parker to choose between his health and imprisonment violated his right to liberty and security of the person, and the violations did not accord with the principles of fundamental justice. Although *Parker* preceded the hearing and disposition of *Malmo-Levine*, it was not the subject of consideration or adverse comment by the Supreme Court.

[134] Several principles emerge from *Parker*:

1. The importance of the right or freedom in issue must be assessed in context rather in the abstract, and its purpose must be ascertained in context: paras. 82, 83:

> 83. The dominant aspect of the context in this case is the claim by Parker and other patients that they require access to marijuana for medical reasons. They do not...assert a desire for marijuana for recreational use. Parker does not claim a right to use marijuana on the basis of some kind of abstract notion of personal autonomy. The validity of the marijuana prohibition must be assessed in that particular context. The context here is not simply that the marijuana prohibition exposes Parker, like all other users and growers, to criminal prosecution and possible loss of liberty. Rather, Parker alleges that the prohibition interferes with his health and therefore his security interest as well as his liberty interest.

2. A liberty interest is engaged by the threat of criminal prosecution and possible imprisonment, as well as by the right to make decisions of fundamental personal importance:

3. Deprivation by means of a criminal sanction of access to medication reasonably required for the treatment of a medical condition that threatens life or health constitutes a deprivation of security of the person, as does preventing access to a treatment by threat of criminal sanction: para.97.

4. At the point where the criminal law and medical treatment intersect, certain principles of fundamental justice apply: para. 117:

> To summarize, a brief review of the case law where the criminal law intersects with medical treatment discloses at least these principles of fundamental justice:
>
> (i) The principles of fundamental justice are breached where the deprivation of the right in question does little or nothing to enhance the state's interest.
>
> (ii) A blanket prohibition will be considered arbitrary or unfair and thus in breach of the principles of fundamental justice if it is unrelated to the state's interest in enacting the prohibition, and if it lacks a foundation in the legal tradition and societal beliefs that are said to be represented by the prohibition.

> (iii) The absence of a clear legal standard may contribute to a violation of fundamental justice.
>
> (iv) If a statutory defence contains so many potential barriers to its own operation that the defence it creates will in many circumstances be practically unavailable to persons who would *prima facie* qualify for the defence, it will be found to violate the principles of fundamental justice.
>
> (v) An administrative structure made up of unnecessary rules, which result in an additional risk to the health of the person, is manifestly unfair and does not conform to the principles of fundamental justice.

[135] The circumstances that prevail in the case of Insite and its users are neither those in *Malmo-Levine* nor *Parker*. Parker suffered from an illness while Malmo-Levine did not. Malmo-Levine used marijuana for recreation and pleasure while Parker used marijuana as an antidote to epilepsy. Although those who inject at Insite are not using controlled substances as an antidote for an illness, they suffer from an illness and the need to obtain the substance by injection is a material part of the illness, as is apparent from the evidence of both Dr. Marsh and Dr. Evans.

[136] While users do not use Insite to directly treat their addiction, they receive services and assistance at Insite which reduce the risk of overdose that is a feature of their illness, they avoid the risk of being infected or of infecting others by injection, and they gain access to counselling and consultation that may lead to abstinence and rehabilitation. All of this is health care.

[137] In my opinion, the *Malmo-Levine* decision, concerned with the use of marijuana for purely recreational purposes, does not resolve the issues raised by the

PHS and VANDU actions, concerned as they are with the health care of addicts resorting to a continuum of services. The actions raise and require an answer to the question of whether s. 4(1) of the *CDSA* violates s. 7 of the *Charter* by arbitrarily infringing the right to life, liberty and security of the person otherwise than in accordance with the principles of fundamental justice. If it does, then so too must s. 5(1), for reasons I will discuss.

(ii) Section 4(1) of the CDSA and s. 7 of the Charter

[138] The right to life and security of the person, and to a lesser degree the right to liberty, are engaged by s. 4(1) of the *CDSA* in relation to the activities at Insite.

[139] A law that infringes life, security of the person or liberty is unconstitutional unless it accords with the principles of fundamental justice. A law that is arbitrary, overbroad or grossly disproportionate in its effect contravenes those principles. It need not be all of those things. A law that is either arbitrary, overbroad or disproportionate cannot withstand *Charter* scrutiny.

(a) Risk to Life

[140] Section 4(1) of the *CDSA*, which prohibits injection within the confines of Insite, engages the right to life because it prevents healthier and safer injection where the risk of mortality resulting from overdose can be managed, and forces the user who is ill from addiction to resort to unhealthy and unsafe injection in an environment where there is a significant and measurable risk of morbidity or death.

The risk of death as a consequence of the use of narcotics is well-chronicled: see the report of the Coroner, *supra,* para. 20.

[141] Not every threat to life commends itself to *Charter* scrutiny. The threat must flow from the actions of the state. As I appreciate its argument, Canada says that the threat to life results from an individual's choice to inject a harmful and dangerous narcotic rather than state action.

[142] With respect, the subject with which these actions are concerned has moved beyond the question of choice to consume in the first instance. As I have said elsewhere in these reasons, the original personal decision to inject narcotics arose from a variety of circumstances, some of which commend themselves to choice, while others do not. However unfortunate, damaging, inexplicable and personal the original choice may have been, the result is an illness called addiction. The failure to manage the addiction in all of its aspects may lead to death, whether from overdose or other illness resulting from unsafe injection practices. If the root cause of death derives from the illness of addiction, then a law that prevents access to health care services that can prevent death clearly engages the right to life.

(b) Risk to Liberty

[143] An individual's liberty interest is engaged because the *CDSA* comprehends the possibility of prosecution and incarceration for the possession of controlled substances: *Malmo-Levine*, para. 84, and *Parker*, para. 101. However, in the present context, the threat to liberty is of considerably less importance than the threat to life and security of the person.

(c) Risk to Security of the Person

[144] Section 4(1) of the *CDSA* threatens security of the person. It denies the addict access to a health care facility where the risk of morbidity associated with infectious disease is diminished, if not eliminated. While it is popular to say that addiction is the result of choice and the pursuit of a liberty interest that should not be afforded *Charter* protection, an understanding of the nature and circumstances which result in addiction, as I have discussed elsewhere in these reasons, must lead to the opposite conclusion. Society cannot condone addiction, but in the face of its presence it cannot fail to manage it, hopefully with ultimate success reflected in the cure of the addicted individual and abstinence.

[145] Canada argues that the right to security of the person is not engaged because those who use Insite do not do so for the purpose of treating an illness, but merely to satisfy the craving for an illegal drug.

[146] Denial of access to Insite and safe injection for the reason stated by Canada, amounts to a condemnation of the consumption that led to addiction in the first place, while ignoring the resulting illness. While there is nothing to be said in favour of the injection of controlled substances that leads to addiction, there is much to be said against denying addicts health care services that will ameliorate the effects of their condition. Society does that for other substances such as alcohol and tobacco. While those are not prohibited substances, society neither condemns the individual who chose to drink or smoke to excess, nor deprives that individual of a range of health care services. Management of the harm in those cases is accepted

as a community responsibility. I cannot see any rational or logical reason why the approach should be different when dealing with the addiction to narcotics, an aspect of which is that the substance that resulted in the addiction in the first place will invariably be ingested in the short-term, and possibly in the long-term, because of the very nature of the illness. Simply stated, I cannot agree with the Canada's submission that an addict must feed his addiction in an unsafe environment when a safe environment that may lead to rehabilitation is the alternative.

[147] Because life, security of the person and to a much lesser degree liberty are engaged by the operation of s. 4(1) of the *CDSA*, I must consider whether the prohibition against possession as it applies at Insite accords with the principles of fundamental justice.

(iii) Principles of Fundamental Justice

[148] Canada says that the compelling state objective of prohibiting the use of hard drugs which are dangerous to users and to society at large, the linkage of the drug trade to organized crime, and the opposition of the international community to narcotics as evidenced by treaties, mean that s. 4(1) is rationally connected to a reasonable apprehension of harm, not arbitrary, and therefore not offensive to the principles of fundamental justice.

[149] Canada's claims are not immune to challenge. International treaties cannot undermine or override domestic constitutional law and Parliament's obligation to ensure that its laws comply with the *Charter*: *Parker* at para.147. Some vigorously argue that the link between drugs and organized crime arises from the profit

associated with illegal trafficking. Canada postulates that by eliminating consumption, trafficking is rendered less viable. Others argue with equal fervour that traffickers have a product to sell, and as with many who possess products, whatever their nature, they will find a market or create one if none exists.

[150] Even if one accepts Canada's assertion regarding compelling state objectives, the resulting law cannot be arbitrary.

[151] McLachlin C.J. addressed the question of arbitrary laws in *Chaoulli v. Quebec (Attorney General)*, [2005] 1 S.C.R. 791, 2006 SCC 35 at para. 129-130:

> It is a well-recognized principle of fundamental justice that laws should not be arbitrary: see, eg, *Malmo-Levine*, at para.135.
>
> A law is arbitrary where "it bears no relation to, or is inconsistent with, the objective that lies behind [it]". To determine whether this is the case, it is necessary to consider the state interest and societal concerns that the provision is meant to reflect. …

[152] In my opinion, s. 4(1) of the *CDSA*, which applies to possession for every purpose without discrimination or differentiation in its effect, is arbitrary. In particular it prohibits the management of addiction and its associated risks at Insite. It treats all consumption of controlled substances, whether addictive or not, and whether by an addict or not, in the same manner. Instead of being rationally connected to a reasonable apprehension of harm, the blanket prohibition contributes to the very harm it seeks to prevent. It is inconsistent with the state's interest in fostering individual and community health, and preventing death and disease. That is enough to compel the conclusion that s. 4(1), as it applies to Insite, is arbitrary and not in

accord with the principles of fundamental justice. If not arbitrary, then by the same analysis, s. 4(1) is grossly disproportionate or overbroad in its application.

[153] The conclusion I have reached in relation to s. 4(1) applies equally to s. 5(1) of the *CDSA*. It is possible that staff at Insite who handle used equipment contaminated by controlled substances, or staff who take possession of any controlled substance for delivery to police, could be alleged to be engaged in "trafficking", which is broadly defined by the *CDSA* to the administration or transfer of a controlled substance. Failure to protect the staff against such an allegation would negative the utility of any determination that s. 4(1) is contrary to s. 7.

(iv) Section 56 of the CDSA

[154] The Crown claims that s. 56 of the *CDSA* provides a mechanism to offset any arbitrary aspect that one may attribute to s. 4(1). In this regard the Ontario Court of Appeal in *Parker*, para. 184-187, said:

> [184] In view of the lack of an adequate legislated standard for medical necessity and the vesting of an unfettered discretion in the Minister, the deprivation of Parker's right to security of the person does not accord with the principles of fundamental justice.
>
> [185] In effect, whether or not Parker will be deprived of his security of the person is entirely dependent upon the exercise of ministerial discretion. While this may be a sufficient legislative scheme for regulating access to marijuana for scientific purposes, it does not accord with fundamental justice where security of the person is at stake. …
>
> [187] ...The court cannot delegate to anyone, including the Minister, the avoidance of a violation of Parker's rights. Section 56 fails to answer Parker's case because it puts an unfettered discretion in the hands of the Minister to determine what is in the best interests of Parker and other persons like him and leaves it to the Minister to avoid a violation of the patient's security of the person.

[155] The unfettered nature of the discretion to exempt is apparent in this case. Following a detailed assessment of medical and social need, the Health Authority applied for an exemption that would permit Insite to operate. The heading under which the Minister granted the exemption was “necessity for a scientific purpose”. No reference was made to necessity for a medical purpose. No reference was made to necessity in the public interest, which, in the context of the DTES, was the over-riding concern.

[156] While I do not conclude that s. 56 of the *CDSA* is unconstitutional, it cannot be relied upon as an antidote to the violation of s. 7 rights that has been established in relation to the users of Insite.

V. The Charter: Section 1

[157] Canada argues that if s. 4(1) of the *CDSA* offends s. 7 of the *Charter*, it is saved by s. 1 as a law that is a reasonable restraint on s. 7 rights in a free and democratic society. In my opinion, the law compels the dismissal of the claim. The principles of fundamental justice are among the most important in society. Any law that offends them will not ordinarily be saved by s. 1: *New Brunswick (Minister of Heath and Community Services)*, [1999] 3 S.C.R. 46, 177 D.L.R. (4th) 124 at para. 99. Given what is at stake, the present case is no exception.

VI. Disposition

[158] In sum, I declare that ss. 4(1) and 5(1) of the *CDSA* are inconsistent with s. 7 of the *Charter*, and of no force and effect.

[159] I suspend the effect of the declaration of constitutional invalidity until June 30, 2009. In the interim, and in accordance with the direction of the Supreme Court of Canada in *R. v. Ferguson*, 2008 SCC 6, 228 C.C.C. (3d) 385 at para. 46, I grant users and staff at Insite, acting in conformity with the operating protocol now in effect, a constitutional exemption from the application of ss. 4(1) and 5(1) of the *CDSA*.

"I. Pitfield J."
