# Supplementary material for: Fighting addiction's death row: British Columbia Supreme Court Justice Ian Pitfield shows a measure of legal courage
Source: Harm Reduct J. 2008 Oct 28;5:31. doi: 10.1186/1477-7517-5-31 (PMC2611970; doi:10.1186/1477-7517-5-31)
Supplement: Additional file 2 — Report of the Task Force into illicit narcotic overdose deaths in British Columbia . The results of a provincial task force examining fatal overdoses due to injection drug use. [file 1477-7517-5-31-S2.pdf]

REPORT OF THE TASK FORCE INTO

Illicit Narcotic Overdose Deaths in British Columbia

Office of the Chief Coroner

Canadian Cataloguing in Publication Data
British Columbia. Task Force into Illicit Narcotic
Overdose Deaths in British Columbia.
Report of the Task Force into Illicit Narcotic
Overdose Deaths in British Columbia.

Issued by Ministry of Attorney General.
ISBN 0-7726-2312-0

1. Narcotic addicts - British Columbia.
2. Narcotic addicts - Services for - British Columbia.
3. Narcotics, Control of - British Columbia.
4. Narcotic Habit - Social aspects - British Columbia
I. British Columbia Coroners Services. II. British
Columbia. Ministry of Attorney General. III. Title.

RV5840.C32B74 1994 362.29'309711 C94-960387-2

Province of
British Columbia
Ministry of
Attorney General

OFFICE OF THE
CHIEF CORONER

Metrotower II
Suite 2035, 4720 Kingsway
Burnaby
British Columbia
V5H 4N2
Telephone: (604) 660-7745
Fax: (604) 660-7766

September 6, 1994

Honourable Colin Gabelmann
Attorney General
Parliament Buildings
Victoria, BC V8V 1X4

Dear Minister:

I am pleased to submit this Report on the Task Force into Illicit Narcotic Overdose Deaths in British Columbia.

It is the result of eight months private and public consultations, and extensive research covering the specific Terms of Reference assigned, as broad and general as they were. In that vein, and while originating with the Minister of Health of the day, the issues extend into your Ministry, Social Services, Education, Native Affairs, and Municipalities. I, therefore, enclose copies sufficient for dissemination to those offices.

In the hope this Report will be received in the spirit in which it was conceived and delivered, my added hope is that it will enure to the benefit of all residents and future generations in this Province.

Thank you.

Sincerely,

Vince Cain

J. V. Cain
Chief Coroner
Province of British Columbia

enclosures

TABLE OF CONTENTS

TERMS OF REFERENCE i

INTRODUCTION iii

EXECUTIVE SUMMARY v

Chapter 1 THE ADDICT 1

Chapter 2 THE EPIDEMIOLOGY OF OVERDOSE DEATHS IN BC 5

Chapter 3 PUBLIC HEALTH / TREATMENT 15
- Part A .... EMERGENCY SERVICES .... page 15
- Part B .... HARM REDUCTION .... page 18
- Part C .... DETOX .... page 24
- Part D .... METHADONE .... page 28
- Part E .... SUPPORTIVE RECOVERY .... page 32

Chapter 4 SOCIAL ISSUES 37
- Part A .... SOCIAL SERVICES .... page 37
- Part B .... POVERTY/UNEMPLOYMENT .... page 42
- Part C .... WOMEN AND CHILDREN .... page 43
- Part D .... FAMILY AND YOUTH .... page 51
- Part E .... FIRST NATIONS .... page 56
- Part F .... OTHER DISADVANTAGED GROUPS .... page 62

Chapter 5 THE JUSTICE SYSTEM 65
- Part A .... POLICE .... page 65
- Part B .... COURTS .... page 68
- Part C .... CORRECTIONS .... page 70

Chapter 6 COSTS 75

Chapter 7 EDUCATION 77

Chapter 8 LEGALIZATION / DECRIMINALIZATION 85

RECOMMENDATIONS 89

DEFINITIONS 95

BIBLIOGRAPHY 103

TERMS OF REFERENC[illegible]

TASK FORCE INT[illegible]
ILLICIT NARCOTIC DEATH[illegible]
IN BRITISH COLUMBI[illegible]

1. **TO INQUIRE INTO AND REPORT** on ways to enhance:

   a) community and government responses to the social, economic, and health care needs of people addicted to the use and abuse of illicit narcotic drugs;

   b) the delivery of service programs affecting particularly, but not exclusively, the disaffected, children, youth, women, First Nations people, and mentally-disabled people in British Columbia.

2. **TO REVIEW** the nature, causes, and extent of untimely and unnatural deaths resulting from the use and abuse of illicit narcotic drugs.

3. **TO REVIEW** the user profile, the market prevalence of cocaine and heroin, and the role of alcohol enhancing lethality.

4. **TO REVIEW** existing treatment and educational programs, including their availability and effectiveness in the prevention of injury and death to people affected directly or indirectly through illicit drug use.

5. **TO REVIEW** inter-agency liaison, communication, enforcement, and control, as they affect the delivery of services in ameliorating the individual and social problems associated with illicit narcotic drug abuse.

6. **TO RECOMMEND** strategies and policies for the enhancement of programs in the delivery of services, to serve as models or be modified to meet individual, community, and provincial needs.

7. **TO RECOMMEND** strategies which will lead to a reduction in the demand for, and a suppression of the supply of, illicit narcotic drugs in British Columbia.

INTRODUCTION

In late June 1993, the Minister of Health and the Attorney General announced in the Legislature the appointment of the Chief Coroner to head up a task force to inquire into what appeared to be an inordinately high number of deaths associated with the illicit use of heroin. The media were simultaneously reporting that these deaths followed the distribution of welfare cheques toward the end of each month. Hence, the headlines associating "Welfare Wednesday" with these events.

In mid July 1993, the Chief Coroner convened a meeting of key people to discuss the nature and extent of the perceived problem. These people included representatives from the health professions and social services, law enforcement officials, native workers, street workers, and recovering addicts. Written and verbal submissions were received and discussed in a communal fashion. As a result, terms of reference for this task force were drawn up and submitted to the Ministry of Attorney General on September 1, 1993. Final concurrence was received on December 21, 1993. On January 6, 1994, the task of inquiring into these matters began in earnest.

It was determined that travelling the province with an entourage of six to ten people in a task force would not be feasible under the circumstances. Consequently, the Chief Coroner undertook to do this singularly, with the assistance of a seasoned, experienced person seconded from the Ministry of Attorney General and a bright, diligent policy analyst from the Coroners Service who also acted as recording secretary. The process involved first of all travelling to key areas of the province to conduct in-camera sessions with people directly involved in, or affected by, the illicit narcotic problem. The purpose here was to obtain the broadest and most accurate assessments from the street scene, or the "real world." This was carried out in January and February 1994.

The second three-month stage entailed travelling to these same locations and holding public meetings. Concerns and issues were presented and discussed openly by members of the general public and, on occasion, by those same workers and addicts who attended the closed sessions. These meetings were held in March, April, and May 1994. The final public hearing held at Carnegie Centre in East Vancouver was originally scheduled for Thursday, May 26, but was postponed to June 9th because "Welfare Wednesday" was thought to have a direct negative impact on the May 26 meeting. Hence, this phase was completed a little behind schedule.

Many written submissions were received privately and many private meetings were held with interested people and groups. A considerable amount of research was done to arrive at some reasonable connections between theory and practice, between fiction and fact. This was done in June, July, and August 1994.

The project would not have been possible without the contributions of many individuals and agencies. It is not possible to list every single individual who helped in some way, but I remain unequivocally grateful to all who offered their opinions and expertise.

While most of the issues and concerns were echoed in each community, I am most grateful for the significant contributions of the following people and agencies: the Infants and Children at Risk Program at Sunny Hill Health Centre for Children and, in particular, Mr. Ron Lindstrom; the YWCA's Crabtree Corner; Ms. Rain Daniels and Ms. Betty MacPhee; Dr. Kendall Ho, Emergency Physician at Vancouver General Hospital; Dr. Stuart Huckin and Dr. Sheila Carlyle, Provincial Toxicology Centre; Mr. John Turvey and Ms. Judy McGuire from the Vancouver Needle Exchange (DEYAS); Mr. Lou Demerais from the Vancouver Native Health Society; Dr. Liz Whynot, from the Vancouver Health Department; and Dr. Bob Fisk and Dr. John Miller from the Ministry of Health.

I would like to express my sincere appreciation to the staff of these organizations and all others for their ongoing efforts in prevention, education, and support of individuals and families from all walks of life who are so profoundly affected by the illicit use of narcotics.

Without the extraordinary talents and efforts of Mrs. Peggy Justason, the policy analyst in the Coroners Service, and Mr. Scott Denoon, seconded from the Ministry of Attorney General, Corrections Branch, who gave so generously of their time and energies, this project simply could not have been completed. Finally, abundant thanks is due to Ms. Dawn Rumsby, our typesetter in the Coroners Office, for patiently and neatly typing this over and over again.

Just as so many bereaving family members expressed the hope that their loved one's death was "not in vain," it is my genuine wish that this report will likewise not be in vain, but rather that it will help in creating a greater understanding of the problem, as well as producing tangible results that will have a positive effect on all of us.

EXECUTIVE SUMMARY

It was clear from the first meeting in July 1993, that the issue was not about death. Rather, it was about life. Appropriately, the Coroners Service was selected to inquire as a result of these deaths, for the service's mandate is "the investigation of death in order to assist the living." The July assembly of professionals, workers, and recovering addicts spoke eloquently about respecting personal and social problems and human needs. They spoke of living, not dying.

Perhaps that is why this report is formulated in the manner in which it is. Death may be considered fairly simple to investigate in one sense. The medical cause of death, given the technology and professional expertise available to the coroner, may be neatly determined. Narcotic overdose deaths, on the other hand, are not always explained solely in terms of excessive intake of the drug. One person may die in a given set of circumstances, while another may not.

And what of the circumstances leading up to and surrounding these deaths? These are not so readily measurable. In fact, they are damned complex and confounding: human behaviour, human failings, social issues, and political issues. Various opinions were expressed, ranging from "What's the worry; they're dead, aren't they?" to "We have a social conscience which ought to drive us to assist those who cannot help themselves."

This report covers complex issues which deal with living, not dying. Certainly Chapter II, on Epidemiology, captures what statisticians and health professionals would expect to see in such a report, but Chapter I on The Addict is really what this report is all about. Unless there is a greater understanding on the part of the general public of who these people are, where they come from, and where they are going — all the elements that have gone into their present state — then I am afraid our collective social conscience will not drive us to assist them. One of the main tenets of this report is to educate the public and thereafter the many who are more directly affected.

The drug problem in British Columbia is very real and very serious. No one in this province is immune to the problem. It is costing the taxpayer an enormous amount of money. It is a social problem, as well as a health problem. The answers are not easily found. Neither are the remedies cheap. The problems cover a wide range of issues, the solutions equally expansive and expensive, but unless these are dealt with head-on and now, future generations may well be unable to contend with the consequences of our generation's unwillingness to face up to reality.

This report is not only about reducing harm to addicted people who suffer physically, mentally, and morally; it also touches on curbing harm to others who are affected by the primary carrier. I speak of the families, the women, the children, and the neighbourhood. This is why Chapter IV, covering Social Issues, flows naturally from Chapter III, on Public Health and Treatment. Treating an afflicted person in isolation simply won't work. Addicts are not alone in this world, and they shouldn't be led to believe that they are. When one examines all the social issues involved here, it boggles the mind. Where does it all begin, where will it all end? What is evident is that there are so many agencies and departments involved in all of these activities that one is led to conclude this is an industry, with many who are perceived to be living off the avails of misery.

The attack on all of this must somehow be coordinated and multi-faceted. There must be some reality of partnership in dealing with the issues. The addicts and those around them, I found, were pointed in any number of directions when seeking help. As a result,

many "fall between the cracks." No wonder they would retreat to "those who understand," where they are accepted; no wonder society has a subculture in the alcohol and drug addiction arena. Their acts are not socially acceptable. Therefore, they too are rejected.

The so-called "War on Drugs" which is conducted by the Justice System can only be regarded as an expensive failure. This whole area, covered in Chapter V, requires a very frank and objective analysis, not of the police, courts, and corrections system in isolation, but rather in partnership with the social issues, the public health concerns, and the treatment perspectives. Police officers told me they're wasting their time on this social issue. Correctional officers indicated quite clearly that jail isn't the place to solve the social problems. This is a health problem with a tremendous social overlay. "When are we going to face up to the realities of this?" many asked.

Education is a most important consideration in this global picture. Chapter VII touches on what is being done in this field, and what might also be done. While recommendations are presented in this and other chapters, I would expect many more such suggestions to come as others read through the various sentences of this report. Just as the report is far from all-inclusive, so too the recommendations are merely general pointers, hopefully in the right direction. The problems have been a long time developing; solutions must be reviewed strategically — perhaps two or three generations down the road — but come they must.

By far the most controversial part of the report will be Chapter VIII, on *Legalization and Decriminalization*. Legalization is a huge issue which, while the drug problem is largely here, does not have a unique British Columbian solution. It is bigger than Canada, indeed bigger than North America. It is international in scope and in law. Canadian laws and United Nations conventions control this matter, but that ought not detract from the reality of the situation in British Columbia. We have the problem, and we must do what we can about it, now. Consequently, I am recommending the establishment of a commission to examine and challenge those legal aspects of the problem, amongst so many other things. The problem must be looked at with regard to not only the aspect of deaths from heroin and cocaine, but rather the entire smorgasbord of available illicit narcotics, both so-called "soft" and "hard" drugs.

Simultaneously, I am recommending the decriminalization of simple possession of specific "soft" and "hard" drugs, the specificity to be left to the experts on that commission. We have a serious problem and I submit that those directly affected ought to be dealt with through a medical model, not a criminal model, even to the point where I am suggesting the possibility of providing heroin to seriously addicted people, in a para-medical model. One material reason for this would be to reduce the demand from the street trafficker, replacing it through the clinic, not unlike the current situation with methadone.

While that is one aspect of the problem, on the other side of the coin, society must continue to deal with importers and traffickers of these still illegal substances. I am recommending that, for the first offence, importers for the purpose of trafficking receive a life sentence of 25 years, without parole. Traffickers in medium to large amounts should receive a mandatory ten year sentence for the first offence, and a life sentence of 25 years, without parole, for the second offence. People who are not citizens of this country and who either import and/or traffic should be subject to the same penalties, with the option of immediate deportation. Bail should not be a serious option. These measures are indeed tough, but they are necessary if we are to develop a reputation for not being a place to import and deal drugs. At the same time, the province has an opportunity to become a place where residents who are burdened with serious difficulties will receive humanistic, professional treatment and support.

It is my considered opinion, having heard all I have heard, and read and discussed what was available, that the opinions and good sense of British Columbians should prevail in these matters. This is their story, not mine. Much of this report is anecdotal, but it is how those involved — addicts, professionals and others — perceive situations to be. Not infrequently, perception is reality. These are their concerns, their aspirations, their hopes for a better society in the province in which they live, as well as their hopes for their children. I merely present them as instructed by the ministers of the day. Controversy, yes; unanimity, no. Regardless, it is their story and I promised them I would tell it "as it is."

I therefore RECOMMEND THAT the Government of British Columbia:

1. Establish an independent body under the aegis of the Legislative Assembly of the Province, with powers of inquiry into matters relating to the use and abuse of illicit and harmful substances in the province.

   This body would be comprised of a permanently appointed chair and qualified people seconded from the Ministries of Health, Social Services, Attorney General, and Education and, where requested or directed, municipal and community representation would be established locally with direct links to that body.

   The body would be entitled the *Substance Abuse Commission.*

   The mandate and terms of the reference of the commission would be to identify and advise the respective ministers of substance abuse issues and resolutions affecting their ministries, individually, and collectively.

   Without restricting the generality of the foregoing, the commission would:

   (a) undertake research into the various treatment models, including methadone maintenance, harm reduction, and abstinence;

   (b) develop an inter-ministry, multi-agency information research capacity to gather and share data on all aspects of substance abuse;

   (c) develop standards, policies, procedures, and service models respecting the care and treatment of substance abusers in the province;

   (d) examine existing legislation governing the controls over illicit and licit substances and, where benefits are identified, recommend amendments to legislation to the Legislative Assembly of the Province.

Chapter 1

THE ADDICT

"Addicts are People of Sand. They have no connections with their roots and no connections with the past or future ... they live in the moment, in the now ... they need structure and comprehensive total assistance ... until we deal with the emotions, everything else is short-term."

I am not certain the general public in the province of British Columbia is familiar with "the big picture" of just who "the addict" is.

The media portrays the most visible, most overtly controversial, most socially, and economically depressed people as addicts. The police, concerned as they ought to be with maintaining the incidence of criminal acts to at least a tolerable level, likewise are accused of hitting on the most visible of addicts who "commit crimes to maintain their habits." These particular groups of addicts were alluded to as "easy pickins" for the police. Paramedics on the street and emergency physicians and nurses in the hospitals are similarly influenced by the types of patients who land on their doorsteps. And who do the social workers, the street level caregivers, see as the addict? What then of the general public?

Perhaps it is time to lift the mask and inquire beyond the obvious, visible scene. There are others in the province who are substance abusers. They come in all sizes and shapes: all classes, colours, sexes, professions, and socioeconomic backgrounds. One addict asked me if I had heard about a lawyer in town who had overdosed and died. He said there was no mention of it in the paper. Another incident concerned a physician; that was publicized, but with some protestations. This is called "denial". One recovering cocaine addict related how at the age of 34 he had lost his entire business — employees and all – over a three year period. His public admission, albeit accompanied by a sense of shame, was indeed a courageous and brave personal act. This is called "admission."

It is difficult to lift the veil, because those who supply the heroin and cocaine to "the invisibles" were not readily available to discuss their clients. They traffic for profit and greed and are not inclined to share information or profits. However, in some communities, recovering addicts who earlier trafficked for profit indicated I would be surprised to learn who were users in those communities. These statistics are not very scientific and perhaps not all that reliable; nevertheless, they are not necessarily implausible. Certainly, the people behind them are not visible. One emergency physician put it this way: "We don't get many pin-stripe suits in here, but they do come, on occasion." Coroners statistics do not reflect the socioeconomic backgrounds of people dying in this fashion, but ambulance attendants seem to indicate that there are indeed calls to residences in middle and upper class sectors of the community.

I point this out to stress the fact that all kinds of people, from all walks of life, can get caught up in substance abuse. Here it is important to point out that one can be addicted to a variety of drugs, including tobacco, alcohol, mind-altering narcotics, designer drugs, cocaine, opiates, and the opiate derivative heroin. I stress this to cause a lifting of the veil, an unmasking of the cover-up, so that those who are damaging themselves and others, psychologically and socially, can avail themselves of this "window of opportunity" to recognize the problematic nature of their substance abuse. This is called "admission," acknowledging that a personal situation is adversely affecting oneself, others, the community, and the nation.

The majority of addicts I spoke with fell into the most visible group. They told horrendous tales of personal tragedy, both before and during addiction. It is not actually possible to come up with a profile which would encompass every addict. Each is unique, each is different, each has his or her own personality, perhaps as nature intended it. This is important to remember when seeking solutions and developing programs for recovery and prevention. Addicts range from the infant who was born with Fetal Alcohol Syndrome (FAS) or Neonatal Abstinence Syndrome (NAS) to the eighty year-old who became addicted through prescription drugs. Each is as unique, and to be treated as distinctly, as the hidden DNA characteristics they inherited, but there are events in their lives, turning points, which have altered their course in life, which appear to be characteristically common to the addictive process.

These are their stories, albeit anecdotal. Perhaps the general public will obtain a greater understanding of "the addict" by listening, just as I did.

She said she came from a pretty good family. Her parents were from the old country — central Europe —and were hard working. They hated drugs and drug addicts:

> *"I was ten years of age, watching TV and saw these programs on drugs. I was curious, I guess. By the time I was twelve I was experimenting with marijuana, and by the time I was fifteen I was a heroin addict. I'm still an addict, but on the methadone program. I've been clean for three years now. I've got two kids. I'm not married and I live on social welfare."*

Later on she indicated that her dad was "a tyrant."

He said his "old man" and his mother were alcoholics. In fact, so were his uncles. They were happy all the time; that's because they were drunk all the time:

> *"There's no way I was gonna touch alcohol. No sir. I went straight into drugs. LSD, marijuana, coke, and heroin. Yes, I'm an addict. I started all this when I was twelve years of age. I never finished school — couldn't really concentrate in the classes. I've had various jobs, but they don't last."*

Another spoke of a lifetime of addiction:

> *"I'm sixty-six years of age. I've been an addict since the forties. I robbed banks in the fifties and sixties. It was the rush of the hold-up and then I blew it all on the rush I got from the needle. I spent most of my life in the Pen, and when I'd get out I'd head right down for a fix. I'm not gonna quit now. I like the stuff. I boost $700 a day to maintain my habit. I've got a good 'old lady' now. She looks after me. By the way, if you're in town this Friday, you could come to court with me. I got caught boosting a leather jacket and my partner and I are appearing."*

His buddy is 58 years old. They are characterized locally by the police as "the grandfather gang."

Then there's the thirteen year old boy who was kicked out of his house, onto the street, by his father The father caught him using his marijuana. And the fourteen year-old who stole some of his dad's cocaine and was then similarly kicked out onto the street. He was told he could come back when he replaced the stuff he had taken. And the five year-old boy whose father caught him playing with matches in the back yard. His dad taught him a lesson by dragging him into the house and placing his bare hand on a hot element on the stove. Twenty three years later he is an angry young man, addicted to heroin, in recovery at a private house. And the eleven year-old girl who shyly visited the police station and asked the police officer to help her parents who were using drugs. The officer did so by discussing the problem that the parents were causing their daughter, all the while ensuring she was protected from any retaliation.

While addiction is more complex than mere personality characteristics, the common thread that appeared to be present in each addict was his or her sense of worthlessness. Not one had any self-esteem. They indicated they had been told consistently that they were "low-life," for want of translation, nothing more than a piece of "feces," and that they would never amount to anything in life. Psychologically they were not only damaged and defeated; they were destroyed. They felt sub-human, bereft of conscience and reason.

It seems important then, to make a distinction between the addict and the addiction. The addiction has an overwhelming power over the body and mind

of the addict. As one recovering addict indicated, he would do anything to obtain his next fix. His wife, children, job, friends -- nothing was more important than getting his next fix. It was an all consuming power over which he had no control. He didn't fear death because he didn't think about death. Not that he considered himself invincible, he simply had to have that fix. In effect, the addiction had completely destroyed his "free will." The power to choose that we normally take for granted was non-existent and, with that gone, any sense of responsibility on his part dissipated. He told me he had been given contracts to kill two people -- and he would have, had he not been able to obtain his drugs by other means. In the recovery stage, he indicated he couldn't really have gone through with the killings.

When I asked a recovering addict what caused him to change his life, his answer was simple: "I ran out of options; I hit rock bottom. I contemplated suicide, and that was unacceptable to me." He also said that until he reached that point, until he personally made a choice to face the pain of withdrawal and the realities of what was happening, no amount of external persuasion or force from others would make him abandon his addiction. Some others referred to "a power greater than myself" as instrumental in making that decision. It was a mystery how this freedom to choose sprang to life; the day before they had no free will and were well on the way to rock bottom.

Once one has some understanding of the addiction, it is easier to understand the addict —neither fully nor completely, granted -- but over and over I heard the expression: "Unless you've walked the walk, you can't talk the talk." However, the little understanding we do have does help in knowing why the actions of addicts are what they are. It remains difficult, however, for society to appreciate, let alone condone, those acts. I refer here specifically to anti-social behaviour and criminal activities.

Our knowledge, understanding, and appreciation of the addict must have some relationship to cause and effect in the real world of substance abuse. The actions of addicts become clearer when we understand and appreciate the addiction. The addiction itself has an origin: it is caused by various forces, both external and internal. The effects are physical, emotional, psychological, and social. The causes have no boundaries, no limitations: anyone, any age, any background, can be affected. Although some substance users may become addicted, others will not. The predisposing factors are multi-faceted and most often unique to the individual.

In order to maintain the addiction, the addict must adopt a philosophy where the end justifies the means. Shoplifting, stealing from home, robbing banks or pharmacies, assaulting others, and prostitution: any means, including anti-social and criminal ones, are justifiable in the minds of addicts, in order to satisfy that overpowering need. These activities are the consequences -- the effects -- of the addiction. First the causes, then the effects. The effects are, pure and simple, symptomatic of this human condition. Establishing the causes in each of these individual cases can be complex and confounding, but they are there, and they must be determined before we talk about treatment and possible solutions. The question that must be asked of society now is: "If we are to gain some ground in this difficult area of behaviour, are we going to treat the symptoms or the causes?"

Addicts harbour a great deal of fear. That may not be the impression presented when they are under the influence of the narcotic -- when they fight, steal, and commit armed hold-ups - but remember, they are masters at deception and persuasion. They have to be, to survive in their focused world. They have only one goal: the next fix. Any means to achieve that end is acceptable. They fear the authorities: the police, the health care professionals and the social workers. The authorities come between them and their needs: the next fix. In the case of the woman addict with children, she not only fears the force of the criminal law, the police, and the courts, but even moreso she fears the removal of her children by Social Services. She fears these possibilities more than death itself.

Others fear treatment and recovery. They take the drugs to relieve the pain of low self-esteem, the various abuses they suffer from the past and in the present, and the fear of what might happen at any time at the hands of the authorities. Many indicated that they wanted out of their situations, but were trapped. They couldn't predict the future, because all they saw and experienced was today, and this created a fear of the unknown when discussing detox and rehabilitation in the future.

Another point which some professional caregivers shared, and which I believe the authorities and the general public ought to understand, is that ofttimes when a young person (e.g. age 10-12) begins to experiment with any of these drugs, and when the addiction sets in and takes hold, the emotional growth of that person stagnates at that age. So, an addict might well be 35 years of age, but might act like a 10-12 year old child. The person's stunted

emotional development is a probable cause of social dysfunction amongst family, friends, and the community. Take, for example, the various abuses that currently hit the headlines and the court dockets. Were they the actions of a 10-12 year-old mind in a 35 year-old body?
Traditional drug treatment strategies based on education, treatment, and enforcement have not provided satisfactory solutions to the problems faced by illicit drug users. These interventions have been based upon older theories of addiction that consider all addicts to be alike and therefore tend to recommend a "one size fits all" solution to their problems. Whenever such a narrow perspective of addiction is embraced, the accompanying solution gains minimal acceptance within the addict community and is difficult to implement by the treatment community. The end result is inevitably program failure.

We must accept the fact that there is no single "magic bullet" solution to the serious health risks faced by illicit intravenous drug users. This includes the very serious risk of accidental fatal overdose. Illicit intravenous drug users are a complex group with diverse problems of varying degrees of severity. Any solutions intended to reduce risk must be multi-faceted and must fit within the social, psychological, and philosophical context of the individual user. In addition, these solutions must fit within a hierarchy of goals ranging from facilitating safe use of illicit drugs to facilitating detox and addiction treatment for those motivated to discontinue their drug use.

There are always those ready to detoxify and enter addiction treatment. Some addicts, after a prolonged period of time, "mature out" and become willing to change their behaviour, accepting and practising abstinence from illicit drug use. Researchers have studied how individuals build motivation that leads to recovery from addiction. In many instances, motivation building is a slow, subtle process occurring over many years. Addicts gradually acquire insight about the negative consequences of their behaviour. As insight strengthens, addicts are confronted with an emotional duality: they may be simultaneously attracted to and repulsed by their behaviour. They become intensely ambivalent about using drugs versus quitting.

Very often their ambivalence is crystallized and suddenly resolved in response to a significant crisis precipitated by some negative consequence of their addiction. At this point, the addict can be highly motivated to either quit spontaneously or enter a treatment program. The existing treatment system needs to be cognizant of the process of motivational change and every effort should be made to both recognize and accommodate individuals who are ready to discontinue illicit drug use. Barriers must be minimized and appropriate treatment resources made available to those who are ready for addiction treatment.

On the other hand, some users may be unwilling to discontinue intravenous drug use, but they may have demonstrated motivation toward reducing the risk associated with this activity. Many of these individuals participate in needle exchange programs, as well as practising safe injection techniques. Because this type of behaviour usually involves some contact with the health care system, opportunity exists to begin to build motivation for further treatment.

Understanding the addict and understanding the addiction: first knowledge, then understanding. Only then can we begin to appreciate what can and must be done for the addict and for society.

Chapter 2
THE EPIDEMIOLOGY OF OVERDOSE DEATHS IN BC

"Even death is not a deterrent - it's always the other guy who's going to die."

The Ministry of Health and the Provincial Toxicology Centre have provided useful analyses of the issues surrounding mortality and morbidity in these drug-related matters, as well as helpful ways and means to ameliorate the human and social problems arising out of substance abuse.

Examining the epidemiological studies and conclusions in concert, there is ample evidence to support the claim that the number of deaths attributable to illicit drug overdose is rising dramatically to the point where it might well be considered an epidemic. Street experience, police and coroner reports, drug seizure analysis, and post-mortem toxicology reports are mixed sources of information that indicate that there are diverse patterns of illicit drug abuse. Heroin and cocaine may be used singly, in combination, or sequentially, and by various routes, including smoking, snorting and injection, usually intravenously. Illicit use of methadone implies intravenous (IV) or combined drug use. Over the past few years, there has been a dramatic increase in the prevalence of intravenous drug abuse of heroin and cocaine as the preferred route of administration, at least as determined by autopsy findings in illicit drug-related death investigations. This trend is most evident for young adult males. In 1993, death attributed to illicit drug abuse was the leading cause of death amongst 30 - 44 year-old males ahead of Acquired Immunodeficiency Syndrome (AIDS) and suicide. Although the majority of deaths are concentrated in the Lower Mainland area, several other geographical areas are affected throughout Vancouver Island and the rest of the province.

The mechanism of death in relation to illicit drugs can be complex. Some instances relate to toxicity resulting from a single (usually high) dose. Others may result from the cumulative toxic effects resulting from multiple sequential doses. Many result from mixed drug interactions, from the effects of heroin and cocaine in combination or in sequence. In some instances, the death is clearly dose-dependent (i.e. a high dose has a high likelihood of causing death) whereas a few deaths are idiosyncratic, with random adverse reactions not related to the dose (e.g. hyperthermia in cocaine abuse). Of increasing significance are the high percentage of deaths in heroin users who are simultaneously ingesting alcohol.

There are also indications from coroners' death investigations that young people, who have been called "naive users," or relatively novice drug abusers are an especially at-risk population. Anecdotal evidence frequently suggests that these users may have been cocaine abusers who by accident or intent purchased and used heroin and subsequently succumbed to an inadvertent heroin or mixed drug overdose. "Partying" with heroin and alcohol has also resulted in a significant numbers of fatal outcomes.

Deaths due to the use of illicit drugs have become epidemic in British Columbia in recent years, increasing from 39 deaths in 1988 to 331 in 1993. This chapter focuses on the illicit drugs heroin, cocaine, and methadone, either used alone or in combination with each other or alcohol. Prescribed morphine and prescribed methadone, however, are not included in this study. The mortality and related information used in this chapter is based solely on the Coroner's determination of cause of death, which incorporates the following:[8]

A) **Pathology /Toxicology**
The opinion of the pathologist who performed the autopsy.

In cases where a drug is suspected or where the cause of death is not established at autopsy, toxicological assays** are performed. (The sequence of assays may be modified by available history.)

AND

B) **Scene Investigation**
Investigation at the scene.

This includes witness statements, police information, and known medical history of the deceased.

An epidemiological description of the problem follows, which was developed from basic data*** provided by the British Columbia Coroners Service, for the period 1988 to 1993. The general trend over time is depicted in Figure 1, with the number of deaths increasing by over 800 percent over the last six years, and doubling over the last two years.

The increasing death rate has occurred in both males and females, although the absolute death rate in males is considerably higher. The ratio of male deaths to female deaths was 4.5:1 in 1993. The crude death rate by gender is shown in Figure 2. The use of a rate allows the increasing number of deaths to be related to the overall annual growth of the population, and confirms the occurrence of an epidemic of illicit drug deaths.

Figure 1: Illicit Drug Deaths, Annual Cases, BC, 1988 -1993

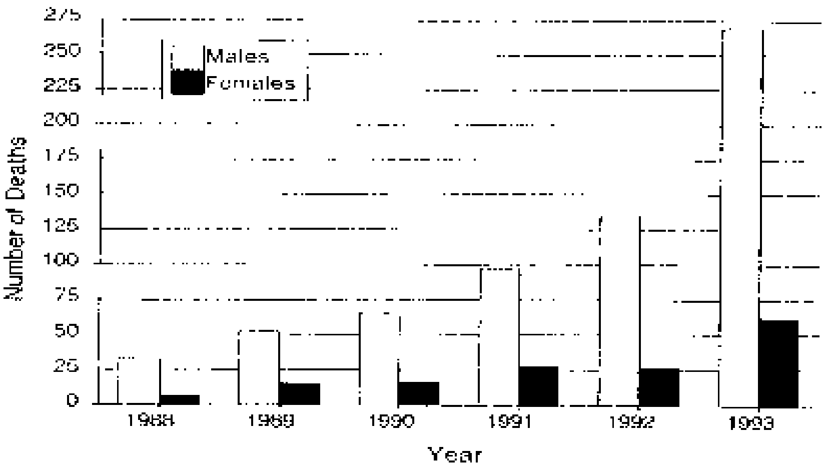

* Personal communication, Office of the Chief Coroner, June 9, 1994

** Deaths may be attributed to an overdose where cocaine and its metabolite, ormorphine, the metabolite of heroin, or methadone, were present in the blood of the deceased at or near time of death, in concentrations above the minimum lethal level.
Minimum Lethal Levels: (Provincial Toxicology Lab)
- Heroin: Blood morphine concentration greater than 0.05 mg/L
- Cocaine: Combined blood cocaine and benzoylecgonine concentrations greater than 1.2 mg/L.
- Methadone: Blood methadone concentration of greater than 0.4 mg/L.

*** Unless otherwise specified, all data utilized in this report was provided by the BC Coroners Service, as reported to May 18, 1994.

Figure 2: Illicit Drug Deaths, Crude Rate Per 100,000 Population, Males and Females, BC, 1988 - 1993

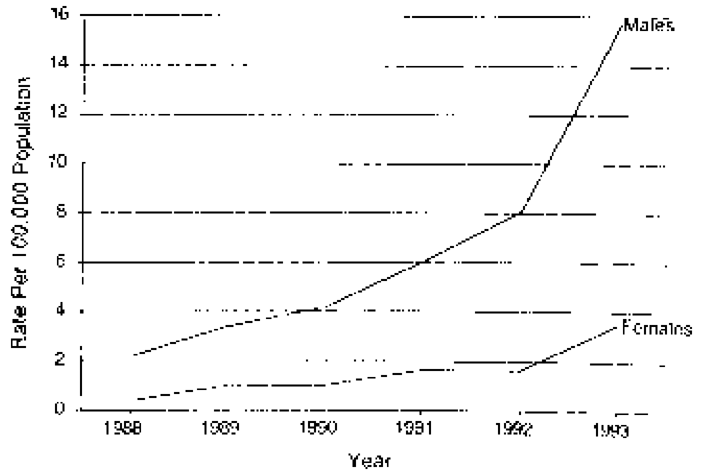

Age/Gender Characteristics
On examining the deaths by age in more detail for 1993, the highest death rate occurred in the 35-39 year age group for males and the 30-34 year age group for females, as indicated in Figure 3.

On examining the increase in deaths by sex and age group (see Figures 4 and 5), the data indicate that the deaths increased moderately in all age groups until 1993, when a major increase occurred in the 30-44 year age group in both sexes, with the rate for males being greater than that for females.

Figure 3: Illicit Drug Deaths, Age SpecificRates Per 100,000 Population: Males and Females, BC, 1993

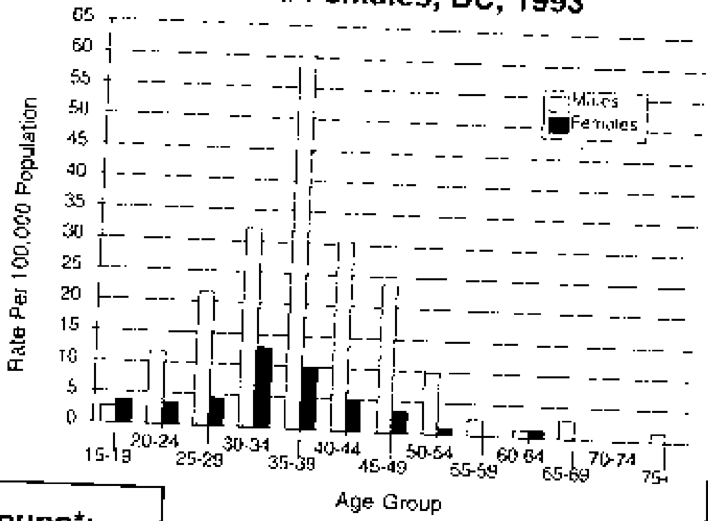

Figure 4: Illicit Drug Deaths By Age Groups*: Males, Rate Per 100,000 Population, BC, 1988-1993

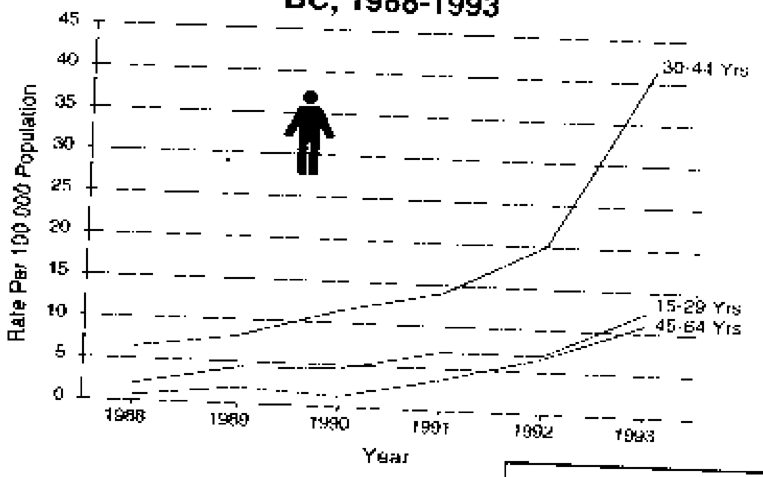

*Selected age groups were omitted due to no cases (0-14 yrs.) or very few cases (65+ yrs.)

Figure 5: Illicit Drug Deaths By Age Groups*, Females, Rate Per 100,000 Population, BC, 1988-1993

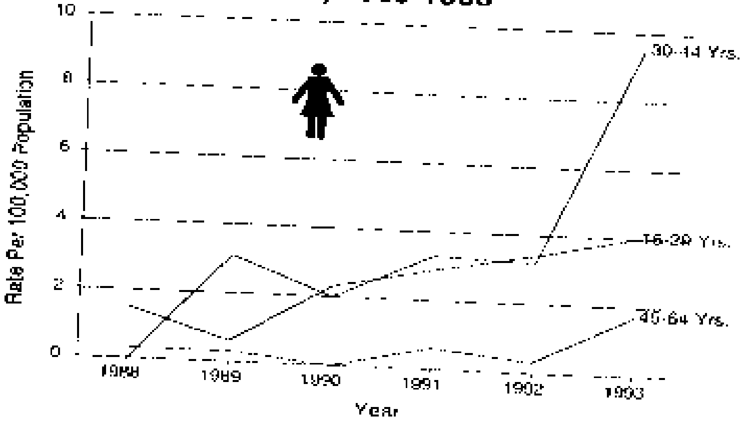

*Selected age groups were omitted due to no cases (0-14 yrs.) or very few cases (65+ yrs.)

Figure 6: Leading Causes of Death*Males, 30-44 Yrs. Rate Per 100,000 Population, BC, 1988 - 1993

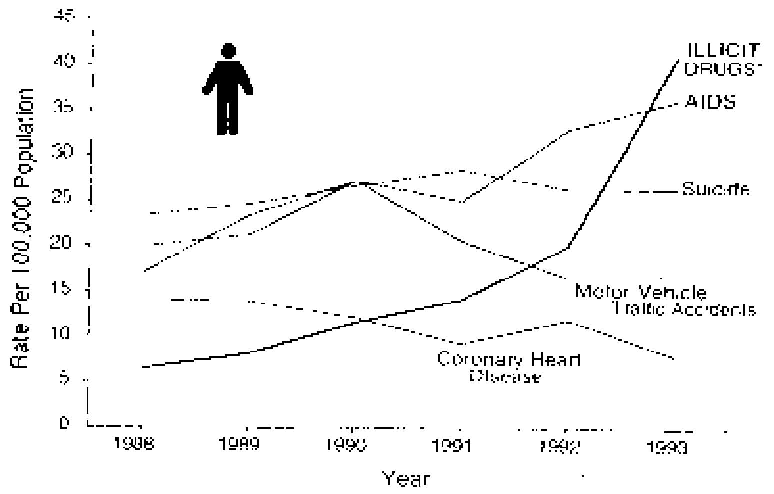

Comparison With Other Major Causes of Death

As the 30-44 year age group has experienced the most significant increase in illicit drug mortality, other major causes of death for that same age group in the population were examined for each sex (see Figures 6 and 7). These data show that illicit drug use has become the leading cause of death for both males and females in this age group in 1993.

Geographic Distribution

Illicit drug deaths have occurred in all areas of the province, as indicated in Table 1 and Figure 8. However, the largest proportion (60 percent) of cases have occurred in the city of Vancouver.

Figure 7: Leading Causes of Death*, Females, 30-44 Yrs. Rate Per 100,000 Population, BC, 1988 - 1993

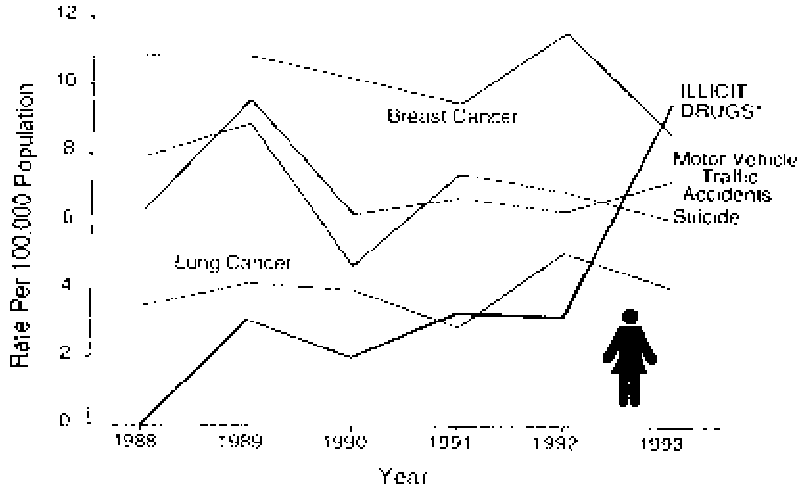

* Data provided by BC Coroners Service
Original Data Source: Division of Vital Statistics, BC, Ministry of Health
Data for 1988-1992 acquired through: Health Planning Database, BC Ministry of Health

Table 1: Illicit Drug Deaths by Health Unit of Occurrence BC, 1988 to 1993

Health Unit	1988	1989	1990	1991	1992	1993
East Kootenay	2	1	1	0	0	1
Central Kootenay	0	0	0	1	0	1
North Okanagan	0	1	0	1	0	3
South Okanagan	0	0	1	0	0	3
South Central	0	0	1	4	3	4
**Thompson-Okanagan-Kootenay**	**2**	**2**	**3**	**6**	**3**	**12**
Upper Fraser Valley	1	1	0	4	4	8
Central Fraser Valley	0	2	0	2	2	4
Boundary	3	2	2	7	11	19
Simon Fraser	2	2	7	6	10	11
**Fraser Valley**	**6**	**7**	**9**	**19**	**27**	**42**
Cariboo	0	0	1	1	0	1
Skeena	1	0	0	4	1	9
Peace River	0	2	0	0	0	1
Northern Interior	0	1	1	2	1	9
**North**	**1**	**3**	**2**	**7**	**2**	**20**
Coast Garibaldi	1	0	0	3	3	1
Central Vancouver Island	0	0	3	6	3	13
Upper Island	0	1	1	2	9	6
Capital Regional District	4	7	5	3	6	21
**Island-Coast**	**5**	**8**	**9**	**14**	**21**	**41**
Burnaby	4	3	6	7	9	9
North Shore	2	1	2	1	3	3
Richmond	1	1	1	3	6	4
Vancouver	18	42	50	67	91	200
**Metro Vancouver**	**25**	**47**	**59**	**78**	**109**	**216**
**B.C.**	**39**	**67**	**82**	**124**	**162**	**331**

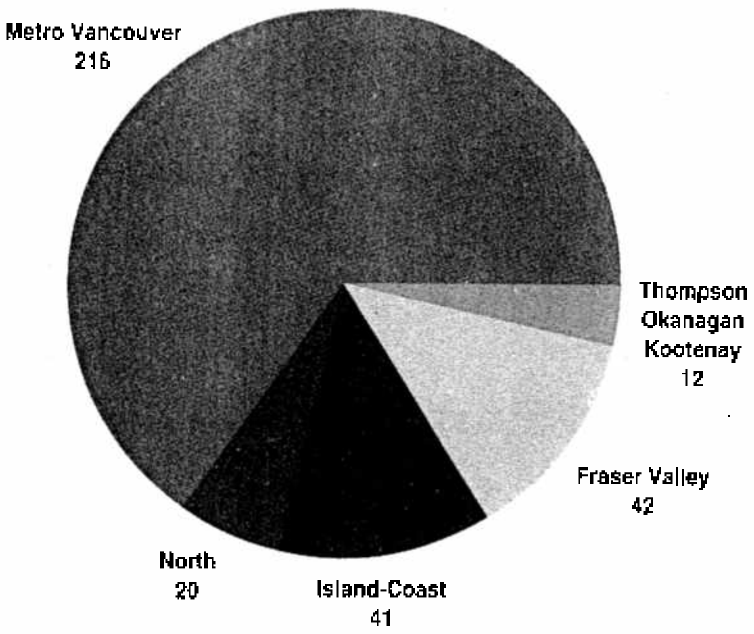

FIGURE 8: Illicit Drug Deaths by Region of Occurence, B.C., 1993

The information in Figure 8 was compiled by the area of the province in which the death occurred. In order to make a more valid comparison of the geographic areas of the province, the 1993 cases were examined according to the area of residence of the deceased (see Figure 9). This excludes those deaths for people with unknown addresses (N = 9) or out-of-province addresses (N = 5). For reasons noted earlier, deaths occurring in people aged 65+ (N = 3) were also excluded.

The provincial average death rate for ages 15-64 years for 1993 is 14.2 per 100,000, a rate which is exceeded only by Vancouver (49.2) and Skeena (17.4). However, on comparison with the median of 5.2 per 100,000, the following areas are also of concern, from higher (11.8) to lower (6.6): Upper Island, Burnaby, CRD, Boundary, Northern Interior, Central Vancouver Island, Simon Fraser, and Upper Fraser Valley.

Figure 9: Illicit Drug Deaths By Health Unit of Residence, Rates Per 100,000 Population, Ages 15-64 Years, BC, 1993

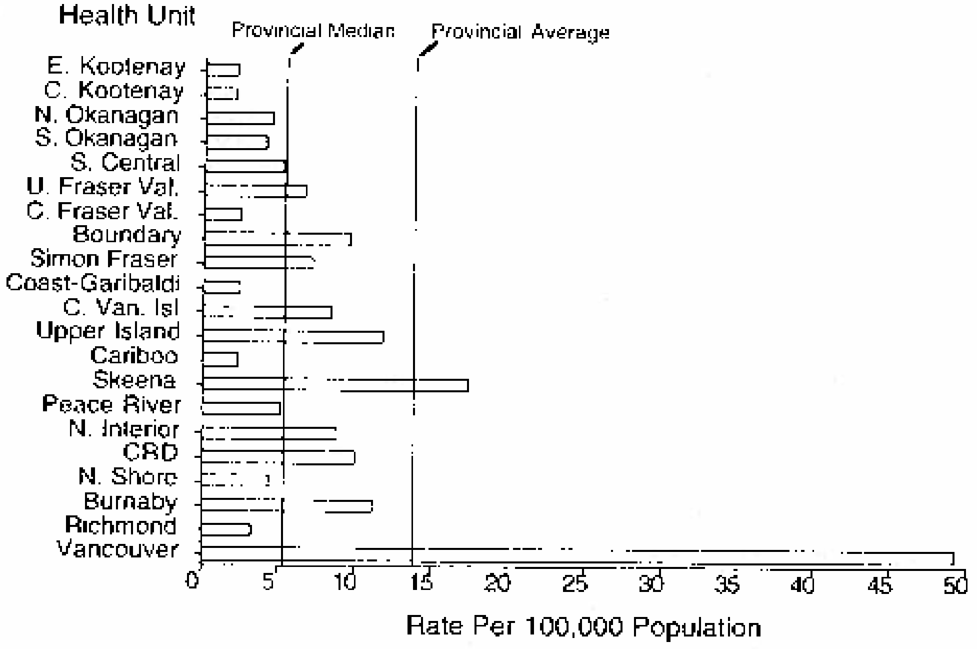

FIGURE 10: Illicit Drug Deaths by Type of Drug, B.C., 1993

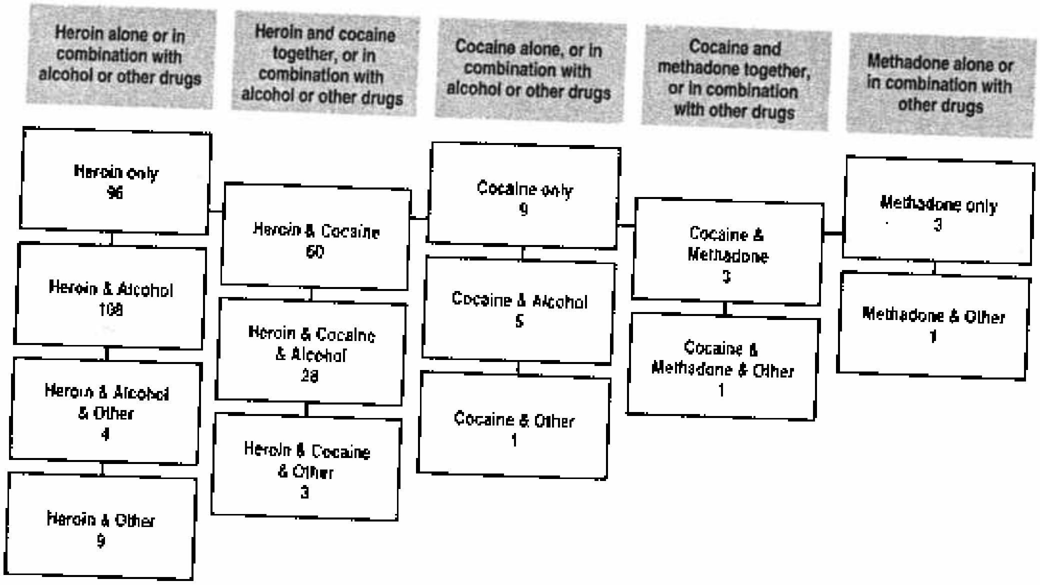

Note. "Other" drugs may include cannabinoids, amphetamines, LSD, PCP, etc.

Type of Drug Use

The major illicit drugs used are heroin and cocaine, either alone, in combination with each other, or in combination with alcohol, methadone, or other drugs. The pattern of drug ingestion associated with lethal outcome, as determined by the BC Coroner's Service for 1993, follows in Figure 10.

Figure 10 chart indicates that lethal outcomes were associated with the following patterns as shown in Table 2.

TABLE 2: Pattern of Lethal Illicit Drug Use, B.C., 1993

Drug Associated With Death	No. of Deaths	Percent (%)
Heroin	298	90
Combinations	223	67
Alcohol	145	44
Cocaine	106	32
Methadone	8	2
Other Drugs	19	6

Note: Categories not mutually exclusive

Heroin was associated with 90 percent of the deaths, while alcohol featured in almost one-half and cocaine in one-third of the deaths. In two-thirds of the deaths, the victims used more than one drug.

The Provincial Toxicology Centre Table of Morphine (Heroin) and Ethanol Deaths within British Columbia (Table 3) represents a subset of individual heroin users who showed toxicologic evidence of mixed consumption of heroin and ethanol at or before the time of death. Blood ethanol greater than 0.02 percent correlates with the equivalent of at least one drink of beer, wine, or spirits being consumed or residually present in the body within an hour prior to death. Blood ethanol greater than 0.10 percent correlates to concentrations in marginal excess of the 0.08 percent level consistent with legal impairment; greater than 0.20 percent is correlates to at least double the legal impairment concentration.

TABLE 3: Morphine (Heroin) and Ethanol Deaths, B.C. 1993

YEAR	Blood Ethanol > 0.02	Blood Ethanol > 0.10	Blood Ethanol > 0.20
1988	13 *(30%)*	9 *(21%)*	4 *(9%)*
1989	16 *(29%)*	13 *(23%)*	1 *(2%)*
1990	27 *(43%)*	19 *(41%)*	5 *(9%)*
1991	53 *(48%)*	36 *(32%)*	20 *(18%)*
1992	68 *(44%)*	41 *(27%)*	12 *(8%)*
1993	170 *(55%)*	126 *(40%)*	43 *(14%)*

Note: Provincial Toxicology Centre Data

The above data suggest that over the past five years there has been an absolute increase in the number of individuals who have died by various means while under the influence of both heroin and alcohol in any concentration. The absolute number of individuals has increased from 13 in 1988 to 170 in 1993. There has also been an absolute increase in deaths where individuals were legally impaired due to ethanol, ranging from nine in 1988 to 126 in 1993. There has been an increasing tendency for heroin users to co-ingest alcohol, as reflected by an increase from 30 percent in 1988 to 55 percent in 1993. These percentages represent the relative number of deaths occurring in conjunction with mixed heroin and ethanol use to that of all deaths involving heroin.

Moreover, there has been an increasing tendency for those individuals to be legally impaired due to ethanol, with this subset having increased from 21 percent to 40 percent of all heroin-associated deaths.

This BC experience is similar to that reported in Germany at the 13th International Association of Forensic Sciences by W. Schulz-Schaeffer, in which there was an emerging subset of deaths occurring in heroin users with high blood alcohol concentrations. A sociological review of this subset in the German study suggested that the majority were long-term alcohol dependents who were developing a secondary heroin abuse pattern and were at high risk of death due to the double dependency, with an average of two years of double drug abuse elapsing prior to death. The BC and European data also suggest that mixed intoxication with heroin and ethanol over 0.10 percent increased the lethality over use of heroin alone, probably due to the additive effects of both agents as central nervous system depressants.

It should be recognized that this epidemic of drug related deaths represents only a small fraction of the total impact that illicit drug use has on our society. This epidemic of deaths does not imply necessarily that there are rising numbers of intravenous users of illicit drugs in BC, as epidemiologists do not have data on the number of users. However, this rapidly rising death rate draws our attention to the fact that illicit drug use impacts everyone in BC and that the expenditures currently being made on controlling the use of illicit drugs are not having the desired results. The immediate apparent cause of this epidemic is the availability of unusually pure heroin resulting in inadvertent overdoses. While this will most likely, to some extent, be self-correcting as users gain more knowledge and legal interventions are pursued, there are underlying issues that should be addressed. Many of the harmful effects of illicit drug use arise from the associated criminal activity and lack of access to appropriate services.

In summary, deaths associated with the use of illicit drugs have increased markedly over the last six years, and can be considered to have reached epidemic status. Illicit drug use has become the leading cause of death in both males and females aged 30-44 years. While the area with the highest mortality rate is Vancouver, all areas of the province have experienced illicit drug deaths, particularly other metropolitan areas in the Skeena, the Northern Interior, and on Vancouver Island.

The majority of deaths occur in males and involve heroin, either alone or in combination with alcohol and/or other drugs. Cocaine is involved with one-third of the deaths, either alone or in combination with alcohol or other drugs. Multiple drug use was involved in two thirds of the cases. Although the statistics do not show whether the people who died from heroin use were occasional users or frequent users, it would appear that frequent users develop tolerance to heroin and are less likely to overdose. This point highlights the fact that occasional users may well have an increased risk of dying from using drugs.

The profile of a typical victim was a male resident of Vancouver, aged 35-39 years, who died while using heroin in combination with alcohol and/or another drug. It is a tragedy that this potentially preventable cause of death has claimed the lives of so many in their prime years, and it is hoped that this epidemiological review will assist in the development of effective preventive strategies.

Finally, an indirect collateral effect caused by the epidemic of drug deaths has been the prodigious administrative impact on the Provincial Toxicology Centre at Riverview Hospital. The centre, which is funded on a fee-for-services basis by the Coroners Services, has had its resources and personnel taxed heavily. The cost of full drug toxicology screens are significant and, in the present context of restraint, there are alternatives but quality of data may have to suffer. As well, there is an immediate need for further research beginning with a need to study the inter-relationship between alcohol and polydrug use in fatalities.

ADDENDUM UPDATE FOR 1994 ILLICIT DRUG DEATHS

Data from the Provincial Toxicology Centre for the first and second quarters of 1994 indicates the absolute number of coroners cases in which heroin, cocaine, or methadone have been detected in concentrations exceeding minimum lethal concentration. The cases are not mutually exclusive. That is, one subject could have more than one drug in combination. Without review of the coroner's file, the cause of death may not be simply attributable to drug abuse alone (i.e. the cause may include falls, motor vehicle accidents, etc.).

The Bureau of Dangerous Drugs (Health and Welfare Canada) analyzes all police drug exhibits for British Columbia and Alberta. They indicate that the purity of heroin on the streets is gradually dropping from a high of approximately 80 percent early in 1994 to approximately 54 percent in July 1994. They also report that designer drugs have not been a major factor in their analyses of drugs in BC.

Table 4: Provincial Toxicology Deaths Within BC, 1994 Update

	Heroin[1]	Cocaine[2]	Methadone[3]
1994 (to June 30)	162	59	7

Note:

1) Heroin – Blood/morphine concentration greater than 0.05 mg/L at or near time of death. Heroin NOT NECESSARILY CAUSE OF DEATH.

2) Cocaine Combined blood/cocaine and benzoylecgonine concentrations greater than 1.2 mg/L Cocaine NOT NECESSARILY CAUSE OF DEATH

3) Methadone – Blood concentration of methadone greater than 0.4 mg/L. Methadone NOT NECESSARILY CAUSE OF DEATH.

An August 1994 consultation with the coroners and the Provincial Toxicology Lab confirms that the numbers of illicit drug-involved deaths are levelling off. In January through March of 1994 the deaths continued at a high level. However, there has been a settling in the months of April to July 1994. These trends will continue to be monitored and reported.

I therefore RECOMMEND THAT the Ministry of Attorney General:

2. Set aside funds for expansion of services for drug screening by the Provincial Toxicology Centre.

I therefore RECOMMEND THAT the Ministry of Health:

3. Undertake research into the inter-relationship between alcohol and heroin in overdose deaths.

Chapter 3

PUBLIC HEALTH/TREATMENT

Part A
EMERGENCY SERVICES

"We've seen them at their worst and at their best ... we get to know them ... then, after eight to ten years, one day they come in dead."

When the call comes in to 911 in overdose cases, it is truly a matter of life or death. Invariably all three emergency services (police, fire, and ambulance) respond. Whoever gets there first must attend to the patient. The *First Responder* program enables the police and fire services in British Columbia to deal with the circumstances in that initial setting.

When the problem is determined to be substance abuse — in particular, a drug overdose — the responsibility for medical care and transfer lies with the Emergency Health Services (Ambulance) paramedic. Some of the patients are dead at the scene. Others are on the verge of death, appearing lifeless to the untrained eye. The miracle drug used in these situations is naloxone or Narcan. It brings them "back to life": not really, but close to it.

Until mid-1992, only ambulances with advanced life support paramedics (EMA 3) were allowed to use Narcan. At that time, six major centres in BC had access to Narcan. In mid-1992, paramedics with an EMA 2 standing were permitted to administer Narcan. As there were EMA 2 attendants in over 100 British Columbia communities, availability and use naturally rose. For instance, prior to mid-1992 there were only seven ambulances with EMA 3's and Narcan, out of a total of 40 ambulances, in the Lower Mainland. Now all 40 ambulances can administer Narcan when required! BC Emergency Services have taken timely steps in providing a wider availability of Narcan through supply and training of EMA 2 paramedics to administer this antagonist.

One of the benefits of Narcan is that it can be, and is, administered for any opiate overdose. So, if there are opiate symptoms, it can be used. If the patient does not respond to the Narcan, then opiate overdose is unlikely and other causes need to be determined.

Narcan usage to February 7, 1994 is shown in Table 5 and Figure 11.

TABLE 5: Narcan Usage in B.C., 1990-1994

FISCAL YEAR	NARCAN USAGES
1990	133
1991	162
1992	468
1993	1002

FIGURE 11: Narcan Protocols in B.C. Ambulances

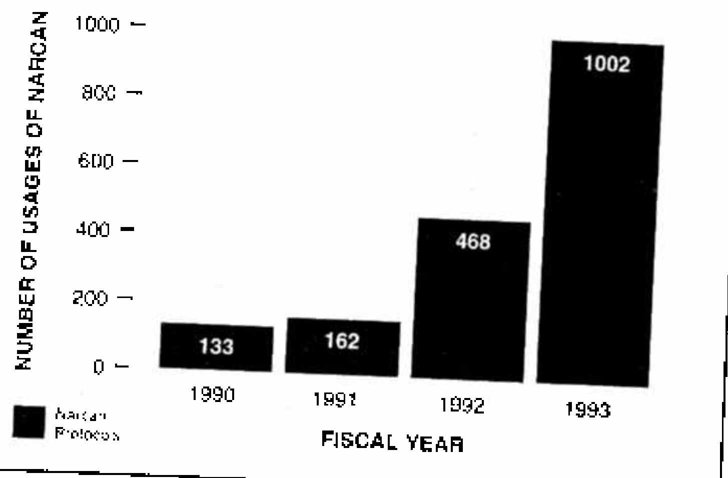

Eighty percent of Narcan applications are now used in the Greater Vancouver area. This ties in with coroners statistics which show that almost 90 percent of the deaths in BC due to drug involvement occur in the Greater Vancouver Regional District (GVRD). Given the statistics we are looking at, BC has the busiest emergency drug overdose rescue effort in Canada. Emergency Health Service studies show an almost even distribution of Narcan protocols throughout the week. Thus, weekends or holidays do not show up as significant in terms of applications of Narcan, even though actual death statistics show elevations for the second last Wednesday and Thursday of each month.
Given the antidote effects of Narcan, I heard a number of arguments concerning making this medication more available. These range from users wanting to have their own supply to the more pragmatic suggestions of making Narcan available from trained personnel through street clinics and needle exchanges.

Time and again, I encountered the need for better exchange of information between street clinics, needle exchanges, emergency services, hospitals, and a range of law enforcement and service providers, as well as addicts themselves. A drug testing and information hot line was proposed as an essential ingredient of an effective drug response system.

One of the serious risks of heroin use is the danger of overdose, a risk that is made all the more real - and that much more frequent - as a result of the wide fluctuations in purity that plague the street heroin market. Since the gap between an intoxicating dose of heroin and an overdose is so slight, overdoses are a frequent result of those temporary fluctuations or the frequency of dosage resulting in a cumulative effect.

There are both similarities and differences between heroin or narcotic overdoses and overdoses of other depressant drugs. While a heroin overdose involves the same respiratory depression, from slow to nearly non-existent breathing and coma, as any other depressant drug overdose, one tip-off to a narcotics overdose is a tight constriction of the pupils of the eyes to near-pinpoint size. Heroin is one of a dozen or so substances that causes constriction of the pupils. Due to the serious depressant action of narcotics in general and heroin in particular, a heroin overdose is a major medical emergency and should be treated as such. Old line junkie "quick-fix" remedies, such as injecting an overdose victim with milk or salt water, are dangerous and without medical benefit. Likewise, the notion of placing the body in a bathtub of cold water has no potential for recovery. These are not sound medical practises. Do they occur? Yes, of course, and people die as a result, too.

The new breed of narcotic antagonists (which plug into endorphin locks even better than heroin, and thus displace heroin from their binding sites), are now available and can reverse the effects of an overdose almost instantly. Drugs such as Narcan are only available through legitimate medical channels. That is one reason why it is so important to get help immediately in the event of an overdose. All the wonder drugs in the world are not going to help if the victim does not receive prompt, professional medical attention.

The paramedics I contacted projected a very professional, sensitive, and caring attitude toward these patients, but two points were made quite clearly. In high volume, repetitive situations, some individuals may well become insensitive. Likewise, following the injection of Narcan - - and remembering that the effects are time limited --- some of these patients can become hostile and violent. Some refuse further assistance, such as transportation to the hospital for follow up treatment. That is not unusual.

Emergency physicians and nurses face the same problem when the patient is admitted to emergency. It is not uncommon for hostility to turn to assault on the nurse or doctor: not a very pleasant response toward those committed to help. It is significant to understand and appreciate that Narcan has a short half life. It loses its effect after an hour or so; the patient can subsequently relapse into respiratory depression. I note this because it is not uncommon for the patient to immediately leave the emergency centre. Hospital personnel were clearly frustrated with this band-aid treatment: the returning, revolving door and the violent, hostile patient. Again, there is the potential for reluctance or refusal, which can result in dire consequences.

It was also pointed out, from experience, that "this is not a disease of the down-and-out." One paramedic related how he had attended "more deaths than I care to count" and that overdose calls were attended "in Shaughnessey and the equivalent in Victoria." He also undertook to poll his colleagues on the problem and possible solutions. Noting that there were "no easy answers," they ranged from "nothing can be done" to legalization and decriminalization.

Another northern paramedic stated that when they try to educate the repeaters on what they are doing to themselves, it does not seem to affect them:

> *"It's like they don't really believe you. Some are scared by the deaths, but most of them it doesn't bother at all. Once resuscitated, they don't want to go to the hospital, even though we explain the short half life of Narcan ... it doesn't faze them at all."*

Interestingly, it was not uncommon to hear remarks like: "I'm surprised at the number of doctors who are not educated or informed of these addiction issues." Many considered education to be the key to opening minds and doors. One said that hospital emergency staff need to use recovering addicts as a resource:

> *"Let's get them while they're down. Keep recovered addicts involved. You don't have to patronize them. They'll earn their keep."*

Indeed, not a pleasant duty to perform, but as one attendant remarked: "It's our job and there are risks: assaultive behaviour, the risk of Human Immunodeficiency Virus (HIV) and AIDS, and so on." A coroner remarked that it is not very exciting and romantic when nothing can be done but to remove the body to the morgue. He was relating how his task so conflicted with the public portrayal of the drug world as often glamorous and exciting. Indeed.

In summary, Emergency Services has moved strategically to make Narcan available through paramedics, thus increasing their lifesaving efforts. As the epidemic continues, more needs to be done. We need to expand the number of trained personnel to administer Narcan at a wider variety of venues, particularly those where street addicts come in contact with health care and aid agencies (e.g. needle exchanges and street clinics). Those working in emergency services and health care need to know more about the addict and addiction. Information sharing between agencies needs to be increased and the general public needs to be informed about calling for emergency assistance as soon as an overdose is suspected. Finally, there may well be a role for recovering substance abusers within emergency health care.

I therefore RECOMMEND THAT the Ministry of Health examine existing programs within the Emergency Health Services with a view to:

4. Providing an educational component for paramedics respecting the addict and addiction, in order to better understand and cope with this type of patient;

5. Educating the public on the importance of calling 911 for an immediate response in suspected overdose situations;

6. Extending the availability and application of naloxone (Narcan), or other narcotic antagonists, at critical locations and in addition to existing medical protocols.

I therefore RECOMMEND THAT the College of Physicians and Surgeons:

7. Liaise with Regional Health Boards respecting an education component for emergency physicians and nurses, in order to better understand addictions and cope with the addict patient;

8. Liaise with Regional Health Boards to consider deploying properly-assessed recovering addicts in a consultant/aide capacity to emergency physicians and nurses, when an overdose patient is brought to the hospital.

Part B
HARM REDUCTION

"We need a different approach ... we must let something go to pick up something new. We need to move from a turbo-prop to a jet with the same funding in this time of budget cuts."

In 1987, the Canadian government adopted harm reduction as the framework for Canada's National Drug Strategy. The primary concept underlying the harm reduction model is to reduce the negative consequences associated with drug use, rather than the traditional focus of reducing the prevalence of drug use. Needle exchange is an example of this approach, where it is readily acknowledged that the spread of HIV and other infectious diseases (e.g. Hepatitis B and HTLV-I/II) through shared injection equipment is a far greater danger to the individual and to the public health in general, compared to the harm associated with drug use. As a harm reduction strategy, needle exchange is considered to be a sensible and pragmatic approach to improving the overall health of injection drug users.

According to researchers, epidemiologists and the Harm Reduction Committee at Vancouver's St. Paul's Hospital, the world-wide development of comprehensive needle exchange programs has been associated with lower HIV incidence rates and crime rates. Despite initial concerns that needle exchange would appear to condone drug use, the researchers indicate there is no evidence in the literature, or from experience in the field, that the introduction of needle exchange programs has led to an increase in drug use, or a lowering of the average age of first time Intravenous Drug Users (IDU's). In fact, it is becoming recognized that needle exchange is often the first line of approach for IDU's who have opted to seek drug counselling and referral to detox clinics.

I was informed that Vancouver established the first needle exchange in Canada through the Downtown Eastside Youth Activities Society (DEYAS). Although DEYAS was incorporated in 1984 and the exchange opened officially in 1989, the society began handing out needles in 1988. Now there are over thirty community-based needle exchanges currently operating across the country. The growth of needle exchange programs in this province has been a direct response to the menace of the spread of HIV infection and its follow-up companion disease, AIDS. Most needle exchanges also instruct clients in safe drug use and injection techniques, as well as making referrals to everything from alcohol and drug counselling to HIV testing to medical intervention to Social Services. In practical terms, this provision of information and equipment may be one of the only effective vehicles to prevent a greater epidemic of HIV and the resultant AIDS deaths.

The inquiry was advised of the fact that in many US jurisdictions, possession of injection equipment is still a criminal offence. Thus, amongst users there is added incentive to be clandestine and share intravenous equipment "rigs" or "fits" to reduce the exposure to police intervention and arrest. As a result, in New York, there is a much higher incidence of HIV and AIDS conversion amongst the IV drug user group. According to *The Public Health Impact of Needle Exchange Programs in the United States and Abroad*, a study prepared for the US Centers for Disease Control and Prevention by the School of Public Health, University of Berkeley, HIV prevalence in New York City reached an estimated 60 percent before levelling off in about 1984. I was advised that the conversion rate in Vancouver is approximately 2.4 percent in the at-risk group of IDU's.

Let me say a few words on the subject of needle exchanges. Each needle exchange I visited in this province differed to some degree from the others. All were operated by dedicated people, some with medical backgrounds, some with addiction backgrounds, some with a mixture of both. The stories of the operators are a clear reflection of their motivation to assist others. The goal is "harm reduction." Their motto seemed to be:

> *"Use if you must ... use safely ... and we'll help, or show you where to get help to quit if you ever wish to."*

The needle exchange operators have a non-judgemental, non-threatening approach to the addict.

The DEYAS Needle Exchange is the largest in Canada, with just over 5000 addicts registered on their computer. In 1993, approximately 817,000 needles were given out with an almost 100 percent return rate. During the first half of 1994, the Vancouver Exchange gave out approximately 520,000 syringes with a return rate of 102 percent. The director and associate manager acknowledge that they are only capturing 40 percent of the addicts at this downtown location near "the Corner" (Main and Hastings). They proudly point out that this is the most cost effective needle exchange in the country, perhaps North America, and that the percentage of IV drug users who have contracted HIV or converted to AIDS is very low in comparison to other large cities in the U.S. Indeed, the Vancouver conversion rate is also the lowest of any major city in Canada. It is interesting to note that in a recent evaluation by an independent group of researchers at the Center for Disease Control (CDC) in Atlanta, the DEYAS Exchange was ranked as one of the best needle exchange programs in North America in terms of the proportion of IDU's served, the number of needles exchanged, the percentage of needles recovered, and the overall cost effectiveness.

Funding continues to be a problem, with across-the-board provincial health care cuts also affecting their operations at a time when the need continues to climb. The DEYAS Exchange alone registered approximately 1,500 new clients in the first six months of 1994. Funding reductions have already resulted in restricting the number of condoms distributed and will result in having to drop some of their services to addicts. Condom distribution to street-involved youth and adults is an ancillary harm reduction service aimed at reducing the risk of Sexually Transmitted Diseases (STD's), including HIV infection. The funding restraints will also mean less time to talk to and counsel those who are interested in seeking help and "getting clean"; other areas will also likely suffer.

In terms of follow-up for those who do ask for help, the director of the Vancouver Needle Exchange says:

> *"For anyone asking for help to get into treatment ... we're missing it here. The `window of opportunity' is brief as it is. However, there are not enough detox beds, there are long waiting lists, and these people can't wait. We need more detox beds to refer people to from the needle exchange, for both youth and mothers with children."*

At another centre, the director indicated that he had become habitually involved with drugs as a young man sharing an apartment in Quebec with a friend who started using it as a "shooting gallery":

> *"I would do his `cottons' until I wanted my own `shit.' I would shoot anything ... my priority was escape ... met my wife in a shooting gallery. Scammed many people. Lost my wife and kid over four years of using. My veins were more important than my kid ... He was a `Saturday night special'; his mum and I were just trying to get high together. My first high was the best and I never again achieved that ... I liked the `nod,' I liked the crowd. I made a lot of dealers rich. Later, I lost my wife and kid. I also lost a lot of good friends ... Now I've been clean 20 years and my `high' now is pride in the Centre.*

> *"We are connected to the town council. The mayor is behind us. We have put sharps containers in police cars. One of the local judges came in and rode with us to understand the problem and said we were doing a great job. We speak at schools ... They can learn from us or they can learn from the bar crowd. Many social problems ... kids on the street escaping abusive homes, get pulled in. We need a Crisis line and 24 hour `outreach workers.' We need a drug and alcohol worker in a mall — available not just on a nine to five basis — and a youth clinic to deal with the runaways and a safehouse for native women and kids."*

He was one of many individuals I met who was operating a youth outreach program associated with a needle exchange. Enthusiasm seemed his key ingredient. It was also clear that most of these centres were underfunded and operating mainly with the contributions of unpaid volunteer labour. They indicated a need for more workers, 24 hour availability, and mobile units. They generally are street front operations in the center of larger cities. They need support resources to refer to when addicts indicate an interest in "cleaning up."

Many needle exchanges are managed by people who have public health experience and an interest in street

clinics, in getting health care to the needy. As one operator told me:

> *"I have to look at drug and alcohol abuse as serving a function ... They had to use drugs for a reason ... For our IV drug-using population, there are common denominators ... outstanding issues of physical and sexual abuse ... almost across the board. Coming from homes where parents were substance abusers also ties in ... most clients are poly-addicted."*

The needle exchange is also an ideal venue for interacting with users. Health care workers may work out of these locations to inform and assist. They may also use the opportunity to study the problem, come to understand the people who use drugs, and perhaps in the future gather street drugs for testing, so that information on drug quality and potential hazards can be disseminated quickly at street level.

I learned that AIDS Prince Rupert recently missed a funding application date and as a result their needle exchange program was placed in jeopardy and faced closure. It is currently soliciting municipal and corporate sponsors to keep the doors open. This is a sad state, considering the potential costs associated with increased risk of HIV infection and the potential harm to users.

Given the special non-judgemental, non-threatening stance of the needle exchange, this may be one of the only venues of neutral contact and communication between street addicts and the world of professionals and helping services. The long time adversarial relationship with police officials through the "War on Drugs" often sets up an irreversibly negative environment and destroys trust, leading the general population to condemn and reject not only life styles but lives as well.

The importance of the needle exchange being the conduit for information is immediately underscored when we learn that 40 percent of the clientele are illiterate. That is the case in Vancouver and one could assume this figure may be fairly accurate for other centres as well. With a large proportion of the needle exchange users unable to read, being able to communicate personally with informed caregivers becomes critical. The merits of written communication to this 40 percent are questionable.

In addition, the need for needle exchanges is underscored when St. Paul's Hospital reports that the number of IDU's being admitted with varying medical conditions is on the increase. As the number of IDU's requiring hospital admission increases, some inner city hospitals are examining the efficacy of providing a needle exchange to those patients who will use drugs while in the hospital. Otherwise, they can be a risk to themselves, health care staff, or visitors, by smuggling injection equipment into the hospital.

It is interesting to note that while terms like *legalization, deregulation, decriminalization,* and *free marketing* — phrases with different meanings for different folks — are bandied about, there is one term that actually captures the essence of what most seem to be aiming for: *harm reduction.*

In any substance abuse, either licit or illicit, harm can result. There are various causes and effects. One could argue beneficial characteristics in some of these substances (e.g. alcohol, tobacco, heroin, cocaine). Conversely, one could get into a series of arguments respecting the intrinsically harmful ingredients. Whatever the above arguments pro and con, drug-taking creates behaviours which appear to benefit some and harm others. Let me suggest some brief observations surrounding the abuse of drugs, abuses which create harm and have negative consequences.

For the individual user, there are the obvious impacts on his or her health, both physical and mental. The addict, not being an island unto him/herself, but rather a social being, engenders harm in others. The obvious recipients here are family and friends. There are also economic costs related to this harm; they are obvious and are alluded to throughout this document. Harm reduction is a stance – or policy – which has as its goal a decrease in the negative consequences to the individual, his/her family and friends, and society in general. I use the word *decrease* because, from all I have heard and read, it is unrealistic to believe in absolutes respecting solutions, or indeed, cures.

The Provincial Health Officer, assisted by a steering committee, submitted the following conclusions from his analysis:

> *"The steering committee considers this epidemic of drug-related deaths to be an urgent public health problem. The committee believes that an approach of harm reduction is the best strategy for*

reducing the number of deaths. Harm reduction has been studied by several organizations concerned about the negative impact of illicit intravenous drug use on individuals and society.

The following are some definitions of harm reduction from the perspectives of three well known organizations:

1. ***Addiction Research Foundation:*** *harm reduction is a pragmatic, public health-based approach to minimizing the actual harmful consequences of drug use to the individual, the family, the community, and society. The harm reduction perspective recognizes that availability of a great variety of psychoactive drugs is a fact of life in modern societies. A combination of social controls, lodged in personal values and social institutions, help to determine less destructive or more beneficial ways to exercise choice.*

2. ***Melbourne Conference on the Reduction of Drug Related Harm 1992/93:*** *the harm reduction perspective encompasses the range of policies and practises which have as their goal the demonstrable decrease of the adverse personal, social and economic consequences of drug use and the promotion of healthy drug-related behaviours in the individual, the group, the community, and society as a whole.*

3. ***World Health Organization:*** *the purpose of harm reduction framework is to direct attention to the careful scrutiny of all prevention and treatment strategies in terms of their intended and unintended effects on levels of drug-related harm.*

Harm reduction has as its first priority a decrease in the negative consequences of drug use. The principle goal of harm reduction is to reduce the harm to society arising from drug use and distribution. Harm reduction can theoretically be accomplished by reducing the number of illicit intravenous drug users through demand reduction strategies such as abstinence-focused prevention and treatment programs. These programs may be contributing to the decreased number of new illicit intravenous heroin users over the past twenty years. There is a perception that society has become increasingly concerned with the dangers of drug use, thus discouraging first-time users from becoming involved with heroin; whether this concern stems from exposure to demand reduction-focused prevention programs is unclear.

However, there is no evidence that abstinence-centred programs reduce the overall harm related to illicit drugs; demand reduction programs have had little impact on the total number of long-term illicit intravenous drug users. A California study reports the number of heroin addicts in 1985 to be essentially unchanged from 1975.

In short, too much emphasis has been placed on abstinence and not enough emphasis on other strategies that concentrate on reducing the level of harm experienced by users that continue to inject. As a result, the community of illicit intravenous drug users is experiencing disproportionate levels of serious medical problems such as HIV/AIDS, STD's, and Tuberculosis (TB). Furthermore, these individuals are at risk for violent injury and death related to criminal activity associated with illicit drugs, as well as at risk of sudden death from drug overdose.

There is now a need to devote more attention to the problems associated with frequent and high risk intravenous drug use, including drug overdose death; transmission of infectious disease (e.g. HIV/AIDS, Hepatitis B); and violence and crime associated with intravenous drug

use. Several harm reduction strategies are available that do address these problems, including needle/syringe exchange, methadone maintenance, vein care and safe injection counselling, condom distribution, and STD prevention information."

Harm reduction, it can be said, has many faces and forms. It is flexible, pragmatic, and realistic, but not necessarily without the potential for public conflict, philosophically and socially. An obvious concern surrounding needle exchange programs is: "How can the state justify providing needles to individuals who will then inject themselves with a harmful, illicit substance?" The answer: by reducing the greater harms to the individual (sharing needles) and society (health care costs associated with HIV/AIDS). The costs, both human (death) and economic (health care) are astronomical. It was stated during this inquiry that for every dollar spent on prevention, another eleven dollars is saved on the other side of the scale:

"Our society has difficulty with the provision of services to people involved in illegal behaviour. We struggle to balance the need to be involved in harm reduction and the message we give by doing this."

Terms and policies such as zero tolerance and total abstinence that ignore other outcome goals (e.g. improved health, safety, etc.) are unrealistic as basic social policy positions. Tobacco and alcohol attest to that. Drugs are no different. Abstinence is one form of harm reduction; it works for some, but not for others. What ought to be done for those others? Methadone is one form of harm reduction; it works for some, but not for others. The thrust of the methadone program was directed toward society; get the addict back into the work force, and reduce the incidence of crime. It is questionable whether there was ever any intention to improve the individual's health. Nevertheless, methadone has been shown to reduce individual mortality and morbidity. Over and over again, I heard from those on that program that "I can at least function again; it's OK." We have to remember that drug addiction is a continuing physical and psychological disorder which requires continuing medical and social care. Addicts have been seriously damaged, the question is: "How can they and society manage and reduce further damage?"

It has become apparent that addicts, in many cases, require some form of maintenance in order to lead some semblance of a life which reduces the harm to themselves and others. Traffickers in illicit substances do not subscribe to that particular philanthropic position; rather, their motivation is greed and money, at any cost. The broader state interest in harm reduction must not only reject and deal effectively with that stance, it must address the health of the addict with a menu of supportive services and programs. I refer here not only to medical treatment which includes the prescription of drugs, but also counselling, housing and employment.

Whatever is adopted in British Columbia, the thrust ought to be toward changing the harmful behaviour of drug abusers and simultaneously changing the attitudes of the public and professionals as well. I suggest that there must be a greater sense of partnership on the part of all service providers and agencies who deal with this problem on a daily basis. Addicts, filled as they are with fear and distrust of all authorities, must be enticed on board through genuine outreach approaches. If society extends its hand, there is a greater chance the addict will grasp the "window of opportunity" to which so many of them alluded.

I believe that a truly cooperative harm reduction philosophy and approach to the drug problem in this province will be a cornerstone strategy for reducing the mortality of heroin and cocaine users. There has to be some compromise on the part of society. We must acknowledge that, in supporting harm reduction programs, we are only chipping away at the problem; all the while, some harms will remain (hence the term reduction). We will have to take some risks in doing so. This approach ought not to be considered the answer to all of the problems related to drug abuse, but if it puts a dent in criminal activity, in robberies, assaults and murders; if it reduces the ambulance calls and Narcan injections; if it frees up court rooms and jail cells; if it cleans up our streets and public places from harassment and discomfort; and if it brings peace and quiet in families and increased employment and productivity, then the harm to all can be said to have been reduced — not completely, but perhaps to a socially tolerable level.

In summary, the area of harm reduction is crucial and urgency is the watchword. I have concluded that the Ministry of Health should assure core funding for needle exchange facilities and programs, undertake strategic planning for the expansion of needle exchange services to provide 24 hour availability as required and mobile units where necessary. The Ministry of Health should also consider expanding its additional harm reduction services provided from

needle exchanges and street clinics to include health care, disease control instruction and supplies, vein care and safe injection information, as well as street drug testing and analysis. I believe there is a need to conduct several pilot studies on the trial issue of supplying maintenance methadone for appropriate clients from needle exchange and street clinic sites. In addition, I found strong arguments for employing recovering addicts and substance abusers in aide support/outreach roles at these sites. I believe there would be significant value in considering (a) pilot project(s) to train personnel to administer Narcan at these same sites. I must also stress the value and unique non-judgemental stance of the needle exchanges, particularly in their role as communications centres and venues for information-sharing between health care providers and street-involved substance abusers.

I therefore RECOMMEND THAT the Ministry of Health:

9. Consider the creation of facilities/clinics which incorporate needle exchange services, naloxone (Narcan) availability, methadone treatment/ maintenance and counselling/outreach programs;

10. Review funding initiatives to the various needle exchange programs, expanding and dealing strategically on the basis of the actual reasonable needs and costs associated with those programs;

11. Collaborate with and provide greater assistance to municipal Public Health Departments and needle exchanges in providing counselling support and health care services through these facilities;

12. Provide assistance in determining the quality of drugs on the street, and in disseminating that information to both health care professionals and the addict population.

I therefore RECOMMEND THAT the College of Physicians and Surgeons:

13. Participate closely and directly in the establishment and operation of facilities/clinics which provide detox, treatment, recovery, and outreach programs, including needle exchange services, the availability of naloxone (Narcan) and/or other narcotic antagonists, and methadone treatment / maintenance programs;

14. Review the medical, personal, and social feasibility of providing a paramedical heroin maintenance program within the service structures of such facilities/clinics;

15. Establish an advisory board, comprised of a cross-section of professionals and lay people, to conduct research and evaluate various treatment and maintenance models, including methadone, heroin, and abstinence and, through that advisory board, establish innovative, flexible, accessible treatment and counselling options, providing client/patient assessment through one centre, and establishing client profiles and treatment plans with the opportunity to refer them to other treatment programs.

Part C
DETOX

"We have to look at the external and internal motivation for addicts. There is a 'window of opportunity' with them which is critically vital ... we must not blame, but rather understand the cycle: drug abuse equals shame equals drug abuse."

Detoxification is the first step in recovery and usually begins after an individual experi ences the effects of acute intoxication, whether from alcohol or drugs. The terms "sobering up" or "drying out" are most commonly associated with detox facilities, originally carried out in hospitals, police cells, or shelters operated by various religious denominations.

Detox centres came into being to treat public drunkenness more as a social and medical problem than as a legal or criminal matter. Generally this involved little more than a place to sleep off the effects of intoxication. More recently, it has come to include the management of the physical aspects of withdrawal and the motivation of individuals toward treatment.

In British Columbia there are a variety of detox facilities. Two are short-term police facilities, while the majority are separate stand-alone centres. Maple Cottage and Vancouver Detox are operated directly by ADS (Alcohol and Drug Programs, recently changed to Alcohol and Drug Services) while others are operated by non-profit agencies. All receive government funding, primarily through the Ministry of Health. ADS lists the following Detox Centres in the province:

- Vancouver Detox
- Police Detox (Vancouver)
- Salvation Army Harbour Lights (Vancouver)
- Salvation Army Pender (Vancouver)
- Maple Cottage (New Westminster)
- Prince George Detox
- Phoenix Centre (Kamloops)
- Pemberton House (Victoria)
- The Gateway (Victoria)

Each facility is responsible for providing detox services primarily to clients in their designated geographic regions, of which there are five: Lower Mainland, Fraser Valley, Thompson-Okanagan-Kootenays, the North, and Vancouver Island. The two main functions of these facilities are initial detox and motivating the client to seek further treatment. A comprehensive study of detox facilities in the province was completed for the Ministry of Health, Alcohol and Drug Programs, in May of 1993, by Peat Marwick Stevenson & Kellog. This study extensively reviewed the available facilities and analyzed the procedures needed in the detox process as a means of evaluating the efficacy of the existing programs and facilities.

This analysis provided valuable background information for the present inquiry. Many agency personnel, professionals, clients, former clients, and members of the public provided observations on the strengths and weaknesses of detox services and made recommendations to improve services and the continuum of care. Recommendations for strategic planning and improvements are contained in the Peat Marwick Stevenson & Kellog report.

In examining the process of managing alcohol or drug withdrawal, supervision is usually designed to minimize the withdrawal symptoms and craving. The supervision will likely reflect one of three dominant models of detox: social, non-medical, or medical. The social (or informal) model, can be undertaken in any facility or even in the patient's home without medical or nursing staff; no medications are required. The non-medical detox model, which takes place in a free-standing residential facility, appears to be the most popular and efficient method. Primarily non-medical staff manage, with nursing supervision available to administer withdrawal medications if required and a consulting physician present on site or on call. The medical detox model is required in acute medical care settings under the supervision of a physician.

In the first phase of "sobering up," a simple, safe, supportive environment is necessary; this phase typically lasts 12 hours. The second "detox" phase involves the management of the withdrawal syndrome which is related to the active duration of the particular drug. Withdrawal from alcohol and short acting sedatives may develop within six hours of the

last dose; withdrawal for the longer acting drugs may not begin for a week.

The length and severity of withdrawal symptoms depend on a number of factors, including the level and duration of drug use, whether or not the drug was taken in combination with other drugs, and the health and nutritional state of the patient. The severity and duration of withdrawal cannot be accurately predicted:

> *"The more severe the physical dependence, the more severe the withdrawal syndrome is likely to be. Some withdrawal symptoms can be life threatening, especially in patients with other medical conditions."*

The final step of the detox process should be to motivate the patient to seek further treatment as a follow-up and to ensure that there is a continuum of care toward complete recovery. This objective may be reached through proper support delivered by detox staff and well-coordinated case management.

The demand for detox services is high, making analysis of program effectiveness in relation to dollars spent critical. In the first instance, the duration of detox supervision required must be evaluated. Simple "sobering up" in Vancouver's Police Detox or in Victoria's Gateway averages seven to eight hours. The full service detoxes, which provide management of the physical aspects of substance withdrawal, preparation for further treatment, and motivation to continue with rehabilitation, are recognized as requiring an average stay of between five and seven days. It is reported that most clients stay two to eight days in full service detox centres, even though longer stays are common.

I was advised of the many treatment protocols which are designed to meet the varying needs of clients and substance abuse problems. Detox, intensive treatment in a residential setting, supportive recovery, intensive treatment in a non-residential setting, outpatient counselling and day programming mark the major treatment protocols. The belief that all treatment in the detox phase must be in a hospital or medical setting has been revised and other more innovative programs are in the offing.

FIGURE 12: Role of Detox Centres in Continuum of Care

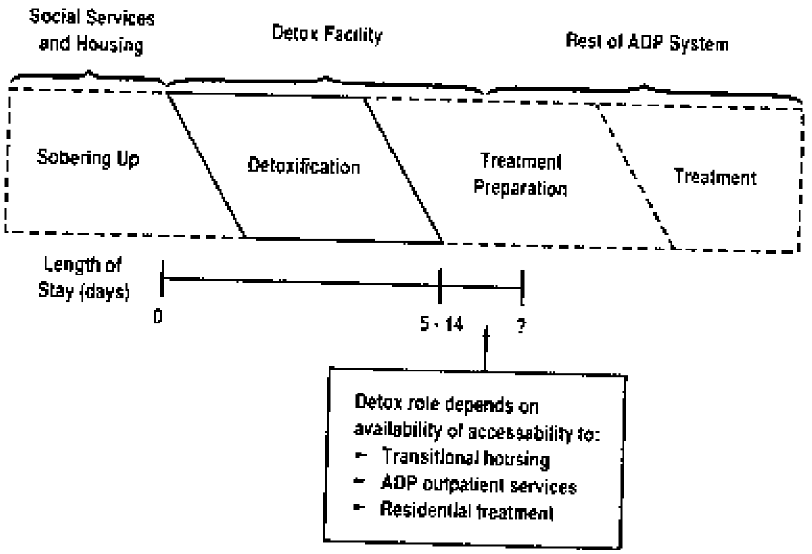

Uncomplicated withdrawal from alcohol requires 5 - 7 days, while withdrawal from long acting drugs and sequential poly-drug usage may require 14+ days to complete detoxification

There is a recognition that different substances require different withdrawal protocols. In examining the Continuum of Care model developed in the Peat Marwick Report (see Figure 12), a sliding time scale is shown to depict the time frame range required in detox for different drugs. It is also important to examine the impact of the trends in substance abuse on detox services. I heard the following during a Burnaby agency meeting:

> *"Only two years ago 15 percent of our patients acknowledged heroin addiction ... now 60-65 percent indicate heroin addiction. We also note heroin use moving to a lower age group in our patient group ... more 20-30 year-olds."*

Indeed, the most commonly abused substance continues to be alcohol. However, despite lower numbers by far, there has been a phenomenal increase in those patients listing heroin as "Client's First Drug of Choice" (229 in 1990-91 compared with 1,914 in 1993-94); an eight-fold increase during a three year period. Of course, a key difference may be a growing willingness to disclose – the one substance is legal (alcohol) the other illegal (heroin).

The most up-to-date data from ADS gives an overview of primary drug type listed on admission (see Table 6).

TABLE 6: BC Ministry of Health Data
Alcohol and Drug Admissions to Detox Services

DRUG TYPE	FISCAL YEAR 1990 - 1991	1991 - 1992	1992 - 1993	1993 - 1994
Alcohol	7517	11381	10220	9522
Cannabis	206	258	238	264
Cocaine	596	1688	1826	1843
Hallucinogens	9	23	15	12
Heroin	229	538	774	1914
Narcotics	213	367	300	276
Other	194	264	280	231

Please note that the numbers reflect only:
a)People admitted to detox services, not the total number of people receiving alcohol and drug services (38,775 in 1993-94).
b) People admitted to ADS-funded services.
c) The client's identified first drug of choice.
d) People seeking treatment: 85 percent of all drug-dependent individuals do not seek any kind of treatment for avariety of reasons.

One referral agency also noted problems with boundaries in gaining access for clients in the Greater Vancouver area. There was concern that if there were beds available at any appropriate facility, then anyone should be able to access that bed, not just a person living in the prescribed area. It was noted that youth detox facilities were apparently reserved for street youth and not for youths who are from homes in the area. However, it was stated officially that there was no intent on a provincial level to limit or deny access to outsiders who are in need.

As will be discussed under the heading *Supportive Recovery*, the object is long-term rehabilitation for those who are motivated; however, care must be taken that detox centres do not become long-term residential care facilities. It would be easy to slip into that role and tie up valuable bed space in much needed detox facilities. It is clear that prolonged stays will diminish the capacity of the centre to accomplish its specialized detox role.

I gathered from my visits to several detox centres that some are more oriented to motivating their patients into further treatment, particularly when that treatment is located on site or close by. In such a case, continuity, as well as some transferability of supportive relationships, can be maintained. For example, at the Phoenix Centre in Kamloops, the Detox Unit is separated from the Residential Supportive Recovery Section by only a few steps. Staff may work in both sections, thus making the transition for the patient much easier. Most importantly, the decision to stay in treatment is much easier when there is a familiarity with the environment, the staff, and other patients.

It can be said generally that some of the inner city detox units had a higher percentage of clients staying fewer days. Some detox facilities tend to focus on clients who will be "sobering up" and detoxing, but do not indicate much interest in following up with treatment. This was said to underscore the concerns about quality of service and the about values and attitudes of caregivers. The "revolving door" is sometimes a result of antiquated attitudes and approaches.

I repeatedly heard of the needs of women, and in particular mothers in acute need of help, with children at home, who went into detox. As soon as they sobered up and were able to function reasonably, they checked out because of concerns about their children who may have been fending for themselves or who were being looked after by friends. Unless long-term treatment facilities or detox facilities recognize the need to accommodate children, mothers in this predicament will continue to

leave these treatment facilities at the earliest possible chance. Many fear, and rightly so, that they may lose their children to Social Services. As one addict indicated:

> *"I went into detox last summer for three days in Vancouver ... worried about my 15 year-old son at home on his own ... I couldn't take it, so I checked out."*

She also felt that the conditions in detox were not very conducive to recovery:

> *"The environment was not good, noise level was high ... fans running ... little sleep, staff were supportive at first, but that changed ... They should have alternative therapy, more support and psych therapy."*

Indeed, Alcohol and Drug Services (ADS) have been responding to some of the issues identified through community groups and previous studies. One example brought to my attention was the *Youth Detox* project. ADS engaged in a community planning process with representatives from the Inter-Ministerial Street Children's Committee, resulting in the implementation of an innovative, non-residential Mobile Youth Detox. This means that trained individuals assist youth in detoxing in an environment that is suitable for the youth; it is not a facility per se, but a more flexible approach to meet the needs of street-involved, addicted youth. So far, by all reports, it has been quite successful. However, there has been much concern expressed about the short supply of money for this developing program.

In touring the province and examining treatment programs, I heard about significant differences in service availability. Some communities had free-standing detox units, others had units in hospitals. Most health care officials, as well as agency and addiction professionals in communities without detox centres, recognized the need for locally established detox units separate and apart from hospitals. Traditionally, hospitals have ended up caring for the cases that come in through their emergency doors when toxic alcohol poisoning or drug overdose is diagnosed. There was a consensus that hospitals are not appropriate for other than the initial emergency treatment of toxic alcohol and drug overdose situations and that longer residential treatment following detox must be provided by facilities other than medical hospitals.

Needless to say, any intervention that offsets addiction and improves quality of life has the potential to save many lives and millions of dollars in future costs. Notwithstanding some of the new innovative and genuine efforts, much more needs to be done to publicize and support these "grass-roots" programs which reflect truly appropriate service to the client that is culturally-sensitive and user-friendly.

I therefore RECOMMEND THAT the Ministry of Health:

16. Examine existing programs within the Alcohol and Drug Services (ADS) and distinguish alcohol detox from drug detox processes, for the information and benefit of both workers and clients;

17. Establish separate facilities for alcohol detox and drug detox;

18. Where feasible, conjoin those detox facilities with treatment centres, providing follow-up health care when those "windows of opportunity" present themselves;

19. Establish and/or support alcohol and drug detox and treatment/recovery facilities strategically throughout the province, beginning with:
    (i) Prince Rupert
    (ii) Prince George
    (iii) Kelowna
    (iv) Nanaimo
    (v) Campbell River;

20. Establish one combined alcohol and drug detox and treatment/recovery centre in the Downtown Eastside area of Vancouver, including counselling and outreach components.

Part D
METHADONE

"Heroin gets in your blood - Methadone gets in your bones."

Methadone is used in many countries, including Canada, for the treatment of opiate abusers. It is a long-acting synthetic opiate agonist with good oral efficacy. Methadone withdrawal treatment by mouth has proven to be effective in suppressing opiate withdrawal following cessation of drug consumption. Methadone is also prescribed daily over months or years to some people with the expectation of reducing illicit opiate use and achieving improved medical and psychosocial functioning. This is termed *Methadone Maintenance Treatment* within the medical community.

Methadone has been available for use in Canada as an analgesic agent since 1948 and for use in the treatment of opiate dependence since 1961. Before 1961, medical treatment opportunities for heroin abusers in Canada were limited because the drug laws prohibited it. However, methadone was used in British Columbia on an experimental basis for short-term withdrawal treatment. This was done through the Narcotic Addiction Research Foundation (NARF) of British Columbia. In 1961, a Methadone Maintenance Program (MMP) was instituted by the NARF. The *Narcotic Control Act* (1961) first permitted this, if in the discretion of the physician it was considered to be "good medical practise."

From early 1968 to the early seventies, there was an appreciable increase in both the number of opiate dependent people in Canada and those receiving methadone treatment. It also became evident during that period that there was a major problem involving the misuse and abuse of methadone in the country. Consequently, the federal Health Department prepared guiding principles for physicians, and the *Narcotic Control Act* was amended in 1972 to ensure greater control over the availability and use of methadone. The effects of the legislation over the next ten years were a reduction in illicit distribution of legal methadone and a decrease in the number of people in the methadone treatment programs.

In 1978 the British Columbia Alcohol and Drug Commission (ADC) instituted a compulsory heroin treatment program. It was abandoned the following year (1979-80) as a result of a court challenge. Concurrently, the drug programs were amalgamated with the provincial alcohol programs. Three years later (1983), the ADC was disbanded and Alcohol and Drug Programs (now Alcohol and Drug Services) was brought into the Ministry of Health. In 1985 a Joint Advisory Committee on the Treatment Uses of Methadone effectively ended the private prescribing of methadone in favour of the government clinic system. The Supreme Court then struck down an injunction against a private physician prescriber, reinstating the status quo of methadone prescribers.

As a result of that decision, the Ministry of Health closed the newly expanded government MMP's in 1987 and the patients were transferred to private physician prescribers. The following year (1988), private clinics were set up for methadone distribution. A few private physicians also continued to prescribe methadone for addict patients. Finally, in 1990, the BC College of Physicians and Surgeons established a Committee on Opioid Dependency to ensure quality of service to methadone patients.

As can be seen from this brief historical review, controversy has surrounded the use of methadone since its introduction in the treatment of narcotic dependency. Great care must be taken in the management of this drug; it has highly addictive properties, with the ever present potential for sale on the black market. In 1985, there were three deaths in Vancouver resulting from overdoses of methadone. In each case, the deceased had been using other drugs as well as methadone. Each had also been multiple doctoring. Coroners' juries made recommendations respecting prescribing, dispensing, assessing, and follow-up. These issues remain today. In summary, the question is how to improve the coordination and control of methadone services in the private and public sectors.

Repeatedly I heard of perceived difficulties with the administration of the MMP under the federal jurisdiction of Ottawa. It was described as "cumbersome

and time consuming," with complaints of urinalysis having to go through federal labs, frequent bureaucratic delays, and a lack of responsiveness to local concerns. Given that more than half of the methadone patients for all of Canada are here in British Columbia, it makes a strong argument for having the provincial government assume this jurisdiction. Furthermore, when we consider the extent of the problem in British Columbia, it makes sense to have more direct control and input into the distribution of federal funds in proportion to the problem.

Figure 13 gives some idea of the national picture, in particular where British Columbia figures in the distribution of methadone.

FIGURE 13: Provincial Methadone Status 1992 - 1993

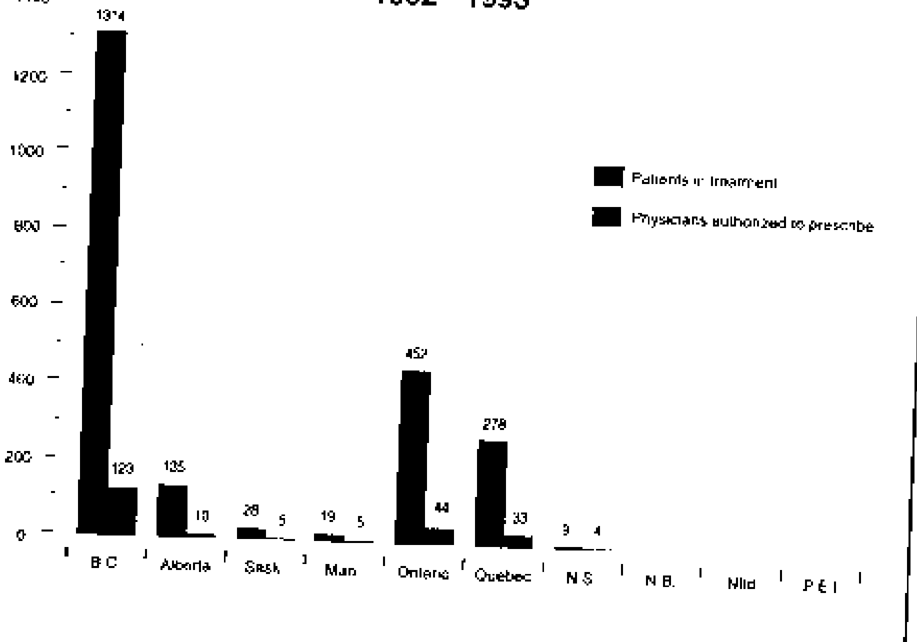

Data from the Bureau of Dangerous Drugs Directorate, Health Canada

Obviously these figures show our province in the forefront on this matter.

What do the addicts have to say about the MMP's? On the positive side, some indicated that it is a worthwhile program. It allowed them to function reasonably well; they could hold down a job and get on with their lives. Some others indicated they really didn't want to stop using drugs; they just wanted an easier way:

> *"Methadone has a real appeal for those with low motivation. For many IV drug users, the 'ups and downs' and the joy of using the needle is part of the picture."*

On the negative side, many addicts stated methadone was a worse addiction than heroin. They feared getting sick. The withdrawal symptoms were more severe and prolonged:

> *"Heroin gets in your blood. Methadone gets in your bones."*

Addicts frequently referred to the program as being too restrictive and, of course, it is. Restrictions were designed to ensure access was controlled as closely as possible so as to prevent new addicts from being inadvertently attracted to a cheap and easy supply of drugs. There is a delicate balance required in this. If access is too restrictive, the addict is turned off and returns to street use. Methadone does not produce the euphoric high associated with heroin either, but it does reduce the need to worry about getting the next "fix," or as one put it: "grinding to get your next fix." Another indicated that it lessened the worry about the possible fallout of criminal involvement.

A case that speaks to the MMP is that of a Vancouver single mother and university graduate. After using various drugs other than heroin for years, she became addicted to heroin while working long hours and raising her son. She related how she has since been on welfare and marginally employed, having lost all her assets to drugs over those years. Last October she was able to get on a MMP after depression brought her to the point of considering suicide. She reports that the program has helped, even though she feels the dosage is not sufficient for her needs:

> *"I could never get quite enough ... just enough to keep you on the edge ... I get diarrhoea and feel my nerves are on the surface of my skin."*

She noted that the only time she feels she can function in a normal life is when she takes more than her prescribed dose of methadone. When she runs out, she is sick for the few days until she can get back into the clinic.

Fearful of talking to her personal doctor, she felt there should be greater flexibility in the MMP, and that she should have more control over her own dosage. She feels that she is normally very productive when she has enough drugs. The rest of the time she is not functioning as well. At least, that is her perception. She concludes that methadone or heroin is the only answer for her.

Generally, it was agreed that each addict must be recognized and treated as distinctly individual. Methadone provides a therapeutic window for those particular addicts who exhibit an indication for the program. Medical experts I spoke with were careful to explain that methadone works best when individuals are properly assessed. Those who have a particular drug of choice (e.g. heroin) may be helped through this. Those who are polydrug users, and that may well be the majority, do not fit into the MMP profile. It would not be an appropriate treatment; there are downsides. Assessments, therefore, ranked high on the priority list of the medical experts:

> *"One should not be hooked on this drug by the system. That is why assessment is so important."*

The MMP was also cited as helpful in identifying other medical problems suffered by addicts. It was seen by not only health professionals, but also others in contact with addicts, as a "window of opportunity" to attend to the general health of the individual and his/her family. Additionally, it allowed for personal contact and was seen as an opportunity to motivate and assist them in other constructive ways. In short, it involves a holistic philosophy that sees "the big picture."

I learned that in some cities methadone is being used in new and creative ways. Needle exchanges in New York are dispensing methadone on the spot. The intent is to reduce the need for street heroin and, consequently, the prevalence of criminal activities. The risk of HIV transmission is also reduced in the process. This is a technique in harm reduction which could be an effective first step in substituting a legally-prescribed drug for the illicit, uncontrolled street drug. The discovery of the stabilizing quality of methadone would be attractive to some addicts who are tiring of the chase, the scene, and worrying about ill health. It may work for some. I repeat, one dollar spent in harm reduction and treatment saves eleven dollars in the long run. MMP's have been shown to reduce the incidence of needle sharing and the frequency of injection. It was said that there is also a decrease in drug-related crime amongst individuals enrolled in MMP's. Participants in methadone treatment also appear to experience lower mortality rates than do their untreated counterparts.

I met with the Special Committee of the College of Physicians and Surgeons, a committee which reviews and monitors these MMP's and the physicians who prescribe methadone in the province. One of the members had just returned from an international conference on drug treatment. He stated that methadone is now used worldwide as a treatment module. The international consensus was that the MMP is a positive force in harm reduction. This is particularly the case in light of, as he put it, the "unholy trio of AIDS, IV drug use, and tuberculosis."

When the committee was asked about the possibility of introducing heroin or morphine in addition to, or in place of, methadone, the simple answer was that these drugs do not fit into the medical treatment model. It would not be considered an appropriate medical intervention. The committee went on to say that heroin provides an immediate euphoric high, which is short lived, and would require more frequent attendance at a clinic or dispensing centre, perhaps up to three times a day more. It was argued that this routine precludes normal living or work activities for the addict, who would instead be forced to focus on the appointment schedule. Conversely, the street workers and addicts reported that "there are other segments of the medical profession in Canada, the U.S., and Europe, which have concluded that providing heroin is a completely appropriate medical intervention and that heroin, in fact, provides a relatively long-lived high, certainly when compared to cocaine." Indeed, Switzerland recently launched a pilot project in which heroin may be prescribed for treating heroin addicts. The Swiss experiment will provide 700 addicts in eight participating cities with medical access to heroin, morphine, injectable methadone and, under some conditions, cocaine. In each city, the program offers lodging, employment assistance, treatment for disease and psychological problems, clean syringes and counselling. The aim is to reduce social harms related to addictions. To join the experiment, the addicts must be at least 20 years old, must be residents of the program city, and must have at least a two-year history of opiate dependence or must have suffered demonstrable health or social problems as a result of opiate dependence. In

addition, other forms of treatment must have failed or have been deemed unsuitable for the participants.

The Special Committee of the College of Physicians and Surgeons argued that heroin "carries" would not and could not be rationalized. By analogy, illicit traffic in "carry methadone" remains a problem. The committee concluded that this has to be regulated through lower doses and more frequent urinalysis to check for illicit drug involvement. Again, a completely different philosophy was expressed at the street level:

> *"The (highly regulated and controlled) model assumes addicts are not competent to inject their own heroin and that they cannot be responsible to ration their own drug dosage ... they can and should be responsible for this."*

In support of this position, one methadone-prescribing physician we contacted had this to say about monitoring and reducing dosages:

> *"The federal law requires supervised urine testing twice a month ... this is a significant expense. I've got a lot of patients, three quarters of them at least, who have never had a dirty sample of urine. What is the point of having 100 clean urine specimens at $54.00 each sitting on a person's chart? And what happens if the guy does blow it and has a dirty sample? You aren't going to throw him out for one dirty sample, are you? So, I'm only doing them about once a month on most of them and, for the ones I'm really worried about, I do lots."*

This doctor described his success at letting the addict decide if and when to decrease the dosage. In other words, he administers a client-directed withdrawal. He further described his success as follows:

> *"I use a really neat way of handling this and I'm really pleased with it ... a technique referred to as 'self-directed withdrawal' ... I say, 'OK you are on this much. You tell me when you're ready to cut it down ... if you're having trouble we'll put it up again'. And if I trust them and they trust me, we do really well with it."*

> *"I've got one that just completed in November. She is so happy; she had a long criminal record and her life is going really well for her now. She's got her child back with her and she has her job. She is living a totally different life. She isn't living with addicts any longer and she doesn't want to have the methadone anymore. She had no adult life before and now she is really enjoying it. If she slips and gets back on heroin she can come back in and she knows that, but she self-directed her own withdrawal. She sat on 4mgs for weeks because she was afraid to go. Then she started to realize that she had forgotten to take it a few times and then she realized that maybe she didn't need it after all ... It's very rewarding to see this happen but it's only a small proportion of people who will go through withdrawal."*

The committee advised me that of those screened for the MMP there was an overall recovery rate of 65 percent. This was taken to mean that, of those on the program, 65 percent avoided illicit drugs, opting rather for this maintenance method. On the other hand, I heard from others that the abstinence model had a 10 percent recovery rate. That is, only ten percent who quit "cold turkey" and without chemical assistance avoided returning to the illicit market. Countering that was a figure provided by the Alcohol and Drug Services which refuted the ten percent. It indicated an abstinence model (Aurora House) showed 72 percent still clean and sober after one year. Even at that, it was suggested that 72 percent was thought to be low. The differences raised here indicate that further research and follow-ups are essential if there is to be any semblance of statistical accuracy for future program evaluations.

The College of Physicians and Surgeons committee concluded to me:

> *"Since the causation is multifactorial and treatment success varies with client profile and stage of addiction, well meaning but simplistic solutions such as the 'War on Drugs' are doomed to fail."*

The College concludes that an effective program must be comprehensive, cost effective, and capable of falling into the current health care system. This must be "based on established techniques."

Part D
METHADONE

I therefore RECOMMEND THAT the Ministry of Health:

21. Research and consider the feasibility of transferring responsibility for the methadone dispensing program in British Columbia from the federal Bureau of Dangerous Drugs to the provincial Ministry of Health.

Part E
SUPPORTIVE RECOVERY

"You not only have to change your playmates; you also have to change your play pen."

Executives from Alcohol and Drug Services (ADS) described a complex array of treat ment services, various levels of client intervention, and variations in staffing and staff training in the program settings. The concept of *Continuum of Care* within an overall objective of treatment for individuals with individual needs was promoted as an ideal. A recent *Review of Supportive Recovery Homes* by a management consulting firm provided ADS with some ways and means to evaluate and assist in providing more effective and efficient treatment for substance abuse.

The concept of *Continuum of Care* in the alcohol and drug field is complex. It is described more as a process than a place or physical setting. A graphic illustration of the treatment process and the settings in which these processes occur is reproduced in Figure 14. The process moves from acute intervention through rehabilitation to maintenance, intersecting with various treatment settings where, depending on their needs and the stage of treatment, individuals are placed.

FIGURE 14: Treatment Process

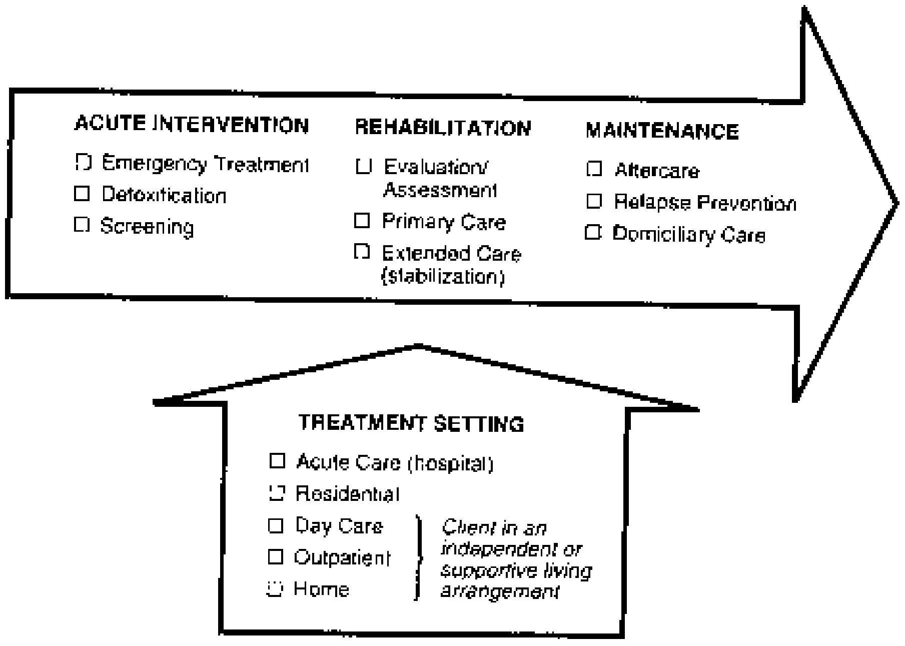

Typically acute intervention is required in the first instance when emergency treatment is required (i.e. physical, social, or psychological intervention). Detox follows for the management of the acute alcohol and/or drug intoxication, which is in turn followed by withdrawal. Rehabilitation is the next

phase, commencing with evaluation and continuing through primary care to extended care. Finally, the plateau of maintenance is reached, characterized by aftercare, relapse prevention and, where appropriate, domestic care.

Study indicates that self-help organizations, halfway houses, detox centres and 12 step programs were founded by recovering alcoholics in the fifties and sixties, mainly in the U.S. Supportive recovery homes generally "have their roots in the social model that is based on Alcoholics Anonymous principles." Supportive recovery homes might otherwise be described as intermediate care settings. They provide transitional living arrangements for those who are moving from hospital in-patient care to independent living. A common feature of the supportive recovery home is the provision of alcohol and drug-free communal living in a supportive environment staffed by nonprofessionals.

From our discussions with recovering and currently addicted individuals, it is clear that, following detox, the recovering client is particularly vulnerable to slipping back into the addiction cycle. In fact, statistics show that a significant number of clients do not remain in detox programs for the duration or desired length of the programs. Many return to the streets or their usual environment, often to the same social situation or pressures that placed them at risk to abuse substances in the first place.

These individuals are likely to become reinvolved and often become candidates for readmission to detox facilities. The fact that there are rules established in most detox centres precluding an individual from more than four admissions per year is a strong indicator of the "revolving door syndrome." It also underlines the fact that, on discharge (planned or simple walk-aways), these patients are in need of further treatment. They may or may not be ready for the structure and commitment of the supportive recovery home.

For those who do complete detox and are serious and motivated to "stay clean," there has to be some form of follow-up care if there is to be any hope of success. Certainly, supportive recovery centres provide a much better transition for the client than a simple return to his or her original environment. One recovering addict perhaps best described this necessary step:

> *"You not only have to change your playmates; you also have to change your play pen."*

This gives recognition to the fact that a simple return to the old environment, which contributed to the process of substance abuse in the first place, will not likely be productive even if the individual returns with an initial resolve to abstain and control his/her life. In my travels I was most impressed with some of the recovery homes that are operating in the province. Some of these receive funding from one, two, or perhaps three different Ministries. There are also those which do not have official funding from the primary ministries, but which operate on the social welfare payments of the recipient clients, who voluntarily reside there and benefit from the supportive environment of the home. In effect, these clients pay for their own treatment, having only a meagre comfort allowance of $60.00 a month for any and all personal expenses. Room and board is supplied. For those who are smokers this is almost a punitive situation, but it speaks loudly of their motivation and willingness to help themselves by financing their own recovery. One such establishment which is managing on its own is the Last Door Recovery Home in New Westminster. There are others, struggling for survival, courageously providing important assistance in the long term. Quality Recovery is another facility in Surrey which is on the fringe for funding. The residents pool their welfare assistance in order to stay in treatment. These individuals must be motivated to accept these conditions and have only token spending money.

Operating successfully for almost ten years, the Last Door has an impressive record, remains full, and is well regarded by neighbours, clients and ex-clients. In fact, testimonials to their success were heard around the province. At one institution, a former resident told of how he had managed to "clean up" and get on his feet through their program. He indicated that he had branched out from the "Last Door" to open his own recovery centre. All went well for a certain period, before the pressure of success and stress saw him gradually get reinvolved in drugs. He fell back into the cycle, back into the penitentiary, but, as he said, he had tried, made his best progress so far, failed and would try again.

One of the valuable techniques that the Last Door appears to employ is the enlistment of recovering addicts who have been part of the supportive group setting for a number of months. These individuals are assessed for motivation by a street-wise and drug-wise husband and wife team and are gradually given more responsibility in the administrative running of the home and in the group processes. Thus, the centre draws from its own recovering clientele a few key individuals who show promise in

terms of helping others recover. The director and staff at this recovery house saw the key to success in passing the reins to the undergraduates, whose self-esteem and pride had grown with their own success. It was strongly stressed that individuals in recovery are a most important resource in the process of others recovering. We have seen this in the strength of both the AA and the NA movements. There is no doubt that recognizing the effort of the recovering individual is an empowering tool which reinforces recovery and consolidates success.

Representative executives from some of the officially recognized and funded supportive recovery homes in the Lower Mainland and Fraser Valley attended in-camera sessions for agencies during the course of this inquiry. Several issues became clear to me. The need for coordinated core funding for supportive recovery homes seems essential. Many operators indicated that they spent an inordinate amount of time chasing funding from a variety of government ministries who may purchase one or more beds in a given centre:

> *"We spend more time seeking the funds than we do helping the people ... We need a guarantee of funding to get on with the job."*

Another factor that became clear to me was the fact that long-term addicts or substance abusers are not likely to benefit to any substantive or sustainable degree from short-term care. I heard the term "spin dry" in reference to the short-term care generally offered and paid for by the Ministry of Health. Perhaps one of the operators said it best:

> *"A guy who has been on the street for 20 years cannot be helped in 28 days. Short-term is not successful. The attraction of the big city is still there for them; they need to be removed from that. Long-term hard-core addicts need long-term treatment."*

Success was achieved with some clients who had been in one program for several months. Women were able to stay up to ten weeks and recently the program was modified to allow mothers to bring children with them. Men were staying 60 days, with some facilities allowing longer stays.

It only makes sense that habit patterns that have taken years — and often decades — to build up are not going to be easily eradicated with a short stay in a recovery home. New replacement attitudes and activities must be built into a repertoire of responses to stress, to family, to work, and to social networks, many or all of which are generally dysfunctional. In terms of financial efficacy, there are many good arguments for adopting the long-term supportive recovery route. The first, of course, is that the chances are better that the addiction process/mechanism may "stay fixed." Obviously the short-term processes are not as effective. This is demonstrated by the "revolving door" of the detox units, and addict/alcoholic accounts of the many times they have passed through detox and recovery, only to return to using drugs or alcohol later on. It was also pointed out that the short-term intensive care program put on in clinics often costs the same amount as a two or three month stay in a supportive recovery home.

The testimonials throughout are anecdotal but fairly clear. Those who have "made the break" usually give the credit to a long-term program that took them out of their environment. They were able to set up a new network of social contracts and new ways of coping with life:

> *"Most of our successes are due to long-term care ... they would not have remained clean without a complete break for a long term."*

In summary, the most important step following detox for those who are motivated is to have available supportive recovery programs. It is clear that, to be effective, these programs must be of sufficient duration to help the recovering substance abuser make the transition to abstinence. The need for residential care in this process is essential and of course, the funding for these facilities and programs must be secure or guaranteed. Many times it was clear that there were simply not enough of these residences. Some proprietors indicated that they had sought funding from various ministries but had never been visited nor assessed and thus had no official recognition. Staffing is always a problem, given low wage rates and little or no job security. Relying on motivated, recovered substance abusers is almost the rule or norm. In turn, clients in residence prove their motivation by trading their welfare cheques for the opportunity to stay and take part in treatment.

I believe that jointly, Social Services administration together with Ministry of Health personnel should take steps to expand their assessment capabilities

with a view to bringing more supportive recovery facilities on stream. The small self-contained home appears to be the best setting and usually fits into the community quite well. It is also conducive to normalizing community experience for those recovering. Here again, there is a role for the recovering substance abuser to assist and, in turn, gain self-esteem as has been demonstrated through the AA and NA programs over many decades.

I therefore RECOMMEND THAT the Ministry of Health:

22. Provide more substantial strategic funding for existing supportive recovery programs which are functioning well;

23. Coordinate and expand the supportive recovery programs, provide longer term recovery, ensure staffing levels are adequate, and optimize small, community-based, home-type environments;

24. Seriously examine and consider the deployment of properly-assessed, recovering addicts to act in a consultant/aide capacity to the staff of detox, treatment, supportive recovery, and needle exchange programs.

Chapter 4

SOCIAL ISSUES

Part A
SOCIAL SERVICES

"We need to work together more."

How many times have alcohol and drug counsellors, mental health workers, and social workers generally echoed that comment? How many times did I hear, "We don't have a lot of experience dealing with this drug ... we need more education on how to handle this drug." They were referring more specifically to heroin, but also generally to cocaine and other substances which had indirect, deleterious effects on those social workers.

It is appropriate to comment, in this part, on an aspect of the problem which gave rise to this task force. I refer to media prominence given overdose deaths immediately following the distribution of welfare cheques through the Ministry of Social Services on what is called "Welfare Wednesday."

On the second last Wednesday of each month, welfare recipients receive cheques from the ministry offices around the province. Distribution methods vary from mail delivery to more direct delivery or personal pick-up. The intent, of course, is that the money would be used for the basic necessities: food, shelter, and clothing. Other various needs are provided for, dependent on personal and family circumstances. The issue of public concern was that, for some of those misusing drugs, these monies were being diverted toward the purchase of illicit narcotics and that, in some instances, those narcotics resulted in the deaths of those individuals. That is true.

The Coroners Service statistics, by day of the week, point out that the incidence of death increased dramatically near the end of the month and specifically on the second to last Wednesday, Thursday, and Friday of the month. Table 7 illustrates the breakdown of deaths by day of the week.

TABLE 7: Illicit Drug Deaths for 1993 by Day of the Week and Week of the Month

FOR BRITISH COLUMBIA:

WEEK	Monday	Tuesday	Wednesday	Thursday	Friday	Saturday	Sunday
1st	7	13	6	10	11	10	14
2nd	6	9	10	9	8	6	10
3rd	9	4	6	10	12	14	9
4th	7	8	22	26	17	11	4
5th	5	4	1	5	7	14	7

There appeared to be no argument or confusion about these incidents and their relationship to welfare cheques amongst street workers or addicts. It was true. For some addicts, that's where the money was going. Indeed, a case was related to me where an addict received his cheque and, down the alley from the welfare office, signed it over to his supplier.

That certainly didn't surprise anyone living in the area. Cash may be more convenient, but traffickers

will take guaranteed cheques. It didn't surprise me at the end of the day, either. The chapter on the addict and addiction says it all. The addiction is very powerful when it comes to making choices and being irresponsible. As one recovering addict said at a public meeting, responding to the question. "And what about food?": "Food?! What's that?!"

When I posed this scenario to a social worker, the matter of fact answer was: "We just hand out the cheques." It was not a facetious response, not cavalier, it was simply the truth, the way it is. The public employee could not be concerned with what happened after he/she had fulfilled the required task. It was the recipient's responsibility to ensure the funds provided were used properly. Indeed they were used properly — in the mind of the addict.

While overdose deaths were a primary topic, there was also a wide range of observations regarding welfare generally. Let me present some that were made over the course of the private and public hearings:

> *"Role models are important. We are what we are taught ... if I know only welfare, lying around doing alcohol and drugs, then where am I going?"*

> *"There is an underground network of information flow but nobody wants to write it down, because of the consequences ... there is a problem with independent living. Why do we have to worry about a kid on welfare? ... why do we have to ask the kid's permission to find out if he's attending school? ... I don't mind sharing information about anyone, I'll tell them and deny it later if that's what it takes to keep someone safe."*

> *"The solution lies in stable housing, independent living. This would make a big difference in their lives, as long as it had a real tolerance range for behaviours. These people have an incredible sense of isolation with most of their welfare cheques going to housing."*

> *"When you are thinking about the economic situation, they are the poorest of the poor ... look at the necessities ... it's a lot cheaper to stay high ... the pain isn't there. These people are this poor, there are not enough shelters for these people ... we need workers out there who can relate to these people."*

> *"I have a methadone license and recently one of our clients was refused funding by Social Services ... cited that it was a medical problem, not a social problem. There's not really enough money to go around for any of these problems."*

> *"Services don't exist for some women because they have a problematic relationship with Social Services. Social Services are not responding to intravenous drug user needs ... if we push the issues around providing services, I have been threatened with job discipline."*

> *"It is common knowledge that most addicts are addicted to welfare as well as drugs. Their welfare cheques are used to purchase narcotics for use and resale and almost always leave the addict penniless, hungry, and often without shelter."*

The typical Social Services drug-involved client tends to be a single female parent. Her companions, male and female, are more often than not entrenched in the drug subculture. She receives income assistance and often requests crisis grants to replace lost or stolen cash. She sometimes "loses" prescription drugs, is hounded by unpaid utility bill collectors, and is noticeable to her neighbours and extended family. Occasionally, and with growing frequency, she is involved in robberies and other crimes, both petty and major. A Social Services supervisor had this to say to me:

> *"Our first contact is normally in response to a report that this woman is involved in heroin or cocaine use with resultant neglect of her children. The Child Protection Social Worker who investigates this report is unique in that he/she is often the first government agent who attempts to engage her in addressing the problem. The Social Worker is often the first notification that this drug-taking behaviour is suspected and will be*

censured by the community. In short, the Social Worker wags a warning finger, identifying risks to the children and perhaps veiled threats of mandated action in the future.

"Almost always, the client is an expert at excuses, directing blame elsewhere, and so full of denial that she never accepts the critical comment or the offer of help. After all, the Social Worker is requesting that she face up to a life of attempting to meet her awesome responsibilities of single parenting at the poverty level. The Social Worker is also attempting to get her to turn her back on the day-to-day support network of friends and replace it with anonymous and distant, paid helpers.

*"Services offered are referral**[s to various programs such as,] *TO ADAPT, daycare while attending counselling or Narcotics Anonymous support groups, homemaker service to support her getting organized and physically cleaning-up her environment, or even Short Term Care Agreements to provide for the children in a resource while she enters a detox or residential program.*

"These services are seldom accepted until the effects of her addiction are blatantly threatening the safety of her children. Then, the typical report is that she went out and didn't come home, or that she has overdosed and the RCMP are taking her to the hospital and who will look after the children? These crises often enable some services to be accepted by the client. Most often, however, we are allowed only short-term participation in a helping role and the client reverts to old behaviours.

"With the exception of apprehension of children, all services are voluntary and only effective when the client is ready to receive them. What brings this readiness about is a mystery, specific perhaps to the individuals social network, a serious scare, arrest, or a falling out with the drug culture. Seldom can our intervention strategies take any credit.

"Early identification of these people is possible if it weren't for the ethical consideration of confidentiality. They are known to the police as associates of arrested drug traders, to FAW's as the people who request extra income assistance for one crisis or another, to emergency ward staff, to the needle exchange program, and to doctors and pharmacists, who easily recognize the profile. Even my agency doesn't consider it has a mandate to investigate simple drug use, unless it is identified that it is resulting in obvious child neglect. Perhaps we should assume that it is always a child protection concern.

"I can only guess that the client profile described above is repeated, more or less, many times in the province. The incidence of reports relating to these situations has increased dramatically in the last few years and our caseloads of children-in-care include children of drug-dependent parents, for short terms during treatment, or as long-term wards of the province.

"The services available to these families are numerous. They include the direct support of a ministry Social Worker and a variety of services to enable access to treatment programs, such as daycare, homemakers, child-in-care services, family therapists, transportation to medical help, and child care workers. As well, for child protection reasons, individualized and creative service plans can be implemented and funded. Aside from drug-dependent parents we also have a mandate for services to youth. A Reconnect program attempts to provide support and referral to drug-involved youth."

This profile was provided within the context of the ministry child protection mandate. It points out some -- just some -- of the many frustrating,

*[editor's notations]

personal difficulties faced by the single mother, the offspring, the social workers, and the community. It oftimes is nothing short of a lose - lose situation for all. It touches on poverty - food, shelter and clothing — the basics in society. It does not mention education for the children, or the difficulties connected with that part of living and coping. Serious substance abuse has devastating effects not only on the user, but on those for whom the abuser ought normally to be held responsible.

In the cities I visited these types of situations were commonplace. One mother of four children pleaded for help to kick her addiction to heroin. She was married to, and living with, an addict. She portrayed a genuine concern for her children, but she was unwilling to leave them to attend a detox centre and treatment program. The nearest was many miles away. Conversely, all she could do at home was inject heroin and lay on the couch all day, impervious and unable to attend to the needs of her children. Her eldest daughter looked after them; she was 16 years of age. What is to happen to those children? What does the future hold for them? Is it any wonder I came across third and fourth generation welfare recipients who were addicted, had no life skills to speak of, and were unable to cope with the simple day-to-day tasks we take for granted?

Social Services workers for the most part, I was told, provide temporary services to clients. Those workers see many in pain because of the various forms of abuse, poverty and physical and mental difficulties. Clients are on occasion physically and verbally abusive, to the point of kicking in office doors and breaking windows. This is definitely difficult for workers and also difficult for other clients in these office situations. The workers indicated the need for more sexual abuse counselling. Mental health counselling has a long waiting list. Housing is not adequate; landlords are the beneficiaries when welfare benefits are increased.

I understand that some hotels in Vancouver's Downtown Eastside, and particularly those in the Main and Hastings area, offer discounted beer (beer for $1) on "Welfare Wednesday." This practise is of major concern in light of reports that the police cite drug use, drug dealing, and serving of minors and intoxicated people as major concerns with some of the beer parlours and pubs in the area. Combined with this is the fact that many of the deaths in the Downtown Eastside area have involved alcohol abuse combined with illicit drug abuse.

This report also mentions in subsequent chapters that people are concerned about the substantial numbers of licensed seats in this very concentrated area. What then can we expect from the people who live here and suffer from addictions? Free enterprise and profitability should not override our collective social responsibility to improve the environment for people living in this community. Indeed, it is a sad state of affairs when we knowingly promote alcohol in such a disadvantaged area.

One might properly ask: "What have all these Social Services matters got to do with illicit substance abuse?" They are, in fact, integral to that issue if we concede that a holistic, as opposed to a piecemeal, approach is to be adopted in seeking solutions to addiction, recovery for the addict, and a diversion for those at risk.

It must also be remembered that it was "Welfare Wednesday" and the number of deaths in the ensuing days which pressed this task force into being. Over and over again, I heard of and witnessed events which pointed out the distinctions between cause and effect, roots and symptoms; between the addiction and the addict. I heard the plea to address the multiplicity of social problems: employment and vocational services, illiteracy and special education, expansion of street services and outreach programs. Housing is needed: some reasonable standard of cleanliness and decency, not an eight by ten hovel with a cot and a hot plate costing, incidentally, $300-$400 per month.

Housing is an example of one fundamental human need: shelter. If the general public were to witness and experience the hovels some addicts exist in, they would perhaps better understand why some of these people need alcohol and drugs to face the world day to day. One addict described how the heroin masked that reality when he said, "After shooting up, I could live under the Hastings Street viaduct and actually believe I was living in a castle." The narcotic provides temporary relief from an environment of social, physical and psychological pain.

It is difficult to discuss specific examples, particularly when one party is critical of another and the other is not available to state its case. This occurred frequently concerning Social Services. One example comes from a northern community, where the observation was made:

> *"Most kids on the street (as young as eight) will not go into group homes. When they are with parents who are not*

parenting well, these kids experience considerable violence at home. Young girls in that position don't want to go home. Social Services wants to send them home, but they are not safe there. They could qualify for "independent living," but that is denied. The girls are invariably drawn into prostitution. Then they are intravenous users; the circle begins. They have incredibly low self-esteem. They needed a safe place to live - without a lot of strings attached."

I cite this as an example not only of housing or shelter, but also of the interrelated difficulties surrounding youth, parenting, abuse, prostitution, intravenous (IV), and health care matters. One worker, in frustration, said pointedly, "Money could be diverted into services ... fewer bureaucrats and more services."

One domestic violence counsellor indicated that 95 percent of her clients seek help because of substance abuse problems. 60 percent of them have been sexually abused and 70 percent of the women who have been sexually abused also have substance abuse problems. None of this information, like women living in poverty because their partners spend money on drugs is new to social workers. It is crucial that consideration be given to not only the primary abuse, but also the secondary victims, usually battered women and children. Alcohol and drugs contribute to family violence and violence in general. Violence is painful, physically and psychologically, and so, to counter that pain, the victims turn to illicit and licit substances.

"The need to work together" could not be a better theme for Social Services. If truly there is a ministry in government that assists and improves the lives of so many of our disadvantaged and disenfranchised, it is Social Services. Unfortunately, the needs are great and the general public's financial ability to address these needs is constrained, but, despite this dilemma and the social workers' overwhelming case loads, they continue to assist, counsel, and refer clients to a limited number of resources. From what I can determine, these workers have one foot in the boat and one on the dock, and are trying to pull the two together. From the top, the system presses for restraint and ever-expanding caseloads; on the bottom, the client experiences increasing distress as a result of few life alternatives.

The concerns that have been highlighted in this chapter reflect issues ranging from improved housing and employment training and support to education for social workers and improved distribution of welfare funds. Implicitly stated within the concerns is the fact that the issues span multiple ministerial mandates. Indeed, some of the social engineering required to respond to these issues may well be serviced under the Social Services umbrella.

I therefore RECOMMEND THAT the Ministry of Social Services:

25. Develop and implement an educational program for ministry employees who work in the fields of social assistance and substance abuse for a greater understanding of the addict and addiction;

26. Develop and establish educational programs in the areas of parenting, life skills, and coping, for the various clients (parents, guardians, and youth) within the welfare networks of the ministry;

27. Consider staggering social assistance payments throughout the month, rather than concentrating them near the end of the month;

28. Consider electronic rent payments to welfare landlords;

29. Conduct a review of existing premises to ensure that reasonable accommodation standards are provided by the welfare landlords and that increases are justifiable and warranted.

I therefore RECOMMEND THAT the Ministry of Attorney General:

30. Review the legality and propriety of "overserving" and offering discount beer or alcohol in beer parlours and pubs on or near "Welfare Wednesday," throughout the province, but particularly in the Downtown Eastside area of Vancouver;

31. Reduce the number of available seats in beer parlours, particularly those in the Downtown Eastside area of Vancouver.

Part B
POVERTY/UNEMPLOYMENT

"In our society, we equate work with value, and that equals self-esteem. It is the value that an individual gives back to the community."

I would submit that long-term unemployment and dead-end low paying jobs contribute to the rate of substance abuse in our province. Although British Columbia is better off than some provinces, we still have a high rate of unemployment, especially with our youth. In Great Britain, where rising unemployment and poverty are endemic, studies of the epidemic heroin abuse in the eighties have given us some of the most compelling evidence we have of the links between drug abuse and social disadvantage. Some of the indicators of social deprivation are unemployment, overcrowded housing, single-parent families, and lack of skills. The poor are becoming poorer in the face of diminishing opportunities. They must not only live with less, but have fewer avenues of support available to them.

Throughout the province, I heard numerous accounts of multi-generation welfare recipients and the homeless, hopeless people who are living on the streets of Vancouver and other large metropolitan areas. Indeed, economic hardship hurts families, perhaps creating an increased vulnerability to drugs. For many, the effects of unemployment or low income jobs are not easily offset by the resources of family or friends.

Material, cultural, and familial changes have had a profound effect on our children, our youth, our society's future. I heard from addicts who say that drugs provide them with a sense of structure: a relief from stress, depression, and the reality of their continuous misfortune. Often drug use is the only reprieve from their troubled and chaotic lives. There is no other meaningful purpose; there are no other alternative activities for them. Many of them have never had a steady job, indeed in some cases they may have given up hoping for a better life.

Poverty, polydrug use and poor health go hand in hand. In Canada, it is reported that one in five children lives in poverty. In British Columbia, poverty is clearly evident in Vancouver's Downtown Eastside and other surrounding areas. Daily life is much more difficult for those in the poorest families and communities, compared to the better-off communities. It is harder to cope day after day with housing problems, financial insecurity, lack of transportation, and lack of work. Indeed, the poorer the community, the fewer the opportunities for proper health care, child care, and education.

In many areas I visited, service providers reiterated the need for meaningful jobs. In fact, many of the recovering addicts we spoke to were now looking toward the substance abuse treatment field for meaningful employment. One community activist pointed out that the system is part of, and may contribute to the problem. He would like to see almost eighty percent of the jobs in the area go to local, disadvantaged people. He sums it up like this:

> *"What happens to the poorest community in Canada; are we the forest for the care workers?"*

For those people who are addicted to alcohol and/or drugs, there is even a stronger connection between achieving long-term abstinence and having a stable job. It does not take a rocket scientist to figure out that if a person does not have meaningful work to go to after treatment, then he/she is at risk to continue the cycle of drug abuse. Indeed, many of the supportive recovery homes and long-term treatment facilities stressed the need for job reintegration and job skills re-training for these people. Furthermore, there is inadequate support in the community for independent living, for securing employment and accommodation. Part of the problem is just educating substance abusers that various programs do exist. This reinforces the concept of providing program information to people at a very young age so they will understand about treatment options, rehabilitation, and other self-help groups:

"I never knew there were any programs. The biggest part of the problem was that I didn't know."

One recovering addict suggested that there be an incentive program to clean up for those addicts on welfare. It could be in the form of more money to get skills training or educational upgrading. It could also be in the form of more resources to help the recovering addict in finding better housing, preparing a resume, and gaining strategies for successful job hunting.

Poor performance in school may eventually result in a youth opting out of the educational system. This may well predispose the individual to low-paying, menial jobs or dependence on social assistance. The results can be measured in increased costs to society due to lost productivity, poor physical/mental health and increased demands on police, courts and corrections. It is reported that one male high school dropout will earn $149,000 less in his lifetime and collect more than $10,000 extra in welfare and unemployment benefits. Over a 20-year period, from 1990 to 2010, the cost of dropouts to Canadian society will be an estimated $14 billion in lost income and $620 million in more unemployment insurance benefits.

The largest concentration of poverty is in single parent families, most of which are headed by women. Children have fewer adult caretakers, supervisors, and mentors, and are therefore more vulnerable to the peer cultures of the street. One worker summarized:

"The real culprit is the user's own low self-esteem. The onus should be on parents and the school system ... failure to pose an educational and ethical challenge for young people. Kids from good families are given too much freedom. The school day is very short ... many students are on the street by two in the afternoon."

I therefore RECOMMEND THAT the Ministry of Social Services:

32. Examine ways and means of creating and implementing job placement strategies for welfare recipients returning from alcohol and drug detox, recovery, and treatment programs.

Part B POVERTY/ UNEMPLOYMENT

Part C WOMEN AND CHILDREN

"What turned my life around from being an active drug addict were people who believed that I was a worthwhile person and who had the patience and compassion to deal with me while I came to believe this myself."

From my interviews with women who are or were addicted, it is clear that improved treatment options are urgently needed. The issues for women are somewhat different than for other groups identified in this report. There is a major gap between what women identified as their needs and the existing treatment system. Services specifically for women and children are extremely limited and fragmented. Indeed, I heard from many addicted women that co-ed detox facilities, which are largely populated by men, may not have realistic entrance requirements and may be neither appropriate nor comfortable for many women.

Women identified the biggest single problem as the lack of provision for child care. Women who abuse drugs are more likely than men to be caring for children. The problem also extends to residential programs; few accept children, and not many women addicts can afford high-quality outside care for their children while they undertake residential treatment, even if they were willing to leave their children for that long.

Compounding this situation is the perception and experience of long waiting lists for alcohol and drug treatment for women. It was not uncommon to hear complaints of a one to three week waiting period for access to detox. I clearly heard from all corners of the province that access to detox ought not to be dependent on the unpredictability of a waiting list. If we are really serious about providing viable

options for women, a range of settings, both institutional and non-institutional, should be provided. Furthermore, funding is needed for a wider range of treatment choices for women and families, which would include residential and non-residential options and would fully address child care needs:

> *"A recent survey of Violence Towards Sex Trade Workers identified 60 percent of the women as mothers. Most of these women were drug-involved, poor, young, and unhealthy."*

The lack of understanding of patterns of sobriety and relapse into the chemical dependency disease process may cut women and their children off from supportive resources when relapse occurs. For some women, substance dependency may be a lifelong struggle and thus treatment options must include the care of children as well as their mothers:

> *"I'm a heroin addict and I think I was born this way. Mom was a heroin addict when I was born. I've tried to quit numerous times, going to detox and through treatment. Welfare only gives you a minimal amount of money to live on. This restricts where I can live and puts me right back into this location."*

I have also heard that there are twice as many men affected by addiction than women, yet there are four times as many men in treatment. According to officials with Alcohol and Drug Services the actual average female to male ratio in treatment for British Columbia is about 25:75. In any case, it appears that women do not seek treatment as often as men do. This may be due to a number of factors and barriers.

The identification and treatment of substance use and its impact on children and their families can be restricted by professional ignorance, denial, and prejudice. We need to move beyond social and racial stereotypes and treat individuals, not populations. Substance abuse issues must be taught and discussed at all levels of health professional education, including continuing education:

> *"Poverty issues, racism, etc ... we've got to get to the kids ... we need supports in school; good day care and someone who believes in the child."*

In terms of the Downtown Eastside area of Vancouver, there was much concern voiced about the shortage of designated detox beds for women and women with children. However, of the three detox facilities in the downtown area, two of them admit male and female clients on a demand basis, with the waiting periods varying from facility to facility:

> *"I did get into the detox for eight days, but they are not equipped for women ... it also took me three weeks to get in. The shelters are all full."*

> *"I know a woman who was trying to get into detox during a crisis; the guy told this woman she was too stoned to go into detox ... she asked him why she would be phoning them unless she needed the service. He hung up on her."*

Conversely, I also heard admissions that the existing detox services were used for emergency shelter:

> *"Let's get honest ... I've gone to detox because I've blown my welfare cheques. Detox is nothing ... it's a bloody daycare centre."*

Many concerns centred around the unavailability of flexible detox options that are specifically oriented to women of child-bearing age. Women in detox are frequently faced with difficult choices about child care during this period, and fear losing custody of their children:

> *"I did think about residential treatment but I couldn't leave my son; he was too young to be home alone."*

> *"I did all the things that Social Services and probation wanted me to do ... and my son was ready to come home; I'm a good mom --- but I confessed to being an addict. I was honest with Social Services and they apprehended my son for permanent guardianship. Now my son is five years old and my daughter is 15 and wants to adopt him when she is 18. Kids are getting hurt out there. They will take a child away from someone who is addicted, for no reason."*

"My son's father just got out of jail for armed robbery; they gave him custody over me because I am a drug addict ... he is also an alcoholic and a drug addict,but because he got another woman pregnant and started another family it looked good in the courts. You try and take an animal's baby away from it and see what happens; we don't even do that to animals ... I am so angry and will be for the rest of my live ... and then they wonder why you want a fix."

"There is still no support for mums in the evenings ... there was a woman recently who left her child in Oppenheimer Park; the child was apprehended. She apparently had no place to leave the child."

There have been efforts to provide improved services for women in some of the existing detox facilities, some of the specialized programs and long-term residential treatment programs for women are now offering child care. Unfortunately, these efforts are too few, the waiting lists too long, the barriers to access too great, especially for more marginalized women and children.

I have been told that women who abuse drugs are more likely to be socially isolated — cut off from formal and informal sources of support — in many cases, isolated from even their family. Clearly for these women who abuse drugs, the drug itself is only one aspect of a much larger set of complex and interwoven problems. For them, substance abuse is often a means of trying to deal with these problems.

The social isolation and stigmatization means that many addicted women are rarely able to call on relatives or friends to help care for their children. Few are willing to give up children to foster care in order to enter residential treatment. This ensures that many addicted women will not go to get help. For these women and children, the cycle of addiction goes on. The children continue to suffer, and "windows of opportunity" and hopes for the future are shattered. I heard from a female addict who started drugs at 16 years. She now has a son who is 16 years old, but she has had no support from the father or from her own family while raising the child:

"I have an older brother and sister, but don't have much of a relationship with them. My sister is a drug and alcohol counsellor, but she has a mainstream view and sees abstinence as the only way. My brother and sister have the Canadian dream (a home, good jobs, etc.) and are not very sympathetic to me or my situation."

Poverty is an underlying issue in the lives of women and children. Mothers run out of money for food the third week of every monthly welfare cheque. For some women this is a particularly vulnerable time:

"The pressures are overwhelming. Everywhere you turn you hit a brick wall. Employment opportunities are nil, housing is nil. Who wants to stay in a house that's like that ... there's cockroaches everywhere."

"Stress, malnutrition is a problem with us here ... there is a lack of communication; these people don't have any phones."

A lack of safe, affordable, accessible housing means that some women are bringing their children up in hotels, rooming houses, or sub-standard shared apartments. Many of these places are "shooting galleries":

"Women need safe housing here; lots would move to the suburbs. There needs to be improved opportunity and improved quality of life. Housing is a major issue. Outreach is important to contact illiterates, etc. Many women are very isolated."

"Sometimes the rooms are so dark ... they are the size of a jail cell, about eight by ten. The cost is $300/month, which is the welfare standard. There is no way to reach anybody from the outside."

Often when we think of substance abuse, we tend to only consider the user. It is critical that some of the secondary victims be recognized: abused women and children. The lack of options for many of these women may have led to their own drug use in the first place. It also locks them into relationships with abusive and drug-using men. While substance abuse may not cause family violence, it is definitely a contributing factor. It is common to find violence,

sexual abuse, and substance use in the same family. Tragically, many of the addicted men and women whom I spoke to cited family addictions (notably alcohol) and sexual and physical abuse as some of the precipitating factors leading to their substance abuse:

> *"Many, many deaths of women occur weekly. Most of them do not make the newspapers. However, these women are aunties, sisters, cousins, grandmothers, and children of the women we work with. The systematic destruction of their families through deaths from alcohol and other drugs is devastating."*

> *"The cities are killing grounds for our young women. When I look at our current system of care in BC I see a critical missing element: residential treatment facilities that focus on women and children. In our society, children stick with women; there are not many male addicts with children in tow. We must interrupt this cyclical process. We need to seriously look at dealing with a very devalued element of our society: women who are addicts and have children."*

> *"Most women have experienced multiple trauma: abuse, violence, multiple deaths, and sexual abuse."*

The perpetrator of the violence is not always the substance abuser, however. The chemical dependency may be with the victim. Women in violent homes may use a variety of ways to cope with abusive behaviour. Transition houses report that 72 percent of the women in their homes are abusers of prescription medication, alcohol, or illicit drugs.

For many, the surrounding environment exacerbates the problems of addiction, family violence, poverty, and marginalization. Women in the Vancouver's Downtown Eastside expressed their concern about the drinking establishments in the area:

> *"I'm concerned about there being 7,000 licensed drinking seats in my area (the Downtown Eastside): 80 percent of all of Vancouver's seats. That's a crime! New licensing in BC should have to purchase these seats from a pool of seats held by the City of Vancouver until such time as the number in this area is reduced."*

Women may fear going to jail or losing their children; often they are also frightened by the male street subculture that surrounds many treatment programs and alienated by the rigidity of therapeutic/community programs. These barriers will continue to keep addicted women from seeking treatment.

In Prince Rupert, one woman (amongst many others) pleaded for a local methadone program to assist her in getting off heroin. There, people on methadone maintenance are bused to Terrace to receive treatment. I was told that Social Services pays roughly $15,000 per month to transport a dozen or more people. Furthermore, the regime of the treatment and the travel requirements mean that none of the people on methadone can maintain a job or care for their children:

> *"I'm an addict who has been struggling for years. If there was a methadone program here I might be able to kick my habit. I have six children I'm trying to raise; I'm a good mom. I have a problem with marijuana, heroin, cocaine, and alcohol. Some of these I've conquered, some I haven't. I can't leave my children to go for treatment, but you can't just walk away from heroin. I can't get out of bed or work any more. I do have a chance to go back to work, but I can't without a methadone program."*

Treatment must be broad enough to identify the role these factors may play in the lives of our families. Furthermore, services for children and families must be culturally sensitive and should take advantage of existing strengths, for example in the First Nations' communities:

> *"Women and children are in crisis and we need an appropriate response; not a treatment program in Vancouver, but here at home where the problems exist. We need child care programs for the women in treatment. We need day programs which allow for follow-up care. The women need a local program so that they can hang on to whatever*

support or familiarity they have left in their lives."

In some areas, such as the Downtown Eastside area of Vancouver and elsewhere throughout British Columbia, successful intervention programs have begun. These include priority admission for pregnant and post-partum women to detox centres, pregnancy outreach programs, public information campaigns, government warning signs at point-of-purchase of all alcohol beverages, a poster campaign by the British Columbia Medical Association in five languages, kits distributed to pregnant women including alcohol and pregnancy information, strategic plans which have been developed for the province to deal with alcohol-related birth defects, and media coverage concerning the effects of alcohol and drugs on infant outcomes. These programs recognize the importance of early prevention and intervention strategies. However, much more needs to be done in order to make a difference in the lives of many of the people I spoke to. We must also remember that some are illiterate.

During my discussions with recovering women, they stressed that linking treatment to training and opportunities for good work is critical for their success, since many of them are trapped in a spiral of drug abuse primarily because they have so few options for legitimate self-support:

"Some of the staff here have come from the streets. This provides employment opportunities that teach them the work ethic. This provides opportunity to give support, improves the learning process, and increases self-esteem. The workers become role models. In many cases it's pre-pre-employment."

A major focus should be for women with substance abuse difficulties who become pregnant. Barriers to treatment may keep addicted women from obtaining prenatal care of any kind. Clearly the consequences of avoiding care can be catastrophic and tragic, particularly when the consequences are largely preventable if adequate care is provided.

Substance use in pregnancy is often confounded by poverty, poor prenatal care, poor nutrition, a history of sexual and physical abuse, and limited access to appropriate parenting role models. The identification of infants born with exposure is often difficult because of fear around the real or imagined repercussions of disclosure. Compounding this situation is early discharge from maternity hospitals. These facts may leave women and children with inadequate options for treatment and limited information about the special care needs of babies and support available to them in the community.

Chemical dependency has a wide impact on individual children, their families, and the communities where they live. Drugs can disrupt the normal development of the embryo and fetus before birth. The range of effects vary depending on the dose, timing, and conditions of exposure. For alcohol, the greater the exposure the greater the effects. Binge drinking (five or more drinks per drinking occasion) even if only engaged in infrequently, is a particularly risky pattern of use. Effects also depend on the individual susceptibility of the mother and the fetus. Children exposed to cocaine and other addictive drugs in gestation may also have higher rates of congenital anomalies, neurobehavioural aberrations and infant mortality (e.g. Sudden Infant Death Syndrome, or SIDS).

Although some children with prenatal exposure may grow up without measurable lasting effects, their combined biological and environmental vulnerability often results in permanently limited potential and disrupted lives. This may include developmental delay, learning disabilities and behavioral problems in school, vocational issues, and adaptive function as adults. This cycle often repeats itself from generation to generation. We know that this vulnerability is preventable, although solutions are in no way simple. Fortunately, there are individuals and families succeeding at making changes in their lives and finding health.

The most immediate neurologic complication seen in narcotic-exposed infants is the presence of a Neonatal Abstinence Syndrome (NAS). This is where the infant is born addicted to a narcotic and begins the withdrawal process at birth. The symptoms include irritability, tremulousness, sweating, stuffy nose, difficulty feeding due to uncoordinated and inefficient suck, diarrhea, and vomiting.

Cocaine readily crosses both the placenta and the blood-brain barrier. Studies have shown an association between cocaine use during pregnancy and a decrease in birthweight and head circumference. However, the studies caution that this effect on growth is probably compounded by maternal undernutrition and polydrug abuse. Early cessation of cocaine use in the first trimester improves the

outcome for the baby and therefore reinforces the importance of entry into drug treatment as early as possible in pregnancy.

Prenatal exposure to alcohol may result in the most common, yet preventable, causes of mental retardation, Fetal Alcohol Syndrome (FAS) and Fetal Alcohol Effects (FAE). FAS is a combination of physical and neurobehavioral birth defects which may develop when women drink alcohol during pregnancy. It is an organic brain disorder which manifests itself in central nervous system damage, growth deficiency, physical anomalies and behaviour, and learning difficulties throughout life.

A child who has a history of prenatal alcohol exposure, but who does not manifest all of the physical or behavioural symptoms of FAS, may be categorized as having Fetal Alcohol Effects (FAE). A child with FAE may not have all the physical abnormalities of FAS. However, the cognitive and behavioural characteristics can be similar and equally as severe. FAE, like FAS, can have equally serious implications for education, social functioning and vocational success:

> *"I lost my sister to a drug overdose (heroin/cocaine). She was 30 years of age and had two beautiful children. One was born addicted; he cannot cope in this world. I cry now when I take him to school and he stands in the corner because he knows its going to be another hard day. I grieve for this child and for his mother.*

> *"I believe that things happen for a reason, so that I could learn from her death. I don't know how her son is going to turn out, but we are going to do our best ... he loves us and we love him."*

It is reported in the US that the medical and educational costs for caring for one FAS child for his/her lifetime may exceed one million dollars. This surely points to the urgency and prudence of early prevention and intervention strategies for young women.

One example of an excellent client-oriented service is The YWCA Crabtree Corner. This is an emergency, short-term daycare and family resource centre located in Vancouver's Downtown Eastside. The centre provides programs, referrals, advocacy, and counselling. They are targeting high risk families: prenatal alcohol and drug abuse and infant outcomes. Eighty percent of these families are First Nations and ninety-five percent are on social assistance. Violence is a daily reality: physical, sexual, and emotional abuse and multiple traumas contribute to women's substance use, misuse, and abuse. There is, indeed, a pressing need for more of these special needs daycare centres in the Downtown Eastside area, beyond that which is being provided by Crabtree Corner.

Outreach programs, such as the SheWay project in Vancouver, also offer an integrated, multi-disciplinary approach to these problems. SheWay is a Coast Salish word meaning "growth." This new inner city project is an antenatal and postnatal outreach and support for women and their children. SheWay provides women and their families with pregnancy and nutrition counselling, alcohol and drug counselling, support for medical care, housing, transportation, and food. Support groups are offered during their pregnancies and infant development monitoring is provided once babies are born. The centre also provides free baby food, formula, diapers (when available), and parenting support for the mothers. These are prime example of how non-profit social and health service groups such as Vancouver Native Health, Crabtree Corner, and Children's Hospital can be partnered with government departments (the Ministry of Social Services and the Ministry of Health) and the Vancouver City Health Department.

One of the major problems identified was the lack of ongoing funding available for such innovative support systems. Lack of secure funding hampers the best service delivery. These types of programs are too few and are either not funded, underfunded, or made to walk a tight rope of funding red tape. These non-profit service agencies never know from one funding request to another where or how much they will have for operation. It is extremely important for the general public to understand that services like this one are making a big difference in the lives of women and children, yet the bureaucratic demands actually hinder rather than assist in the process. It is critical for services which target the high risk groups and provide day care and ongoing follow-up to have ongoing and assured funding. This is what I heard from Crabtree Corner:

> *"Funding for the FAS prevention project was difficult ... initially the provincial government did not respond to our funding request, but the federal government came up with some funding. We don't have the infrastructure to be*

always writing proposals for the government ... it takes too much time."

"We are running at a $100,000 deficit each year. The YWCA covers this."

"Crabtree daily sees women struggling with alcohol and drug issues. We have only one community outreach worker and one FAS/NAS prevention worker to do the work. We are underfunded and overwhelmed. We desperately need two additional community outreach workers to go into hotels to connect with women and their children."

At Sunny Hill Health Centre for Children in Vancouver, the Infants and Children at Risk Program became involved in the Coroner's Task Force following one fatal and two near fatal heroin overdoses by mothers of three children in the program. There were also several children whose mothers had been the victims of drug-related homicide. The specialist physicians, nurses, and caregivers at Sunny Hill are painfully aware of deaths and disability that result from ongoing addiction. They provide care for infants and children prenatally exposed to alcohol and other drugs and also support their families and community caregivers.

For the last five years, Sunny Hill has cared for 45 - 70 infants per year as in-patients for drug-related withdrawal symptoms. As out-patients, children up to 19 years of age are also seen for multi-disciplinary assessments which focus on the developmental, educational, and behavioural effects of prenatal substance exposure. Families are encouraged to remain involved in the care of their infants, thus minimizing the effects of separation. Parents are accommodated for a period of time at Sunny Hill. Sunny Hill liaises with the family physician and other community supports to address some of the medical and social issues facing the family. In addition, Sunny Hill offers medical and nursing consultations to communities where they live. Over 700 of these children from birth to age six receive ongoing follow-up.

Clearly, in British Columbia, FAS, FAE, and NAS are public health issues with broad significance. Interwoven with them are complex social issues of poverty, sexual abuse and violence, racial and gender marginalization, and HIV. Understanding the effects of drug use in pregnancy is complicated by the presence of many confounding variables associated with the drug using life style, which may have an independent effect on infant outcomes (e.g. failure to obtain adequate prenatal care, cigarette smoking, alcohol use, low pre-pregnancy weight, and low weight gain during pregnancy, acquisition of sexually transmitted diseases during pregnancy and polydrug use).

To emphasize the magnitude of the problem, a recent study in the Downtown Eastside area of Vancouver recognized that the rate of drug and alcohol use during pregnancy was as high as 36-46 percent in two census tracts:

"45.7 percent of 1535 infants born in the Eastside of Vancouver have been prenatally exposed to alcohol and other drugs ... there is a high incidence of FAE, if not FAS, in this community."

However, the problem exists in other more remote areas of the province, too. In northern BC and the Yukon, a study of 586 children with disabling conditions found that FAS and FAE accounted for 31 percent of children with disabilities (14 percent of the children had FAS and 17 percent showed FAE). Fifty-nine percent of the children had learning disabilities and 59 percent had speech/language disorders; all of these also had alcohol-involved pregnancies.

For some women and families, having a child can lead to a detoxifying experience; our public and private resources would do well to support them at this time in their lives.

There is a lack of adequate early identification, treatment and follow-up services for infant children and adolescents born with prenatal substance exposure. The general health and developmental services provided through community health nurses and Infant Development Programs offer an excellent opportunity for developing a comprehensive treatment of this problem. However, this cannot happen without adequate training, funding, and broad social and political support.

In addition, there is a scarcity of basic research of the developmental outcome for children with prenatal drug and alcohol exposure within the Canadian social and cultural context. There is also very little known about successful treatment interventions for individuals and families. We do not understand why some children are resilient to

difficult and unpredictable circumstances. With some understanding of what supports success, we can begin to address prevention more effectively. At present, we do not have even basic prevalence data about the number of children born in the province with prenatal substance exposure. Without this essential information, it is difficult to plan future directions in this field here in British Columbia.

During the final stages of writing this report, I was encouraged to hear that the Minister of Health has announced the establishment of a new Centre of Excellence for the Treatment of Women's Addiction Problems. The centre, to be located at BC Women's Hospital and Health Centre in Vancouver, will combine intensive treatment for substance misuse along with research and training for service providers. The centre is scheduled to open its 25 residential beds in January 1995. I am hopeful that this centre marks the beginning of a focused and continued effort to address the needs of women, not only in Vancouver, but in the outlying communities as well. It is important for the public, and for those who control the purse strings within government, to recognize that the biological vulnerability caused by prenatal drug exposure, coupled with the environmental vulnerability which often follows, puts our children at risk for significant adverse developmental outcomes that go far beyond any exposure during pregnancy. As a society we must adequately provide for this significant population at risk.

In summary, over the course of this review, several major themes or principles emerged. Some, like motherhood, were hard for anyone to disagree with. Other principles were, indeed, more focused. All of them, however, were insightful and in my view had some merit. I encourage readers to think deeply and carefully about the issues related in this chapter.

One of the most frequent principles expressed was that the enigma of substance abuse should be the obligation of all levels of government and multiple ministries. People were angry and frustrated that if their particular problem didn't fit a certain profile or a certain ministry mandate, then they as individuals were insignificant, perhaps even disposable. It didn't matter if they fell through the bureaucratic cracks. Most everyone I spoke to cited the need for improved collaborative initiatives to fund and evaluate education, prevention, and treatment programs, especially for women and children. The perfect example mentioned earlier is the day care issue. Substance abuse issues must be taught and discussed at all levels of government and health professional education, including continuing education.

The next most important principle was that there should be a leading focus on women and women with children who had substance abuse problems. Here, education and support for these young mothers was felt to be the key to the prevention of FAS, FAE, and NAS. In this arena, the first most critical tenet would be to protect existing successful programs that are a vital part of a comprehensive approach to prevention and treatment (e.g. the Needle Exchange, Crabtree Corner, SheWay, and others). Then new or improved programs must be established to meet the demand.

I therefore RECOMMEND THAT the Ministry of Health:

33. Establish local treatment centres for family substance abuse, incorporating special needs daycare for children whose parents seek short or long-term substance abuse treatment.

I therefore RECOMMEND THAT the Ministry of Social Services:

34. Provide adequate day care, travel, and financial support to mothers attending substance abuse treatment programs;

35. Examine ways and means of increasing the availability of appropriate housing, encompassing a range of settings, including community homes, independent living arrangements, safe houses, and transition houses for recovering addicts, particularly women, children, and the disabled, in order also to discontinue exposure to an alcohol and/or drug-violent environment;

36. Provide increased funding for support programs which target women with substance abuse problems, including a wider range of treatment choices for women and families which address child care needs with residential and non-residential options;

37. Review ministry policies and practises which remove children from mothers or families suffering from addiction, recognizing such threats to parents seeking treatment and the benefits of early intervention and counselling referrals to treatment agencies.

I therefore RECOMMEND THAT the Ministry of Attorney General:

38. Discuss the merits and possibility of obtaining William Head Institution from Correctional Service Canada (CSC), for the purpose of converting it from a federal to a provincial centre for women and children, to be used in the detox, treatment, and supportive recovery of people suffering from substance abuse and related problems.

Part [illegible]
WOME[illegible]
AN[illegible]
CHILDRE[illegible]

Part D
FAMILY AND YOUTH

"Heroin was the master of my son's life and played a big role in his death ... I always stood by my son, bailing him out of jail time and time again, knowing each time, after each court hearing that jailing him up was not helping him at all. I gave my son all the love and help I could ... I've cared for him while he's been sick, but could never give him the treatment he so desperately needed ... Thank you for caring for people like my son when the majority of people have forgotten or would rather not hear about them at all."

– Letter from a mother with a broken heart.

Family is primary; they are the first caregivers, even before birth, the first examples a child copies, the first models for behaviour, the first teachers of coping mechanisms that will be part of the young person's repertoire of responses to the world. The importance of these early experiences cannot be stressed enough when it comes to substance abuse.

When I refer to family, I not only refer to the traditional concept of family, but to all variations imaginable -- indeed, to any people charged with the guardianship of young minds and bodies. This too, in my mind, includes the larger extended families and the community as a whole.

The first potential impact or parental influence has been dealt with: the exposure of the unborn fetus to alcohol or drugs through consumption of these substances by its mother. Until very recently, the effects of FAS, FAE, and NAS were not known. The thalidomide babies brought home the importance of substance transfer and effects beyond the umbilical boundary. This topic has been examined under other headings in this report and only underlines the importance of family behaviour on the developing child, even before birth.

Time and again, the importance of parental examples were demonstrated in stories I heard around the province. The impact of homes where substances were abused is one of the strongest common determinants of future behaviour. First, there is the immediate effect: parental neglect or absence, children having to fend for themselves while the parents partied. Then, there is the tragedy of physical and sexual abuse associated with inebriation or the toxic effects of alcohol or drugs. Many stories were told of the battering and brutality of parents toward each other and toward the children in advanced stages of intoxication. I was advised that nearly all addicts registered at needle exchanges have reported physical and sexual abuse in their homes as children; they are scarred, often for life, by the memories of violence in the home and toward the children.

Many of the young women involved in sex trade work on the streets of our major cities have backgrounds of sexual and physical abuse by parents and family who took advantage of them while under the influence of alcohol or drugs. The great majority of

the First Nations people residing in Vancouver's Downtown Eastside report these conditions on their home reserves and cite this as the number one reason they would not consider a return to their home communities. Despite how difficult, unappealing, and deprived the current situations are, for many they would rather suffer these indignities than contemplate returning to their reserves. I heard of one native girl who died of an overdose in Prince George. People who knew her told of how she, as a little girl, had been handed around as a sexual party favour on a routine basis during drinking parties.

Is it surprising that we have such damaged lives, who in turn have such low self-esteem and who turn to alcohol and drugs to kill the pain? I heard many accounts:

> *"My family were always laughing and happy all weekend ... I didn't know until I was older that they were always 'snapped on booze'."*

> *"My family were always drunk. I said I would never drink, so when I left home I went straight into drugs."*

Certainly, these negative examples of parental behaviour are found at one end of the continuum in contributing to substance abuse, but what of the hard-working parents who are busy putting the good things in life together for their families, as was described in the Okanagan? Typical is the young married couple who wants to start out with the perfect life in the perfect package. Social workers described how, at the outset, the newly married couple wants a big home, new furniture and appliances, a new car, and a boat. The fact that both parents will of necessity have to work often means that they will not be there for their children, to guide, to console, to listen, to be interested, or to be vitally involved in building personality and self-esteem. These children tend to be the "latch key kids." One physician noted that he was happy his wife was able to be home when his children got in from school. He noted that their home tended to be a gathering point and that many of their children's friends came over after school to socialize with their kids and talk to his wife about their problems at school and home. While time-consuming for his wife and a strain on their food supply to some degree, this was the best insurance, as he saw it, that his children did not get into drugs and alcohol or sexual activities after school as was happening in some of the other parent-free homes in the neighbourhood:

> *"I'd love to see it where one of the parents could be home at 3:00 every day ... have staggered hours ... to be there for the kids. The kids are always at our house.*

> *"That would make a big change in our society ... it's a built-in generation gap. It used to be you took your kids everywhere ... the extended family concept doesn't exist here any more."*

There are also other families with dysfunctions of another sort. Lack of love and denial of emotion was felt to be the underlying cause of misplaced values in another individual who indicated that he came from a family where winning and excellence was everything and tears were not tolerated, certainly not for the boys. After losing everything to a cocaine habit — business, friends, wife, and children — one young man in recovery told me that he learned during his recovery about true family values and for the first time since he was a toddler he had "hugged his Mum and Dad on Mothers Day this year." He found it sad that he had to lose everything to make that discovery, but he will now be able to rebuild his life. He was 35 years of age.

Family and home environment are very important in the equation of healthy adjustment and often, though not always, an absolute necessity to building healthy lives. So much more could be written about the family and home, about the loss of traditional values and respect for others and their property. A native chief and elder eloquently stated at a public hearing how saddened he was at this breakdown of the fabric of society. He referred to what had happened in his native community and in the white community as well. We live in a materialistic, hedonistic society: the "what's in it for me" world. When faced with the unavailable and unattainable, irresponsible and unable to cope with reality or to escape the competition, shame, and pain, some resort to substance abuse for transitory pleasure.

There seemed to be a natural inclination, on the part of those who came forward, toward family. We often heard the expression: "I lost everything - my wife, kids, family." There seemed to be a natural longing for the support of the mother and father, in spite of some of those parents being lost in the fog of substance abuse. Always, there was that commitment on the part of parents who did not want their children leading the life they now found themselves caught up in.

Family and values; they seem to figure significantly in the life and death of the heroin addict:

"The real issue is our societal ability to value, protect, nurture, and teach our children, whether in a family context or not."

Of the addicts I spoke to, most started some form of substance abuse in their teen years, some in their preteen years. This was generally in response to a range of problems, complex and interrelated, including physical and sexual abuse, joblessness, homelessness, peer pressure, and boredom:

"Nearly one hundred percent of the girls in youth detention in our community have been sexually abused, and three quarters of the boys have been sexually, physically, and emotionally abused. These kids are in adult bodies and, with their backgrounds, it's a wonder they make it at all."

"We get parents phoning us all the time and asking us to fix the kid. but what about them? ... its the whole thing; 90 percent of the kids on the street have been sexually abused."

As with other age groups, substance abuse crosses the social strata. Youth is one of the main targeted groups; they can tell you where to buy the drugs, whereas the adults in the community cannot. It is part of the culture. Indeed, like it or not, our youths are the targets and they often have the means to buy drugs. One counsellor expressed his concern about the profitability and attitudes of some:

"Not long ago, Probation sent me a youth for counselling. When I asked the youth what it was all about, he explained he had been picked up selling drugs at the arcade and advised he was a seller, not a user. At the time he was living on the street but now that he had been picked up, he made up with his parents and now his overhead was reduced because he was back at home. Selling drugs was now so profitable that he was making more money than his father: no GST, indoor work, and a high level of contact with his friends. He could not understand why this was not a good activity to pursue."

Many people I spoke with were very concerned about youth moving into minor criminality as a form of recreation. Youth are seeing drug use and the drug culture as a very viable alternative. Explanations for this departure from the "norm" values included instant gratification, perhaps a reflection of their parents "keeping up with the Joneses." They may be the casualties of the "more and best syndrome" or simply bored with their lives. In Prince Rupert, counsellors reflected on the home environment vis-a-vis parental involvement and coined the phrase "the B parents: Bingo, Bars, and Board Meetings," meaning that parents were just too damned busy and they often presented the wrong role models:

"Parents think they can smoke dope and not effect the kids."

Drugs are not the main problem in their lives, but rather a means of coping with more pressing problems.

Alcohol abuse was cited largely in the backgrounds of many who came from dysfunctional families, with one or both parents being alcoholic. However, each case again was very individual:

"One of the toughest factors was that the parents wanted to party with the kids (this boy had sought help for his substance abuse problem) ... the parents offered him dope and booze."

"The youngest we are dealing with is 13. There are addictions in the family background and the whole family is in recovery: an alcoholic and co-dependent environment, second generation. The child is out of school at 13 and cannot return until September. He started experimenting at nine years of age."

"The ages are getting younger, needles are being found in parks frequented by local youths ... It goes back to the family. When we ask them if their parents know where they are, they respond, 'They don't even know what day it is!'"

In some cases parents, in frustration, wash their hands of these "delinquent kids." Often, by then it is too late to work with the families:

> *"We have seen an increase of 12 - 16 year-olds who aren't even living at home. They are either living on the streets or staying with a friend after their parents kicked them out."*

Some people I talked to felt the justice system was partly to blame, with the family losing its ability to discipline the children. In theory, the government has placed a high priority on adolescent prevention services. However, at the street level, the written commitments have largely not been supported with concrete services. As one alcohol and drug counsellor put it:

> *"Policy wise, we're good, but we don't have the resources ... For example, youth workers on site; what's the use of the policy if we don't have the follow-up resources."*

Concerned caregivers and outreach workers in all parts of British Columbia reflected that there are increasing numbers of younger drug users, 12 years and up. Frustration was also expressed over bureaucratic red tape which drives the cost of health care up, resulting in few programs being able to meet the rigorous standards. Often people used the expression "Penny-wise and pound-foolish" to describe the poor funding situation which virtually guarantees that few programs can fulfil their potential:

> *"There is only one program in the Okanagan for juveniles ... the cost is five times as much because 24 hour care on a one-to-one basis is required. Community licensing makes it more difficult; regulations drive this."*

> *"There are too many juveniles — 14, 15, and 16 — who are regular drug users — Pot, LSD — but they are working their way up. They are involved in gangs and criminal activity to support the drugs. How do we effectively deter that problem so they can get back on track? Youth treatment programs are almost non existent. How do I get a young offender off drugs through treatment programs when the service isn't there?"*

Alcohol and drug use is perceived by social workers and counsellors to be not necessarily a cause, but a facilitator in many of our youth suicides. Again, people who were trying to ameliorate the situation recalled tragic instances where clients completed suicide while taking drugs. Another case worker expressed her frustration over the lack of psychiatric resources for suicidal youth.

One professional reiterated that the best prevention happens with adolescents. He stressed that drug use usually has many different etiologies. It could be a psychiatric problem where the child is self-medicating. The child may be depressed, sometimes in the very early stages of manic depression or schizophrenia, and may fall into experimentation. Many kids are also suffering from lack of psycho-social and spiritual support during this crucial time in their lives. Many of the people we spoke with supported a very strong representation of advocates for families and children in any kind of planning:

> *"I recall a youth that we lost who committed suicide during a very bad trip on LSD; he was in the company of a friend. That friend survived, but had spent the whole night talking himself out of suicide too."*

> *"There has to be a preventive measure for children. There's no damned psychiatrists north of Nanaimo. We work with volunteers and it takes two to three weeks to even get an appointment for a suicidal youth."*

The general consensus was that it is better to intervene early than to wait for young people to become seriously addicted adults and then try to treat them.

Most of the youth at risk are not reached by conventional treatment and prevention programs. Many kids in need of help are falling through the gaps in services; this is especially true in the outlying regions. Although there are over two dozen Youth Outreach Reconnect offices throughout the province and a Mobile Youth Detox in Vancouver, some of the street outreach workers I spoke to expressed their dismay at the myriad of constrictions, restrictions, and jurisdictional problems for services. There was much frustration in their descriptions about getting

people to the resources. They suggested better inter-ministry planning and core funding for services and one agency to look after it all.

In most locations, people perceived that there was a lack of detox and residential youth services. In some cases, youths were referred to Alberta, creating a disjointed program which isolates the youth from parents and community support. Adolescent needs for detox can be more immediate. If programs are not accessible during a time of need, often this means the youth will go back to a pattern of abusing:

> *"There should be one Youth Ministry; there's Mental Health versus Social Services versus Attorney General ... it's crazy and so compartmentalized. The Youth Detox for kids is only for street kids; I can't put some kids in because they don't fit the profile. I do whatever I can to get the kids into Maple Detox, but boundaries are a real issue."*

Here, again, it is important to note that there is no official intent to limit or deny access to outsiders who are in need. Perhaps it is once again a case of demand exceeding resources:

> *"I picked up a patient yesterday in Burnaby. However the youth's home was in Vancouver. There was a bed in UBC, but they said he wasn't in their catchment area. I spent the whole day looking for treatment for him and he's still in Emergency; its a political thing about boundaries."*

> *"Kids have detoxed in our EPS homes because there is no place else to hold them. A lay couple working with this kind of behaviour with very little support from outside professionals is a very poor place to detox."*

> *"In Penticton there is no doctor-assisted or youth detox. The closest is Kamloops with a two to 14 day wait and youth are not accepted on weekends."*

> *"I tried to get a kid into detox and it was a joke. I took him to my house and kept him off coke. It was okay for a while, but I was given the run around. It was 14 days before he finally got in ... I had to phone every day. We don't have enough resources to deal with the major problem that we have."*

Still others felt that often youth mistrust conventional authorities and suggested there should be treatment options which are distanced from law enforcement and the juvenile justice system.

Most formal help for drug abusers is reactive instead of proactive and mainly provided to those who have already been caught in a drug-oriented life style for some years. By that time, much damage may have already been done: involvement in a violent drug subculture, sexually transmitted diseases, and other drug-related health problems:

> *"We get many, many calls for narcotic withdrawal but we cannot accept them ... enormous requests for youth, but it is not appropriate to mix them with an adult population ... the resources just aren't there."*

Treatment must be more attractive, accessible, and creative to abusers, especially those at high risk of becoming abusers in their natural surroundings. Most professionals I spoke with felt that there is an urgent need to reach young drug abusers early, but cautioned about placing too much of the responsibility on teachers:

> *"Education at the elementary school stage is really important. Teenagers are much harder to reach and many of the high risk kids have dropped out by that time. We have to get the parents to carry some of the load and take their blinkers off. Teachers are overworked."*

The matter of education is examined in a separate chapter. However, knowledge does not necessarily translate into decreased use: in some cases, it may actually increase use (e.g. where scare tactics are used). The simple facts about drugs and their actual costs are enough to convince most people. Exaggerations add nothing, and may create an appeal to experimentation by unsuspecting young people.

In this chapter the issues really cross all boundaries. However, the common themes expressed at the community level are acknowledged here. Since

Part D FAMILY AND YOUTH

some of the best prevention and intervention occurs with adolescents, the education system should be making alcohol and drug education/prevention a number one priority. The courts and the Ministry of Social Services should also take a lead role in recognizing that troubled and drug-involved young offenders need a variety of personal counselling, substance abuse treatment options, and ongoing family counselling if they are going to turn their lives around. Sentencing involved youth to an adult correctional facility is not appropriate and may harm the future of the youth. Moreover, a suspended sentence does not give the right message to a young offender. Most felt that sentencing options should include mandatory counselling and substance abuse treatment that involves not only the youth, but the family as well. Finally, without exception, people stressed the need for immediate across-the-board response for an adolescent in need of detox and follow-up treatment:

> *"We as a society in general tend to think that it's not my friend or neighbour and that's not a true picture. Our society doesn't think it's their issue."*

I therefore RECOMMEND THAT the Ministry of Health:

39. Improve and ensure access to detox facilities for young substance abusers (alcohol and drugs);

40. Continue and expand mobile youth detox programs to communities with an established need.

I therefore RECOMMEND THAT the Ministry of Social Services:

41. Develop and establish follow-up programs for parents and youth in care who have completed detox and treatment programs within other ministries.

I therefore RECOMMEND THAT Municipalities throughout the province take the lead role in:

42. Providing community activity centres which encourage constructive life styles and skills, with an educational component which incorporates counselling in substance abuse.

Part E FIRST NATIONS

"We should walk the road together and be equal partners in solutions."

One group of British Columbians who are separate and who distinguish themselves from the remainder of the population are the aboriginals, the native population, First Nations people (these identifiers are used interchangeably throughout the province).

When I first began this study, it was pointed out that 37 - 40 percent of the population of Vancouver's Downtown Eastside are First Nations people. Approximately 30 percent of those registered at the needle exchange near Main and Hastings are native people. It was also stated that alcohol was a greater problem than drugs within this group. That led to the observation that there are some 7,000 seats in beer parlours within a six block radius of Main and Hastings.

The number of First Nations people reportedly dying of drug overdoses in 1993 was 31 out of a total of 340 for all British Columbians. That number is probably larger due to some natives not being identified as First Nations. One of the issues that a number of aboriginal people brought to my attention was that the coroner's statistics (for illicit drug overdose deaths) do not reflect the whole picture of deaths from their community. They asked, "What about the murders and the children who died at home while their parents were out doing drugs or those people who just disappear and are never found?"

This underscores the point that the number of overdose deaths is only the tip of the iceberg in terms of human costs. And this does not take into account the deaths related to another abused substance: alcohol.

It has been demonstrated, historically, that the native suicide rate is higher than the average population and that many of these suicides are precipitated by alcohol and drug abuse in the family and community. We know that aboriginal people are disproportionately represented in risk populations, especially those for alcohol and drug treatment and in the provincial correctional system. Time and time again during my interviews, agencies providing treatment for aboriginal people who are alcohol or drug users have identified many of their clients (up to 90 percent) as victims of childhood sexual abuse.

Social Services and Native Health Services also noted in Prince George, Prince Rupert, and the Interior a heavy predominance of aboriginal peoples amongst those victims, and reported that many First Nations people in these centres are trapped in poverty, having escaped abusive backgrounds on their home reserves. The perception was that they do not easily access drug and alcohol services that are available. Indeed, I heard that there is a real need for specific detox (alcohol and drug) services for natives, particularly for females with children. There have been some very tragic losses in the past year in the native community in Prince George — some well known, as well as some youthful, victims:

> *"For native people, there is a gap in service, real or perceived ... they will not access mental health counselling but sometimes will access alcohol and drug programs ... it's frustrating because sometimes we don't know how to help them ... it is very hard for some of these people to find the inner resources to keep going."*

The native addict population appears as a visible minority within a visible minority. They were characterized by their brothers and sisters, by social workers, health care workers and law enforcement personnel alike as residents in a state of despair and hopelessness. The native people themselves expressed an internalized feeling of oppression, prodded by public expressions of racism. Not unlike the vast majority of addicts, they have a very low image of self-worth and esteem, the abnormal and unnatural extension of which ends in self-destructive behaviour. As noted above, all of these characteristics are present in the substance abuse population in general.

Attitudes of racism were cited by most people in most communities as an underlying issue for all minority groups, but especially for the native population. As one person pointed out at a public meeting on the issue:

> *"We are all victims of victims ... attitudes are caught; they are not taught."*

An hereditary chief and elder cited the need for values amongst people in authority. In referring to local politicians, police chiefs, native chiefs and the like, he forcefully articulated how they and society had lost their sense of direction because of the loss of solid, positive values in society and in themselves. In addition to pointing the finger at society in general, he was critical of his people as well; there was a lack of values in all:

> *"I have 18 grandchildren and I know the end result if things are not right. My family has been hit hard ... I also know of families of four that have disintegrated because of drugs. In my family, it was only one of my children, out of ten."*

The natives have an expression: when you abuse your body, your spirit leaves you. They said there are four ways an addict can end up: in jail, in a mental hospital, in recovery, or in death. One 44 year-old recovering native indicated he was 16 years of age when he became addicted. He had been abusing for nearly 30 years. It was a life without feelings or compassion. From the time he was addicted he started thinking of suicide. "Addiction is the slowest form of suicide I know of." Survival was said to be just one step above death.

In Vancouver, the DownTown Eastside/Strathcona Community Focus Group for First Nations expressed support for existing programs and acknowledged that great strides had been made in understanding some native concerns. However, they also noted the lack of knowledge or will to understand native styles and norms and their relationship to service delivery. For example, the practise of housing people in separate bedrooms in detox and treatment facilities does not recognize native cultural traditions which are based on close family ties and communal living.

Drug use is a social condition which persists because it meets human needs in a world of declining opportunities. People today are increasingly subjected to severe socioeconomic stress and insecurity. Furthermore, with ever increasing financial pressure on the social support network, the helping resources have never been so uncertain. For the aboriginal community, the plight has been recognized and community efforts are concentrating on prevention. However, according to many, the human exodus continues. Young people are leaving their villages, running away from abuse and sorrow or because they have nowhere to live, to a life they are little prepared for:

> *"The greatest need is housing on the reservation. Our people are moving because they have no homes where they came from."*

Many of the First Nations people have been subjected to lifelong indignities and still continue to suffer from festering emotional wounds inflicted when they were very young children. Just as I heard in non-native communities, the young child or young adult will self-medicate to ease the environmental pain that he/she is suffering. Native families suffering from oppression have lost their identity and consequently have low self-esteem and self-respect:

> *"The problem is everybody's problem. The reason I left home is that there's not much there. You try your best to reach your goal and you get slapped down and kicked around: 'You're good for nothing.' It really hurts ... I dropped out of school because people made fun of me ... I left home because of that and went to Vancouver and it was worse ... If you are told you are good for nothing then you believe you are good for nothing."*

Social workers advised of this group's overall reluctance to seek help. They would not seek counselling for mental health, but they would seek counselling for alcohol and drugs on occasion. I inquired of some, when the conversation seemed appropriate, why they would leave their communities for a life in the Downtown Eastside of Vancouver. They indicated to me quite clearly and explicitly that they were escaping from the various forms of abuse rampant in their own communities. One stated: "I went from the frying pan into the fire."

Family violence, and specifically sexual abuse, has many effects, including continuing the abusive cycle of alcohol and drugs. These cycles must be dealt with or the dysfunction in native communities, indeed all communities, will continue to grow. It is the responsibility of everyone in the community to intervene.

In pointing this out on a northern reserve, one professional stated:

> *"Eighty percent of the kids on the reserve are doing upwards of 50 Tylenol (plain) a day. A number of high school kids are taking 20 - 30 Tylenol a day. Some take them with wine and beer, others with water."*

Many times over, the native workers and addicts alike indicated that in addition to treatment, they must be removed from "the environment." However, when most return from a treatment centre, they return to a "no support" situation. They are back in the "frying pan":

> *"They need job training and education. That's the other part; if we are going to sober up a bunch of people, we have to have something after that in place."*

Another recovered addict I spoke to indicated that his life had been filled with violence and crime. Most of his drug-addicted years (several decades) had been spent behind bars for various escalating crimes: B & E's, auto theft, armed robbery, attempted murder, escape from prison, and dangerous use of firearms. Much of his poignant life story related to the loss of his culture and values and the subsequent violence and decay in aboriginal family life. Conditions on the reserve while he was growing up were appalling:

> *"Mother and Father were only permitted to go so far in school. In their time Native Education was still restricted. Mother was finally sent to a residential school.*
>
> *"My father and his brother ran away and finished school at a residential school. Their first house was on a reserve, but that house was later moved to another location on the reserve. This is a social wound that hasn't healed to date. They were moved so that the mining company could use the land they were living on.*

"Our neighbours were both sick alcoholics. One child died of hunger and froze to death. The children were starving all the time (it was a family of ten). Later, one of the kids died from drinking with the father."

He suffered considerable indignities at school; he sums up his experience in seventh grade:

"We learned at a young age that the books of consideration were not balanced, as far as native child versus non-native child. We were lost as far as learning any class work: always behind. I got strapped three times that year. In fall, I got roughed up by the principal and suspended and expelled from school, plus I got both barrels at home: Father and Mother. I tried to go back the following year, but never caught up from being behind: troubles at school. I left before being expelled; me and my cousins left school the same day. We put our books at the office and left."

After listening to many submissions on this issue, I can only say that if we fail to listen, we fail to stop the cycle. It is everyone's problem and everyone's solution:

"Doctors' prescriptions didn't rescue me; it was self-will and family love and friendships that really saved a wretch like I was!"

Another recovering addict was grateful he didn't stop doing drugs just to survive; he actually got a life out of it; his pleasure now was helping other addicts realize and capture that same return to life, a life clearly less painful and hopefully enriched:

"I hear kids say, 'Oh, it's too late to change for the better'; my case is a sign that it is never too late to bring good into your own life, 'cause no one can break addiction and the crime wave except the person addicted and committing crimes! ... there is always good weather after the storm."

The lack of services and trained service providers in isolated areas was pointed out time and time again. Discussion on the lack of training for service providers focused on cultural sensitivity and cross-cultural awareness. Because most mainstream services do not have aboriginal staff or utilize volunteer participation, many First Nations addicts may encounter what appears to be racism and lack of understanding, which can result in inappropriate service responses or no service response at all. Fortunately, there have recently been a number of programs aimed at rectifying this situation:

"Halfway houses are needed; they need to be manned by people that have been through the problem and understand it. I had to go over a mountain ... but, what's inside is what counts. People should have compassion and understanding, regardless of what the person looks like. I don't want to see my kids go through this."

However, funding issues remain the most pressing concern for agencies providing these services. Some of the agencies we spoke to stressed their dismay at the amount of time required to submit funding proposals and then follow them up through the bureaucratic process. Problems cited by the smaller agencies included lack of open and sincere consultation, no agency visits, excessive reporting demands, favouritism of some agencies above others, and the creation of brokerage funding processes. These perceptions of the bureaucracy may well fuel some of the attitudes and feelings of hostility, thus creating a system of care wrought with adversarial pitfalls. One administrator called it "Bureaucratic Terrorism":

"While senior and intermediate level civil servants are a necessary evil for any government, collectively, and in some cases, individually, they wield enormous power and control over small agencies such as ours. Quite often, this control is openly exhibited in ways bordering on the hostile."

The BC Aboriginal Health Council provides Alcohol and Drug Services with advice and consultation on aboriginal health care. The Council manages funding from the BC government in areas of addictions, mental health, and family violence. In partnerships with the provincial and federal governments, the

council operates, manages, and delivers health-related services and programs to assist native communities provide holistic health services. The process of apportioning funding for specific programs was criticized as being "nothing but a bureaucracy-driven process which has resulted in a brokerage type of decision-making," thus excluding many aboriginal groups from receiving adequate funding.

In Nanaimo, I heard that the local Council on Addiction had recognized that the city needed a detox centre. Despite their identification of the problem, their enthusiasm and plans, the request to the Minister of Health for funding assistance was unsuccessful. In desperation, Alcohol and Drug Services, Mental Health, Social Services, the Nanaimo Regional Hospital, the Salvation Army, and the Nanaimo First Nations pooled some money to open an underfunded, undersized Response Centre. The centre now provides emergency, non-medical detox care for alcohol and drug-dependent individuals who live in the Nanaimo Regional District. The Centre is open 24 hours a day, seven days a week, providing a vital link between the hospital and appropriate supportive recovery agencies and services in Nanaimo. This partnership evolved out of a crisis, unfortunately, and the centre was only able to open six beds because of financial constraints:

> *"Competition for acquiring money for programs is so great out there. While the committees are fighting to get dollars, there are people out there dying ... everything is program-driven and money-driven."*

Presenters expressed concern about the need to train aboriginal people to fill the service roles that need to be filled as aboriginal communities take control of their own services. One native health care representative supported ownership of the problem and the realization that there are skill shortages in the areas of education, prevention, and promotion: "We need our own people to counsel and be role models." In community after community, I heard the plea for detox programs, residential family treatment centres, and "safe houses" or transition homes for families in crisis which are culture, age, and gender-specific. Over the past 20 years, great strides have been made in the area of education for aboriginal groups. Bands, tribal councils, and urban groups have taken some control over the education of their young people. They are meeting basic educational requirements while at the same time teaching young people about their heritage. A key stepping stone for the future will be a continued and truly optimum degree of assistance for aboriginal groups in the area of education.

Some of the native services I encountered and others which were described to me reflect a great richness in culture and strengths drawn from the family and native spirituality. The traditional "medicine wheel," a fundamental element of native beliefs which incorporates psychological, spiritual, cultural, and emotional areas, is an important part of the teachings of many. I include a short description of a cross section of programs reflecting such diversity and focus. There are many more which are not mentioned here. Perhaps there is a lot that society as a whole can learn from the aboriginal concepts and historic wisdom of the elders. Some of the programs meet the needs of those who are motivated, while others strive to improve the lives of more marginalized groups.

For example, there are 21 native friendship centres in the province. I was able to visit several of these centres throughout the province. Most offer drug and alcohol counselling and take a holistic approach which would include the inter-relationship of family violence and substance abuse. One treatment centre that many spoke of is Round Lake Treatment Centre, located in the interior of the province, which provides assessment, individual and family counselling, and life skills and co-dependency programs.

Other examples include the Helping Spirit Lodge, a transition house and family violence centre, founded by and for aboriginal people in Vancouver. Its guiding principles include restoring aboriginal women to their traditionally-respected place in the family, community, and society; stopping the cycle of family violence; creating and supporting healthy families; and using traditional healing methods. Helping Spirit Lodge programs include Aboriginal Spousal Assault Program and Choices, a 16-week pre-employment program incorporating career planning and job interview skills.

Hey-way'-noqu' Healing Circle for Addictions Society offers many services that take a holistic approach to assisting urban aboriginal people in healing from addictions in a culturally relevant way. In addition to a two year out-patient program, there is a weekly "Talking Circle," special educational and therapeutic programs, and referral, follow-up, and after-care services. Hey-way'-noqu' has a long-term plan to develop a training program in sexual abuse counselling.

Tsow-Tun Le Lum Society Alcohol and Substance Abuse Treatment Centre in Lantzville is a residential recovery home designed and built by the community. Their treatment program employs a holistic approach and emphasizes native culture and tradition. In January 1992, the centre began an 18-week demonstration project for sexual offenders. The centre also provides anger management workshops and counselling which deal holistically with issues such as family violence and alcohol and drug abuse.

The SheWay Program in Vancouver is multi-agency represented and provides an integrated approach to community-based prevention. SheWay provides services to First Nations peoples, but also extends its services to all in the community. These services include, amongst others, pregnancy and nutritional counselling and various outreach programs, as described in the previous chapter. The Sheway Program is focused on "motherhood and family" and is instrumental in the prevention of FAS. This service has been operating for more than a year and, by all accounts, it is extremely successful.

In addition to programs designed by the native community, there are also those in the larger context provided for through federal and provincial venues.

The National Native Alcohol and Drug Abuse Programs (NNADAP) supports Indian and Inuit people and their reserve communities in establishing operating programs aimed at arresting and offsetting high levels of alcohol, drug, and solvent abuse. NNADAP is administered by the Medical Services Branch (MSB) of Health Canada. Projects are administered locally within First Nations communities and usually employ one to three individuals. NNADAP is basically a decentralized program, with prevention projects being delivered in First Nations communities by First Nations people. Most bands employ a community-based approach which incorporates education and crisis intervention.

Amongst the aboriginal community in downtown Vancouver there is little mobility and little desire to venture out of the familiar environment of "the Corner" (Main and Hastings). These people are subject to racial intolerance and shunned to various degrees by those who live in the area, as well as the surrounding community. Services which need to be provided to this community, which represents 37 - 40 percent of the Downtown Eastside population, must be easily accessible; that is, within walking distance. This is essentially a ghetto. Merchants, beer parlours, landlords, and various service agencies are dependent on these poverty-stricken people. Many, when asked why they don't leave or return to their home reserves, indicate that they cannot go back because the conditions on home reserves were worse than they experienced on "the Corner." Many don't want to go back, no matter how miserable their lives are in the city. They have "burned all their bridges" with home communities and families because of this.

The Vancouver Native Health Society (VNHS) provides a number of services, including a walk-in day and evening clinic. These services are open to anyone in the area. However, many of the clients served are from very marginalized groups. For example, the evening clinic is utilized by street-involved people or those who do not have medical coverage. The clinic accepts all people wishing to see a doctor, regardless of racial origin. Unfortunately, this may not bode well for the agency; they have been put on notice that their funding will be in jeopardy if it is determined that too many non-aboriginal people are accessing the service.

The clinic cites how the HIV-positive native faces double discrimination. They are often not welcome in any other environment, even downtown food banks. The much lower life expectancy of an average Downtown Eastside AIDS victim is set at approximately two years. This compares with an average of 12 years for an "uptown" AIDS case, which tells a powerful story in terms of living conditions, health care, and available services. The term "leper colony" was used on occasion to describe this unfortunate group. This indeed seems an apt description of the kind of rejection they face.

VNHS houses the SheWay program. It also provides home health care and a food bank for HIV/AIDS patients, is involved in a partnership with St. Paul's Hospital to provide in-hospital visits with patients, and jointly administers rent subsidies for people who are HIV positive or who have AIDS. In terms of education, VNHS offers street-involved aboriginal women life-skills training. This program is made possible through a contribution from the Ministry of Social Services. Future plans include housing for hard-to-house youth. An eight-unit apartment is planned.

The philosophy that "We can't change the world but maybe we can make it better" was voiced in this setting. That philosophy is fitting for this organization which provides services to a particularly challenged group of clients. I heard that the federal Indian and Northern Affairs Department could not assist them, because these First Nations peoples are no longer on reserves. It was difficult for them to

Part E
FIRST NATIONS

understand this. It was also stated that when the VNHS approached native bands for assistance, they were not really interested in helping. A "leper colony"? Indeed.

After listening to, and reading many submissions, I was left with the impression that it has all been said and written before. However, I repeat here the essence of what people reflected concerning aboriginal issues in their respective communities. I do this to emphasize that if both federal and provincial levels of government are serious about assisting First Nations people, they must continue to heed the calls and ameliorate the situation for many of the First Nations people.

I therefore RECOMMEND THAT the Ministries of Aboriginal Affairs and Social Services:

43. Continue and expand multi-cultural education and training to service providers.

I therefore RECOMMEND THAT the Ministry of Aboriginal Affairs:

44. Review present outreach services and support for native people who are substance abusers;
45. Review assistance provided to native detox, recovery, and treatment centres, with a view to assisting in the development of band and local responses in addressing alcohol and drug issues in the respective locales;
46. Review the need for and provision of assistance to native youth drop-in centres and community-based clinics which offer alcohol and drug services, counselling, and education;
47. Invite native people, including recovering substance abusers, to participate more fully in the planning of regional and local services and programs.

Part F
OTHER DISADVANTAGED GROUPS

"What is needed is hope ... most of them are acutely aware of the dangers but choose to ignore them because they don't have any hope. They need a stake in our society; they have no prospects and they don't care."

Another specific group of people which may be at a disadvantage in the current system are those with a dual diagnosis: alcohol or other drug dependency issues, along with a psychiatric diagnosis. In Alcohol and Drug and Mental Health terms, dual diagnosis is restricted to mean the presence of addiction with psychiatric problems. In meetings with service providers, outreach nurses report more mental health clients are using illicit drugs than in the past. Most of the references to dual diagnosis patients were from the downtown area of Vancouver. However, this is an extremely mobile population who have been attracted to the downtown core because of the low rents, services, and the personal attachments formed. A recent assessment of mental health needs for the area reported that the downtown core has a high concentration of individuals with mental health and behaviour problems, with approximately 200 seriously mentally ill in the downtown core who are not receiving mental health services. The report recommended directions for services which would benefit this clientele.

At the public meeting at Carnegie Community Centre in Vancouver, several people spoke about the work that had been done in this community to identify the needs of special interest groups in the Downtown Eastside and Strathcona areas. Several focus groups (including dual diagnosis clients) identified barriers to accessing programs; most notably, some had been refused entry into services such as detox because of their behaviour and alcohol or drug abuse history. Another barrier cited was the stigma and shame associated with the label *dual diagnosis* and the fear of "being made fun of." Members of this focus group did not trust existing programs and agencies;

rather,they felt alienated from and fearful of them. It was their consensus that there is no alcohol and drug facility and a considerable lack of services for this group in the Downtown Eastside and Strathcona areas.

Clearly, there still remain barriers to access for this disenfranchised group of people. The Director of the Dual Diagnosis project sums it up in his 1992 report:

> *"There are many alcohol and drug programs already existing in the downtown core and certainly a significant number of this population have a serious alcohol and drug problem. Sometimes, however, they have difficulty accessing these programs, as many of the staff in them are not familiar with the seriously mentally ill and the problems they present. In the last three years we have initiated educational workshops and staff exchanges between Greater Vancouver Mental Health Society (GVMHS) and Alcohol and Drug Programs. More needs to occur and, if it turns out that Alcohol and Drug Programs cannot accommodate the seriously mentally ill, then a separate facility should be considered."*

One of the major issues facing this population is their distrust and fear: fear of agencies, fear of legal and financial obligations, fear of losing children, and fear of other people's attitudes. A number of people I spoke to stressed that people with serious mental illness, and especially those afflicted with dual diagnosis, are easily victimized and often robbed and coerced into taking drugs. Contributing to this picture are the underlying social issues, such as institutional down-sizing and the lack of subsequent follow-up for these patients, as well as inadequate housing and living conditions.

While there have been great strides made recently in identifying the needs and providing relevant programming, the Ministry of Health should continue to fund and expand initiatives in this area. As the previous studies have pointed out, there is a pressing need for the continued priority of its housing program, improved and better-funded drop-in centres, and continued training for service providers in all areas of alcohol and drug treatment. Police, general practitioners, social workers, psychologists, and public health workers need continuing education in this area. Clearly, the community should also be aware of and support projects which provide safe environments or refuge. As noted by a service provider:

> *"The difficulty remains as to how to motivate these people to obtain treatment. It should be noted, however, that the need or desire to socialize is quite high and if there were alternatives to the bars and beer parlours such as drop-ins already mentioned, the consumption of these substances would likely be reduced significantly."*

Another smaller and all-but-forgotten group are the trans-gendered (usually a person born as a male but who is emotionally and psychologically female). These people are often confused with or generically mixed in with transvestites. Many of them may be illiterate, homeless, malnourished, and victimized. Survival and growth of safe houses and drop-in centres is tenuous. Indeed, as one service provider lamented:

> *"Funding has been almost impossible to obtain since government has difficulty deciding which department may have responsibility and private foundations seem loath to be identified with trans-gendered people."*

There were also multi-cultural concerns which surfaced during my consultation with service providers and community groups. These concerns included lack of understanding of different cultural views of addiction and treatment, inability of counsellors to serve clients in their language, insufficient cultural sensitivity on the part of mainstream counsellors and workers, and lack of substance abuse expertise amongst ethno-cultural groups and serving agencies. The most commonly mentioned sub-groups were Vietnamese, Indo-Canadian, and Hispanics. Again, perceptions and attitudes create barriers for access to treatment and support.

One of the things that many people suggested was improved emergency response through partnerships between service providers. The challenge in each community is how to deliver a cost effective, efficient service. Such collaborations make more efficient use of available resources and encourage a team approach to resolving crises. In many communities, this is beginning to happen with liaison programs between hospitals and mental health

centres and collateral agreements which extend beyond these to include police and other agencies involved in services to those in crisis.

I therefore RECOMMEND THAT the Ministry of Health:

48. Prior to release from mental health facilities, provide people with, or assist them in securing housing which will ensure the necessities of life and community support.

Chapter 5
THE JUSTICE SYSTEM

Part A
POLICE

"It's not so much an enforcement issue as it is a larger social issue ... If enforcement worked, the US would be drug-free."

In the early eighties, police intelligence and analyses reported that the Southeast Asian countries of Laos, Burma, and Cambodia (known as the "Golden Triangle") remained the traditional sources of heroin for British Columbia, albeit not discounting the international supplier of heroin comprised of Iran, Afghanistan, and Pakistan (known as the "Golden Crescent"). While intended largely for the European market, the Golden Crescent supply created opportunities for French, Italian, and Sicilian organizations to expand their markets on the eastern seaboard of the United States. There was some evidence of occasional shipments of this product reaching British Columbia from New York.

Police intelligence also suggested that in the seventies the drug of choice was cocaine and the heroin problem in British Columbia was in decline. In the early eighties, this problem threatened to grow again. Externally, there was a world surplus and internally, there was a growing "recreational" market and change in trafficking patterns.

In the late eighties and early nineties, there evolved a dramatic change in trafficking of cocaine and heroin in this province: to wit, an increase in volumes and a decrease in prices. The one major reason was the arrival and presence of the predominantly Asian (Chinese and Vietnamese) trafficker. The numbers of traffickers increased; new dealers were (and still are) a daily event for the police.

The reason for the drop in price is easily explained: supply and competition. There was an over-abundant supply of heroin and cocaine. To capture the market, considerable competition developed amongst traffickers. Many small groups or organizations existed for the sole purpose of selling drugs. Traffickers come from many diverse backgrounds. Some have been forced into trafficking to support their addiction. For others, one shipment can financially set up an immigrant family or young Canadian for life. This may be why the police are seeing younger people, as well as immigrants (tourist "mules"), emerging more frequently.

It is common for Chinese and Vietnamese traffickers to sell both cocaine and heroin. It is strictly business; many are not users of these drugs. Cellular phones and pagers allow for easy contact and instant delivery of the drugs. For those traffickers who speak little or no English, they refer to cocaine and heroin as "up" and "down" respectively. With contacts in Hong Kong and Vietnam, supplies are easily arranged and imported by land, sea, and air. The latest advice is that the cocaine cartels in South America are now experimenting with growing the opium poppy in an effort to break into the North American market.

In the seventies and eighties, the heroin user community was a fairly tight-knit group, with new faces not readily received. Police undercover methods were not always productive. Today's traffickers use pagers and cellular phones. When I asked an addict just how readily available a drug was, he asked what I wanted and said he could have it delivered to my office within 15 minutes. Times have changed.

Furthermore, in the seventies and eighties, heroin seizures were analyzed at three to five percent purity. Pure ounces were not commonly for sale on the street. In the nineties, heroin appears to be rarely cut with any diluent. It is imported in a pure state (80 percent and above) and distributed, even at the user

level, in the same form. Supply is plentiful; competition is keen. One ounce of that pure heroin can be purchased for $ 2,000. In the seventies and eighties, that ounce would have been cut four or five times and each cut would have sold for $ 15,000-$20,000 (i.e. $60,000 to $80,000 for the original ounce). Yesterday it was $ 80,000; today -it is $ 2,000. That incredible selling price makes it affordable to virtually anyone. The addict who spent $35.00 for a cap of three to five percent in the seventies and eighties can now obtain 40 caps at five percent for the same price.

Police suggest that the number of traffickers and users is increasing on a daily basis. With the addict population growth, new markets being sought by traffickers, the high purity of the drug, and the relative ease of purchase, an increase in overdoses and sudden deaths should not unduly surprise us.

What then are the police saying as I travel the province and listen to their experiences and thoughts on the problem?

> *"While there are still small groups of the old users, the next generation is now involved. The Vietnamese influence is drastic; they are seeking out 14-15 year-old kids to smoke heroin in the day time. These traffickers are generally not users; it's a hard community to infiltrate. Canadians are unaware of who these refugees are; many made up names on their way into the country. They have violent backgrounds and they are violent: the courts and jail are a joke in their minds. They take advantage of women, both Vietnamese and Caucasian, using them as carriers and slaves."*

> *"The police are not even keeping their heads above water. They face the same people over and over again. We may need to dispense with the enforcement programs that are not working. We must quit burying our heads in the sand. The problem is there, but not acknowledged for one reason or another."*

Referring more specifically to addicted people, one police officer believed that "locking them up and throwing away the key" is not necessarily the answer. He likened heroin addiction to "a merry-go-round, a revolving door." Even when help was readily available and the addict took advantage of that opportunity, he still eventually returned to his old friends and haunts, only to relapse. Education at the junior high school level and educating teachers and parents alike were seen as early steps in preventing the spread of this "disease," as some termed it. Heavy enforcement and penalties for traffickers and education and treatment for non-users and addicts was the formula proposed by some. Conversely, one police officer characterized it as: "Three strikes and you're out - lock 'em up and throw away the key."

It is well recognized by law enforcement agencies that a good proportion of chronic drug users commit criminal acts to support their drug habits. The majority of addicts they deal with lack the social skills, education, and type of employment necessary to obtain sufficient money through legitimate means. Police estimate that 60 percent of non-drug crime in British Columbia is motivated by illicit drug use, or committed in the course of illicit drug activity.

In distinguishing between heroin and cocaine users, one officer cited the latter as generally "high achievers, educated professionals who can afford the drug and who use cocaine in the morning to achieve, and heroin at night to unwind." He went on to observe that heroin-only users tend to be "down-and-outers, lower class, downtown skid road types." He spoke also of South American cocaine flooding onto the market as the more socially acceptable drug.

There appeared to be an overall sense of frustration and anger on the part of law enforcement officials, which was directed toward most, if not all, agencies outside their own: the courts, legislators, and bureaucracies. Immigration was a high target, both in terms of admissions and deportations. Social Services and legal aid were targeted as well. Police officers called for greater inter-agency cooperation and exchange of information, albeit recognizing the constraints imposed by protection of privacy legislation, as well as administrative policy directives.

In the same breath, officers conceded that what they encountered and what surfaced in the public eye provided but a small glimpse of the actual problems society faced. The drug problem was alluded to as the "tip of the iceberg," and as such, it was conceded that this is not so much an enforcement issue as it is a larger social issue. Hence, some are pointing now in the direction of education, treatment, and cooperation. Indeed, a ray of light appeared to beam on the broader public interest, as opposed to the narrow enforcement focus of arrest and incarceration, a focus devoid of hope. The futile merry-go-round, the "revolving

door" of arrest, detention, parole, and relapse, with all its consequent costs, over and over again, is reluctantly recognized with dismay.

Measured in purely financial terms, there are ironically winners. Many obtain their livelihoods out of the various systems designed to deal with these substance abuse problems. It is indeed an industry. On the other hand, someone must pay the bills; we all know who that is. Measured in terms of human costs, the expense is horrendous, ending in violence and death. The reputed "war" in this field is perhaps better characterized as decades of skirmishes, with frankly "no end in sight." Perhaps some form of truce might best be called for.

One aspect of the drug problem which received considerable comment from both law enforcement officials and addicts, particularly recovering addicts, was the area of trafficking and immigration. I related earlier the sources for the importation of heroin and cocaine. The obvious extensions of the producers and distributers of these illicit drugs are the couriers and traffickers, most of whom do it for profit, not personal use. And let us not forget the "backers," unseen but not unclean. What has happened more recently is that not only is the product of Chinese and Vietnamese origin. So too are the traffickers who find their way into Canada through both legitimate and illegitimate means. The intelligence indicates that some offshore refugees, of unknown backgrounds save for race, find their way to British Columbia and, with previous connections in their homeland, not only obtain and import these drugs, but establish themselves and others in the local distribution of them. In the same fashion, intelligence indicates South and North American immigrants, some of whom are refugees, have adopted the same course, with homeland connections. At the moment, it is cocaine for them; in the future, it will be heroin.

Lest anyone raise the accusation of racism to distract from the issues here, it should be clearly understood that law abiding South and North Americans, Asians and other immigrants generally reject these activities. Lawbreaking reflects adversely on them as visible minorities. Furthermore, recovering addicts who experienced the addiction were fairly specific at directing their remarks only to those who "were not users, who were dealing drugs for profit and greed only, and who didn't give a damn who they dealt to, even young kids." The authorities (police, medical, and social) who shared their experiences and views on this matter were of the same mind. Their concern was how to stop the damage that was being inflicted on users, their families and the public. They were not interested in getting embroiled in racism.

An example was presented of a young Vietnamese immigrant, reportedly a refugee, who had been charged three times with possession for the purpose of trafficking, released on bail three times, and was receiving legal aid and social assistance. This same trafficker drove a late model car and reportedly "had $30,000 in his back pocket." Anecdotal? Partly. A recovering addict did not have a great deal of sympathy or affection for that type of trafficker. He did not believe a long jail term and deportation was the answer. Rather, he suggested "execution and then deportation." The police expressed a frustrated dismay and disgust. And the public?

The point being made throughout by all contributors was that race, colour, creed, or ethnic background is not the issue when it comes to the importation and distribution of illicit substances. The issue is the product and the person. Neither had a right to be present in British Columbia, and if there were no right, then there was a duty on the part of the authorities to ensure removal.

There is a common public perception that the drug problem in British Columbia is a law enforcement problem for the police and the courts. This is quite natural, for the public is of the view that discouraging the rise of drugs and solving the problem can be achieved through enforcement: punishment and fear. However, the failure of this approach is equally apparent. Harm to the community and user alike has not been reduced, perhaps because the goal is punishment, not harm reduction. In some quarters, this would then call for more police, more courts, more jails, and harsher penalties. In other quarters, this would call for a re-examination of policies and practises, a shift to some other model or models to deal with the issues. While a portion of the drug problem will remain a law enforcement problem, the time has surely arrived for society to re-examine, re-define, and clarify the balance between public safety and harm reduction.

One day, society may come to acknowledge that drug users generally don't fit into the enforcement or criminalization model. The two theories used in that model are:

(a) incapacitation - "Lock them up and, while they are in jail, they cannot use/deal drugs."
(b) deterrence - "The threat of punishment will persuade them both generally and specifically against using/dealing drugs."

Part A POLICE

The "revolving door" experience in arrests, courts, jail, and parole shows us clearly that neither (a) nor (b) has any effect on drugs users, for the most part. When I asked addicts their reasons for stopping the use of drugs, few, if any, gave the threat of punishment and incarceration as the reason.

Drugs are readily available in jails; some addicts quit in jail, others begin in jail. One thing is for sure; the environment in jail lends itself more to the criminal mentality than to recovery, restoration, and reintegration into a normal personal and social life style. Indeed, I would suggest that our prisons and penitentiaries encourage personal connections for the drug community when they return to the street; in effect, they provide a schooling for those connections, an "old boys network." And lest we forget, those heavily-addicted people have pretty tough lives to lead on the street, surviving day-to-day, with violence around every corner, homeless, ill, and with the threat of death attached to every "fix." So really, jail can be more of a relief than an incapacitation or deterrent.

Clearly, there is a requirement for continuing police involvement in the substance abuse problem here in British Columbia. The fundamental question that will always be present in such a dynamic field is: "What is the most efficient, effective way to provide that service in the interests of public safety and individual well being?"

I therefore RECOMMEND THAT the Ministry of Attorney General:

49. Enter into discussions with the federal Minister of Immigration respecting the need to review and determine the status of non-Canadians who import and traffic in large quantities of narcotics;

50. Explore the feasibility of entering into discussions with the federal Minister of Justice and the Solicitor General of Canada, with a view to applying revenue obtained through the Proceeds of Crime legislation in British Columbia to the cost of prevention, treatment, and recovery programs here in the province.

Part B COURTS

"The courts should distinguish between users and pushers ... they have made enforcement virtually impossible."

A sense of frustration and anger on the part of law enforcement officials toward the courts was noted earlier. In many instances, that feeling stemmed from the initial arrest and charging of the accused trafficker. The reference point in many instances was detention vs. bail.

A police officer indicated how enforcement techniques had been recently targeting primarily traffickers as opposed to users, but even with that, he expressed frustration with the process thereafter:

> *"Traffickers are being charged and held for court. They are subsequently released the next day with court orders. They are then quickly encountered again trafficking on the streets. In talking to these people, the courts are merely a hinderance and referred to as a 'cost of doing business.' These people know that it will not cost them anything, as a lawyer is provided through legal aid and it is very unlikely that they will spend any time in jail."*

The frustration alluded to does not go unnoticed by the general public either. However, it is the law — or is it the application of the law, or a combination of the two?

The police see the trafficker making bail and going back into business within 24 hours of initial arrest. The fact that taxpayers supply legal aid lawyers for offenders in this category is particularly difficult for police and the public to grasp. This becomes clear in a striking manner when an accused comes before the court on multiple charges arising while on bail for previous charges.

When we consider that an offender's ability to pay is taken into account in the qualification formula for legal aid, vis-a-vis the knowledge that significant

profits are being generated in the sale of drugs, it seems, after one or more convictions for drug-related offenses, particularly trafficking by a non-user, that provision of further legal aid should be the subject of very close scrutiny.

The annual estimated cost of the provincial legal aid program in British Columbia is approximately $86 million and was viewed as a growth industry for lawyers and law firms. This is not to suggest that legal aid is not a worthwhile program; rather, its application to offenders with previous convictions on drug trafficking charges is viewed by many as making a mockery of this program. It was also felt that the taxpayer was indirectly supporting drug traffickers, ensuring profits for them and the lawyers who assisted them. A fair and reasonable rule, based on reality, was asked for.

It is interesting and instructive how British Columbia courts view and apply the law in regards to sentencing after conviction. I think it would be helpful for the general public to know this, hopefully gaining a greater understanding of the process and rationale in sentencing offenders.

Drug offenders, like all offenders, are subject to four guiding principles of sentencing following conviction. They are:

(a) rehabilitation;
(b) protection of the public;
(c) general deterrence;
(d) specific deterrence.

The courts do not recognize punishment as a guiding principle of sentencing.

In cases where there are convictions for trafficking or possession for the purpose of trafficking, the courts in British Columbia distinguish between so-called "soft" and "hard" drugs. In the case of "soft" drugs, the sentences imposed are generally under two years, unless a large scale operation is shown or the circumstances are unusual. The courts are saying a jail sentence should be imposed in most of these cases. In the case of hard drugs, the courts find it a duty to deter people from both using and trafficking in them. In these cases, judges are guided by the principles of general and specific deterrence, calling for imprisonment for more than two years.

Interestingly, where the courts are presented with a particularly severe public problem (e.g. heroin), there is a sense that heavier sentences ought to be prescribed. Also significantly, where an addict appears for sentencing, the importance of rehabilitation comes into play. Sentencing is an individualized process; the courts' application of these guiding principles is dependent upon the facts of each case. The Provincial and Supreme Courts in British Columbia are also guided by previous sentencing decisions on similar factual situations. There have been two recent cases of cocaine and heroin trafficking where the Supreme Court justices recognized a severe public problem and imposed heavy sentences. There is also a case in Provincial Court where the judge wondered aloud whether sentencing drug dealers to a large number of years in jail would, in fact, deter others from getting involved in the sale or distribution of large quantities of drugs.

The British Columbia Court of Appeal has not imposed the maximum sentence of life imprisonment since 1984, holding the maximum sentence should be reserved for the worst case scenario. When compared with the Alberta and Ontario Appellate Courts, the overall drug sentences in British Columbia appear less severe.

The conclusions I draw from all of this is that the police perspective is probably correct insofar as jail terms are concerned. The Provincial Court judge who wonders at the merits of heavy sentences is probably correct too and the Supreme Court justices who impose lighter sentences are probably sound in that position. Ironically, they are all correct, for jail appears to be neither a general deterrent nor a specific deterrent within the corrections system. The only principle remaining is "protection of the public," and I am frankly unable to find any substantive evidence in support of that. British Columbia has more heroin and cocaine, more traffickers selling at bargain basement prices, more addicts, and more deaths. So, whatever we are doing in this province vis-a-vis the criminalization model, it is not working. More police, more courts, more jails? More "revolving doors"? Is this a formula for reducing harm to the community, the addict, and others?

I therefore RECOMMEND THAT the Ministry of Attorney General:

51. Examine and discuss with the federal Minister of Justice and the Solicitor General of Canada the merits of mandatory maximum life sentences, without parole, for people importing and trafficking in large quantities of narcotic substances.

Part C
CORRECTIONS

"Jail is not the best social institution to deal with our social problems."

During visits to institutions I spoke with both line and staff personnel, as well as inmates who, for the most part, were serving time for offenses related to their substance abuse problems.

Opposing views were heard: "Jail saved my life! I was on a self-destructive road and jail allowed me to clean up and take a new look." "Jail! I didn't get started on drugs until I went to jail ... there were more drugs in jail than on the street." One written account perhaps summarizes the motivation: "Every day that you're high in prison is a day you're not doing time."

Managing the problems of drug importation and consumption in correctional centres is a major concern. A few short years ago the focus of internal correctional security was in attempting to prevent the smuggling and production of alcohol. Search, seizure, and prevention of the production of contraband "brew" accounted for a good portion of correctional officers' attention. That has changed. Now there is a concentration on preventing the inflow of contraband drugs. Both federal and provincial corrections administrations are looking at the two potential "choke points" of dealing with the problem of drugs in jail: interdiction (preventing drugs from getting in) and detecting the distribution once in jail.

In first examining the matter of importation, I was advised that it is virtually impossible to prevent drugs from coming into correctional facilities, no matter what the security classification, whether it is a federal or provincial institution, for males or females, for adults or youth. The methods are innumerable, often ingenious, but usually stemming from visits, returning prisoners "suitcasing" drugs in body cavities, staff or contractors "packing in," and perimeter drops. Often threats of violence, debt collection, and various forms of persuasion are employed to pressure weaker inmates, their family, or their friends into taking part in moving contraband drugs into institutions.

One correctional centre reported its vertical tower configuration with outdoor patios provided convenient landing sites for tennis balls filled with drugs or frisbees with drugs taped to the bottom. In one case a bike rider on the move was observed throwing a ball over his shoulder to hit a patio exercise area, without even slowing down. Installing additional wire screening over the patios was believed to be successful. However, smaller projectiles may well be deployed to breach this barrier. These scenarios are repeated at virtually every centre.

In the case of correctional facilities with reduced security, outside work gangs are in a position to pick up "drops" or "throw ins" from passing vehicles. Usually only short-term inmates, or those who are trusted by virtue of having minimal records, are classified to work gangs. However, this presents no problem to the inside traffickers. Pressure is applied to these inmates to cooperate, retrieve, and deliver the drug drops. Most often, these same "mules" will take the punishment for handling the drugs if caught, rather than face the more punitive action of their fellow prisoners for disclosure of drug trafficking operations or ringleaders' names.

There are ways and means of introducing drugs into correctional facilities. There are also a variety of motivations, with significant amounts of money to be made for those who are willing to take the risks. I have raised the matter of coercion and intimidation: threats against the individual prisoner or his family to cooperate in the smuggling or distribution operations. One correctional centre supervisor notes the management problem created by the inmate drug trade:

> *"We get an awful lot of assaults over drugs. Inmates go to drug awareness programs set up for people on the street. They can't get away from drugs in there and if you're not a user you're not trusted. Guys get forced into the situation, come off the street clean and they get forced to use."*

In examining the methods of direct importation by inmates and visitors, there are a number of considerations. With the emphasis on rights and freedoms, intrusive searches of prisoners and their visitors can only be considered when there are "reasonable and probable grounds to believe" that drugs are being

carried. The court tests of this rule indicate that there must be more than a rumour or a tip; rather, there must be some fairly substantive grounds, in order to justify ordering the search of a prisoner.

In the case of visitors, the same rule applies, with the important proviso that, on entering the institution, the visitor is asked to submit to a search if wishing to proceed with the visit. If the person agrees to submit to the search, a signed acknowledgement of this agreement is obtained. If drugs are found on the visitor, the police are called; technically, corrections officials cannot detain the individual. If the visitor refuses the search, the visit may simply be cancelled and the person will leave. They may also be removed from the inmate's visits list for a period of time.

Complaints concerning searches being employed as a method of harassing inmates and visitors are not infrequent in federal and provincial corrections facilities; this tends to keep staff on the defensive. Correctional officers are reportedly cautious, given the perceived "flack" they can incur from the prisoners' legal advocates, the Ombudsman, and correctional investigators.

Staff must often rely on inmate informants to intercept drugs coming in, but this has its hazards for all concerned as well. One official put it this way:

> *"It's a constant game. You never know if it's good information or a 'set up' ... a test to see how quickly you respond or a possible diversion.*
>
> *"In some cases, the person bringing it in is as much a victim as a perpetrator. Visitors are often under threat or fear and feel they have to bring the drugs in. Prisoners who are non-users are forced to have their visitors bring the drugs in, thus avoiding close scrutiny."*

I understand from corrections personnel that there are increasingly few disincentives for the importation and use of drugs in correctional centres. In a recent case in northern BC, an inmate was caught with a load of drugs in the adult facility. He pleaded guilty in Provincial Court to possession, and upon sentencing, was given an absolute discharge, effectively neutralizing the disincentive to deal drugs in jail. This situation suggested to the corrections administration that they are wasting their time and the courts' time in pursuing these cases.

It was observed that the increase of inmate rights and freedoms has brought about a situation that cannot be controlled by the authorities anymore. Full access to phones has meant that drug deals and drops may be arranged with ease and avoidance of detection. That same free access to phones has seen an escalation in the interference with witnesses, the planning of escapes, and the arrangement of drug deals. "These measures are not in society's best interests," a provincial prison administrator told me.

At another provincial correctional centre, I learned there had been a number of close calls with overdose incidents. Fortunately, no one has thus far died from an overdose in the provincial system. Officials are concerned that the odds are not good for this success to continue. Correctional Service Canada (CSC) has recently lost inmates to drug overdoses, including a double overdose incident involving a visitor who is believed to have brought drugs in. Federal authorities were concerned that the combination of valium and heroin can be quite lethal in that the valium may block the effect of Narcan. One federal warden noted that an inmate recently died in his arms even after the administration of three "hits" of Narcan.

Some prison administrators volunteered that sometimes drug use can be helpful in "keeping the lid on." One senior provincial official told me:

> *"You are not going to eliminate drugs, just control them. It's easier to get drugs in jail than on the outside. They have no reason to quit in jail. The drugs are not so much the problem; its the economy and the concern of mixing drugs, as well as keeping non-users from being victimized."*

In realistically recognizing the drug usage problem in jails and in attempting to minimize the harm (particularly the potential harm of HIV transmission), correctional services, both provincial and federal, have taken steps to at least minimize the risk to health. The BC Corrections Branch has implemented a policy which makes bleach available to inmates for the cleaning of needles (which remain technically contraband), hopefully making the practise of intravenous drug use in prison safer from a health standpoint.

Put very simply, this is a program of harm reduction. CSC recently completed a study by the Executive Committee on AIDS in Prison (ECAP). There are a number of recommendations to address the problem.

Part C CORRECTIONS

Theoretically, the institutional setting should be the ideal treatment facility. There is control, access to counselling, and time to reflect and plan for the future without the normal or extraordinary pressures of "life in the fast lane," so often the case for those on the street. Again: "Jail allowed me to clean up."

I repeatedly heard that substance abuse services are lacking in correctional centres. The Burnaby Correctional Centre for Women has one AA meeting each week. An alcohol and drug counsellor also visits, but cannot possibly deal with the volume of clients. There are no release planning facilitators available to assist with arrangements for those women returning to the street without resources. It is little wonder that they gravitate back into the depressed areas and return to a life of substance abuse. There is little planning ahead and no follow-up unless parole or probation follows jail. An identical story was related by a senior correctional official in an adult male centre. The question arises: "Is it the responsibility of the correctional services to provide for this after care?" With no positive bridge between the institution and the street there is little hope:

> *"The guy who gets out without resources ends up back in the pub scene ... and then its just a matter of time before he is back in trouble, certainly back abusing alcohol, drugs, or both."*

With respect to assessing community methadone clinics for released inmates, we heard of the ironic "Catch 22" that former addicts face when they attempt to get on Methadone Maintenance Programs (MMP's)immediately following release from prison. It seems that the rule is that you have to use heroin before you can get on: "You need three positive urine tests" before being screened for inclusion, "but I quit using ... I don't want to go back to it." Even though these people may not want to return to illicit drugs and are looking to methadone in order to avoid the drug scene, they tell me they end up being forced to take heroin initially to obtain treatment. One person asked if it would be possible to look at setting up a methadone referral prior to release. He explained that when one's system is clean, if you are having to use street drugs to get into a program, you might die in the process. It was a question of reduced tolerance.

Another respondent noted that she was on a six month MMP when sentenced to jail. She had to wean off of it and found this worse than quitting heroin. A few agreed that they could be connected to street clinics on discharge, which would reduce the harm to them and perhaps help them avoid returning to the illicit drug scene. This idea was also recommended for consideration by inmates I met at a federal centre.

Another position presented was that MMP's in institutions could assist inmates who are in withdrawal from street drugs or community MMP's. One inmate indicated that she had eventually completed withdrawal from methadone and had "got nothing — no other withdrawal medication — for it ... I've seen girls going into the system who had been on methadone, and they couldn't get out of bed for three months." Anecdotal exaggeration? Corrections officials advise me it is unthinkable that an inmate would not receive medical attention in those circumstances. I relate it here simply to show the inmate's perception of the pain associated with withdrawal from methadone. The point also being made was that the street MMP is a government harm reduction program based on chemical dependency, yet there is no recognition of the continuing need for methadone treatment once the person enters into the correctional system.

In a controlled setting such as a correctional institution, a methadone maintenance clinic program would help offset the illicit drug trafficking inside. It might also provide testing stabilization and counselling to assist the inmate for release. Actually, an officially sanctioned medical program in correctional centres might well be consistent with the community programs. It is the next logical step to supplying bleach solution to promote harm reduction amongst IV drug users in jail.

On the other hand, I heard of decisions on the part of inmates who had been substance abusers to stay clean once they got out, regardless of the circumstances:

> *"Jail helped me sober up ... I had used and cleaned up in jail. I got it totally out of my life. I began to look spiritually ... and when I got out my brother who I was close to died. I stayed off ... got a job drug counselling for 14 years. Then over a three year span I began to slide ... first alcohol, then pot, then back slowly to heroin. I went through detox three or four times."*

This inmate argued that medical personnel do not understand the addiction process or the impact of withdrawal. He told of not being able to sleep for

three or four days during withdrawal, and of the intolerance of medical personnel. He acknowledged that addicts manipulate and lie for medications, but he appealed for understanding. Another inmate related:

"I've lost a lot of friends and family ... some to overdose, some to suicide on cocaine. A percentage of deaths are accidental ... coke on top of heroin."

In speaking with several older addicts I was afforded the classic picture of the "revolving door" with corrections and addiction. At age 62, one spoke of his working life on heroin, his industrial injury, his escalating heroin addiction, crime to support his habit, and the cycle of prison and release: the waste of time and money. He called for a legitimate option, methadone clinics. He spoke for legalized drugs (not free drugs) in order to break the cycle.

In viewing the interrelationship of substance abuse and corrections, I should mention the close link between FAS, FAE, and NAS and adolescent delinquency. Some authorities postulate a linking between FAS, FAE, and NAS syndromes and juvenile recidivism. The results of two studies have tied these syndromes to corrections. In 1974, Bergman found a large proportion (50 percent) of juvenile offenders displaying learning disabilities and a BC study by Waldie and Spreen (1993) showed an increased susceptibility to delinquency of youths with learning disabilities. It appears that there is indeed a link to the characteristics of adolescents and young adults with FAS, FAE, or NAS who frequently suffer both language-based and social learning disabilities.

At a recent conference held in Vancouver, it was suggested that FAS youths in the justice system have poor reception of social cues, poor ability to learn from experience, and general impulsiveness consistent with certain types of learning disabilities. Limited language comprehension, poor memory, and lack of understanding all mitigate against such youths throughout the criminal justice system:

"The way the youth presents himself/herself socially (i.e. apparently lacking in remorse or not appreciating the seriousness of the crime) may also result in a less favourable disposition."

"Incarceration may not serve either to deter future crime or to allow for rehabilitation but may, in fact, worsen the situation, due to the victimization and inappropriate modelling that can occur in the prison environment."

The implications for corrections are manyfold. First, one might expect a greater influx of clients into youth and adult corrections in both community and institutional services. The continued prevalence of alcohol and polydrug involvement by pregnant women will tragically ensure greater numbers of youth finding themselves learning disabled and susceptible to delinquent involvement.

The fact that this greater influx of FAS/FAE youth will not likely benefit from correctional contact, particularly in the institutional setting, does not auger well for society, the corrections system, or the individual. It is clear that alternatives to treatment of FAS/FAE, youths must be developed and must extend beyond the correctional setting.

At the same conference, a Supreme Court justice indicated that children with learning disabilities of every variety account for a large portion of the intake in the juvenile system. He noted: "In the adult system, by far, most crime is alcohol or drug-related, either directly or indirectly." He called for more community support systems to deal with these problems and the continued interest of community groups to assist with this issue.

In summary, the area of corrections holds many significant problems, yet perhaps some promise. Clearly the various correctional systems do not currently possess the answers or the ideal treatment programs to address substance abuse or drug problems: not for society, not for the corrections system, and not for the individual. The institutions are full of individuals who have been involved mainly as the consumers, and who are serving sentences for crimes related to obtaining drugs or for crimes committed while under the influence of drugs. Reduced internal restraint and diminished responsibility impact on the individual, the courts, the correctional system, and society as a whole. We must begin to see "those individuals at the bottom of the drug chain as victims of society rather than as criminals." Where possible and, depending on the risk to society in terms of collateral criminal offense patterns, we must look at establishing model medical treatment, social engineering, and assistance, rather than criminalization, punishment, and incarceration. Offenders involved in major drug dealing or trafficking operations would be ineligible for such programs and should be incarcerated in prison facilities separate from treatment facilities.

Part C CORRECTIONS

For those offenders who are addicted and living lives of crime to support their habits, there may be alternatives, depending on the drugs involved. MMP's operated in treatment centres or jails could assist individuals in stabilizing their lives and preparing for adapting to new and productive activities on the street. Comprehensive treatment programs for select sentenced inmates, ideally operated separate and apart from correctional centres, could utilize new directions in dealing with substance abuse, focusing on a range of treatment paradigms from abstinence to withdrawal to controlled maintenance, depending on the problem and the motivation of the offender. I am convinced that experimental drug treatment — supplying prescribed medications to offenders — would go a long way in neutralizing the drug trade and its adverse affects in our correctional institutions. We are halfway there when we supply bleach solution for inmate needle users, thus acknowledging the existence of the problem and the need for harm reduction. Again, lift the veil of denial and directly tackle harm reduction in jails by working realistically with those inmates who have the addictions. Some will benefit, some will not; the key will be careful assessment for inclusion in treatment programs with possible use of controlled medication where appropriate. It does not make sense that MMP's are operational in the community but are not allowed when addicts are under sentence and are subsequently required to provide "dirty" urine samples to get back on community programs following release.

Incentives for involvement in these specialized treatment programs should include the following: consideration for transfer to reduced security, early release, parole, work programs, employment training, liberal temporary absence programs, involvement of family in treatment, conjugal visits, or conjugal units on site. Inmates involved would necessarily agree to follow the program and agree to urinalysis as part of assuring compliance with program demands. A major degree of client-directed activity in the final stages would assist in promoting personal responsibility.

By providing these treatment options in special correctional or community treatment centres, there should be a reduction in the current overcrowding of jails, particularly at the level of provincial corrections.

I therefore RECOMMEND THAT the Ministry of Attorney General:

52. Establish mandatory alcohol and drug educational programs for young offenders, both institutional and community-based;

53. Provide an alcohol and drug detox and treatment program for young offenders, both institutional and community-based;

54. Designate one or more separate facilities within the adult correctional system as detox and treatment centres;

55. In collaboration with the Ministry of Health, provide an assessment, detox, treatment, and counselling program at that/those specific centre(s);

56. Where feasible, extend the educational, treatment, and counselling aspects of those programs to parents and guardians of offenders;

57. Review and examine the feasibility of establishing a methadone treatment and maintenance program within the provincial correctional system.

Chapter 6

COSTS

"If we could get away from the dollar cost and look at the human costs ... the human costs go on and on ... they never end."

Studies abound internationally and nationally in the field of substance abuse. There is a wealth of written materials in this area. Yet, from researchers, policy analysts, and bureaucrats alike, I have heard the call for more data, more information, and more research, if government is to make rational, effective decisions. To that I respond: "Balderdash!" The time has come to make decisions based on what we presently know, and from hindsight, what we can predict the future will hold. The administrative process is a decision-making process. It is time for decisions.

Let us, as a start, look at some of the economic costs associated with substance abuse, both licit and illicit substances.

The obvious, very visible, areas are law enforcement, health care, and social welfare. Then, we need to throw in catch-all phrases like prevention and treatment which run through those primary areas. Related, indirect costs, such as traffic accidents and workplace mishaps, reduced productivity, plus many others, can be incorporated for good measure. Suffice it to say that there are many, many costs associated directly and indirectly with substance abuse. The figures are guestimates at best, based on the best calculations researchers can assemble. These are imperfect scientific calculations, but like so much of the other scientific data in this field of human endeavour, it is probably sufficient for our purposes.

In 1989, drug law enforcement costs for British Columbia were estimated at $68 million. That included policing, federal prosecutions, corrections, and legal aid. Another $151 million was spent on the policing of non-drug crimes which were nevertheless related to drug use, including thefts, B & E's, weapons offenses, and crimes of violence. Federal and provincial programs directed at the prevention of substance abuse accounted for $13.6 million. Treatment services and employee assistance programs amounted to another $17 million. Health care programs for methadone maintenance, needle exchanges and infants of substance abuse mothers came in at $3.1 million. Social assistance for drug users was estimated at $20.6 million. Injury claims connected with automobile accidents approximated $39 million, while reduced productivity and workplace accidents having a drug relationship totalling $48.3 million.

The above 'guestimates' were for the year 1989. They totalled $360 million. Taking inflation into account, the figure in 1991 was estimated at $388 million. Consequently, three years later in 1994, the estimate could be said to be in the vicinity of $500 million. Depending on the formula used, the estimated cost in British Columbia reportedly reached as high as $653 million in 1989. Compare that with $2 billion in Ontario (1986-87) and $1.2 billion in Quebec (1988).

Statistics Canada and Health and Welfare Canada reported that, in 1989, British Columbia continued to have one of the worst drug problems in Canada. More people in BC reported having tried marijuana, cocaine, heroin, and other drugs than in any other province. BC had one of the highest rates of frequent drug use. The province also had the highest per capita rate of convictions for possession offenses and the second highest rate for trafficking offenses.

While one could question the statistics and calculations, there is no question to the average person on the street that we have a problem and that it has an adverse economic impact on governments,

community institutions, and the private sector. This is, of course, in addition to the serious health, social, and economic problems faced by drug abusers and their families.

Interestingly, it appears that the majority of these costs are incurred to support professionals and agencies directly involved in fields such as law enforcement and health care, while the target groups, the addicts, receive little in direct benefits. The costs appear to be more directly related to maintaining the symptoms and status quo, rather than diverting and redirecting the monies to the root causes and ultimate solutions for these personal and social problems.

One police officer wrote of his experiences in an interior community. He spoke of the increase in the number of armed robberies where knives and other weapons were simulated. A syringe full of "AIDS-tainted blood" was a weapon, with threatening becoming common. In addition to those costs in armed robberies, he went on to write:

> *"Thefts are a popular way for heroin and cocaine users to obtain funds to satisfy their habits. Residential and commercial Break and Enters continue to be a problem within the communities. Cigarette and tobacco thefts are common offenses. It is suspected that heroin users are responsible for a good number of these offenses. A majority of charges laid in these cigarette/tobacco thefts are against known heroin users. It is a well known fact that cigarettes are relatively easy to dispose of and that the culprits can obtain approximately 50 percent to 75 percent of their true value. This is in comparison to other types of stolen property (e.g. televisions, stereos, etc.) where the average profit is only approximately ten percent of the actual value of the property. Cigarettes and tobacco products are a known commodity in trading for heroin and cocaine. Information sources have on numerous occasions indicated that they support their heroin and cocaine use by cigarette thefts."*

How does one measure costs? The majority of those with whom I spoke talked mostly of what it costs to operate and maintain their treatment programs, of day-to-day costs just to survive. I didn't hear much on the costs referred to in this part of the report. Not too many spoke in terms of the millions of dollars being spent on the front end of the system and little was said on what is expended on the back end, except to say that not enough is being provided for the treatment of substance abusers. This usually came out in relation to the human costs. They need help; it is costly and that is where the money should be provided, but is not.

Chapter 7

EDUCATION

"We should build the fence at the top of the cliff, instead of at the bottom."

In travelling this province conducting public and agency meetings, it became crystal clear how important education is to prevention strategy. Prevention is synonymous with education. Indeed, this has not changed much over the years. Education has been identified since the early seventies as an important prevention strategy.

According to experts in education, prevention promises a better life for all: financially, socially, physically, and spiritually. S. R. Torjman in *Essential Concepts and Strategies*, states that prevention encompasses four strategies: education, regulation, skill development, and environmental factors. The three targets of prevention are the person, the drug, and the setting, or environment. Certainly this point was stressed in the 1991 Royal Commission on Health Care and Costs. The commission recommended that four public health strategies be adopted: the proclamation and enforcement of legislation to control the availability of drugs; initiatives to modify people's attitudes and behaviours; initiatives to teach people skills to make responsible decisions regarding drug and alcohol use; and the creation of environments more conducive to healthy behaviour.

During the course of this inquiry, most of the prevention concerns concentrated on school-based and broad-based community education. However, one of the things that was impressed upon me was the need for improved education within our most fundamental of institutions: the family. Children need self-esteem to stand up for themselves and for what they believe. Affirming words, supportive actions, and opportunities for success belong first of all at home. Only then can we expect a reflection of understanding and caring back into our communities, our schools and our institutions.

Self-esteem was not the only issue that came out loud and clear; so did responsibility. Most people I talked to felt that children are not always taught that people are responsible for their actions. Again, our schools and institutions can best work with a foundation built at home. Without the fundamental value of responsibility instilled at a very young age, it is difficult, but not impossible, for society to retroactively program this value. Without exception, addicts and professionals said we should start the actual learning process in the preschool home, even with parents during the prenatal period.

Sadly, while resources in this area exist, there are few comprehensive delivery modes and little coordinated approach to reach parents, particularly those parents who happen to be in the high risk category. Indeed, there are many single parents who could readily benefit from supportive information, skills, and resources in this area.

Children copy the adults in their lives. It was not that surprising to hear many people who have become addicted to drugs explain that this behaviour was learned at home. Often, negative impressions produce resolves not to follow in their parents' footsteps; later, however, different substances are found to abuse. Just as often, though, the actions of parents are copied:

> *"Dad did drugs ... I went straight to alcohol ... Later I got into drugs."*

> *"My parents didn't realize that I knew they were doing drugs and that I knew where they kept their stash ... I started using theirs ... until my Dad caught me and threw me out of the house ... I couldn't go back until I replaced his coke."*

The following best expressed the issue of the importance of early education:

> *"In the first 12 years of life children are building their 'tool box' for life. The decisions youths make later are based on the attitudes, values, skills, and impressions learned in the first 12 years."*

Even if the home environment has not been that positive, there is research to demonstrate a resiliency factor amongst some youths from disadvantaged homes, or from families where there were poor or abusive situations. Some of these children will rise above their background, as we have seen time and again. This was the recent message at a June 1994 University of Victoria Conference on *Strengthening the Family*. Also, significant influences by positive adults outside the home have proved effective. If a teacher or other mentor can reach a youth at risk, this can often make a difference in terms of a positive outcome.

Evidence that education works, particularly with youth, can be seen in the seat belt campaign to "buckle-up" and dentists' advertising to brush and floss. Current, age-appropriate information about drugs, alcohol, and other substances is required for all youth, but particularly during adolecence, in order to make competent decisions. "Just say No" messages are simplistic and no longer relevant in our society:

> *"Studies show that kids, even in preschool, are greatly influenced by alcohol advertising. Kids in elementary school will be able to tell you the names of beers, even when they have no idea who the Prime Minister of Canada or the President of the U.S. is."*

Overcoming myths is an important part of education as well. For instance, there is a myth that beer is harmless, not addicting, and has no affect on functioning. The fact that beer contains alcohol and can have all the effects of alcohol must be conveyed to youth. Similarly, there are myths amongst youth and "the now generation" that marijuana, various psychoactives, and cocaine have nothing but positive and enhancing effects for users. They must be informed of all the effects if they are to make the competent decisions alluded to earlier.

Truth in education is extremely important. Most people agree that scare tactics don't work. In my travels I heard many stories of individuals who became curious after listening to anti-drug propaganda at school. The book *The Black Candle*, published by a temperance activist in the early days of this century, includes extravagant tales of preposterous drug effects, some involving sexual aggressiveness and racial differences in response. This book is often cited as an example of ridiculous accounts about illicit drugs which actually may have spurred experimentation and discredited some of the more genuine and important messages about substance abuse. It would appear that the exaggerations undermined rational consideration and evaluation of the real properties and hazards of some of the then-legal drugs of that time, such as morphine and cocaine. As a result, they have been regulated and outlawed.

A June 1994 article in the British publication *The Economist* notes that many police authorities and people who work with substance abuse problems in Britain are calling for more accuracy and less doctrinaire teaching about drugs:

> *"They want teachers to admit to children that taking drugs can be pleasant as well as harmful ... 'The days of frightening people are over,' argues Mike Goodman, the director of Release, the National Drugs Advice Agency."*
>
> *"Many children, after all, realize that moderate soft-drug use may be no more harmful than alcohol or tobacco. You have got to be honest with children."*

While we can continue to argue that scare tactics do not work and integrity in anti-drug messages does work, the fact remains that youth will experiment. If they discover that the message they have been given is blatantly wrong, they will be less likely to listen to the very real cautions and messages concerning the more addictive and dangerous drugs.

Not only is it important to get the point across about honesty in drug messages to educators, we must first and foremost sell the importance of early drug and alcohol education. A key in this process will be school courses to promote analysis of and critical thinking about the media and advertising, such as the *Learning for Living (LFL)* curriculum. Approaching the Ministry of Education to support, fund, and include these programs in curriculum development is a positive way to proceed. These messages and images may then be incorporated into the school programs at appropriate levels.

The electronic media, of course, are important to education at the earliest stage of development. Studies underline the importance of TV and its impact on youth, who on average spend more time in front of the TV than in any other activity in the home. Equally, parental example is significant, particularly at early ages. However, concentrating or relying on parents to relay these messages verbally, over the option of electronic media, would be comparatively ineffective in promoting substance abuse education. Media, specifically the electronic media, in concert with parental example and communication, could be an effective force.

Unfortunately, we may be in a losing battle with the electronic highway and the visual media. Opposing messages and images are directly and subliminally impressed. "The exciting life," portrayed in action-filled dramas and "the glamorous life," portrayed in programming and advertising are often associated with alcohol and drugs. It is not easy to neutralize these images and forces through public service ads in the same media. They would have to be crafted to portray the same enthusiasm and ingenuity as is evident in, for instance, cigarette and alcohol advertising. Also, they would need to air as frequently as those ads, in order to get the desired exposure and effect. Young minds are impressionable. In their quest for pleasure and the absence of discomfort, there must be some confusion in these mixed messages.

There have been a number of recent developments in the substance abuse prevention field. Partnerships are being established which make sense and should be encouraged: the Child Youth Committee, inter-ministry committees, cross-ministry mandates, as well as the individual Ministries of Health, Social Services, Attorney General, and Education, are all addressing issues affecting children and their families. There are some resources for youth in place: the Inter-Ministry Committee for Youth at Risk; alcohol and drug counsellors at many secondary schools; parent teen mediation; mental health initiatives; and various other cooperative pilots which are intended to get agencies to work together. It is timely in this era of restraint to focus on those areas of critical importance, such as inter-ministerial commitments to prevention and education.

Teaching aids and elementary school books have already been developed with realistic dilemmas and role playing exercises. Some are designed to help parents identify the issues surrounding drug and alcohol use and ways to contend with these issues in the home and school environment. Some excellent training materials for high risk groups such as drug offenders and hard core users have also been produced. The resource book *Interact* was developed for the ADS (formerly ADP) and was apparently funded through the TRY budget campaign. One agency noted that despite frequent requests for this publication, this resource book remains out of print because of restraint.

In recent years, there has been a pilot project for school-based prevention (School-Based Prevention Project or SBPP). This three-year project has been implemented in BC secondary schools, funded by the Ministry of Health. The SBPP employs school-based prevention workers to prevent and reduce alcohol and other drug misuse amongst adolescents. In addition to the school-based prevention worker, the school-based team consists of a teacher, a school administrator, a learning assistance teacher, and a school counsellor, who help to coordinate the services of other health and counselling professionals. The thrust of the program is to improve individual awareness and responsibility, including enhancing self-esteem. Substance abuse prevention content focuses on equipping the student to handle societal pressures around the use of alcohol, tobacco and other substances, both legal and illegal, medical and non-medical. Services for students include assessment, in-class support services, placement, counselling, and other medical services. I am advised that this is an expensive program which goes beyond primary prevention.

The *Learning for Living Curriculum (LFL)* was designed to provide coordinated, sequential, age-appropriate health education which integrates health and physical education, home economics, contemporary issues, social roles, communication skills, and reproductive biology. This offers a comprehensive health education program to children throughout their schooling years. The program helps students reinforce attitudes and behaviours that increase decision-making and coping skills, amongst others. Although it is a commendable program, I was advised that the Ministry of Education has recently reduced its support and priority in this area. The province-wide training of teachers for implementation of the *LFL* program has been terminated. This means that few teachers will likely teach *LFL* as they will feel unprepared and unsupported doing so. Also disconcerting is the fact that *LFL* has been introduced in the elementary schools, but will not now be carried forward into the senior levels of school. In my estimation, based on public and agency input,

this is the one area which requires support. The public appears to consider the *LFL* Curriculum to be high on its list of priorities.

There also seems to be some basic disagreement about who should be providing substance abuse education. On the one hand, I heard that the teachers are already burdened with too much responsibility; on the other hand, most felt that school was the ideal vehicle. Teachers have built a relationship of trust with youth, and this provides an opportunity to reach most children. One education specialist suggested that teachers should receive more comprehensive training in substance abuse prevention, creating a health educator to deliver the LFL Curriculum.

I also heard that police should do law enforcement, not education, that they are not trained educators. "Are we sending the right message?" and "Are the programs applied evenly?" were questions that I heard. Most police forces have school liaison officers who talk to the children about a whole spectrum of issues, including substance abuse. Many educators and parents commended the police efforts to assist in this process. They felt that the police had a definite role in public awareness but that the lead role for educating children should rest with the teachers, with the support of specialists. One youth outreach worker I spoke to said, "The key is to have people with the skills to make the connection and to reach out to the kids."

Another important factor in education and prevention is involvement at the grass-roots or community level. An example is TO ADAPT (Thompson-Okanagan ADAPT), a non-profit society which helps member communities promote local prevention efforts. TO ADAPT coordinated a series of assessments pin-pointing common needs and differences for prevention strategies. These assessments, which were funded by Health and Welfare Canada, provided the basis for an inter-community project called *Community Based Prevention Response*. This project targets at-risk children from grades five to seven and their parents. It has four community prevention strategies as well as a component to provide regional training for interested communities tailored to meet each community's specific needs. Service groups have also become active in meeting community needs. I was advised that Kiwanis, Rotary, the Lions, and a number of other service clubs contribute substantially to community-based drug education material. Perhaps local business and community service groups could play a more significant role in supporting and funding some of the community prevention programs which are not fully provided for through government core funding.

It is also important not to lose sight of the various ethnic groups that are an important part of this problem in our society. Asian, Indo-Canadian, Hispanic, and many other groups are forming significant proportions of our population, particularly with growing immigration. In many of these cultural groups there are strictures and taboos against certain substances. Meanwhile, the youth from these families are in the mainstream of Canadian society and will adopt some of the behaviours of the dominant society. This may be through peer pressure, advertising, a desire to identify with the "in crowd" or escape from their own culture, or a combination of these factors. In Vancouver, the Alcohol-Drug Education Service (ADES) is an agency which is dedicated to reducing the effects of alcohol and drug abuse on families and communities. ADES develops materials for schools and communities, including parent groups. Interestingly, they have developed ethnic materials for Asian, Indo-Canadian, and Spanish- speaking communities. In some cases they have created videos and in one case a pregnancy calendar was designed to present an FAS/FAE message to young mothers.

I am sure there are many more programs that I have either not mentioned or been exposed to. However, despite the existence of these various programs and initiatives, there is more to do in this area if British Columbians are to encourage education in substance abuse. Many people I spoke to were still concerned that "there are drug deals going down in the school grounds" and "resources are not as reachable or accessible as they should be." They continue to worry about their children not having accurate or objective information or the practised skills to cope with normal peer pressure and increasingly aggressive drug dealers. For various reasons, some areas have progressed further in terms of education and service delivery than others; the materials and support systems are either not evenly applied or not equally available in all regions of the province.

It is important to educate not just at the introductory level of drug experimentation and initiation, but also throughout the community of users and addicts. Two areas identified as urgent targets for harm reduction, with the potential of saving lives, were educating the users in prison and on the street.

As mentioned earlier in this report, the Executive Committee on AIDS in Prison (ECAP), created in 1992 by the federal Solicitor General to promote and

protect the health of inmates and staff and to prevent the transmission of HIV and other infectious diseases amongst inmates, offered several recommendations regarding to education. The committee recommended that "existing educational programs for inmates and for staff be improved by including more input from external community-based organizations, experts and peers." The key is to promote safe behaviours surrounding the use of IV drugs, particularly the cleaning of injection equipment. The use of bleach as a cleaning agent, though controversial, has nevertheless been implemented.

Also on the corrections scene, the importance of education for both staff and inmates was the focal topic of a joint federal/provincial corrections and health conference in the spring of 1993 entitled The *Challenge of Choice*. Arising from this conference was the establishment of a joint federal/provincial Health Education Steering Committee, the first of its kind in Canada, "to determine the feasibility of a joint education program for correctional staff and inmates." The director of Health Services for British Columbia Corrections will chair a steering committee to establish an education program throughout correctional centres in BC. Research has confirmed the need to expand existing educational programs and prevention measures in an attempt to keep the current low levels of HIV infection from climbing. In recent studies, HIV prevalence rates were reported as high as two to five percent of inmate populations comprising injection drug users, younger inmates, and women.

It was noted during the *Challenge of Choice* conference that the higher risk behaviours and lower literacy levels of inmates, combined with their general mistrust of information provided by the system mitigate against standard education programs; thus, there is a challenge to develop new methods to convey existing and expanding knowledge.

And what of educating street addicts and users concerning safety, as new knowledge becomes available? I heard and read a great deal that awareness of the lethal combinations of either alcohol or other drugs with heroin should be part of a street-wise education program:

> *"The facts are of the utmost practical importance, for hundreds of deaths a year might be prevented by warning addicts not to shoot heroin while drunk on alcohol or under the influence of central nervous depressants or other drugs."*

Again, the value of the needle exchange and the outreach workers as the vanguards in the educational process is underlined. There is little doubt that new discoveries and new cautions, warnings, and better information will come from continued research. These must be conveyed to the street users, as well as the not-so-visible "recreational" users at the other end of the social spectrum. Education and the communication networks are paramount to containing and minimizing the problems associated with illicit drug use. The ideal in a drug and alcohol education program would be to cover a table with drugs and have no one want to take any of them. In reality, however, a total eradication of drugs — complete interdiction or zero supply —is out of the question.

While I believe society may not be able to turn the corner on stemming the drug supply, that is not to say that we shouldn't try to discourage imprudent use. I suggest it is important to focus on the demand side and reach the potential user at the earliest possible moment to hopefully promote healthy decisions and lower levels of drug use or non use, as the case may require. The key is the potential for harm and the level of use; a lot of aspirin taken at once can kill, whereas a small amount of heroin may be fairly benign. Any drug can be extremely harmful.

Last year, the province spent $86 million on alcohol and drug treatment programs, not to mention the money spent on enforcement, courts, legal aid, and corrections. I suggest that we seriously consider refocusing on more appropriate prevention modes. In carefully assessing the money being spent on treating adult alcoholics, let us examine preventing children and youth from developing patterns of harmful drug use. This is primary prevention: long-term versus short-term. Let us focus on education and prevention. I was advised that the *TRY* program was funded through a five cent tax on draft beer. This is an example of applying a small amount of the profit on a substance in order to deal with the problems created by that substance.

In examining educational methods and techniques, I believe there are some very effective tools now available that may not have been publicized or supported widely enough. We should teach youth to develop critical thinking around advertising, to analyze what is going on in a particular piece of ad work, and to decide if this is to be believed or not. These techniques develop a constructively critical mind, one that will take responsibility for the personal decisions made surrounding the consumption of products.

We should be educating our young people and working with parents as to how FAS, FAE, and NAS affect the unborn child. A special primary prevention manual (Trainer's Kit) for FAS/FAE prevention and management has been recently developed, funded by the Ministry of Health and produced by the British Columbia FAS Resource Society in collaboration with BC's Open Learning Agency. In this regard, there have been both federal and provincial Health Ministry advertising campaigns focusing on the dangers of drug and alcohol consumption by pregnant women and the effects on unborn children. There is also a great need to educate parents about the importance of setting examples for young children. Many affected youth and adults find themselves in trouble with the law. The disabilities associated with FAS/FAE impact all aspects of the justice system. Efforts such as these must continue to be prioritized, and promulgated as widely as possible. These efforts should be replicated for other alcohol and drug issues (e.g. high risk youth, women and addictions, seniors and drugs, as well as prevention for young children). This is all very expensive.

I recognize that there must be the political will to get this education launched. This year, we are spending $86 million on alcohol and drug treatment, with little of that going for education. "We should build the fence at the top of the cliff, instead of at the bottom." The real measure of cost effectiveness will be when we can put a greater portion of the treatment money into early education programs. We as a society, and particularly educators, must reach out to potential users. It is an important place to allocate funds for that long-term gain.

Much research has already gone into ensuring our educational materials are on track. There are volumes of US materials available, much of it focused on the philosophy of "Just Say No." The general consensus is this is too simplistic and ineffective. The US "drug war" literature was evaluated by many as "too negative and not very helpful." Education and communication are key to preventing personal and social problems flowing from illicit drug use. We must catch them before the problem (at an early age), during the problem (on the street), and throughout the serious failures (in jail). Thereafter, we must focus on recovery and rehabilitation. We never stop learning, even through death.

In 1991, the Royal Commission on Health Care and Costs recommended that the province establish an Alcohol and Drug Commission (ADC). Furthermore, the royal commission advised that at least one-third of the resources of this proposed ADC should be allocated to prevention. However, to date this has not been accomplished. Indeed, from my brief exposure to this field, there appears to be a need for specific authorities to be accountable to ensure that prevention efforts receive the priority and resources needed to be effective. In support of the royal commissions' recommendation for a governing body, and as a means of accomplishing it, there is a strong argument for placing a dedicated tax on alcohol to raise a multi-million dollar prevention/education fund annually. I would further suggest that the prevention fund be utilized to assist non-government provincial prevention agencies, community (lay) groups doing local prevention work, and those doing prevention research, evaluation, and program follow-up.

In addition to a general provincial prevention/education fund, the Ministry of Education should allocate sufficient funds to adequately implement a health education program for all ages, by training teachers to deliver the *Learning for Living* curriculum or its qualitative equal at all grade levels. Likewise, the ministry should review locally developed alcohol and drug education resource materials with a view to selecting the best for local school needs.

Additionally, the Ministry of Health and the Ministry of Education should place a priority on assisting and educating parents in positive drug and alcohol prevention strategies. Similarly, there should be initiatives developed for educating pre-parents, street-involved youth, and street people. Again, we need to focus on appropriate educational techniques and materials, taking into account the illiteracy factor.

Finally, linked to the above initiatives is the need to develop a greater number of appropriate education/prevention programs for clients and inmates in corrections facilities.

In summary, education is a key in a range of strategies which hold promise for preventing drug misuse, including overdose deaths. The best results will likely be obtained from a multi-faceted prevention program where education activities are supported by other prevention strategies. I must stress that education is more than disseminating facts and information. Skill development and practise are part of this process, as are the related goals of building self-esteem and a sense of responsibility, understanding, and learning to cope. Unfortunately, unless money, people, and time resources are earmarked for prevention, it is likely that the rhetoric will continue and little will change.

I therefore RECOMMEND THAT the Ministry of Education:

58. Expand and support existing teaching modules and develop locally-relevant teaching modules within the secondary school curriculum dealing with life skills, substance abuse, coping, and parenting, for use throughout the province;

59. Consider the establishment of permanent, trained substance abuse counsellors at designated schools (primary and secondary) throughout the province, to be available to both students and parents in the community;

60. Seriously consider deploying recovering addicts, who have been properly assessed, to act in a consultant/aide capacity with the school counsellor.

Chapter 8

LEGALIZATION/ DECRIMINALIZATION

"I think that criminalization is one little chunk of the problem. It has to be looked at in its entirety. If you decriminalize it you, will take out one chunk of costs, but you may be increasing it in another area."

The literature generally notes that these terms — legalization and decriminalization — are frustratingly vague and confusing and have different interpretations according to the interpreter. Perhaps the broadest meaning given is "unrestricted legitimate access to drugs."

Legalization can mean total deregulation of the production, sale, and use of all drugs (both so-called "soft" and "hard" drugs), so that the government has no business interfering with individual choices. However, then exceptions and qualifiers surface: they should not be sold to minors; they should be sold only through government-managed stores; advertising of the products should be prohibited, or at least controlled; and so on. Perhaps deregulation can be described as a sort of controlled, government regulated, free marketing of drugs.

Legalization has also been called decriminalization. The two terms were often used interchangeably as I travelled the province, especially in terms of the use of drugs. The basic idea is that people shouldn't get a criminal record or go to jail for merely using drugs. In other words, punishment should be dropped from the dictionary of justice for substance abusers, or, they would say, if there is to be punishment, it should be light, like a small fine — certainly not jail. Furthermore, they would add, law enforcement should concentrate on those heavy duty traffickers who import and sell for profit and greed, and, in so doing, wreak so much misery on the less fortunate users: the "Merchants of misery," as I heard them called.

Another approach presented was not so much deregulating drugs, but rather legalizing and decriminalizing them through a medical treatment plan. Merseyside (Liverpool, England) was cited as the example. In effect the methadone program would fall into that category, and the suggestion was put forward to expand that to a heroin program. The methadone program is strictly controlled, with guidelines and conditions for its use by a certified physician or pharmacist.

In some people's minds, these programs, policies, or practises were construed as legalizing the drugs. The fact is, no country has legalized the sale of so-called hard drugs. Even in Holland, where the practise is believed by many to be a legalization of the drugs, this is not so. Dutch authorities still deal with particularly heavy traffickers within the criminal model and with users through social services and medical treatment models. The Dutch, while still acknowledging the existence of the law, tolerate the sale and use of small amounts of marijuana and hashish, but apparently not heroin or cocaine. Instances like this have also occurred recently in Germany, Canada, and some American states. This is all done as a matter of practise or policy in some quarters; it is not done through legislation. The laws are still on the books.

The bottom line therefore is that legalization of illicit drugs has not yet occurred in any country. There have been no changes to the laws governing the sale and use of these illicit substances. There has been, however, through policy and practise, a

decriminalization in the use of small amounts of these drugs. What I believe is generally recognized now, at least through my discussions with officials, the general public, and users alike, is that while decriminalization will not end all the drug addictions and related problems, it would ameliorate many negative personal, family, and social consequences arising from the punitive criminal model, insofar as the user is concerned and affected.

We cannot reduce the harm these illicit substances are causing, unless we determine the personal and social root causes which give rise to the demands for these drugs. We have not been able to find solutions through incarceration and punishment. On the contrary, that solution is founded on negatives: resentment, hostility, and despair. I suggest that society must now reject negative criminal sanctions as the source of social control in drug abuse and turn rather to some other methods of control.

Time and time again I heard the following expressions: personal values, family values, role models, education, treatment, jobs, and housing. And yes, spiritual values: one of the basic tenets of the twelve step program in Narcotics Anonymous and Alcoholics Anonymous. We ought not to shy away from the use of that term *spiritual*, nor from applying it in whatever form it suits the user. The expression *holistic* was alluded to when recovering addicts and caregivers were discussing treatment programs. Both body and mind must be involved, neither one to the exclusion of the other.

Agencies involved in the drug abuse problem will not conquer or reduce the personal and social harms until they know, understand, and challenge the root causes of these problems. It is simplistic to argue that legalization of the sale and use of drugs would, for instance, curtail violence, thefts, prostitution, and so on. It may to some degree, but there is some suggestion that most addicts, unlike alcoholics, were involved in criminal activities before their addiction. In other words, the addiction was not the original and proximate cause of the criminal activity, but rather the result. What then were the causes of the original criminal activity?

In the various discussions and readings, the criminal acts and the experimental beginnings seemed to arise from the various influences and pressures to which the addict was exposed before his/her addiction. It has been suggested, even by recovering addicts, that freeing up the manufacture, sale, and use of drugs would exacerbate, not ameliorate, those earlier and original personal and social problems. Society has to determine and dislodge the deeply-rooted social problems before it can satisfy the various drug abuse demands being made of society.

In terms of harm reduction, if legalization is not the answer for society, is there not a middle course, such as decriminalization? If the criminal model is not working, should there be an expanded medical model for treating those who are caught up in the web of addiction? Would such a program reduce the harm to addicts, and equally important, to society?

It was clear from listening to confirmed and dedicated addicts that their physical cravings for heroin were uncontrollable. It was also clear that their need for drugs was interwoven with the drug subculture and criminal activities. Not only were addicts accepted in that environment, but what they did was acceptable. Conversely, neither they, nor what they did, was acceptable in the outside community. I had the distinct impression that legalizing or providing drugs to them would not, in itself, stop them from wanting more, better, and different drugs, nor from committing criminal acts and associating with others in their subculture. Methadone is being provided, but it is more addictive, more painful and "not good enough" for some of them. So it is sold on the outside, or "jacked up" with heroin. However, it is wrong to generalize.

For some, methadone works. For others, it does not. When the addict personally acknowledges that his/her time has come, makes a commitment to reform, and there is a support net to catch him or her - then it can work. However, the majority consensus was adverse to 'legalization', i.e. total deregulation of the production, sale and use of narcotics. Providing drugs to addicts who are completely caught up in the drug culture nourishes the growth, it does not snip the roots of the addiction, the crimes or the culture.

The issues of deregulation, decriminalization, and legalization cannot be fully addressed without considering the so-called "recreational" user. Most of what is written above alludes to serious abusers, the disadvantaged, the derelict addicts who are seen as a "blight on society." The "recreational" user is supposedly reasonable and in control in the use of these drugs; he/she can supposedly manage the intake and lead a normal life. The legalization argument is supported by alluding to tobacco and alcohol. "They are legal," proponents argue. Furthermore, in acknowledging the physical ills which flow from their abuse, they argue that the State nevertheless treats and cares for them. Abolition didn't work; people are going to smoke and drink

anyway. Abusers are treated and the government reaps considerable revenue from sales. There are more deaths from cigarettes and alcohol than from illicit drugs. These are all very accurate statements. It was also argued that if drugs were legalized, it would encourage people with severe problems to go to hospitals and other helping social agencies. Again, I believe this to be true, for many addicts expressed their fear of the police, courts, doctors, and social workers. Fear is a reality for addicts: not fear of the drug, but of everything around the addiction.

Countering this position was the argument that we ought to attend to the current abusive users, not increase the problem through sales. It was said that legalization would naturally increase availability and sales, with lower prices. This is probably true, but drugs are freely available now, at "reasonable" cost. Indeed, an addict offered me a number of "flaps" of heroin at a public meeting — for free. Then again, if alcohol is any indicator, increased availability seems to lead to increased consumption, with increased social and public health costs. No matter the price for the product or the various consequences, people will continue to buy and use tobacco, alcohol, and drugs. Isn't it a bizarre contradiction that we would argue for increases in taxes on an abused substance, in order to pay for the damages caused by the consumption of that substance. Society permits — perhaps creates — misery and despair and then spends like "drunken sailors" in an attempt to manage what it created.

Some others argued that if British Columbia adopted the "legalized" position, we would have an influx of addicts from not only the other provinces, but also from the United States and other countries. Others argued to the contrary. Who is to say? I think it is more important to weigh the social, economic, and public health consequences of legalization and to reflect on the quality of life and the kind of society we wish to build upon for our children, and their children, in the province of British Columbia.

It is abundantly clear to me that the drug problem here cannot be altered through the criminal justice system, the police, the courts, or the corrections system. If we are to reduce the harm to individuals (the addicts and their families) and to the various communities around the province, we are going to have to adopt broader social strategies to deal with drug abuse.

We have to disabuse ourselves of the notion that jail is the answer for users. Neither short sentences, long sentences, nor uniformity (consistency) of sentencing mean anything to the user. We have to establish alternatives to imprisonment. The cyclical process must end. It is simply ineffective, costly, and unjust. We must come up with more options for assistance and treatment and more accessibility. Removing threats and fears from users would allow them to "come out of the closet" and, with some outreach mechanisms, would provide a variety of programs for treatment and ultimate harm reduction. The money spent in policing, sentencing, and serving time would be much better spent on curing the causes, rather than labouring over the symptoms.

The sense I obtained respecting the traffickers, those who import and distribute drugs for profit and greed, is much different. They are, as one Supreme Court justice characterized, "Merchants of Misery." I would add to that "Distributors of Death." Given the continued demand for more enforcement and more resources on the part of law enforcement officials, I suggest society could benefit much more, financially and socially, by having the police concentrate specifically and precisely on these Merchants and Distributors. The courts would also do well to ensure the incapacitation of these people to the full extent allowed under the law. The police will have to review their policies, objectives, and goals with a view to establishing strategies which are innovative, more prevention/treatment-oriented, and more subculture community-based. This will require more labour-intensive approaches to community policing. It may well be that more of a social worker approach to preventing drug-related crimes, is needed, as opposed to the strictly law enforcement approach. We can no longer pour vast sums of money into the jails and prisons, all to no effect, when an available remedy is present to restrain, correct, and treat in the original instance.

The European experience around controlled legalization of certain drugs is reported as being positive. The Merseyside Regional Health Authority in England has recorded a decrease in drug-related acquisitive crime since authorizing the prescribing and dispensing of a wide range of previously-illicit drugs, including heroin. In many instances, these drugs are prepared in a smokeable form, thus eliminating the intravenous route of injection for many users. In Amsterdam, legalization has not occurred but law enforcement is very pragmatic. Law enforcement efforts concentrate on large scale distribution of heroin and cocaine; actual drug users are treated liberally and not forced to move underground.

Some users have minimal or no contact with the health care system and may be unwilling to acknowledge the risk associated with their behaviour. For this group, access to a system that controls the legal distribution of their drug of choice may be the only answer to reducing the immediate problem of death from drug overdose. Many of these individuals are injecting unknown substances of uncertain quality and quantity. Access to standardized, quality-controlled substances may be the safest option for them. This particular strategy is, of course, controversial. Any changes to existing legislation will require great political will and cooperation between provincial and federal politicians. Furthermore, the public will need to be properly educated about the rationale for making such a decision and reassured about any perceived negative consequences of legalization. In particular, the public will be concerned about the effect that legalization would have on the youth of the nation, who are perceived to be particularly vulnerable to changes that might increase legal access to mood-altering substances.

Legalization should not be considered the panacea or solution to substance abuse problems plaguing British Columbia and the rest of the country. It would not solve all the anti-social and criminal acts committed by confirmed addicts. Nor do I believe legalization would increase the incidence of those acts. On the contrary, what it would do is create that necessary "window of opportunity" for the addicts who have lost hope and freedom of choice. It would afford them the chance to get medical treatment by a physician and get their lives on track or give them some other sensible, reasonable way of dealing with their existence. This ought to provide or allow the person to function risk-free in the family and the community.

In summary, the matter of legalizing heroin, cocaine and other forms of now-illicit drugs requires considerably more thought than what is contained in these few pages. However, the facts are fairly evident that people are buying and using these products in British Columbia. They have been for some time and will continue to do so in years to come. It is also self-evident that people are importing and selling these products at considerable profits. Considerable harm — even death — can result from the consumption of these drugs. Costs are incurred by the general public in managing the problems associated with substance abuse, particularly illicit drugs. It is both a social problem and a health problem. Steps must be taken to control the problem, both immediately and strategically.

I therefore RECOMMEND THAT the Ministry of Attorney General:

61. Enter into discussions with the federal Ministers of Justice and Health on the propriety and feasibility of decriminalizing the possession and use of specified substances by people shown to be addicted to those specific substances;

62. In concert with the establishment of a Substance Abuse Commission, seriously inquire into the merits of legalizing the possession of some of the so-called "soft" drugs, such as marijuana.

RECOMMENDATIONS

I therefore RECOMMEND THAT the Government of British Columbia:

Establish an independent body under the aegis of the Legislative Assembly of the Province, with powers of inquiry into matters relating to the use and abuse of illicit and harmful substances in the province.

(see RECOMMENDATION 1, page ii)

This body would be comprised of a permanently appointed chair and qualified people seconded from the Ministries of Health, Social Services, Attorney General, and Education and, where requested or directed, municipal and community representation would be established locally with direct links to that body.

The body would be entitled the *Substance Abuse Commission.*

The mandate and terms of the reference of the commission would be to identify and advise the respective ministers of substance abuse issues and resolutions affecting their ministries, individually and collectively.

Without restricting the generality of the foregoing, the commission would:

(a) undertake research into the various treatment models, including methadone maintenance, harm reduction, and abstinence;

(b) develop an inter-ministry, multi-agency information research capacity to gather and share data on all aspects of substance abuse;

(c) develop standards, policies, procedures, and service models respecting the care and treatment of substance abusers in the province;

(d) examine existing legislation governing the controls over illicit and licit substances and, where abuses are identified, recommend amendments to legislation to the Legislative Assembly of the Province.

I therefore RECOMMEND THAT the Ministry of Health:

1. Examine existing programs within the Alcohol and Drug Services (ADS) in order to:

(a) distinguish alcohol detox from drug detox processes, for the information and benefit of both workers and clients;

(see RECOMMENDATION 16, page 27)

(b) establish separate facilities for alcohol detox and drug detox;

(see RECOMMENDATION 17, page 27)

(c) where feasible, conjoin those detox facilities with treatment centres, providing follow-up health care when those "windows of opportunity" present themselves;

(see RECOMMENDATION 18, page 27)

(d) improve and ensure access to detox facilities for young substance abusers (alcohol and drugs);

(see RECOMMENDATION 39, page 56)

(e) continue and expand mobile youth detox programs to communities with an established need;

(see RECOMMENDATION 40, page 56)

(f) establish and/or support alcohol and drug detox and treatment/recovery facilities strategically throughout the province, beginning with:

(i) Prince Rupert
(ii) Prince George
(iii) Kelowna
(iv) Nanaimo
(v) Campbell River

(see RECOMMENDATION 19, page 27)

(g) establish one combined alcohol and drug detox and treatment/recovery centre in the Downtown Eastside area of Vancouver, including counselling and outreach components;

(see RECOMMENDATION 20, page 27)

(h) provide more substantial strategic funding for existing supportive recovery programs which are functioning well;

(see RECOMMENDATION 22, page 35)

(i) Coordinate and expand the supportive recovery programs, provide longer term recovery, ensure staffing levels are adequate, and optimize small, community-based, home-type environments;
(see RECOMMENDATION 23, page 35)

(j) seriously examine and consider the deployment of properly-assessed, recovering addicts to act in a consultant/aide capacity to the staff of detox, treatment, supportive recovery, and needle exchange programs;
(see RECOMMENDATION 24, page 35)

2. In keeping with the harm reduction model:
(a) review funding initiatives to the various needle exchange programs, expanding and dealing strategically on the basis of the actual reasonable needs and costs associated with those programs;
(see RECOMMENDATION 10, page 23)

(b) collaborate with and provide greater assistance to municipal Public Health Departments and needle exchanges in providing counselling support and health care services through these facilities;
(see RECOMMENDATION 11, page 23)

(c) provide assistance in determining the quality of drugs on the street and in disseminating that information to both health care professionals and the addict population;
(see RECOMMENDATION 12, page 23)

(d) undertake research into the inter-relationship between alcohol and heroin in overdose deaths;
(see RECOMMENDATION 3, page 14)

(e) research and consider the feasibility of transferring responsibility for the methadone dispensing program in British Columbia from the federal Bureau of Dangerous Drugs to the provincial Ministry of Health;
(see RECOMMENDATION 21, page 32)

(f) prior to release from mental health facilities, provide people with or assist them in securing housing which will ensure the necessities of life and community support;
(see RECOMMENDATION 48, page 64)

(g) establish local treatment centres for family substance abuse, incorporating special needs daycare for children whose parents seek short or long-term substance abuse treatment;
(see RECOMMENDATION 33, page 50)

(h) consider the creation of facilities/clinics which incorporate needle exchange services, naloxone (Narcan) availability, methadone treatment/maintenance and counselling/outreach programs.
(see RECOMMENDATION 9, page 23)

3. Examine existing programs within the Emergency Health Services with a view to:
(a) providing an educational component for paramedics respecting the addict and addiction, in order to better understand and cope with this type of patient;
(see RECOMMENDATION 4, page 17)

(b) educating the public on the importance of calling 911 for an immediate response in suspected overdose situations;
(see RECOMMENDATION 5, page 17)

(c) extending the availability and application of naloxone (Narcan), or other narcotic antagonists, at critical locations and in addition to existing medical protocols.
(see RECOMMENDATION 6, page 17)

I therefore RECOMMEND THAT the College of Physicians and Surgeons:

1. Participate closely and directly in the establishment and operation of facilities/clinics which provide detox, treatment, recovery, and outreach programs, including needle exchange services, the availability of naloxone (Narcan) and/or other narcotic antagonists, and methadone treatment/maintenance programs;
   (see RECOMMENDATION 13, page 23)

2. Review the medical, personal and social feasibility of providing a paramedical heroin maintenance program within the service structures of such facilities/clinics;
   (see RECOMMENDATION 14, page 23)

3. Liaise with Regional Health Boards respecting an education component for emergency physicians and nurses, in order to better understand addictions and cope with the addict patient;
   (see RECOMMENDATION 7, page 17)

4. Liaise with Regional Health Boards to consider deploying properly-assessed recovering addicts in a consultant/aide capacity to emergency physicians and nurses, when an overdose patient is brought to the hospital;
   (see RECOMMENDATION 8, page 17)

5. Establish an advisory board, comprised of a cross-section of professionals and lay people, to conduct research and evaluate various treatment and maintenance models, including methadone, heroin, and abstinence and, through that advisory board, establish innovative, flexible, accessible treatment and counselling options, providing client/patient assessment through one centre, and establishing client profiles and treatment plans with the opportunity to refer them to other treatment programs.
   (see RECOMMENDATION 15, page 23)

I therefore RECOMMEND THAT the Ministry of Attorney General:

1. Establish mandatory alcohol and drug educational programs for young offenders, both institutional and community-based;
   (see RECOMMENDATION 52, page 74)

2. Provide an alcohol and drug detox and treatment program for young offenders, both institutional and community-based;
   (see RECOMMENDATION 53, page 74)

3. Designate one or more separate facilities within the adult correctional system as detox and treatment centres;
   (see RECOMMENDATION 54, page 74)

4. In collaboration with the Ministry of Health, provide an assessment, detox, treatment, and counselling program at that/those specific centre(s);
   (see RECOMMENDATION 55, page 74)

5. Where feasible, extend the educational, treatment, and counselling aspects of those programs to parents and guardians of offenders;
   (see RECOMMENDATION 56, page 74)

6. Enter into discussions with the federal Ministers of Justice and Health on the propriety and feasibility of decriminalizing the possession and use of specified substances by people shown to be addicted to those specific substances;
   (see RECOMMENDATION 61, page 88)

7. In concert with the establishment of a Substance Abuse Commission, seriously inquire into the merits of legalizing the possession of some of the so-called "soft" drugs, such as marijuana;
   (see RECOMMENDATION 62, page 88)

8. Review and examine the feasibility of establishing a methadone treatment and maintenance program within the provincial correctional system;
   (see RECOMMENDATION 57, page 74)

9. Discuss the merits and possibility of obtaining William Head Institution from Correctional Service Canada (CSC), for the purpose of converting it from a federal to a provincial centre for women and children to be used in the detox, treatment, and supportive recovery of people suffering from substance abuse and related problems;
(see RECOMMENDATION 38, page 51)

10. Explore the feasibility of entering into discussions with the federal Minister of Justice and the Solicitor General of Canada, with a view to applying revenue obtained through the *Proceeds of Crime* legislation in British Columbia to the cost of prevention, treatment and recovery programs here in the province;
(see RECOMMENDATION 50, page 68)

11. Examine and discuss with the federal Minister of Justice and the Solicitor General of Canada, the merits of mandatory maximum life sentences, without parole, for people importing and trafficking in large quantities of narcotic substances;
(see RECOMMENDATION 51, page 69)

12. Set aside funds for expansion of services for drug screening by the Provincial Toxicology Centre;
(see RECOMMENDATION 2, page 14)

13. Review the legality and propriety of "overserving" and offering discount beer or alcohol in beer parlours and pubs on or near "Welfare Wednesday," throughout the province, but particularly in the Downtown Eastside area of Vancouver;
(see RECOMMENDATION 30, page 41)

14. Reduce the number of available seats in beer parlours, particularly those in the Downtown Eastside area of Vancouver ;
(see RECOMMENDATION 31, page 41)

15. Enter into discussions with the federal Minister of Immigration respecting the need to review and determine the status of non-Canadians who import and traffic in large quantities of narcotics.
(see RECOMMENDATION 49, page 68)

I therefore RECOMMEND THAT the Ministry of Social Services:

1. Develop and implement an educational program for ministry employees who work in the fields of social assistance and substance abuse for a greater understanding of the addict and addiction;
(see RECOMMENDATION 25, page 41)

2. Develop and establish education programs in the areas of parenting, life skills, and coping, for the various clients (parents, guardians, and youth) within the welfare networks of the ministry;
(see RECOMMENDATION 26, page 41)

3. Develop and establish follow-up programs for parents and youth in care who have completed detox and treatment programs within other ministries;
(see RECOMMENDATION 41, page 56)

4. Provide adequate day care, travel, and financial support to mothers attending substance abuse treatment programs;
(see RECOMMENDATION 34, page 50)

5. Consider staggering social assistance payments throughout the month rather than concentrating them near the end of the month;
(see RECOMMENDATION 27, page 41)

6. Consider electronic rent payments to welfare landlords;
(see RECOMMENDATION 28, page 41)

7. Conduct a review of existing premises to ensure reasonable accommodation standards are provided by the welfare landlords and that increases are justifiable and warranted;
(see RECOMMENDATION 29, page 41)

8. Continue and expand multi-cultural education and training to service providers;
(see RECOMMENDATION 43, page 62)

9. Examine ways and means of creating and implementing job placement strategies for welfare recipients returning from alcohol and drug detox, recovery and treatment programs;
(see RECOMMENDATION 32, page 43)

10. Examine ways and means of increasing the availability of appropriate housing, encompassing a range of settings, including community homes, independent living arrangements, safe houses, and transition houses for recovering addicts, particularly women, children, and the disabled, in order also to discontinue exposure to an alcohol and/or drug-violent environment;
(see RECOMMENDATION 35, page 50)

11. Provide increased funding for support programs which target women with substance abuse problems, including a wider range of treatment choices for women and families which address child care needs with residential and non-residential options;
(see RECOMMENDATION 36, page 50)

12. Review ministry policies and practises which remove children from mothers or families suffering from addiction, recognizing such threats to parents seeking treatment and the benefits of early intervention and counselling referrals to treatment agencies.
(see RECOMMENDATION 37, page 50)

I therefore RECOMMEND THAT the Ministry of Education:

1. Expand and support existing teaching modules and develop locally-relevant teaching modules within the secondary school curriculum dealing with life skills, substance abuse, coping, and parenting, for use throughout the province;
(see RECOMMENDATION 58, page 83)

2. Consider establishment of permanent, trained substance abuse counsellors at designated schools (primary and secondary) throughout the province, to be available to both students and parents in the community;
(see RECOMMENDATION 59, page 83)

3. Seriously consider deploying recovering addicts, who have been properly assessed, to act in a consultant/aide capacity with the school counsellor.
(see RECOMMENDATION 60, page 83)

I therefore RECOMMEND THAT the Ministry of Aboriginal Affairs:

1. Continue and expand multi-cultural education and training to service providers;
   (see RECOMMENDATION 43, page 62)

2. Review present outreach services and support for native people who are substance abusers;
   (see RECOMMENDATION 44, page 62)

3. Review assistance provided to native detox, recovery, and treatment centres, with a view to assisting in the development of band and local responses in addressing alcohol and drug issues in the respective locales;
   (see RECOMMENDATION 45, page 62)

4. Review the need for and provision of assistance to native youth drop-in centres and community-based clinics which offer alcohol and drug services, counselling, and education;
   (see RECOMMENDATION 46, page 62)

5. Invite native people, including recovering substance abusers, to participate more fully in the planning of regional and local services and programs.
   (see RECOMMENDATION 47, page 62)

I therefore RECOMMEND THAT Municipalities throughout the province take the lead role in:

1. Providing community activity centres which encourage constructive life styles and skills, with an educational component which incorporates counselling in substance abuse.
   (see RECOMMENDATION 42, page 56)

DEFINITIONS

MEDICAL TERMS

Acquired Immunodeficiency Syndrome (AIDS): AIDS results from infection with the HIV virus. The syndrome is characterized by progressively weakened immunity, which makes the patient susceptible to opportunistic infections and unusual cancers.

Agonist: A drug capable of combining with receptors to initiate drug actions; it possesses affinity and intrinsic activity.

Analgesic: A compound capable of producing analgesia; i.e. one that relieves pain by altering perception of nociceptive (pain) stimuli without producing anaesthesia or loss of consciousness.

Antagonist: Something opposing or resisting the action of another. Narcotic antagonists neutralize the action of the narcotic, thereby providing specific treatment for narcotics poisoning. The antagonist of choice is naloxone (NARCAN).

Antenatal: Occurring before birth; compare Postnatal below.

Autopsy: Postmortem examination; an examination of the organs of a dead body to determine the cause of death or to study the pathologic changes present.

Blood-brain Barrier: A selective mechanism of blood vessel permeability opposing the passage of most ions and large-molecular compounds from the blood to brain tissue.

Congenital: Born with; existing at birth. Refers to certain mental or physical traits, anomalies, malformations, diseases, etc., which may be either hereditary or due to an influence occurring during gestation up to the moment of birth.

Endorphin: Naturally occurring opiod peptides originally isolated from the brain but now found in many parts of the body.

Ethanol: Alcohol; ethyl alcohol; drinking alcohol.

Etiology: The science and study of the causes of disease and their mode of operation.

Euphoric: Adjective form of euphoria (a feeling of well being).

Fetal Alcohol Effects (FAE): Alcohol-related birth defects. FAE is the term used to describe children who have some of the Fetal Alcohol Syndrome (FAS) characteristics but lack the complete morphological syndrome (facial deformities).

**Fetal Alcohol Syndrome (FAS):**	Alcohol-related birth defects. FAS is a recognizable pattern of malformation, encompassing neurobehavioural abnormalities and cognitive impairment, prenatal and/or postnatal growth retardation, and characteristic physical and facial features.
**Gestation:**	The term of pregnancy, usually approximately 40 weeks.
**Half-life:**	The time during which a drug or substance is decreased to half its original concentration and eliminated from the body.
**Hepatitis B:**	Inflammation of the liver caused by infection due to hepatitis virus Type B or "serum hepatitis.' The recognized route of infection for Type B is parenteral (by intravenous or other types of injection). Other routes of transmission include sexual contact.
**Human Immunodeficiency Virus (HIV):**	This virus is the etiologic factor most closely related to AIDS. Risk groups include homosexual and bisexual men who are sexually active, intravenous drug abusers, prostitutes and their clients, those engaged in unprotected sexual promiscuity, hemophiliacs, especially those who have been treated with Factor VIII concentrate, and heterosexual partners of those in high-risk groups (i.e. those with a history of multiple blood transfusions).
**HTLV-I/II:**	Human T cell Lymphotropic Virus types I and II.
**Hyperthermia:**	Hyperpyrexia or extremely high fever.
**Injection:**	Introduction of a substance into the subcutaneous cellular tissue, a vein, or an artery.
**Intravenously:**	Injection into a vein.
**Mechanism of Death:**	The physiological derangement or biochemical disturbance produced by the cause of death which is incompatible with life (e.g. respiratory depression). The cause of death is the disease or injury responsible for initiating the train of events, brief or prolonged, that produced the fatal end result.
**Minimum Lethal Levels:**	The lower level of concentration of a drug in body fluids to produce death. This lethal range is a reflection of multi-national, experience citing cases which appear to be drug overdoses.
**Morbidity:**	Disease or illness; the ratio of sick to well in a community.
**Mortality:**	Death; fatal outcome. The mortality rate is the number of deaths occurring relative to specific living populations.
**Neonatal Abstinence Syndrome (NAS):**	Drug-related withdrawal symptoms in a newborn infant.
**Pathology:**	The medical science and specialty practise concerned with all aspects of disease, but with special reference to the essential nature, causes, and development of abnormal conditions, as well as the structural and functional changes that result from the disease processes. Anatomical Pathology is the sub-specialty that pertains to the gross and microscopic study of organs and tissues removed for biopsy or during postmortem examination, as well as the interpretation of the results of such study.

Placenta: Organ of metabolic interchange between the fetus and the mother.

Postnatal: Occurring after birth; compare Antenatal above.

Therapeutic: Relating to the treatment of disease or disorders by remedial agents or methods.

Toxic: Poisonous; pertaining to a toxin; the state of being poisonous.

Toxicology: The science of poisons, including their source, chemical compositions, action, tests, and antidotes.

Trimester: A period of three months; one-third of the length of a pregnancy.

Tuberculosis (TB): An infectious, communicable disease, caused by the presence of Myobacterium Tuberculosis, which may affect almost any tissue or organ of the body, the most common seat of the disease being the lungs.

DRUG-RELATED TERMS

Depressants: Agents that reduce nervous or functional activity, such as sedatives or anaesthetics. Depressant drugs initially depress the inhibitory pathways of the central nervous system, making the user feel relaxed, even euphoric. These agents include alcohol, tranquilizers and narcotic analgesics.

Alcohol:

Alcohol is considered a drug because its main ingredient, ethanol, acts on the brain as a depressant. Tolerance is created with regular use of alcohol and results in more alcohol being needed each time to achieve the same effect.

The short-term effects of alcohol include impairment of motor and perceptual skills, gastric stimulation and relaxation of inhibitions. The long-term risks associated with abuse are cirrhosis of the liver, gastro-intestinal hemorrhage, hematologic disorders, neurologic damage, hepatitis, heart disease, and overdose.

Heroin:

Narcotic analgesic with pain-killing and addictive properties. Heroin is a semi-synthetic compound made by chemical modification of morphine. Morphine and Codeine are derivatives of opium, which comes from the Asian poppy plant. In the body, heroin rapidly converts back to its chief active metabolite, morphine.

Street terms for heroin include Smack, Junk, Horse, and Black Tar.

The short-term effects of heroin are pain relief, decreased respiration, constipation, nausea, itching, and sweating. Long-term risks include mood instability, reduced libido, constipation, and overdose.

Methadone:

A synthetic narcotic drug; an orally effective analgesic similar in action to morphine but with slightly greater potency and longer duration. Used as a replacement for morphine and heroin.

Morphine:

The chief active ingredient in opium.

Designer Drugs: Synthetic substances that are prepared by underground chemists to resemble existing potent but legal drugs, usually in the narcotic or amphetamine categories.

Fentanyl analogs:

Similar in structure to fentanyl, but illegal. Their effects are similar to heroin and morphine and they create the same type of addiction.

Amphetamine-like "new" drugs:

MDA and MDMA. Both synthesized drugs are known as psychedelic amphetamines. They possess psychedelic properties like LSD and have the stimulant properties of amphetamines. MDMA is also known as "Ecstasy' or "Adam'"

Meperidine analogs:

Meperidine is a synthetic narcotic known by the trade name Demerol. It has effects similar to those of morphine and heroin and is a coveted street drug when heroin is scarce. The main analog is MPPP, a more potent drug than meperidine but one which presents significant risks of overdose. Moreover, an impurity formed during the clandestine manufacturing of MPPP is extremely dangerous.

Hallucinogens and Mind-altering Drugs: Drugs that induce hallucinations or psychedelic alterations in perception, thought, and affect. This group includes LSD, PCP, and inhalants. Hallucinogen abuse has been associated with severe depression, paranoia, and prolonged psychosis. Pre-existing psychiatric disorders may be of primary significance in these cases.

Marijuana:

Marijuana comes from the plant Cannabis Sativa. The green leaves of the plant are rolled into a cigarette or "joint" and smoked. Loose marijuana is also smoked using various kinds of pipes. Marijuana contains hundreds of chemicals, the most important being THC, the main chemical responsible for the "high." Marijuana- related drugs have some effects similar to the sedatives and, particularly in higher doses, to the hallucinogens.

Short-term effects include increased heart rate, reddening of the eyes, some impairment of motor skills, and euphoria. Long-term risks include lung damage in extreme cases and sedative effects.

Hashish:

Hashish, the concentrated resin of the plant has an even higher level of THC. It is usually dark brown or black resin that can be broken into small pieces and smoked.

Hashish oil is a powerful tar-like substance. One or two drops will usually be placed on a cigarette or joint.

Street terms for marijuana include Pot, and Ganja.

LSD:

Lysergic acid diethylamide (LSD) is the most powerful and well-known of all hallucinogens. LSD is a colourless, tasteless powder that can be found in a capsule or in a pill. Since the powder is extremely water soluble, and easily absorbed, drops of LSD are placed on blotters. LSD and other hallucinogenic drugs produce significant changes in perception, mood, and thought. These very often include visual hallucinations and distortions, where the user is aware that these experiences are not real.

Short-term effects of LSD include perceptual distortion, heightened sensory experiences, increased blood pressure, and nausea. Long-term risks include possible flashbacks, tolerance to effects, and some psychological difficulties for a few.

PCP:

Phencyclidine (PCP); also known as Angel Dust. It was first invented as a human anaesthetic but because of severe side effects its use on humans was discontinued. It was later used for a time in veterinary medicine. In its pure form, the drug is a white crystalline powder that can be snorted or sprinkled on tobacco, parsley flakes, or marijuana and then smoked. In liquid form, it can be injected.

Short-term effects include time and space distortion, dissociative state, and the risk of overdose. At high doses it can bring coma and death.

Mushrooms & Cacti:

Other hallucinogens include mescaline, the naturally-occurring hallucinogen found in the Mexican peyote cactus, as well as other cacti in South America. These hallucinogens have the same effect as LSD, but last for a shorter period of time.

Illicit: Not permitted, unlawful.

Licit: Conforming to the requirements of law.

Naloxone (Narcan): A synthetic narcotic antagonist which has been used clinically since 1962. Recovery is often dramatic. However, severe respiratory depression may recur within several hours. Narcan is devoid of pharmacologic action when administered in the absence of narcotics.

Narcotics: Drugs derived from opium or opium-like compounds, with potent analgesic effects associated with significant alteration of mood and behaviour and with the potential for dependence and tolerance following repeated administration.

Opiates: Any preparation or derivative of opium.

Opiod: Denoting synthetic narcotics that resemble opiates in action.

Opium: The dried juice of the unripe capsule of the opium poppy.

Poly-addicted: Addicted to more than one substance.

Polydrug users: People using one or more drugs in varying combinations.

Stimulants: Agents that arouse organic activity, strengthen the action of the heart, increase vitality, and promote a sense of well-being; classified according to the parts of the body upon which they chiefly act (i.e. the cardiac and respiratory systems). The principle drugs of abuse in this group are the amphetamines and cocaine. Life-threatening doses produce hyperthermia, convulsions and cardiovascular collapse.

The short-term effects of amphetamines include increased heart rate, restlessness, euphoria, irregular breathing, and the risk of convulsions and overdose. Long-term risks include high blood pressure, anxiety, tension, insomnia, skin rash, overdose, and paranoia.

Cocaine & Crack:

Cocaine is one of the most powerful central nervous system stimulants known to mankind. It produces heightened alertness, inhibition of appetite, and intense feelings of euphoria and has been widely used in medicine as a local anaesthetic. Cocaine is found in the leaves of a South American shrub called the Erythroxylon coca.

Crack is a form of cocaine that has been chemically altered so that it can be smoked. It looks like small lumps or shavings of soap but has the texture of porcelain.

Street terms for cocaine include Snow, Rock, Nose Candy, and Coke.

Methamphetamine:

A new synthesized form of the well-known drug methamphetamine or "Speed." Methamphetamine produces mainly central nervous system stimulation, reduction of hunger, and an overall feeling of well-being.

The term ice is a street name that refers to a form of crystal methamphetamine (actually methylmethamphetamine) which can be smoked.

Street terms for methamphetamine include Speed, Crank, Crystal, and Meth, depending on the physical form (crystal vs powder), the geographical area, and the dealer.

Abstinence: Refraining from the use of something (e.g. alcohol, drugs, food, etc.).

Acute Intoxication: Poisoning; not chronic, but of short and sharp course.

Addiction: A phsiological dependence on a drug to sustain or maintain a physically desirable state or to avoid undesirable physical effects of withdrawal from that drug (see dependency).

Boosting: Slang for shoplifting.

Brew: Inmate slang for alcohol fermentation from food stuffs.

Co-ingest: To take more than one substance at the same time or within a short time frame.

Contraband: Items which it is unlawful to possess (e.g. weapons, illicit drugs, alcohol or other materials which are not allowed in correctional facilities).

Dependency: Physical dependence is said to occur when the body has become adapted to a drug and needs it to function properly.

Epidemiology: The study of the distribution and causes of health problems in human populations.

Habituation: A psychological dependence on a drug, or related activity, to maintain a mental state of satisfaction. Many drugs are both addicting and habituating.

Idiosyncratic: Relating to or marked by an idiosyncrasy or peculiarity.

Incidence: Number of new cases of a disease, illness, or condition in a specified period of time.

Interdiction: Intercept, prohibition, or banishment; a governmental prohibition of commercial trade, intended to bring about an entire cessation for the time being of all trade.

Prevalence: Total number of people with a disease, illness or condition in a specified period of time.

Primary Prevention: A prevention approach which seeks to prevent a disease or health problem before it occurs, often during a period of susceptibility. In the area of substance use, primary prevention aims to prevent the use and/or misuse of alcohol and other drugs amongst those who have never used them or to delay the age at which onset of use occurs.

Residential Treatment: Drug or alcohol treatment with a room and board or residential component.

Secondary Prevention: A prevention approach which seeks to detect disease, illness, or health problems very early in their development and to treat them promptly in order to help prevent the full blown condition. In the area of substance abuse, secondary prevention involves identifying adolescents who have just started to use alcohol and/or other drugs, helping them to quit, cut down, or use in moderation and protecting them from the consequences of misuse, while seeking a more permanent solution.

Tertiary Prevention: The rehabilitation or treatment of a condition to prevent death, permanent disability, or reoccurrence of the condition. In the area of substance use, tertiary prevention includes treatment programs that help addicted youths recover and relapse prevention programs that aim to keep the newly recovered addict substance-free.

Tolerance: Tolerance sets in when the user needs even higher doses of a drug to feel the same effects or needs to use the drug more often.

Withdrawal: When the user abruptly stops using the drug, the body reacts by going through withdrawal symptoms. Traditionally associated with the use of drugs like alcohol and heroin, where the reactions were easily identified. Today experts agree that cocaine and even marijuana have their own set of withdrawal symptoms, such as craving, depression or sleep disturbance.

BIBLIOGRAPHY

Alexander, B.K. Peaceful Measures - Canada's Way Out of the 'War on Drugs'. Toronto: University Press, 1990.

Alexander, B.K., T.M. MacInnes and B.L. Beyerstein. "Methadone Treatment in British Columbia: Bad Medicine?" Canadian Medical Association Journal, 1987. 136:25 - 28.

---. "Methadone and Mortality Among Drug Users." British Columbia Medical Journal, 1988. 30:160 - 3.

Atkins, N. "The Cost of Living Clean." Rolling Stone, 5 May 1994. 41 - 42.

"Bad News Again: Teen Drug Use Increasing." Narcotics Control Digest (Washington Crime News Services), Vol. 24, no. 3, 2 February 1994.

Baselt, R.C. Disposition of Toxic Drugs and Chemicals in Man, 2d edition. Davis, CA: Biomedical Publications, 1982.

Begley, Sharon. "One Pill Makes Youth Larger, and One Pill Makes You Small." Vancouver Sun, 5 February 1994.

Bell, R. "The History of Drug Prohibition and Legislation." International Criminal Police Organization, ICPR, September - October 1991.

Bell, S. "Heroin Claiming Renewed Grim Toll." Vancouver Sun, 24 January 1994.

Bergerman, R.T. "Illicit Narcotic Deaths in BC." Drug Intelligence & Field Operations, RCMP, (unpublished) n.d.

Bethell, B.J., N. Barty and C.D. Bellward. "A Drug Abuse Program for Grades 4 - 7." BC Teacher, January 1994.

Black's Law Dictionary, 5th ed. St. Paul: West Publishing Company, 1979.

Boyd, N. High Society Legal and Illegal Drugs in Canada. Toronto: Key Porter Books, 1991.

Brecher, E.M., and Editors. Consumer Reports: Licit and Illicit Drugs. Boston: Little, Brown & Co., 1972.

British Columbia. Ministry of Aboriginal Affairs. A guide to Aboriginal Organizations and Services in British Columbia. Victoria, March 1992.

---. Ministry of Attorney General, Coordinated Law Enforcement Unit. "Cocaine: Demand Reduction Strategies, Final Report, Victoria, June 1987."

---. Ministry of Attorney General, Coordinated Law Enforcement Unit. "Cocaine: Demand Reduction Strategies, Appendices & References, Victoria, June 1987."

---. Ministry of Attorney General, Coordinated Law Enforcement Unit. "The Costs Associated with Illicit Drug Use in British Columbia in 1989." September 1992.

---. Ministry of Attorney General, Coordinated Law Enforcement Unit. "Updates on Drug-Related Projects, Victoria, April 1992."

---. Ministry of Attorney General, Coordinated Law Enforcement Unit. "Overview: CLEU's Contributions Toward the Supply and Demand Reduction of Heroin." Ridell, B., Communication with Chief Coroner, (unpublished) March 1994.

---. Ministry of Attorney General, Coroners Office. "Illicit Drug Statistics Update." March 1994.

---. Ministry of Attorney General, Drug Strategy Committee. "Provincial Drug Abuser Monitoring Program, Special Report Heroin Update." January 1993.

---. Ministry of Health, Prevention and Health Promotion Branch. School-Based Prevention Model Handbook. Victoria, March 1994.

---. Report of the British Columbia Royal Commission On Health Care and Costs, Closer to Home. Victoria: Crown Publications, 1991.

---. Ministry of Women's Equality. Report of the British Columbia Task Force on Family Violence: Is Anyone Listening? Victoria, 1992.

British Columbia Civil Liberties Association. "Comments On the Treatment and Management of Methadone Patients In Private Care in BC." June 1986.

Brown, E.R. and B. Zuckerman. "The Infant of the Drug-Abusing Mother." Paediatric Annals, 20:10/October 1991.

Buckley, L. Ralph. "Report of the Vancouver Downtown Eastside/South Mental Health Needs Assessment Survey (October 1991 to April 1992)." BC Mental Health Society and Greater Vancouver Mental Health Service, 1993.

Burroughs, William S. Naked Lunch. New York: Grove Weidenfeld, 1959.

Canada. Action on Drug Abuse - Making a Difference. Ottawa: Ministry of Supply and Services, 1988.

---. Department of Justice. Submission to the Task Force Into Illicit Narcotic Deaths in British Columbia, (unpublished) June 1994.

---. Ministry of National Health and Welfare. Canada's Drug Strategy. Ottawa, 1992.

---. Ministry of National Health and Welfare. Licit and Illicit Drugs In Canada. Ottawa: Ministry of Supply and Services, 1989.

---. Ministry of National Health and Welfare. National Alcohol and Other Drugs Survey Highlights Report, 1989. Ottawa: Minister of Supply and Services, 1990.

---. Senate. Minutes of the Proceedings and Evidence of the Standing Committee on National Health and Welfare. Booze, Pills & Dope, Reducing Substance Abuse in Canada. Issue No. 28, 1987. Second Session of the Thirty-third Parliament 1986 - 1987.

CPS Compendium of Pharmaceuticals and Specialties, 27th ed. Ottawa: Canadian Pharmaceutical Association, 1992.

Carey, N., T. Oberlander, C. Loock and P. Robeson. Sunny Hill Health Centre for Children. Submission to Task Force on Illicit Narcotic Deaths, (unpublished) June 1994.

Clark, Gordon. "Back From the Brink." The Province, 25 June 1993.

"Cocaine an Addictive Killer: Drug Expert." Vancouver Sun, 2 June 1993.

College of Physicians & Surgeons of BC. "Proposal To Improve The Quality of Care for Methadone Maintenance Clients in BC." 30 April 1992.

---. "Task Force. Illicit Narcotic Deaths Summary." Communication to the Chief Coroner (unpublished), 1994.

Conroy, J. "Issues for Families and Communities: The Adolescent with FAS in the Criminal Justice System." Notes from presentation to Conference to Address FAS and NAS, Vancouver, 1994.

Cooper, Mary. Wasted Lives The Tragedy of Homicide in the Family. Vancouver: BC Institute on Family Violence, July 1994.

Cooper, Mary, A. Karlberg and L. Pelletier Adams. Aboriginal Suicide in British Columbia. Vancouver: BC Institute on Family Violence, April 1991.

Cotts, C. "Smart Money." Rolling Stone, 5 May 1994, 42 - 43.

Currie, Elliot. Reckoning: Drugs the Cities the American Future. New York: Hill Wang, 1993.

Daum, Kimber-Ann. "Clean Up Attitudes Before We Clean Up the Streets." The Province, 8 August 1993.

Dedyna, Katherine. "The Young and the Blasted." Times Colonist, 25 February 1994.

"Dialogue on Drugs." Newsletter of the Concerned Citizens' Drug Study and Educational Society, Vol. 5, No. 1, Spring, 1994.

Directory of Substance Abuse Services In British Columbia 1994-95. Kaiser Youth Foundation/Alcohol and Drug Services, BC Ministry of Health.

Dogoloff, Lee I. "A Perspective on Needle Exchanges." American Council for Drug Education. n.d.

Downtown Eastside/Strathcona Alcohol and Drug Advisory Committee. "Community Voices: Results of Seven Focus Group Discussions Concerning Alcohol and Drug Services in the Downtown Eastside and Strathcona." March 1994.

Drug Information Reference. 2d ed. BC Drug and Poison Information Centre, 1987.

"Drugs Women's Top Scourge." United Press International as reported in the Province, 03 June 1994.

"Dutch Drug Policies Changing in Light of Increasing Criminality." Criminal Justice Newsletter, Vol. 4, No. 2, March/April 1994.

Elliott, Patricia. "A Life in the Drug Trade." Saturday Night, December 1994.

"Experts on AIDS and Prisons Release Final Report." Canadian AIDS News, March/April 1994.

Farrow, M. "Rich Nations Failing Kids, Hard-hitting Report Says." News Article on UNICEF. Second issue of 'Progress of Nations'. Vancouver Sun, 22 June 1994.

---. " 'Highly Addictive' Khat Arriving Via White Courier." Vancouver Sun, 4 December 1993.

Fayerman, Pamela. "Children as Young as 5 Years 'Play Roles' in Drug Deals." Vancouver Sun, 16 March 1994.

"Forfeiture of Foreign Drug-Related Assets in the United States." ICPR, May - June 1992.

Frankfurt Resolution. Final Agreement Between the European cities of Amsterdam, Frankfurt, Hamburg and Zurich. (1990)

Gould, T. "Drug War? What Drug War?" WMA Winners, 1993. 54 - 57.

Gunning, K. F. (President. Dutch National Committee on Drug Prevention) "Crime Rate and Drug use in Holland." 1993.

Hall, Neal. "Public Has Distorted View of Youth Role in Gangs, Study Says." Vancouver Sun. 31 January 1994.

"Heroin Heaven Bedevils Police." Globe and Mail, 13 September 1991.

Huckin, S.N. "Pure Heroin Overdoses." Communication with the Chief Coroner, (unpublished) 1994.

---. "Statistics Concerning Heroin and Cocaine Deaths." Unpublished letter 26 January 1994.

Illicit Drugs & Alcohol: Facts and Information. Toronto: Alliance for a Drug Free Canada. 1992.

Interpol General Secretariat. "The Drugs Situation in Europe After 1992." ICPR, July - August 1990.

---. " 'Ice' and 'Ecstasy' Two Dangerous Psychotropic Drugs." ICPR, July - August 1990.

Jiwa, Salim. "Pint-sized Pusher." Province, 15 March 1994.

Johnson, M.E. "Empowering Our Communities' Health Promotion Strategies: YWCA Crabtree Coroners's Community Based Model." Presentation at Conference to Address FAS and NAS, Vancouver, 1994.

Kalter, H.D., A. J. Ruttenberg and M.M. Zack. "Temporal Clustering of Heroin Overdoses in Washington, DC." Journal of Forensic Sciences (JFSCA). Vol. 34, No. 1, January 1989.

Karel, R. "New Swiss Program Will Distribute Hard Drugs to Addicts." Adapted from the American Psychiatric Association publication Psychiatric News, reprinted in Dialogue on Drugs, the Newsletter of the Concerned Citizens' Drug Study and Educational Society. Vol. 5, No. 1, Spring 1994.

Lavoie, Judith. "Fear, Threats on Streets Outreach Firing Boosts AIDS Risk." Times Colonist, 21 April 1994.

---. "Grief, Anger Mix as Tonto Remembered." Capital Regional News, 27 January 1994.

Le Dain, G. Interim Report of the Commission of Inquiry into the Non-Medical Use of Drugs. Ottawa: Information Canada, 1972.

LeDrew, R. "To Adapt." Prevention Connection, British Columbia Ministry of Health. Vol. 1, No. 7, May 1994.

Loock, C. "Prevention of Alcohol-Related Birth Defects Fetal Alcohol Syndrome." Presentation at Conference to Address FAS and NAS, Vancouver, June 1994.

Loock, C., C. Kinnis, B. Selwood, G. Robinson, S. Segal, J. Blatherwick and R. Armstrong. "Targeting High Risk Families: Prenatal Alcohol/Drug Abuser and Infant Outcomes." Department of Paediatrics, Faculty of Medicine, University of British Columbia, 1993.

Lovett, A.R. "Wired in California." Rolling Stone, May 1994. 39 - 40.

MacPhee, B. "Fetal Alcohol Syndrome: Education and Prevention." The Canadian Journal of Ob/Gyn & Women's Health Care. Vol. 4, No. 6, 1992.

Maddin, D. "Heroin, We Must be Willing to Make a Hard Decision." The Vancouver Sun, Friday, June 16, 1978. p.4. Reprinted in Dialogue on Drugs, the Newsletter of the Concerned Citizens' Drug Study and Educational Society. Vol. 5, No. 1, Spring, 1994.

"MAGLOCLEN, International Heroin Conference Special Report, June 1993." Tori, S.P. Report on the Middle Atlantic - Great Lakes Organized Crime Law Enforcement Network, The National Drug Intelligence Center, The U.S. Drug Enforcement Administration, and the U.S. Drug Enforcement Administration, and the U.S. Customs Service International Heroin Information Sharing Conference, April 1993.

McMullen, David. "OD Deaths Shouldn't Be Hushed." Times Colonist, 13 December 1993.

McNicoll, A. "The Case Against the Decriminalization of Cannabis." A special report for law enforcement agencies, 1978.

Memorial Service for Lives Lost in the War on Drugs. BC Anti-Prohibition League, 1993.

Middleton, Greg. " 'Tough Guy' Met Drug Vengeance." Province, 30 January 1994.

Miller, J. and R. Fisk. "Epidemiology Report on Illicit Drug Deaths." BC Ministry of Health and Ministry Responsible for Seniors. Communication with Chief Coroner, (unpublished) May 1994.

Mitic, W. "Just the Facts?" Prevention Connection, BC Ministry of Health. Vol. 1, No. 7, May 1994.

Moore, M. "Chemical Dependency, An Overview." Presentation at Conference to Address FAS and NAS. Vancouver, June 1994.

Morris, Barry. "Welfare Reform Attacks Poor." Letter to Editor, Vancouver Sun, 31 January 1994.

Murphy, E. The Black Candle. Toronto: Coles, 1922/73.

Nadelmann, E. and J.S. Wenner. "Toward a Sane National Drug Policy." Rolling Stone, 5 May 1994. 24 - 26.

Nahas, G.G. "Breakthrough Research Links Illicit Drugs with Gene Impairment." PRIDE International, 1992.

National Institute on Drug Abuse for the Office for Substance Abuse Prevention. "Cocaine/Crack The Big Lie." Rockville, MD: National Clearinghouse for Alcohol and Drug Information, 1985.

Netherlands, Ministry of Welfare, Health and Cultural Affairs. "The Netherlands Drug Policy." ICPR, September - October 1991.

"No Quick Fix - Society Still Dithering While Heroin Keeps Killing." British Columbia Report, 14 June 1993.

Oscapella, E. "AIDS, Intravenous Drug Use and Criminal Justice Policy." September 1993.

---. "Witch Hunts and Chemical McCarthyism: The Criminal Law and Twentieth Century Canadian Drug Policy." Notes for an address to the Society for the Reform of Criminal Law Conference - 100 Years of Criminal Codes, Ottawa, June 1993 and to the Canadian Congress on Criminal Justice Congress '93, Quebec City, October 1993.

Parfitt, B. "The Adolescent with FAS in the Criminal Justice System." Presentation at Conference to Address FASS and NAS. Vancouver, 1994.

Parker, J. "The Junk Equation - Heroin." Alcohol and Drug Programs, Ministry of Labour and Consumer Services. Temp, Arizona: D.I.N. Publication 1989.

Peat Marwick Stevenson Kellog. "Comprehensive Review of Detoxification Facilities in British Columbia." BC Ministry of Health, Alcohol and Drug Services, 1993.

---. "Review of Supportive Recovery Homes in British Columbia." BC Ministry of Health Alcohol and Drug Services, 1993.

"Police Crack Columbia Drug-Smuggling Cartel: Millions of Dollars Worth of Assets Seized." Associated Press as reported in the Vancouver Sun, 27 February 1993.

Preston, Brian. "The Damage Done." Vancouver Magazine, March 1994.

Professional Guide to Diseases, 3d ed. Springhouse, PA: Springhouse Corporation, 1989.

Pynn, Larry and Robert Sarti. "Children of Poverty." Special Report of the Vancouver Sun, 7 May 1994.

Rangel, C. "Arguments Against Legalization Drug Abuse Update." September 1988.

The Red Book 1992. Vancouver: Information Services, May 1992.

Redd, Ellen. "Frightening Rise in Cocaine-Linked Sudden Death." Medical Post, 22 July 1986.

Riley, D. "The Policy and Practice of Harm Reduction." Canadian Centre on Substance Abuse, Policy and Research Unit and University of Toronto, Department of Behavioural Science. n.d.

Robertson, Anthony. "Tragedy Fuels Search in Drug Labyrinth." Province, 3 August 1994.

Rothon, Diane. "HIV Prevalence in Adult Correctional Centres in British Columbia." Research Paper: BC Ministry of Attorney General, February 1994.

Ruel, J.M. and C.P. Hickey. "Diversion of Methadone: What are the Risks?" B.C. Medical Journal. Vol. 35, No. 6, June 1993, 420 - 421.

Sabbag, R. "The Cartels Would Like a Second Chance." Rolling Stone, 5 May 1994. 35 - 37, 43.

Sabourin, S. "Drug Money." ICPR, July - August 1991.

Sarti, Robert. "Life on the Street No Treat for Teens, Study of 600 Homeless Kids Reveals." Report on McCreary Centre Society Study. Vancouver Sun, 17 May 1994.

Schmoke, K. "Side Effects." Rolling Stone, 5 May 1994. 38 - 39.

Schuchard, M.K. "Marijuana Use Has High Correlation with Violent Death in Sweden." Atlanta, GA: PRIDE International, 1993.

Schuckit, M.A. "Methadone Maintenance: Is It Worth The Price?" Drug Abuse & Alcoholism Newsletter. Vista Hill Foundation. Vol. 21, No. 4, August 1992.

Schwartz, G.R., P. Safar, J. Stone, P.B. Storey and D.K. Wagner. Principles and Practice of Emergency Medicine. 2d ed. Philadelphia, PA: WB Saunders Co., 1986. 1744 - 1757.

Seguin, Rheal. "Waste in Drug Fight Assailed: Quebec report criticizes conflicting messages on abuse." Quebec Bureau, as reported in the Globe & Mail, 29 January 1993.

"Single Sees Trend to Unified Substance Abuse Approach." Canadian Centre on Substance Abuse. News Action, Ottawa: CCSA - CCLAT. Vol. 1, No. 6, December 1990.

Skirrow, J. "Substance Abuse: Time for Change." Canadian Journal of Public Health. Vol. 85, No. 1, January/February 1994. 10 - 11.

---. "The Dutch Experience, Part I: 'Observations of a Drug Policy Dedicated to harm reduction'." News Action. Ottawa: Canadian Centre on Substance Abuse Newsletter. Vol. 1, No. 6, December 1990.

Slavik, W. and R. Faist. "Alcohol and Drugs in Alberta, Perspectives on their Use and Effects." Alberta Alcohol and Drug Abuse Commission, June 1993.

Stedman's Medical Dictionary, 25th ed. Baltimore, MD: Williams & Wilkins. 1990.

Steinberg, N. "The Law of Unintended Consequences." Rolling Stone, 5 May 1994. 33 - 34.

Streissguth, A.P. "Prevalence of Substance use and Long Term Outcomes of Prenatal Exposure to Alcohol/ Cocaine." Notes from presentation at Conference to Address FAS and NAS, Vancouver, June 1994.

Streissguth, A.P. "The 1990 Betty Ford Lecture, What Every Community Should Know About Drinking During Pregnancy and the Lifelong Consequences for Society." Substance Abuse. (1991) 12 (3), 114 - 127.

Streissguth, A.P., J. M. Aase, S.K. Clarren, S.P. Randels, R.P. LeDue and D.F. Smith. "Fetal Alcohol Syndrome in Adolescents and Adults." Journal of American Medical Association, (1991). 265(15), 1961-1967.

Streissguth, A.P., T. Grant, C.C. Ernst and P. Phipps. "Reaching Out to the Highest Risk Mothers: The Birth to 3 Project." (Tech. Rep. No. 94 - 12). Seattle: University of Washington, Fetal Alcohol & Drug Unit, 1994.

Thornton, K.R., H.J. Will, F.G. Heywood, D.A. Booth, J. Comfort, A. Till, S. Hill and L. Brownlee. "Report on the Actual Hospital Cost of Clearly Identifiable Alcohol and Drug Dependencies." Province of British Columbia, Ministry of Labour and Consumer Services, Alcohol and Drug Programs, February 1990.

Trebach, A.S. and J.A. Inciardi. "Legalize It? Debating American Drug Policy." American University Press Public Policy Series, 1993.

"UK Doctors Back Limited Medical Use of Marijuana." Vancouver Sun, 31 January 1994.

United States. Department of Justice, Drug Enforcement Administration. "Domestic Monitor Program, Drug Intelligence Report, January - March 1993."

---. Department of Justice, "Domestic Monitor Program, 1992 Annual Summary." June 1993.

---. Department of Justice, "Domestic Monitor Program, 1991 Annual Report." July 1992.

---. Department of Justice, "Report From the Domestic Monitor Program (July - September 1992)."

United States National Institute of Justice. "Drug Use Forecasting, Fourth Quarter 1991." Research in Brief, March 1993.

Vickers, J.D. "The Adolescent with FAS in the Criminal Justice System: The Criminal Justice System." Summary of remarks at Conference to Address FASA and NAS, Vancouver, 1994.

Walker, Michael. "Expense of Controlling Drugs Defies Logic." Vancouver Sun, 30 July 1993.

---. "What I Really Said About Drugs." 1993 Communication, The Fraser Institute.

Wilkinson, F. "A Separate Peace." Rolling Stone, 5 May 1994. 26 - 29.

Wilkinson, P. "The Young and the Reckless." Rolling Stone, 5 May 1994. 29 - 32.

Witkin, Gordon. "How Politics Ruined Drug-War Planning." US News & World Report, 22 February 1993.

---. "A New Assault on Cocaine." US News & World Report, 11 January 1993.

Queen's Printer for British Columbia©
Victoria, 1994
