# Supplementary material for: Fighting addiction's death row: British Columbia Supreme Court Justice Ian Pitfield shows a measure of legal courage
Source: Harm Reduct J. 2008 Oct 28;5:31. doi: 10.1186/1477-7517-5-31 (PMC2611970; doi:10.1186/1477-7517-5-31)
Supplement: Additional file 3 — Health impact of injection drug use and HIV in Vancouver. A report on the epidemic of injection drug use in the Vancouver area commissioned for the local health authority. [file 1477-7517-5-31-S3.pdf]

825422

39

HEALTH IMPACT

OF

INJECTION DRUG USE

AND HIV

IN VANCOUVER

This report was prepared by Dr. Elizabeth Whynot on request of Vancouver's Medical Health Officer, Dr. John Blatherwick, and Dr. Anne Vogel. It was prepared with the assistance of Margaret Powell, and benefitted greatly from input of various people including: Laurie Webster, Maureen Lunsford, Evanna Brennan and Susan Giles, Stella Tsang, Dr. Alastair McLeod, Dr. David Patrick, Lou Demerais, and Carole Brown.

May 6, 1996

TABLE OF CONTENTS

		Page
I.	INTRODUCTION	1
II.	RECOMMENDATION SUMMARY: MEDICAL HEALTH OFFICER'S REPORT	2
III.	CURRENT VANCOUVER INFORMATION RE IDU AND HIV	4
IV.	RECENT AND CURRENT PLANNING AND RESEARCH INITIATIVES	9
V.	COMMUNITY CONSULTATIONS SUMMARY	14
VI.	CURRENT INTERVENTIONS AND GAPS	16
VII.	RECOMMENDATIONS, EXPANDED DISCUSSION	27

I. INTRODUCTION

Injection drug use in Vancouver is increasingly associated with severe consequences for the users themselves, for their communities, and for service providers. This increase in consequences is a result of many factors: an increasing number of injection drug users, increasing rates of HIV infection in users, changing patterns of injection drug use, increased numbers of mentally ill persons using injection drugs, worsening poverty and homelessness.

Illicit injection drug use has major impacts on the Vancouver health system, including:

- Increasing incidence and prevalence of symptomatic infectious diseases, including HIV/AIDS (> 750 known infections in Vancouver IDUs), Hepatitis A,B, & C skin and blood infections and endocarditis.
- High frequency of drug overdose resulting in significant morbidity and mortality (> 300 deaths in Vancouver in 1994 and 1995).
- Increased hospital and emergency service utilization: HIV-related disease, septicemia, endocarditis, emergency room visits, ambulance responses.
- Fetal exposures with both short term and long term consequences (estimated 30% of infants born to mothers living in Downtown Eastside).
- Increasing pressure on all community level outreach, nursing, and medical services.
- Increasing need for community level palliation and hospice.
- Worsening consequences of associated conditions such as mental illness.

The purpose of this report is to summarize the information about this urgent problem, to describe current efforts to mitigate it, and to provide a number of clear recommendations for the Vancouver Health Board and the Ministry of Health. A short summary of recommendations is presented first, entitled MEDICAL HEALTH OFFICER'S REPORT, MAY, 1996.

II. MEDICAL HEALTH OFFICER'S REPORT: MAY, 1996

The Medical Health Officer presents the following recommendations and background:

A. RECOMMENDATIONS:

- That the Vancouver Health Board identify the prevention and reduction of illness resulting from injection drug use in Vancouver as a priority.

- That the Vancouver Health Board support coordination of funding from bodies and ministries responsible for research, prevention and service provision within Vancouver with respect to injection drug use and HIV/AIDS,

- That the Vancouver Health Board support the Medical Health Officer to provide coordination and leadership in implementing the following objectives:

    1. Reduction of health risks associated with injection drug use in Vancouver:

    - In partnership with Alcohol and Drug Regional Programs to develop a coordinated community substance abuse program for injection drug users in Vancouver, to include both addiction management and drug abstinence alternatives.

    - Discussion with the Ministry of Social Services re reallocation of savings from reduction of benefits for persons with substance abuse problems.

    - Initiation of a process with other provincial ministries, the municipality, the community and clients to develop a plan to increase housing for injection drug users.

    - Support of community development initiatives within the injection drug use community.

    - Continued support of needle exchange and street nurse outreach programs, including increased capacity to reduce risk in needle sharing environments.

    - Development of improved, specialized mental health services and counselling for injection drug using community.

- Partnership with the College of Physicians and Surgeons to develop and implement a more accessible system of methadone maintenance for opiate users in Vancouver, and investigation of other methods of addiction symptom management.

- Development and implementation of safe injection programming

2. <u>To coordinate and improve health, outreach, and residential services for injection users with HIV and other illnesses</u>:

This objective has three components:

1. Enhanced, integrated primary and secondary care

2. 24-hour Care Centre

3. Outreach, Housing, and Hospice

B. BACKGROUND:

The background for these recommendations is included in the following sections of this document. This report summarizes the extensive epidemiologic information and numerous consultations and planning initiatives highlighting the urgency of this problem.

III. CURRENT VANCOUVER INFORMATION RE IDU AND HIV:

1. **How many injection drug users are there?**

We don't know.
Popular estimates: between 5 and 15,000

Basis for estimates:

- "Registered" needle exchange users = 6,000 (approx);
- In any month, 2000 of those registered use exchange;
- Methadone registrants:
    - 1800 in B.C.; often estimated that proportion of methadone to (opiate) users is 1:10. (*Note that currently only 50 of these are registered with a physician practising in Vancouver's Downtown Eastside.)

Can we get a better estimate?

B.C. Centre for Excellence in HIV/AIDS (Dr. Stephanie Strathdee) is investigating the possible application of "capture/recapture" methods to derive a different estimate. This is part of a multi-centre initiative (Canadian Consortium to Characterize Injection Drug Users), which would apply this method to achieve estimates in Vancouver, Montreal, and Toronto. A study like this in Glasgow a few years ago estimated 15,000 IDU's in a city of 500,000.

2. **What do we know about the users?**

User profiles are available from needle exchange. (reports appended): male:female 70:30; 78 % are in their 30's; 25-30% are Aboriginal, etc. There is no information about what proportion of registered users of fixed site are Vancouver residents. About 22% of all needles are exchanged at outreach sites; presumably a high proportion of outreach (mobile site) clients are either residents of or frequent visitors to Vancouver.

3. **What drugs do they use?**

No survey or formal prevalence data are available. However, there are other kinds of information:

- Observations of service providers, e.g., needle exchange, clinics: heroin and cocaine are the predominant drugs of choice; mixed use is common; older age group (40's +) more likely to use heroin alone; drug use trends

vary with " marketing" e.g., how cheap is the drug; alcohol is also a factor for some; cocaine users have a much more chaotic life.

- Alcohol and Drug Services client data base (1994 Vancouver Detox information appended). Selection bias is a big factor in this data.

- Data regarding illicit drugs as cause of or contributing to death (Coroner's charts appended): Heroin and cocaine are the major illicit drugs identified, with heroin more frequently recorded as a direct cause of death, and cocaine as a contributor to death from other causes, e.g., suicide.

- "Point Project" outbreak investigation: both cocaine and heroin use identified, with cocaine use being associated with higher risk behaviour. (The final report of this project is attached in appendix.)

4. **Where (in Vancouver) do they live?**

- No systematic surveys available, therefore information is from service provider observation:

- Highest visibility of IDU's is in Downtown Eastside, with increasingly visible use in Strathcona (along Main Street), Downtown South, Grandview-Woodlands, along Hastings to Hastings-Sunrise; in Mount Pleasant; i.e., most obviously CHC's 1, 2, 3,& 5, but IDU's living in all areas.

- Users in Downtown live in substandard hotels or on the street; point project found correlation between HIV seroconversion and poor housing

- Needle exchange estimates that 20 % of users in Vancouver live in DES (Vancouver Strategic Plan for HIV/AIDS Care).

    - Social housing, Co-ops, etc. have difficulty in accepting IDUs.
    - Cheap, poorly kept apartments in Grandview Woodlands, Hastings Sunrise, Mount Pleasant are housing for many users.

5. **How many Vancouver IDU's are infected with HIV?**

Estimates of this proportion are derived from BC Centre for HIV/AIDS Control testing data (appended):

- It is not known what proportion of IDU's in Vancouver have been HIV tested.

- All testing indicators support the view that the proportion of HIV infected injection drug users is increasing; a conservative estimate of this proportion is between 7 and 12%; it is estimated that there will be 300

newly positive tests in injection drug users in Vancouver in 1996.

- Since the onset of testing (October, 1985), >750 HIV positive tests occurred in persons identifying injection drug use as their only risk factor; an additional 250 positive tests occurred in those reporting IDU and other risk factors related to sexual activities.

- At least 50% of those positive tests have occurred since the beginning of 1994.

- Of approximately 2000 reported AIDS cases in B.C., approximately 100 have been in IDUs. This proportion is likely to increase significantly in the next few years, as seroconversion increases.

- 20-25% of AIDS cases in St. Paul's' hospital report injection drug use as identifiable risk

6. **Why do they get infected?**

This was the question behind the "point project", a case control study (281 participants) to determine factors associated with HIV seroconversion in injection drug users. The point project identified various factors associated with needle-sharing leading to seroconversion:

- Cocaine use
- Frequent injection (associated with Cocaine use)
- Unstable housing; migration
- History of sexual abuse
- Depression

Point project also found that injection drug users who also use drugs by other routes were less likely to seroconvert to HIV.

7. **What else do we know about HIV positive users?**

- 35% are female

- A disproportionately high number are Native; for example, in B.C. in 1995, 21% of new HIV positive tests in women and 10% of new tests in men were from Aboriginal people. (Possible under-estimate; for 20% of clients' ethnicity was not recorded.)

- Some get pregnant: at Sheway pregnancy outreach program, 15% of current clients have volunteered that they are HIV positive.

- Increasing numbers of mentally ill individuals are HIV positive IDUs.

- B.C. Centre for Excellence in HIV/AIDS has received funding for a "longitudinal" study of injection drug users living in or frequenting the Downtown Eastside. The intention is to identify and follow 1000 IDU's for several years and collect information on a number of factors prospectively (VIDUS study; description appended.)

8. **What impact does HIV in IDU's have on health services now?**

- Downtown Clinic: monthly totals of visits by known HIV positive clients has more than doubled in 18 months from 60 or 70 to 140 + (appended).

- St. Paul's hospital estimates 20-25% of inpatients receiving care for HIV illness are IDU's (St. Paul's provides about 75% of AIDS related hospital care in B.C.); Vancouver Hospital estimates 75% of AIDS inpatients are IDU's.

- An estimated 22,000 hospital bed days will have been used in 1995 in B.C. hospitals by people with HIV/AIDS between 4 and 5000 will have been used by IDU's.

- BCCDC street nurses provide primary care to > 200 HIV positive women, mostly users.

- Increasing numbers of HIV positive users are requiring home care and palliative care.

- Estimated cost of provision of hospital care for 1 person with HIV illness in 1 year: $150,000 (1992).

9. **What do we think will happen in next few years** ?

- Increasing numbers of persons now HIV positive will develop HIV related illnesses (poverty, stress, hunger, injection drug use may all hasten progression of HIV-related disease).

- Factors increasing rate of progression in HIV: poverty, stress, hunger, IV drug use.

- Without more active preventive programming (see below), numbers of new infections are likely to continue increasing.

10. **What are the other important health issues related to IV Drug use?**

- Other communicable diseases: hepatitis A, B, C; tuberculosis.

- Mental health problems often coexist, may increase risky behaviour.
- Hospital admissions for other reasons, e.g., septicemia and endocarditis; pneumonia.
- Frequent minor and major injury.
- Support the development and implementation of safe injection programming within Vancouver.
- Overdose, fatal and non-fatal: impact on emergency service system; major cause of death in young people in B.C.
- Substantial effects of prenatal exposures on outcomes for infants.

11. **Is there anything that can be done to reduce health problems associated with injection drugs?**

There is accumulating evidence from other cities that active preventive activities such as harm reduction and health promotion among injection users can reduce health risks:

1. reduction in injecting behaviour : Several programs in the United Kingdom, including Glasgow, Edinburgh, and Liverpool report reduced injecting behaviour with various interventions. (Described in Appendix O, item 14, available on request)
2. reduction in HIV seroprevalence in injection users: See Appendix G for description of successful programming in several cities.

IV. RECENT AND CURRENT PLANNING AND RESEARCH INITIATIVES

1. PLANNING:

(a) **Maternity Care for Substance Using Women and their Infants**:

- Established 1994.
- Members: Health Board Prevention, St. Paul's, B.C. Women's,B.C. Children's Sunny Hill Health Centre; Y.W.C.A. Crabtree, Sheway program.
- Purposes to improve service continuum for substance using women and their children; reduce health impact from antenatal exposures.
- Closer to Home funding: Midwifery outreach, Sunny Hill outreach, B.C. Women's substance abuse program.

(b) **Multiple Access Model and In-Patient Planning Group**:

- Established in early 1995 by St. Paul's.
- Members: Hospital personnel including physicians, nurses, social workers, planners; community agencies including needle exchange, public health MHO, Clinic staff and Home Care, Provincial Street Nurses; some users; alcohol and drug regional programs, Centre for Excellence HIV/AIDS, etc.
- Purpose: To improve coordination and develop proposals and strategies for this population.
- Hospital: Some harm reduction measures (e.g., patient access to needle exchange); medical and nursing protocols to mitigate effects of withdrawal for IDUs admitted to hospital.

(c) **Multi-Diagnosis Planning Group**:

- Established early 1995.
- Members: Agencies and front-line workers from community and hospitals working with multi-diagnosed individuals (injection drug use + psychiatric diagnosis + HIV).
- Purpose: To improve coordination and develop interventions and strategies for this population.

(d) **Detox Working Group**:

- Established by Alcohol and Drug Regional Programs in 1995, completed work in April, 1996.
- Variable membership.
- Purpose: To develop a plan for use of Vancouver portion of funds released when Pender Detox closed.
- Presently awaiting recommendations of a contractor re a

community-designed "sobriety station".

- Process criticized by some agencies on grounds that a sobering facility is only a very small part of what is really needed (See Objective 1 in Chapter II).

(e) **Safe Ride**:

- Established in 1994/1995.
  Members: Vancouver Social Planning; Vancouver Police Department; Health Board; Community agencies such as DERA, Recovery Club, Gastown Business Association; Alcohol and Drug Regional Programs (including detox).
- Purpose: To develop an alternative (to police or ambulance) system for transportation of severely intoxicated individuals to detox facility.
- "Safe Ride" operated by Vancouver Recovery Club, receives short-term funding from Central City Foundation.

(f) **VEADA**:

- Vancouver Eastside Alcohol and Drug Association.
- Consortium of interested individuals and agencies concerned about lack of response to various community consultations (see below, IV-2.) with respect to alcohol and drug problems; formed summer, 1995.
- Very detailed recommendations re increased resources, and their design.
- Group is currently dormant, pending action of Vancouver Health Board.

(g) **Vancouver HIV/AIDS Strategic Plan**:

- Established 1994 by St. Paul's administration: Co-Chaired by St. Paul's and Division of Continuing Care.
- Members: St. Paul's representatives (administration, physicians, nursing; Community Health); Continuing Care, Prevention programs, Community agencies (AIDS advocacy organizations, Downtown Eastside agencies), care providers.
- Purpose: To develop and implement a coordinated strategic plan for HIV/AIDS Care Services in Vancouver.
- Strategic Plan document completed in summer, 1995; implementation groups and sub-committees continue to meet; recommendations re IDUs are included in Chapter II.
- Task Force on management of substance users in Vancouver hospitals, convened by COUTH, are scheduled for Tuesday, April 30, 1996.

(h) **"Planning Proposal for the Provision of Coordinated Chemical Dependency Services in the City of Vancouver" (November, 1995):**

- Prepared by COUTH; Steering group members from Vancouver Health Board; Greater Vancouver Mental Health, Alcohol and Drug Services, Vancouver Hospital and St. Paul's Hospital.
- Summarizes some issues and provides a preliminary inventory of services; some hospital utilization data included.
- Issues identified: Inconsistent mandates, communication barriers, resource lack, limited understanding of harm reduction, lack of awareness, lack of standardization.

(i) **Decriminalization Discussion:**

- A Community meeting (January, 1995) initiated by Downtown Eastside Residents Association and facilitated by Health Department, discussed meaning and potential implications of "decriminalized" or "legalized" prescription of drugs of choice to addicts in the area.
- Attended by police, College of Physicians and Surgeons, Alcohol and Drug, Vancouver Health Department, Community agencies, Community members and consumers, Vancouver City Councillors.
- A variety of points of view, from extreme fear of consequences, to belief that such a program would increase community safety.
- An interest group continued to meet, developed a literature review, summaries of program world wide, and a draft research proposal to examine feasibility of a pilot.
- Summary and draft materials available from E. Whynot; project currently on hold.

(j) **Task Force on Coroner's Recommendations:**

- Established in late 1994 by provincial cabinet.
- Chaired by Dr. John Anderson, Provincial Advisor for Alcohol and Drug Programs.
- Interministerial representation at provincial level.
- Purpose: To examine report of B.C. Coroner on Deaths from Illicit Drug Overdose, and make recommendations re implementation.
- Status of implementation is not known.
- Coroner's recommendations included many in Chapter II, including increased substance abuse programming at community level.

(k) **John Howard Society Future Search Conference Group:**

- Established in 1995.
- Members: John Howard Society of Lower Mainland, Vancouver Health Board, including regional office and Alcohol and Drugs

Regional Programs, Vancouver Police Department and R.C.M.P. Drug Awareness Program, Vancouver Coroner, Community agencies.
Purpose: To use a future search format to develop a substance abuse strategy, consensus in Vancouver; suggested date, November, 1996.
Group meets to plan invitation list, format, etc.

2. **RESEARCH**:

CCENDU:

- "Canadian Community Epidemiologic Network on Drug Use".
- Established in late 1994 under auspices of Health Canada and Canadian Centre of Substance Abuse.
- Purpose is to develop a Canada-wide network of cities collecting and monitoring information on a variety of local indicators re substance misuse towards the improvement of ability to intervene.
- Vancouver group: Drs. A. Vogel and E. Whynot from Health Board, Vancouver Police Department, R.C.M.P. Drug Awareness Unit, B.C. Provincial Coroner, Division of STD/AIDS, Alcohol and Drug Regional Programs, St. Paul's.

** Initiative received approval of its funding in March, 1996; funds will be available to collate information on local indicators for a 1995 report.

St. Paul's Emergency Room Study:

- Received funds through CCENDU initiative and Health Canada to develop a proposal for improving information re impact of substance misuse on St. Paul's emergency department.
- Proposal has been submitted to Health Canada on March 31, 1996; purpose: to determine the proportion of persons using St. Paul's emergency who are substance users, the degree to which substance use is the cause of the admission, and the financial impact on the hospital.

B.C.C.D.C. Enhanced HIV/AIDS Surveillance:

- Ongoing surveillance project of Division of HIV/AIDS to analyze HIV testing data to predict the impact of HIV-related disease in B.C.
- Recent information about numbers of First Nations persons infected with HIV is incorporated in chapter III.
- Some recently collated 1995 information is appended.

Point Project:

- Case control study to determine factors contributing to HIV seroconversion

in a group of IDUs in Downtown Eastside; research completed in summer, 1995; report released in April, 1996.

- Research advisory group includes community agencies, B.C. Centre for Excellence in HIV/AIDS, B.C.C.D.C. Division of STD/AIDS.
- Summary Report is appended; summary data and recommendations have been included in Chapters II and III above.

VIDUS:

- Vancouver Injection Drug Use Study.
- 3 year prospective study of 1000 injection drug users in Vancouver; funded through B.C. Ministry of Health.
- Collaborative project among Community Advisory Board (same as Point Project above), B.C. Centre for Excellence in HIV/AIDs, and B.C. Centre for Disease Control.
- Intake for study to begin in May, 1996; research coordinator has been hired and office has been rented.
- To track HIV incidence and prevalence, and develop recommendations for strategies and interventions to reduce risk for IDUs.

Canadian Consortium to Characterize Injection Drug Users:

- National group of epidemiologists.
- Intention to complete a multi-centre capture-recapture study to estimate numbers of IDU's in Vancouver, Toronto, and Montreal.
- Proposal now in development, to be submitted for funding in July, 1996.

V. SUMMARY OF COMMUNITY CONSULTATIONS:

Safer City Task Force (1992-1993):

- Section 4 deals with "Drugs".
- Extensive discussion about issue of "decriminalization".
- Recommendations included "a research project to summarize the effect of drug use on Vancouver's quality of life and its costs; back to the Health Department, in cooperation with the Provincial Government, to evaluate drug treatment services in Vancouver and develop a comprehensive plan to help people addicted to drugs".

Alcohol and Drug Programs Community Consultation (1992):

- Initiated 1992, evolved into DES/Strathcona A&D Advisory Committee (see below)
- Materials attached in appendix
- Analysis of population demography vs. alcohol and drug funded programs showed services disproportionately allocated to adult males (proportion of allocation for youth has improved somewhat); relevance of existing services to particular groups (women, youth, first nations, IDUs) questioned.

Community Voices: (1994)

- Report completed by Downtown Eastside/Strathcona Alcohol and Drug Advisory Committee.
- Committee initiated by Region 1 Alcohol and Drug Programs in 1992 (see above)
- Committee purpose to discuss how a coordinated community response to alcohol and other drug problems could be developed; subcommittee of DES/Strathcona Coalition.
- "Community Voices" reports on results of 7 focus group discussions with consumers and community members.
- <u>Areas of concern</u>: access for all community residents, prevention, coordination among relevant support agencies and ministries, participation by consumers and community.

Core Women Report: (1995)

- Interviews with 142 women in Downtown Eastside.
- Transition housing, street outreach workers, and alcohol and drug services were the first 3 priorities of this group.
- Community members as service providers, and improved coordination of services are key issues.

Detox Services Report: (1995)

- Contracted by Alcohol and Drug Services, Region 1.
- Purpose to develop recommendations for replacement of detox services in regions previously served by Pender Detox.
- Recommendations about consumer & community input, more diverse and flexible programming, urgency of need to develop alternatives for detox.
- Detox working group (IV-1, above) attempting to implement some recommendations of this report.

VI. CURRENT INTERVENTIONS AND GAPS:

1. PREVENTION AND EARLY INTERVENTION (HARM REDUCTION) ACTIVITIES:

These are defined as activities with the general purpose of reducing health consequences of IV Drug Use.

a) **DEYAS Needle Exchange**:

- Exchanged 1.8 million needles in 1995, and massive numbers of condoms (reports appended);
- Now administered, funded through the Provincial Needle Exchange program (Adult Clinical and Addiction Services).

Problems/Gaps: Very effective program which requires continuing support; should be supported to develop more intense outreach to most marginalized users, eg. hotel outreach program; peer support programming

b) **B.C. Division of HIV/AIDS Prevention Programs**:

i) Street Nurse Program:

- Outreach nurses specializing in sexually transmitted disease education, prevention, and treatment.
- Offices on Richards street, Main Street above needle exchange; also visit jail and do street outreach on foot and in needle exchange vans; some evening service.

Problems/Gaps: This is an effective program doing a large number of STD tests including HIV, distributing condoms, treating many. Nurses provide primary care of STDs, most effective preventive method for curable STDs. The line between prevention and treatment is blurred especially with respect to continuing management of HIV positive clients. Resource base may need to be enhanced if as HIV illness increases, placing too much primary care load on STD nurses. Effective linkage with treatment services is essential.

ii) Street project: Counselling program for HIV positive IDUs.

- Education and support re HIV/AIDS aimed at Latin American population in DES.

- Health promotion approach.

c) **Alcohol and Drug Treatment**:

- Programs include detoxes (Vancouver Detox and Cordova Detox); outpatient clinics (none in Downtown Eastside/Strathcona or Hastings Sunrise); rehabilitation centres (provincial mandates); supportive recovery houses.

- Alcohol and Drug regional programs also funds workers in several outreach agencies including YWCA Crabtree (FAS/NAS Prevention Coordinator), DEYAS, and Youth outreach programs such as Nexus and Odyssey.

- Youth Detox programming has received extensive support in last few years, and is seen as both relevant and accessible; (most recent improvement in this service was initiated by Minister of Social Services as part of the Vancouver Action Plan).

Problems\Gaps : Report after report (Sections IV and V above) have described these gaps eloquently and exhaustively. Accessibility and relevance of programming for IV drug users are huge issues. The experience of agencies serving IDU's is that the current set of A&D services is largely inadequate to the needs of injection users. There is no comprehensive plan regarding development of resources in the area of highest injection drug use concentration, i.e. Downtown Eastside.

d) **Emergency response**: (ambulance; safe ride; emergency departments).

- The emergency response system provides early intervention to prevent overdose deaths, and development of illnesses related to exposure.

- In the last few months, Central City foundation has provided funding for a "safe ride" program, initiated by Police and Social Planning.

Problems/Gaps: Safe ride program needs evaluation plan for continued funding. This program currently exists in isolation; not yet part of a (non existent) substance abuse plan for the high-risk community it serves.

- Emergency response system is expensive; alternative models require consideration.

e) **Symptom Management:**

- Methadone: B. C. College of Physicians manages the B.C. methadone program. It has been actively increasing the number of physicians with methadone licences and has established a users advisory committee.

Problems\Gaps :

i) Methadone: There is only 1 physician with a methadone license practising in the Downtown Eastside.

ii) Methadone is not useful in mitigating symptoms of cocaine withdrawal.

iii) Methadone alternatives: Methadone is not appropriate or acceptable in all situations or for all opiate addicts. Unfortunately, it is the only "substitution" medication approved under federal law for mitigation of withdrawal symptoms. Many public health officials and bodies, including the B.C. Provincial Health Officer and the Canadian Public Health Association have called for a change in this legislation to allow legal prescription of drug of choice such as heroin or cocaine. They point to increasing evidence from other countries, that such prescription can be managed safely and has great potential to reduce many of the consequences of the illicit drug trade. (See Decriminalization discussion in Section IV-1 (i))

iv) Pain management protocols for injection drug users: Pain is a common accompaniment of HIV related diseases. Stress caused by pain is reported to increase drug seeking behaviour by injection users. Practical pain management protocols would reduce stress.

f) **Other community prevention agencies:**

There are a number of agencies advocating and providing AIDS prevention education for particular disadvantaged communities. These include, including:

2. <u>Treatment Services</u>:

These are defined as services providing clinical care for IDU's with HIV, related and contributing problems

a) **Clinics and outreach**:

Downtown Community Health Clinic:

- Most complete medical service in DES, including 3 physicians, a nurse practitioner, practical nurses, pharmacy (can dispense AIDS medication), laboratory linked with St. Paul's, limited dental services; houses tuberculosis outreach nurse, Downtown Community Support Services, subsidized food store.
- Monday to Friday, 8:30 - 4:30 (early closing Wed.)
- Some physician sessions used for outreach to Portland Hotel and to support street nurse clinic for HIV positive patients.
- Clinic coordinator, physicians, College of Physicians and Surgeons beginning discussion of provision of methadone for clinic patients within context of ongoing medical care

Vancouver Native Health Society Clinic:

- Drop-in clinic with 1 physician; houses Sheway and midwifery outreach and limited pediatric follow-up for substance affected infants.
- Monday to Friday, 10:00 am to 8:00 pm; Saturday afternoon clinic.

**The only after hours and weekend medical service in DES.

Street Nurses, STD/Oak Tree outreach:

- Program described above under Prevention.
- Nurses provide the only primary care link for a group of very marginalized women, many with HIV.
- Limited physician back-up, provided by Oak Tree Clinic, Provincial Division of STD/AIDS, and Downtown Clinic.

Downtown South Clinic:

- Established 1995 in new Continental Hotel on Seymour Street; mandate to serve clients in single room occupancy hotels in Downtown South; physician, nurse, alcohol and drug counsellor, and mental health services; some injection users, HIV positive, but minimal overlap with DES; mandate in development.

- Opening hours are Tuesday to Saturday, 12:00 to 8:00 pm.

Services to Substance Using Women and their Children:

- Includes Sheway program (community health nurse, nutrition counsellor, outreach worker, social workers, infant development worker); Midwifery outreach (just beginning) and Sunny Hill outreach (limited pediatrician service and part time nurse clinician); close links with Crabtree FAS/NAS prevention program.

- multi-agency partnership; housed at Native Health, which is a partner.

- All clients current or recent substance users; an increasing proportion are HIV positive

Private practitioners:

- There are approximately 7 family physicians in private practice in the Downtown Eastside. A number of them provide primary care to injection users; however, many are reluctant to make home visits. The proportion of users attending private physicians in preference to clinics is unknown. This is in stark contrast to the 26 physicians in Strathcona, who have few users in their practices.

- At the time of this report, the only physician in DES prescribing methadone is a private practitioner. Arrangements are now being made to train some clinic physicians in methadone prescription.

Tuberculosis Outreach:

- The Division of Tuberculosis Control provides an outreach nurse to supervise treatment for marginalized clients living in or around the DES. The Division has recently received funding to provide an additional nurse to do tuberculosis testing. The TB outreach nurse is based at DCHC.

b) **Home care nursing**:

- A significant amount of home care nursing is provided in the Downtown Eastside, with nurses visiting clients in their "homes", which may be very substandard hotels and rooming houses, or any of the various social or supported housing in the area. Home care nurses provide a clinic twice weekly at the Portland hotel.

- Home care services are not available in the evening in the Downtown Eastside because of safety issues; visits to clients in residences considered unsafe may be limited.

- Provision of increased security measures to staff is being explored.

c) **Hospice/palliation**:

Clients in the DES, including injection users, do qualify for hospice services. However, the circumstances in which many of them live, and the problems of prescribing pain medication for addicted persons, mean that a different model of care must be developed. Some Downtown clinic physician sessions have been allocated to work on this problem with providers in the continuing care hospice program. Provision of increased security measures to staff is being explored.

May Gutteridge Place:

- Only residential hospice in Downtown Eastside; originally designed for general palliation in marginalized community.

- Limited number of beds; palliative patients may be "bumped" because of HIV infected IDUs with more acute illness.

Normandy Lodge:

- Palliative facility for HIV patients; limited ability to provide service for IDUs in need of hospice care because of AIDS-related dementia.

<u>**Treatment Services Continuum Problems\Gaps**</u>:

- Because treatment services are best understood as a whole, the gaps and problems are described from the point of view of the service continuum.

  - <u>Coordination</u>: Current administrative and funding structure makes coordination of planning and resource development in Vancouver difficult. For example, the separate

administrations of the nursing resources, including VHB administered home care nurses, nurse practitioners, community health nurses, the provincially administered street nurses and tuberculosis outreach nurse, and GVMHS mental health nurses make it difficult to understand and plan the prevention/treatment continuum.. There is a similar issue for physicians working for different agencies and in private practice, although there is now a (beginning) coordination strategy.

- Accessibility: e.g., current unit location of home care nurses reduces accessibility and ability to be flexible.

- Safety of all service providers in the Downtown Eastside and other areas is a major concern, reducing service availability.

- Methadone:

  i) As clients become sicker, restrictions of methadone program become punitive. Clients can't get out for methadone, can't pick up methadone, etc. Currently many clients must travel outside Downtown Eastside to find a methadone-prescribing physician.

  ii) Currently, only a few physicians prescribe methadone; patients may therefore receive methadone from one physician, medical prescription from another, and psychiatric medication from a third; or, a physician with a methadone license carries a disproportionate number of very ill patients.

- Workable program for pain management for users.

- After hours, weekend care; only one medical clinic open on Saturday afternoon and some evenings (Native Health).

- Information sharing, liaison: quality of service for clients would be improved if caregivers at various sites through whole continuum of care could share information efficiently

- Medical outreach: limited physician sessions available for "home" visits (application has been made to Alternate Payments Branch to remedy this).

- 24 hour "short stay, safe discharge, safe rest": A number of planning groups have identified the need for such a facility, especially the Multiple Access Model Working Group and the Multidiagnosis Planning Committee (reports appended). The committees have identified various needs to be served by such a facility, including safety for homeless patients, "triage" of medical problems (before attending emergency), transition from hospital to community for patients discharged from emergency, short stay treatments, communication centre for all medical facilities in area, repository of " liaison" data.

- Hospice Lack:

  There are only ten long term care facility beds for AIDS in the province (Normandy); facility largely unable to cope with injection drug users.

3. **Outreach support for injection drug users**:

i) These are defined as one-to-one outreach services aimed at improving capacity of individuals to access needed supports and receive needed care at their place of residence.

Home support services (funded through Continuing Care):

- In addition to Home Care nursing and rehab services, other homemaking and personal support services are also funded.
- Services are provided on the assumption that the client has a "home"; IDUs may live in alleys.

Native Health AIDS Outreach:

- Funded through Adult Clinical Programs; 2 full time workers (practical nurses) providing outreach for >400 HIV +Aboriginal people, many injection drug users.
- Has identified a large number of individuals not previously accessing programs provided through continuing care or MSS.
- Overwhelmed by numbers

St. Paul's/Native Health Liaison:

- Originally funded as hospital/community partnership; 2 social workers to provide liaison services with community (including

home community) for Aboriginal clients admitted to St. Paul's.

Assertive Community Team:

- Service of Greater Vancouver Mental Health, working out of St. Paul's and St. Vincent's Hospitals.
- Outreach to very marginalized clients, many of them with both mental health and injection drug problems.
- Intensive one-to-one support re lifestyle stabilization.

Sheway:

- Described in treatment section; program includes an outreach worker; partnership with Oak Tree Clinic under development to focus this activity particularly for HIV positive women.

Outreach Support Services Problems/Gaps:

- High intensity type of service; all available services currently working at capacity.
- Lack of adequate housing makes access of some funded services impossible, e.g. home support services.

4. **Housing\Residential Care**:

Subsidized and Social Housing:

- Minimally available for injection drug users, especially multi-diagnosed; most facilities will not house more than a few.

Portland:

- Hotel leased by Downtown Eastside Residents Association; housing for more than 70 very marginalized clients, virtually all injection users, a high proportion also with HIV and/or mental illnesses; not a "facility", hence few rules; some service agencies visit, including home care nurses; home support now provided in Portland; Central City Lodge kitchen provides a limited number of meals for residents in the Portland.
- Support from province and municipality has resulted in plans to build a new Portland, time line for opening about 1 year; LTC has arranged extended home support for at least 6 units in new building, so possibility for palliative care

- Increasing numbers of IDUs essentially homeless; existing and injecting in highly unsatisfactory conditions.

Problems\Gaps:

- Constant discrimination against injection users in all DES housing (except Portland). There are many examples of recently developed social housing with rules specifically prohibiting users or effectively disqualifying them for other reasons. Services and housing tend to become "gentrified". This is an urgent problem, which greatly increases unsafe behaviours. We desperately need another Portland; also housing for women and children.

- Inadequate life skill support systems to assist users in maintaining housing and making use of existing services.

The following recommendation list is based on information provided in the preceding sections, on information collected in other cities with similar problems, and on discussions with front-line providers and injection drug users.

The recommendations are:

- That the Vancouver Health Board identify the prevention and reduction of illness resulting from injection drug use in Vancouver as a priority.

- That the Vancouver Health Board ensure coordination of funding from branches of the Ministry of Health and other bodies responsible for research prevention and service provision within Vancouver with respect to injection drug use and HIV/AIDS. These include the Vancouver Health Board itself (Prevention Program, Continuing Care, and hospitals); Alcohol and Drug Regional Programs and Adult Clinical and Addiction Services Branch (needle exchanges, response to Coroner's Report); Divisions of STD/AIDs, and Tuberculosis Control; Health Canada Research initiatives.

- That the Vancouver Health Board support the Medical Health Officer to provide coordination and leadership in implementing the following objectives:

  Objective 1: Reduce health risks associated with injection drug use in Vancouver.

  To achieve this objective, the following steps should be addressed as soon as possible:

  - In partnership with Alcohol and Drug Regional Programs to develop a strategy to support the implementation of a relevant, accessible, coordinated community substance abuse program for injection drug users in Vancouver:

    To include both addiction management and drug abstinence alternatives; to be developed and managed at the community level by experienced front-line agencies with significant consumer input; intake to be located in or easily accessible to neighbourhoods with the highest concentration of injection drug use, starting with but not limited to the Downtown Eastside; flexible programming to allow consumers to pursue treatment outside of areas of risk; to have programs appropriate and accessible for particular groups of consumers particularly First Nations peoples and women.

nurse clinic, Downtown clinic, Native Health, Downtown South, Richards Street, Drake)

- Improve outreach sites for service delivery, e.g. Portland

- Continue to develop safe community sites for provision of intravenous drug therapies

2. <u>24-hour Care Centre</u>

♦ Support establishment of a working group to develop a proposal for a 24-hour (health) care centre in the Downtown Eastside.

  - To work from several recent consultations which have identified the need for a 24-hour health care site in the Downtown Eastside (Multiple access Model Working Group, Multi-diagnosis Planning Group, Detox working Group).

  - To consider nature of service to be provided, capacity to address a variety of needs, possible locations, integration and communication with existing services, etc.

3. <u>Outreach, Housing and Hospice Plan</u>

♦ Develop a support service and housing plan for HIV infected IDU's, with or without mental health or other problems.

  - Planning for housing to include users, community agencies, continuing care, housing, social services, and municipal representatives (could city-owned hotels become a part of this strategy?)

  - To include increased hospice capacity for IDU's; consider a resource specific for marginalized injection drug users, with in-house clinical and outreach staff.
