# Supplementary material for: Fighting addiction's death row: British Columbia Supreme Court Justice Ian Pitfield shows a measure of legal courage
Source: Harm Reduct J. 2008 Oct 28;5:31. doi: 10.1186/1477-7517-5-31 (PMC2611970; doi:10.1186/1477-7517-5-31)
Supplement: Additional file 4 — HIV, Hepatitis, and injection drug use in British Columbia: pay now or pay later? A report on the population health impacts of injection drug user by British Columbia's Chief Medical Health Officer. [file 1477-7517-5-31-S4.pdf]

HIV, HEPATITIS, AND INJECTION DRUG USE IN BRITISH COLUMBIA

– PAY NOW OR PAY LATER?

NOTE:

This report was created in a software product that did not support conversion to pdf files.

The graph shadings in Figures 3, 7, and 13 do not appear as in the published version.

To obtain a hard copy of this report, please complete a Publication Order Form.

HIV, HEPATITIS, AND INJECTION DRUG USE IN BRITISH COLUMBIA

– PAY NOW OR PAY LATER?

Dr. John S. Millar
Provincial Health Officer

June 1998

Copies of this report are available from:

Office of the Provincial Health Officer
B.C. Ministry of Health
3rd Floor, 1810 Blanshard Street
Victoria, B.C. V8V 1X4
Canada
Telephone (250) 952-0876
Fax (250) 952-0877
http://www.hlth.gov.bc.ca/pho/

Canadian Cataloguing in Publication Data
Millar, John S.
HIV, hepatitis and injection drug use in British Columbia

ISBN 0-7726-3612-5

1. HIV infections – Transmission – British Columbia – Prevention. 2. Hepatitis – Transmission – British Columbia – Prevention. 3. Intravenous drug abuse – Complications – British Columbia. 4. Narcotic addicts – Diseases – British Columbia. 5. Narcotic addicts – Services for – British Columbia. I. British Columbia. Ministry of Health and Ministry Responsible for Seniors. II. Title.

RA644.A25M54 1998 614.5'99392'009711 C98-960194-3

Preface

The Provincial Health Officer is required by the *Health Act* to report independently to British Columbians on their health status, on health issues, and on the need for legislation, policies, and programs that will improve the health of the population. In addition to producing an annual report, the *Health Act* states that reports should be issued from time to time in the manner the Provincial Health Officer considers most appropriate.

Health Goals for British Columbia were officially announced in March 1998. Goal 6, "reduce preventable illness, injury, and premature death", includes a specific objective to reduce deaths from the use of illicit drugs. This report provides information on the progress being made to address the epidemic of deaths and disease resulting from injection drug use in British Columbia. Recommendations are made on measures that should be taken to pursue the provincial goal and objective related to illicit drugs.

The problem of drug abuse will never be entirely eliminated, but certainly the harm and costs resulting from injection drug use can be greatly reduced through cooperative efforts to create and implement a well-designed and adequately funded plan. The recommendations in this report are intended to improve public understanding of the problem of drug addiction and to facilitate development of a comprehensive, province-wide strategy.

The epidemic of deaths and infections related to injection drug use is not the sole responsibility of any one agency or level of government. Solutions require coordinated action involving several provincial ministries (Health, Children and Families, Education, Human Resources, Attorney General, Municipal Affairs & Housing, Aboriginal Affairs), the federal government, municipal governments, police forces, health professionals, social workers, teachers, street outreach workers, community agencies, volunteers, and others. Injection drug users themselves must be included in the planning process.

John S. Millar, M.D.
Provincial Health Officer

Executive Summary

What Do We Know about Injection Drug Use in British Columbia?

For the past decade, British Columbia has had an epidemic of deaths and disease related to injection drug use (IDU). Overdose from IDU has become the leading cause of death for adults age 30-49 in this province, with more than 300 deaths annually. The leading cause of new cases of HIV infection is now IDU, and we have epidemics of hepatitis B and C related to IDU as well.

While this epidemic is centred in the downtown eastside of Vancouver, it is a province-wide problem requiring a provincial strategy. The outbreak is a symptom of broader problems – child abuse and neglect, drug use, untreated addiction, poverty, and marginalization – that need attention throughout British Columbia.

Drug dependence is a chronic, relapsing medical condition; in this, it resembles other chronic conditions such as high blood pressure, diabetes and asthma. Making proven treatments available for injection drug users – with the compassion, respect and adequate care they deserve – will help them to recover from or cope with their addiction. Adequate, appropriate treatment for IDUs has been shown to provide individual and societal benefits through reductions in the spread of communicable diseases and the criminal activities associated with traffic in illicit drugs.

The continuation of this epidemic represents a failure of societal values and attitudes. It is also a continued major cause of avoidable death and disease, leading to the waste of almost $100 million in direct government costs annually in British Columbia.

What Should Be Done?

Since the Chief Coroner's report on narcotics-related deaths was released in 1995, harm reduction services such as needle exchange, street nurse programs, and methadone maintenance have been expanded significantly. These efforts are a good start, but the health and social problems associated with injection drug use are still with us, and a number of service gaps and opportunities remain.

This report shows that for a modest annual expenditure, British Columbia could greatly reduce the death, disease, and criminal activity associated with injection drug use. $6 million invested in expanding methadone availability could save as much as $30 million annually in direct government costs.

Methadone alone is not enough – a full system of care is needed. Furthermore, methadone is effective for heroin addiction, but not for drugs such as cocaine. The epidemic of IDU in British Columbia is now being driven more by the injection use of cocaine than heroin. Therefore, other treatment options will be needed for non-heroin users.

To truly address the root causes of addiction and particularly IDU, there must be greater commitment to primary prevention. This requires concentrating on early child development and addressing the larger issues of poverty, unemployment, illiteracy, inadequate housing, mental illness, social isolation, violence and abuse, discrimination, and crime.

Health Goals for British Columbia were officially announced in March 1998. Goal 6, "reduce preventable illness, injury, and premature death", includes a specific objective to reduce deaths from the use of illicit drugs.

The Ministry for Children and Families has also established outcome objectives to reduce the rate of drug-induced deaths.

To pursue these provincial goals and objectives, the following actions are recommended:

- Establish one authority in British Columbia, such as a Substance Abuse Commission, responsible for developing and implementing a comprehensive provincial substance abuse strategic plan to include a vision, clear goals, objectives, measurable outcomes, and intersectoral strategies for reducing substance abuse.

- The Substance Abuse Commission should report annually on the inputs, outputs, and results of addiction services, as measured against the objectives and indicators in its strategic plan.

- The provincial substance abuse plan should address the following:

  - Improved public understanding of addiction.

  - Improved quality of care for all children as a primary prevention measure for preventing addition later in life.

  - Comprehensive programs to educate children about substance abuse and to help children develop self-esteem and skills for making responsible decisions about substance use.

  - Improved mental health services.

  - Improved social services for IDUs, including housing, street outreach, and needle exchange.

  - Enhanced and coordinated addiction services, including increased methadone availability to serve an additional 1,500 heroin users (1,000 in Vancouver and 500 elsewhere) and a 50% increase to detoxification, residential care, and counselling for non-heroin IDUs.

  - Pilot testing of controlled legal availability of heroin in a tightly controlled system of medical prescription.

  - Reduced incarcerations for possession of controlled substances. Pilot testing of the "drug courts" process used in the United States.

  - Improved data and information systems to allow for better accountability regarding the numbers of addicts served and the costs and outcomes of intervention.

CONTENTS

Executive Summary i

Introduction 1

Provincial Health Goals 1
Context 1
What Do We Know about Injection Drug Use? 2
Why Do We Have This Epidemic? 3
Why Should There Be Provincial Action? 4

Epidemiology 5

How Severe Is the Problem? 5
What Proportion Of IDUs Are Infected? 8
Is the Problem Limited to Vancouver? 9
Economic Burden 10

What Should Be Done? 13

The Organization Of Addiction Services 13
Quality Child Care, Mental Health Services, And Housing 14
A Harm Reduction Approach 15
Increasing the Availability of and Accessibility To Addiction Services 16
A Drug Court Program 18

What Is The Business Case for Expanding Addiction Services? 19

Methadone Therapy 19
Expanded Detoxification, Residential Care and Rehabilitative Counselling Services 20
Priorities for Expanding Services 20

Conclusion 21

APPENDIX

A: Acknowledgements 23
B: References 24
C: Health Region Data 26

1. Introduction

Purpose and Scope

Health Goals for British Columbia were announced by the Minister of Health on March 9, 1998. Goal 6 is to "reduce preventable illness, injury, and death" and includes an objective to reduce the number of deaths related to illicit drug use (B.C. Ministry of Health, 1997). The Ministry for Children and Families, in *Measuring Our Success*, has also set specific objectives to reduce the rate of drug-induced deaths among children, youth, and adults (B.C. Ministry for Children and Families, 1997).

This report provides information on the progress being made to address the epidemic of deaths and disease resulting from injection drug use in British Columbia. Recommendations are made on measures that should be taken to pursue the provincial goals and objectives related to illicit drugs.

This report does not address the issue of reducing the supply of drugs*, nor, in detail, issues related to corrections or specific Aboriginal addiction problems.

* *Studies have shown that treating injection drug users is much more cost-effective than attempting to reduce the supply of illicit drugs. Treatment also reduces demand among the heavy users who fuel the drug trade.*

Provincial Health Goals

In March 1998, health goals for British Columbia were officially announced. The six goals, approved by Cabinet, go beyond health services and include the many social, economic, and environmental factors that affect our health. To help implement the goals, there are 44 specific objectives and indicators for tracking progress. One of the objectives is "to reduce deaths from use of illegal drugs".

Copies of the health goals document are available from the Office of the Provincial Health Officer.

Context

In 1995, a task force headed by British Columbia's Chief Coroner released its findings on the causes and extent of narcotics-related deaths, along with recommendations for a harm reduction approach (Cain, 1994). This Office has also reported on the epidemic of narcotics deaths and the related epidemic of HIV, in Provincial Health Officer's Annual Reports for 1994, 1995, and 1996.

Since the Chief Coroner's report was released, planning and research initiatives have taken place. Services such as needle exchange, street nurse programs, and methadone maintenance have been increased significantly, and a Methadone Patients Advisory Committee has been formed to advise government and those responsible for the methadone maintenance program on consumer issues.

The media, the provincial government, the federal government, the addiction treatment community, and the City of Vancouver have focused on addressing the problem of injection drug use – in the downtown eastside of Vancouver in particular. These efforts are a good start, but the health and social problems associated with injection drug use are still with us, and a number of service gaps and opportunities exist. Injection drug use is a province-wide problem, requiring a provincial strategy.

A provincial HIV/AIDS strategy is being developed, and a 10% ($5.4 million) increase in funding for substance abuse services was announced in February 1998. This present report is intended to assist in the development of these overall strategies.

The epidemic of deaths and infections related to injection drug use is not the sole responsibility of any one level of government.
Indeed, solutions must involve many agencies outside of government.

To make improvements will require a collaborative effort involving several provincial ministries (Health, Children and Families, Education, Human Resources, Attorney General, Municipal Affairs & Housing, Aboriginal Affairs), the federal government, municipal governments, police forces, health professionals, social workers, teachers, street outreach workers, community agencies, volunteers, and others. IDUs themselves must be included in the planning process.

The development of a provincial strategy – with goals, indicators for measuring progress, and good leadership – will be critical.

Terms

Injection drug use (IDU): Injection of an illicit (illegal) drug into a vein or artery. The drugs involved are primarily heroin and cocaine, either alone or in combination with each other.

Injection drug users (IDUs): Regular, frequent users of injection drugs.

HIV: Human immunodeficiency virus, the agent that causes AIDS.

Hepatitis B and C: Viral diseases of the liver. Like HIV, hepatitis B and C can be spread from one infected person to another through sexual contact and needle-sharing.

Methadone: A synthetic narcotic drug. Used as a replacement for heroin and morphine.

What Do We Know about Injection Drug Use?

Substance abuse disorders are like many other chronic illnesses (e.g., diabetes, hypertension, asthma). They are caused by a combination of genetics, human biology, and environmental factors. Early childhood care and socioeconomic circumstances can markedly affect the likelihood that a person with the genetic or metabolic predisposition to contract these disorders will actually go on to develop them.

Addiction is neither a lifestyle choice nor a moral lapse. Research on the brain shows clearly that addiction is a disease of the brain, with measurable and demonstrable changes in brain physiology, chemistry, and performance. These changes can be reversed with appropriate treatment, which is usually a mix of drug therapy, psychological counselling, and social support.

Addictions – like diabetes, hypertension, and asthma – may lead to death if inadequately diagnosed and treated. For most chronic conditions, including injection drug use (IDU), good outcomes require both good patient compliance with treatment and behaviour change such as weight loss, smoking cessation, or regularly taking prescribed medication. Low income, unemployment, concurrent mental illness, homelessness, and lack of social supports make it difficult for patients to comply with treatment.

For chronic conditions, including IDU, there is often no cure. Treatment focuses on amelioration and extending, to the degree possible, the otherwise reduced life expectancy.

IDU differs from other chronic illnesses in several ways. Often, IDUs have an associated mental illness. Because of the illegality and expense of the drugs used, IDUs may have a life on the move with inadequate housing, food, or care. IDUs must use a black market, where the product is of uncertain composition and strength. They may have to engage in criminal activity and may be in and out of the courts and jails. They are often viewed as "difficult to manage" by care providers, and may have behaviours and appearances that are disturbing to others. In short, IDUs are viewed as criminals and derelicts, rather than as persons with legitimate illness.

IDUs – unlike people suffering from diabetes, hypertension, or asthma – are often rejected, discriminated against, and abused because of their condition. This attitude contributes to the genesis and continuation of this epidemic. It underlies the general lack of political will to address the problem in North America.

Our improved understanding of addiction suggests that it is time for this to change and for society to recognize that IDUs – like people with other chronic diseases – are suffering human beings deserving of our compassionate care and respect.

Why Do We Have This Epidemic?

This question has been extensively studied in a number of reports in this province (Cain, 1994; Whynot, 1996; Temporary Advisory Sub-Committee on Narcotics Harm Reduction, 1997; Something to Eat, 1997).

Many drugs – nicotine, tobacco, alcohol, amphetamines, opiates, cocaine, etc. – act directly on the reward centres in the brain. From the earliest times, humans have experimented with and sought out chemical substances which alter their consciousness. For some individuals, psychotropic drug use becomes harmful, and they become dependent or addicted. Studies show that the "pool" of potential addicts may be fuelled by social conditions of poverty and despair.

The precise reasons for using injection drugs – why, when, and how IDUs fall into this trap – are as varied as the users themselves. The drug itself is only one aspect of a much larger set of complex and interwoven health and social problems.

Although many factors contribute to injection drug use, the following are important in explaining the present epidemic of injection drug use in British Columbia:

- Failure to provide optimal environments for young children. Large numbers of children are growing up in deprived circumstances or dysfunctional families, where they may be exposed to poverty, inter-generational drug use, breakdown in family relationships, and physical, emotional, or sexual abuse. Failure to provide optimal conditions during these early childhood years increases the likelihood of developing substance abuse problems later in life.

- Inadequate provision of care for the chronically mentally ill.

- Availability of cheap heroin, often in unexpectedly high concentration, and cocaine for injection use.

- Illicit nature of drug use, forcing users to criminal activity and jail.

- A persistent societal attitude that forces IDUs to the margins of society, where they are compelled to live without adequate housing, food, health care, and social supports.

- Inadequate organization and capacity for addiction treatment for IDUs.

Why Should There Be Provincial Action?

Once it is realized that injection drug users are suffering from a chronic, relapsing medical condition similar to diabetes or high blood pressure, it becomes obvious that they are as deserving of compassion, respect, and adequate care as anyone else. The continuation of this epidemic is a measure of the failure of our society to live up to this value.

The provincial, federal, and municipal governments have already recognized that action is needed, and considerable funding has been provided to address the problem in Vancouver. A <u>province-wide approach</u> is now needed.

There is another reason for a change in public attitudes and actions that goes beyond societal values. IDUs in British Columbia are dying not only of overdose. They are also dying of communicable diseases that are transmitted through sharing injection equipment and unprotected sexual contact. These include HIV, hepatitis B, and hepatitis C. British Columbia has higher rates for these conditions than other Canadian provinces. Because of these communicable diseases, IDUs comprise a source of infection that is leading to increased illness through sexual contact and other routes in non-IDUs in all communities in British Columbia. This puts everyone at risk, particularly the young who may be experimenting, both sexually and with drugs.

There is a further reason for a change in our approach to this problem: the criminal activity needed to purchase illegal drugs affects people in all communities through burglaries, robberies, and other forms of personal and property crime. Moreover, the costs associated with the criminal justice system and the health care system represent a considerable burden on the taxpayer. This "opportunity cost" means that tax dollars are not available for such needed purposes as effective health care interventions, the provision of quality child care, or government debt and deficit reduction.

2. Epidemiology

How Severe Is the Problem?

In 1993, deaths due to illicit drugs, primarily heroin, reached epidemic proportions in British Columbia (Figure 1), and illicit drugs became the leading cause of death in adults age 30-49. The increased number of deaths is thought to have been caused by the availability of unusually pure heroin at very low street prices, which resulted in accidental overdoses (Cain, 1994).

Figure 1 Deaths due to Illicit Drugs, B.C., 1988-1996

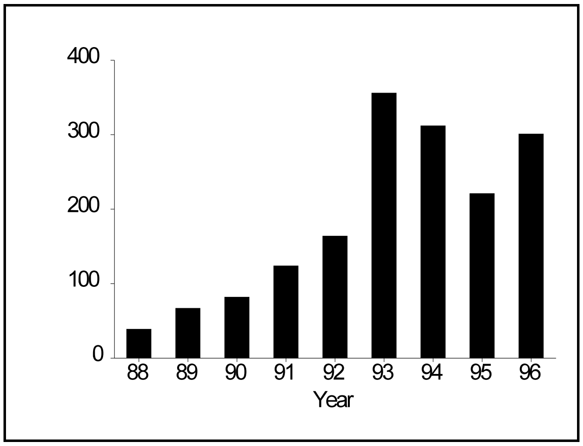

Source: B.C. Coroners Service.

Although the number of illicit drug deaths has since levelled off, this does not mean that the problem of illicit drug deaths is under control. In 1996, 301 illicit drug deaths occurred in this province. In one week recently, there were seven drug-related deaths in downtown eastside Vancouver. The drugs involved are primarily heroin and cocaine, either alone, in combination with each other, or in combination with alcohol or other drugs.

Illicit drugs have surpassed AIDS as a cause of death (Figure 2) and as a cause of premature death, as measured by potential years of life lost (Figure 3).

Figure 2 Deaths due to Illicit Drugs and HIV/AIDS, Ages 30-49, B.C., 1988-1996

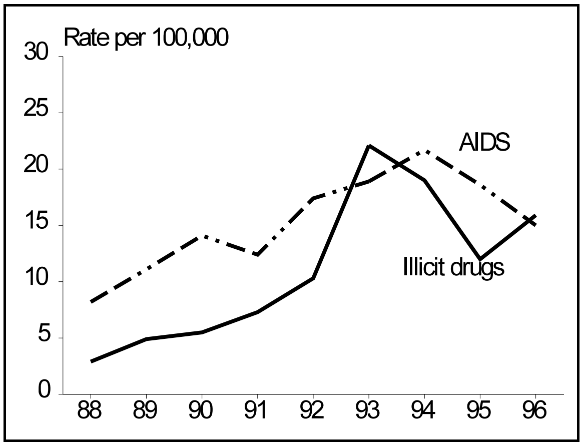

Sources: Illicit drug deaths from B.C. Coroners Service. Deaths due to AIDS/HIV (ICD9 042-044) from B.C. Vital Statistics Agency, Vital Statistics Annual Report 1996.

Figure 3 Potential Years of Life Lost (PYLL, age under 75 years), B.C., 1996

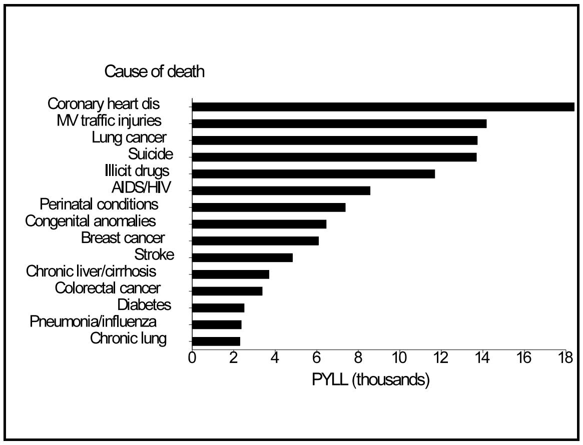

Sources: B.C. Coroners Service (illicit drugs) and B.C. Vital Statistics Agency, Vital Statistics Annual Report 1996 (all other causes).

The British Columbia Centre for Disease Control first reported a growing outbreak of HIV infection among injection drug users in 1994, on the basis of laboratory testing data.

British Columbia's Enhanced HIV Surveillance System – the first of its kind in Canada – confirmed that this increase in positive tests reflected an increase in the number of people with newly acquired infection. Among injection drug users, 338 new infections were recorded in 1995, 387 during 1996, and 253 in 1997.

Although there is still a high rate of new HIV infections among men who have sex with men, their rate of transmission has been declining. In 1994, injection drugs users replaced men who have sex with men as the group in which most new infections occur (Figure 4). Injection drug users now represent about half of the new HIV infections recorded.

HIV is not the only problem among injection drug users. Hepatitis C, a disease first recognized in 1989, has spread among this group.

The availability of a blood test has greatly reduced the risk of acquiring hepatitis C through blood transfusion. However, disease spread is still occurring, most often due to needle-sharing, with 8,201 cases reported in 1997. Of the newly-identified hepatitis C cases, an estimated 80% are related to injection drug use.

To date, British Columbia has accounted for more than half of all hepatitis C cases reported in Canada, and our rate is currently more than four times the national average (Figure 5). This is due to a higher rate of injection drug use, as well as to more complete reporting in British Columbia than in other provinces.

Figure 4 Number of Persons Testing Newly Positive for HIV, Injection Drugs Users (IDU) and Men who have Sex with Men (MSM), B.C., 1985-1997

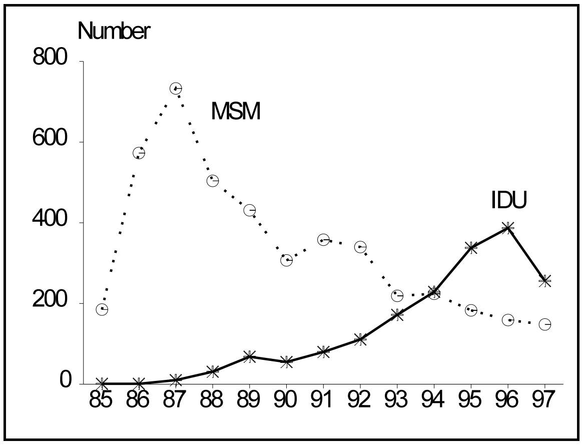

IDU includes the following categories: injection drug use, men who have sex with men/IDU, and sex trade worker/IDU. MSM includes men who have sex with men and women who have sex with women. Source: STD/AIDS Control, B.C. Centre for Disease Control Society.

Figure 5 Reported Rates of Hepatitis C, B.C. and Canada, 1992-1997

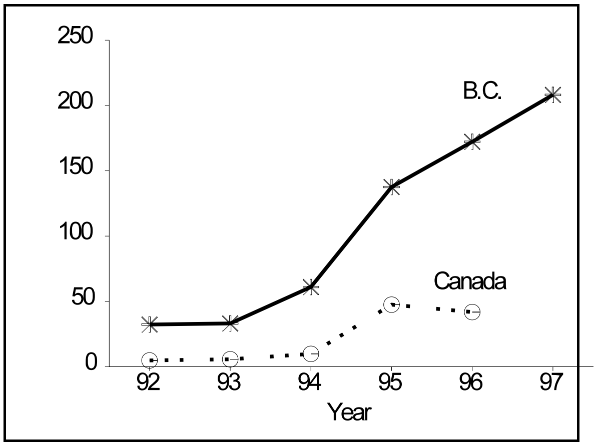

Source: Epidemiology Services, B.C. Centre for Disease Control Society.Unpublished tables, June 1998.

Hepatitis B is another infectious disease that has shown an increase, in part due to needle-sharing associated with injection drug use. In 1980, 16 cases of hepatitis B were reported in British Columbia. By 1990, the number of reported cases had climbed to 915, and B.C.'s rate was almost three times the national average (Figure 6).

In response to the increase in hepatitis B cases, a province-wide immunization program, starting with grade six students, was introduced in 1992/93, along with increased efforts to target individuals in risk groups (IDUs, persons with multiple sexual partners, and household and sexual contacts of chronically-infected persons).

In 1996, the number of new (acute) cases began to decline (Figure 7). As a result of the immunization program, a progressive decline in the rate of new hepatitis B infections can be anticipated. However, hepatitis B will remain a problem for several years to come.

Figure 6 Reported Rates of Hepatitis B, B.C. and Canada, 1986-1997

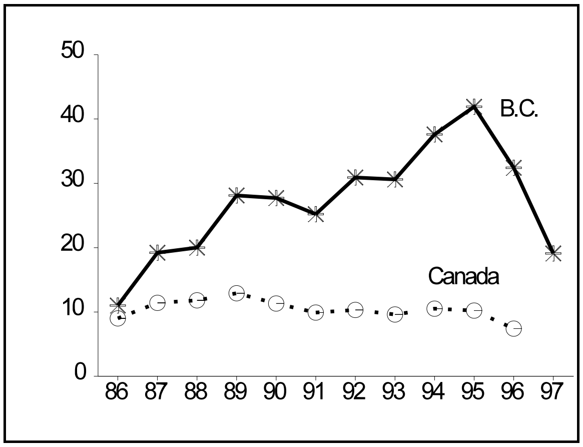

Source: Epidemiology Services, B.C. Centre for Disease Control Society.Unpublished tables, June 1998. Rates for Canada 1986-1996 and for B.C. 1992-1997 are for acute and undetermined cases. B.C. rates 1986-1991 are for total reported cases.

Figure 7 Reported Rates of Hepatitis B, B.C., 1986-1997

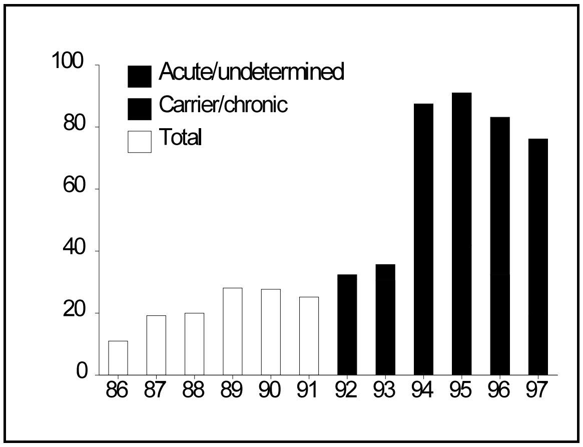

Source: Epidemiology Services, B.C. Centre for Disease Control Society. Unpublished tables, June 1998. Acute cases are persons recently infected. Undetermined cases are persons in whom it could not be determined on the basis of bloodwork at hand whether they were acute or chronically infected; some of these undetermined cases may ultimately be classified as chronic. Breakdowns for acute, undetermined, and chronic cases not available prior to 1992.

What Proportion of Injection Drug Users are Infected?

The number of injection drug users and the proportion who are infected with HIV and hepatitis C are not known precisely. It is estimated that there are about 15,000 regular, frequent IDUs in British Columbia (there are likely many more occasional IDUs). Current estimates are that about one-quarter are HIV-positive, and that most (88%) have hepatitis C (Table 1). These estimates apply to high risk downtown eastside Vancouver users and may not be generalizable to other areas. However, all indications support the view that the proportions are increasing.

A 1995 study, the Point Project, conducted by the B.C. Centre for Disease Control and the B.C. Centre for Excellence in HIV/AIDS, found that four factors in particular were associated with injection drug users becoming HIV-infected:

- Borrowing syringes
- Unstable/poor quality housing
- Frequent injection (four or more injections per day)
- Cocaine use

A consistent finding in the Point and VIDUS projects has been the association of HIV infection with unstable housing – mostly among people living in single room occupancy hotels. These hotels have been shown to play the role of "shooting galleries". Virtually all of this housing is found in a few square blocks around the 100 block of East Hastings Street in Vancouver. The concentration of injection drug users in a small area of Vancouver appears to have resulted from gentrification of other neighbourhoods, concentration of services that are available, and consistent in-migration from across the province and country.

Superimposed on these neighbourhood changes was the sudden increase in injection cocaine use in 1991. The shift from heroin to cocaine as the drug of choice has increased the opportunity for sharing syringes, because cocaine users inject much more frequently than do users of other drugs.

Epidemic modelling shows that such an increase in contact rate between individuals can explain going from a situation of low and stable prevalence to a full-blown epidemic. Comparisons with Montreal, Cape Breton, and other Canadian centres confirm the association of epidemics among drug users with widely-available injection cocaine.

Table 1 Estimated Prevalence of HIV and Hepatitis C among Injection Drug Users, Vancouver B.C., 1988-1997

Estimates	1988-89	1994	1995	1996	1997
Number of injection drug users in B.C.	6,000 to 10,000		5,000 to 15,000		15,000
Injection drug users in Vancouver:					
Percent who are HIV positive [1]	1%-2%	2%	7%		10%-23%
Percent who have hepatitis C					88%
Annual incidence of hepatitis C					26%

[1] Figures for earlier dates are based on voluntary testing, while later figures are from prospective cohort studies. Thus, estimates for the different years are not directly comparable. The 23% is from the Vancouver Injection Drug User Study (VIDUS), conducted by the B.C. Centre for estimate Disease Control, B.C. Centre for Excellence in HIV/AIDS, and a Community Advisory Board. Over 1,000 injection drug users from the highest risk areas of Vancouver are being followed over time to estimate rates of new infection and to determine factors associated with infection or protection.

Is the Problem Limited to Vancouver?

While problems associated with injection drug use are concentrated in Vancouver, all areas of the province have residents newly-infected with HIV (Figure 8), people receiving HIV drug treatment (Figure 9), cases of hepatitis B and C (Figures 10 and 11), and illicit drug deaths (Figure 12).

In the fourth quarter of 1997, newly diagnosed HIV infections outside Vancouver exceeded those in Vancouver, for the first time.

Figure 8 Persons Testing Newly Positive for HIV, Vancouver, Other Metro, and Other Areas of B.C., 1996

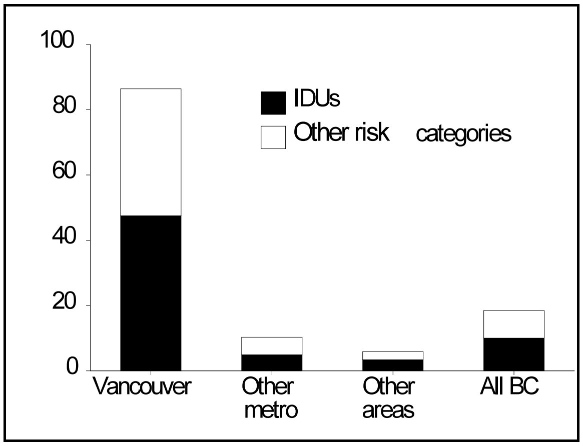

Number of newly positive HIV cases (persons who tested HIV positive for the first time) in 1996, per 100,000 total population. Source: STD/AIDS Control, B.C. Centre for Disease Control Society. Unpublished tables, November 1997.

Geographic regions: In Figures 8-12, data are by region of residence. "Other metro" includes the Simon Fraser, Burnaby, Richmond, North Shore, and Capital health regions. Data for the 20 health unit/health department areas are provided in Appendix C.

Figure 9 Participants in the HIV/AIDS Drug Treatment Program, Vancouver, Other Metro, and Other Areas of B.C., November 1997

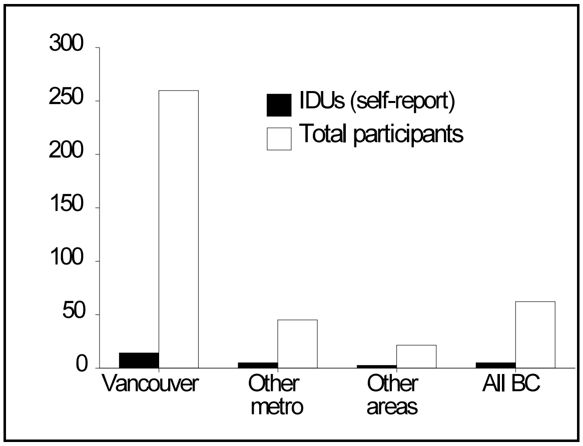

Number of active participants in the HIV/AIDS Drug Treatment program, total and number who report that they are active injection drug users, per 100,000 total population. Source: HIV/AIDS Drug Treatment Program, B.C. Centre for Excellence in HIV/AIDS.

Figure 10 Reported Cases of Hepatitis C, Vancouver, Other Metro, and Other Areas of B.C., 1997

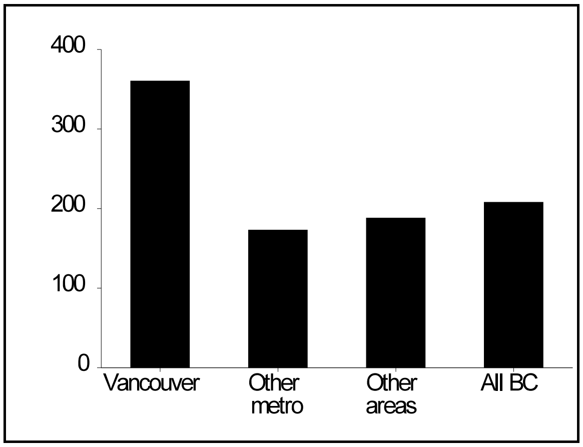

Source: Epidemiology Services, B.C. Centre for Disease Control Society. Unpublished tables, June 1998.

Figure 11 Reported Cases of Hepatitis B, Vancouver, Other Metro, and Other Areas of B.C., 1997

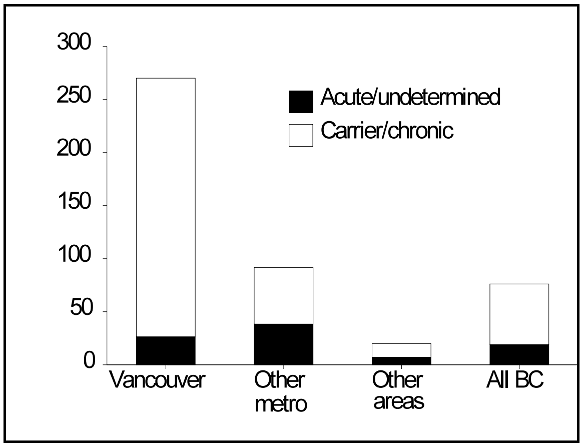

Number of reported cases of hepatitis B. Acute cases are persons recently infected. Undetermined cases are persons in whom it could not be determined on the basis of the bloodwork at hand whether they were acute or chronically infected; some of these undetermined cases will ultimately be classified as chronic. Source: Epidemiology Services, B.C. Centre for Disease Control Society. Unpublished tables, June 1998.

Figure 12 Illicit Drug Deaths, Vancouver, Other Metro, and Other Areas of B.C., 1996

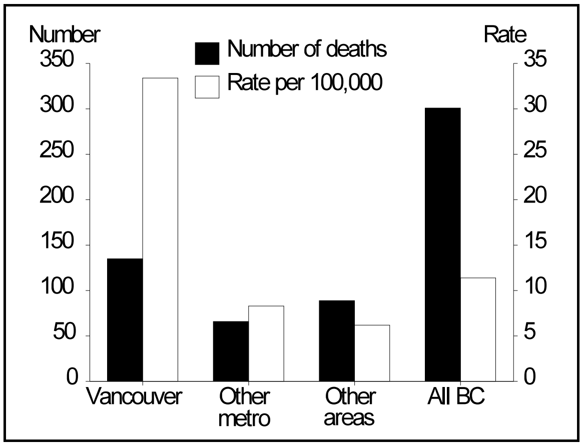

Deaths due to illicit drugs, as determined by the B.C. Coroners Service, by health region of residence, 1996. Rate is per 100,000 population age 15-64. Source: Number of deaths from B.C. Coroners Service, Vancouver.

Economic Burden

Illicit drug use is very costly in British Columbia. A comprehensive analysis by the Canadian Centre on Substance Abuse (CCSA) (1992) estimated that illicit drugs cost the British Columbia economy $209 million annually.

Because tobacco and alcohol are more commonly used than illicit drugs, their total costs to society are higher. Nonetheless, illicit drugs represent a large burden of illness, tend to involve younger victims, and may be more costly per case. According to the CCSA study, British Columbia had the highest per capita cost for illicit drugs – an estimated $60 per capita (Figure 13) in 1992. These costs are potentially avoidable.

Figure 13 Per Capita Cost of Substance Abuse by Canadian Province, 1992

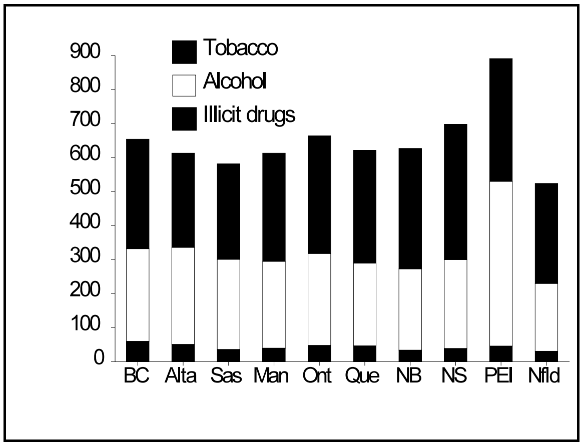

Source: Single, E., Robson, L., Xie, X., & Rehm, J.. The Costs of Substance Abuse in Canada: A Cost Estimation Study. Canadian Centre on Substance Abuse.

To estimate the current direct costs to government of illicit drug use, data from the Canadian Centre on Substance Abuse study have been updated with some actual costs related to injection drug use and HIV/AIDS. These updated figures estimate the direct costs arising from health care and law enforcement at about $96 million annually (Table 2).

Table 2 Estimated Direct Costs to Government of Illicit Drug Use, B.C., 1997

	Est'd Cost ($ thousands)
Direct health care costs	
Hospitalization	$5,172
Co-morbidity	$2,400
Residential Care	$4,854
Non-residential treatment	$1,316
Ambulatory care	$1,458
Prescription drugs	$1,500
Other health care costs	$321
Total	$17,021
Law enforcement	
Police	$37,161
Courts	$20,020
Corrections	$20,020
Customs and excise	$1,508
Total	$78,710
Total direct costs	$95,731
Direct costs per IDU	(dollars)
• Total direct costs per IDU (total of 15,000 IDUs in B.C.)	$6,382

Co-morbidity costs are based on 200 AIDS/IDU cases @ $12,000 per case. Prescription drugs based on 1997 costs for anti-HIV medication for IDUs; data provided by HIV/AIDS Drug Treatment Program, B.C. Centre for Excellence in HIV/AIDS. All other costs based on 1992 data for B.C. in Table 13 of The Costs of Substance Abuse in Canada: A Cost Estimation Study by Eric Single, Lynda Robson, Xiaodi Xie, and Jurgen Rehm, Canadian Centre on Substance Abuse. Estimates were adjusted for the B.C. Consumer Price Index (9.7% increase between 1992 and 1997) and estimated increase in the number of IDUs (25% increase, from 12,000 in 1992 to 15,000 in 1997).

The estimates in Table 2 do not include:

- Health care costs for diagnostic services and preventive programs.
- Costs of treating hepatitis B and C related to injection drug use.
- Costs to society resulting from criminal activity, e.g., theft, property damage.
- Costs related to unemployment and lost productivity, which lead to the need for income assistance, unemployment benefits, or other social assistance costs. Income assistance alone may be costing as much as $67 million annually (see page 18).
- Costs and offsetting savings related to premature death.

Hence, these cost estimates are conservative and provide a very low approximation of direct avoidable costs to government. Of the direct costs in Table 2, about 82% are law enforcement costs, most of which are associated with untreated IDUs. (There are no reliable estimates of the number of untreated IDUs. Of the estimated 15,000 IDUs in B.C., 4,000 are enrolled in methadone therapy; the number receiving other forms of treatment is not known precisely.)

A recent Ontario study based on in-depth interviews with 114 opiate users estimated the annual direct costs to government to be $33,761 per untreated IDU*, as follows (Stewart, 1997):

$29,164 in law enforcement costs

$ 4,597 in health care costs

$33,761 total direct costs per untreated IDU

* *In this study, an untreated IDU was a regular (at least daily over the past week) user of heroin or other opiates, who was not currently in treatment and had not sought treatment in the three months prior to the study.*

3. What Should Be Done?

To reduce deaths, criminal activity, and communicable diseases related to injection drug use, it will be necessary to expand and improve treatment services for IDUs. It will also be necessary to have both a strategic plan and an organization provincially that will ensure that related issues such as early childhood care, mental health services, and social housing are being addressed. Issues regarding the supply and availability of illicit drugs and enforcement actions are important considerations, but are beyond the scope of this report.

The Organization and Planning of Addiction Services

While it is beyond the scope of this report to provide a detailed plan for reorganizing addiction services in British Columbia, some problems with the present organization have been identified, and some solutions are apparent.

Some of the principal problems are as follows:

- Lack of understanding about the problem of addiction, by the public and politicians.
- Lack of coordination, with addiction services funded and provided through different ministries.
- Lack of a coherent, cross-jurisdictional plan with a vision, goals, objectives, measurable outcomes, strategies, and evaluation.
- An inadequate information system and insufficient data to monitor outcomes and evaluate the success of programs.
- Inadequate resources for services needed.
- Lack of application of evidence-based interventions. What "works" is not always provided, due to ideological bias or lack of information. Conversely, what is provided may not be what is known to work.

The Chief Coroner, Vince Cain, in his 1994 report recommended the formation of a Substance Abuse Commission for the province (Cain, 1994). Such a Commission could bring together services that, at present, are administered in separate ministries such as the methadone program in the Ministry of Health and alcohol and drug services in the Ministry for Children and Families.

To ensure that the breadth of issues involved in addictions is addressed, the Commission needs to represent IDUs themselves and those involved in various aspects of substance abuse services: addiction treatment, mental health, early childhood care, health care (HIV, hepatitis B and C), police, housing, social services, education, aboriginal services, attorney general (corrections, parole), and local government.

Such a Commission should have responsibility to develop a strategic plan with a vision, clear goals, objectives, measurable outcomes, strategies, and an information system that enables evaluation and accountability. Building on objectives in *Health Goals for British Columbia* and *Measuring Our Success*, objectives and targets could be set, such as reducing the number of drug overdose deaths to fewer than 50 and newly HIV-positive IDUs to fewer than 200 per year. Reflecting the harm reduction approach addressed below, specific objectives could be set to reduce injection use of drugs, reduce the associated criminal activity, and reduce the occurrence of unprotected sexual contact among IDUs.

The Substance Abuse Commission must be accountable, through the Minister of Health, to government and to the people of British Columbia. It should annually submit a report as to resources used to combat substance abuse in the province and the results achieved as measured against the objectives and indicators in its strategic plan. This report should be submitted to the Minister of Health and be tabled in the legislature.

Recommendation 1: *A Substance Abuse Commission should be established, with responsibility to develop a strategic plan to include a vision, clear goals, objectives, measurable outcomes, and strategies for reducing substance abuse in British Columbia. Goals and objectives that could be included in the strategic plan include:*

- *Reducing the number of drug overdose deaths to fewer than 50 per year.*
- *Reducing the number of newly HIV-positive IDUs to fewer than 200 per year.*
- *Reducing the prevalence and frequency of injection drug use.*
- *Reducing needle-sharing.*
- *Reducing criminal activity associated with injection drug use.*
- *Reducing the occurrence of unprotected sexual contact among IDUs.*

Recommendation 2: *The Substance Abuse Commission should report annually to the Minister of Health on the inputs, outputs, and results of addiction services, as measured against the objectives and indicators in its strategic plan.*

Quality Child Care, Mental Health Services and Housing

It is beyond the scope of this report to comment in detail on these aspects of addressing the epidemic of injection drug use in the province. However, the importance of mental health services, housing and early childhood care must be recognized.

Mental Health Services and Housing

Unless IDUs are provided with stability in their lives, treatments will not be fully effective. This means that there must be an ongoing commitment to providing adequate mental health services and social housing for IDUs.

The Ministry of Health has published its Mental Health Plan (B.C. Ministry of Health, 1998), and services are being improved. The Ministry of Health, Ministry of Municipal Affairs and Housing, the Vancouver/Richmond Health Board, and the City of Vancouver have set a good example of intersectoral collaboration in a recently-announced project to increase the availability of social housing for IDUs in the downtown eastside of Vancouver. These initiatives are a good start.

Quality Care and Education for Children

To truly address the root cause of addiction and particularly IDU, there must be greater commitment to providing quality care and education for young children. Early childhood care is important for all children as a primary prevention measure for preventing addiction later in life, and for at-risk children whose parent(s) are addicted.

The creation of the Ministry of Children and Families has shown the government's commitment to address this problem. The Ministry has begun some pilot projects to determine the most effective ways of providing better care for children in British Columbia. Unfortunately, the Ministry has had to manage

a series of problems related to their mandate to take children into protective custody. This problem is discussed in the Provincial Health Officer's 1997 Annual Report, and some solutions are offered. Among these is a recommendation that British Columbia commit to providing universal access to quality care for all children, whether by a parent or someone else, without financial barriers.

Recommendation 3: *Adequate mental health services, health care, housing, and social support should be provided to IDUs at all stages of addiction and recovery.*

Recommendation 4: *Adopt the principle (modelled on the principles of medicare) that all children in British Columbia should have access to good quality child care without financial barriers.*

Recommendation 5: *Provide school and community education programs dealing with substance abuse, life skills, coping, and parenting.*

A Harm Reduction Approach

The term harm reduction has many meanings. The Canadian Centre on Substance Abuse has suggested the following definition:

> A policy or program directed towards decreasing the adverse health, social, and economic consequences of drug use without requiring abstinence from drug use (Canadian Centre on Substance Abuse National Working Group on Policy, 1997).

There are several components to a comprehensive harm reduction approach:

- Inclusion of users in planning.
- Peer support groups for users.
- Street outreach services.
- Needle exchanges and provision of condoms.
- Mental health services.
- Medical services including HIV and hepatitis testing and treatment.
- Vocational training.
- Nutrition services.
- Adequate housing, clothing, drop-in shelters.
- Youth services.
- Aboriginal services.
- An array of addiction treatment services that includes detoxification, counselling and support of abstinence where desired, maintenance on methadone and other substitution drugs, and the capacity for medically-supervised heroin maintenance.

In a harm reduction approach, comprehensiveness and coordination of programs are crucial. Methadone, like needle exchanges, is not enough. A full system of care should be provided, including a timely assessment that matches clients to the most appropriate treatment regime. For example, while some clients require residential detoxification services, a significant proportion of IDUs can be effectively managed through community-based detoxification.

Coordination of harm reduction services is essential. Linkage between mental health and addiction services is an area of particular concern. Many IDUs have a concurrent mental illness of some type, and substance abuse disorders are common among persons with mental illness.

Co-morbidity (the presence of two or more mental health disorders) is a major issue in mental health services planning, in British Columbia and elsewhere. Because there is considerable overlap in clients seen by mental health and addiction treatment services, coordination is required to ensure a comprehensive, "seamless" system of care.

Many services in the past have been based on an abstinence model. While this is changing, there are still many barriers to effective care because of a carry-over of the old approach. For example, IDUs who are being treated with methadone or being maintained on other drugs may be denied housing or access to mental health services because they are not drug-free.

Although harm reduction services have been expanded considerably in recent years, services remain fragmented and in some cases, inadequate and inaccessible (Health Officers Council, 1998). All components of a harm reduction approach need enhancement. A more detailed discussion follows.

Recommendation 6: *Improve coordination between mental health and addiction services.*

Increased Availability of and Access to Addiction Services

There are about 15,000 IDUs in British Columbia. There are no accurate data on what drugs are being injected, but it appears that some are injecting only heroin, most are injecting heroin and cocaine, and some are injecting cocaine alone or with a variety of other drugs.

Methadone Therapy

Methadone therapy is clinically useful in patients who are using heroin alone or in combination with other drugs. In response to evidence on the effectiveness of this intervention, British Columbia's methadone program has tripled in size in the past two to three years. At this time, approximately 400 physicians are prescribing methadone, and 4,000 are enrolled in methadone treatment. Not all IDUs are seeking this treatment at any given point in time, but further expansion of methadone services is required to meet current needs.

Switzerland, which has a well-developed national strategy for injection drug use, has 2,000 methadone treatment spaces per million population (Fischer & Rehm, 1997). Applying these estimates to British Columbia would mean expanding the methadone program to serve a total of 8,000 clients. A number of authoritative reports have recommended that methadone availability be enhanced to provide service to an additional 1,000 clients in Vancouver and 500 in the rest of the province (Whynot, 1996). Such an increase – to 5,500 methadone treatment spaces – would bring British Columbia closer to the service level benchmark established in Switzerland.

It is widely accepted that to be optimally effective, methadone therapy should be accompanied by counselling. Plans to enhance the availability of methadone should include the necessary counselling services.

At present, there are a number of barriers to methadone therapy in British Columbia. First, many methadone clinics charge a user fee. In some clinics, patients are charged $325 per month for clinic services and $70 per month for methadone. Pharmacy fees are also a barrier. While this may be less expensive than illicit heroin, the cost of methadone is clearly prohibitive for many addicts, particularly those who are unemployed.

Not only is this a policy that deters IDUs from seeking therapy, but it is contrary to the spirit of the *Canada Health Act* and to well-established research findings that user fees discourage the use of effective services. User fees may also require IDUs to continue criminal activity to raise the necessary funds. The charging of user fees for methadone therapy should be discontinued.

Heroin IDUs also experience other barriers to methadone treatment. There is a rigorous assessment process, and those accepted into the methadone program must follow requirements for medical supervision, daily visits, and random urine sampling. Some IDUs find these rules and regulations too restrictive. Stringent program requirements, in addition to the discomforts of withdrawal and side effects of methadone, may be a considerable disincentive to seeking care. There needs to be research into the provision of "low threshold" acceptance into the methadone program.

Other Addiction Services

The epidemic of IDU and drug overdose deaths in British Columbia is now being driven more by the injection use of cocaine than heroin.

Methadone is effective for heroin addiction and for IDUs using heroin in combination with other drugs, but not for drugs such as cocaine used without heroin. At this time, there is no clearly effective pharmacological treatment for cocaine addiction. Therefore, other treatment options will also need to be expanded, including detoxification, residential care, maintenance on other drugs, rehabilitative counselling, and alternative therapies such as acupuncture.

Existing services need considerable expanding. A 10% increase in funding for alcohol and drug services was announced by the Ministry for Children and Families in February 1998. However, an increase of 50% or more may be needed. There will also be a need to provide for the use of other innovative treatments such as LAAM[*], as they are demonstrated to be effective.

As with methadone therapy, there are considerable barriers to access for these other services. Residential detoxification facilities, for example, are very limited geographically; the only facility on Vancouver Island is in Victoria.

[*] *Levo-alpha-acetyl-methadol (LAAM) is a drug that acts like methadone, but is administered on alternate days or three times per week in an oral pill form. Studies have shown LAAM to have similar favourable outcomes to methadone, with the advantage of fewer trips to the pharmacy and avoidance of some of the stigmas of methadone (Temporary Advisory Sub-Committee on Narcotics Harm Reduction, 1997).*

Recent research findings from Zurich show that even when there is full methadone availability, there are some heroin IDUs who do not optimally benefit from maintenance on methadone (Uchtenhagen, 1997). For those IDUs who have not responded to methadone or other treatments, medically-supervised heroin maintenance, alone or in combination with methadone, works well to reduce criminal activity, reduce the sharing of injection equipment, increase employment, and improve the physical health of participants. The Swiss study found that controlled heroin administration had a net economic benefit of 30 US dollars per patient per day, largely due to reduced criminal justice and health care costs (Nadelmann, 1998). It is important to note that in the Swiss trial, heroin administration was only part of a comprehensive set of health, social, and psychological interventions provided to participants (Farrell & Hall, 1998).

The implementation of controlled legal availability of heroin, in a tightly controlled system of medical prescription, would require considerable preparation – in public and professional understanding and attitudes and in legislation. Planning for a pilot project should begin now, so that it will be available when methadone is fully available and accessible throughout the province. Such a trial could assess the impact of different drugs and treatment methods, e.g., oral and injectable, on health status, risk behaviours, employment, and criminal activities of participants.

Recommendation 7: *User fees for methadone therapy (clinic fees, pharmacy fees) should be discontinued.*

Recommendation 8: *Controlled legal availability of heroin, in a tightly controlled system of medical prescription, should be pilot tested as an option, as part of a comprehensive harm reduction program.*

A Drug Court Program

In the United States, the drug court program is provided for non-violent offenders whose involvement with the criminal justice system is due primarily to their substance addiction. Rather than going through regular court and being incarcerated, eligible offenders are diverted into a comprehensive treatment program. The treatment program includes:

- Frequent encounters with a therapist for counselling, therapy, and education.
- Monitoring by frequent urine and other drug testing.
- Frequent status hearings before a drug court judge.
- A rehabilitation program including vocational, educational, family, medical, and other support services.

This program has shown very good results in terms of reduced drug usage and abstinence, reduced recidivism, and considerably reduced costs per defendant (Drug Court Clearinghouse, 1996; National Association of Drug Court Professionals, 1997).

Recommendation 9: *Pilot test the drug courts process used in the United States.*

---

4. What Is the Business Case for Expanding Addiction Services?

The important issues of improved mental health services, housing, and early childhood care are addressed elsewhere (Mental Health Plan; 1997 Provincial Health Officer's Annual Report). In this section, the business case for considerably expanding the availability of addiction treatment services is considered.
To develop a business case, the following elements need to be considered:

- Costs of treatment services. For this discussion, the costs of methadone therapy and the costs of non-methadone interventions including detoxification, residential care, and rehabilitative counselling are included.
- The avoidable costs to government – and so to the taxpayer – related to untreated IDUs.
- The effectiveness of treatment interventions.

Methadone Therapy

For any therapeutic intervention to be effective, there must also be provision for such needs as housing, social support, vocational training, and treatment of co-existing mental illness and medical conditions like HIV. Given these, then methadone treatment is recognized as being very effective in achieving the harm reduction objectives of reducing injection drug use and criminal activity and unprotected sexual activity. In some studies, 70-80% of heroin IDUs show significant improvements in these areas.

Maintenance on methadone in British Columbia costs $2,000 annually for methadone alone and about $4,000 annually if methadone is complemented with appropriate counselling and mental health care.

Based on the recent Ontario study of opiate users (Stewart, 1997), the direct cost to government in British Columbia for an untreated heroin IDU is about $30,000 annually.

To expand methadone availability, with the necessary counselling and mental health care, to serve 1,500 additional heroin addicts would cost $6 million annually (1,500 IDUs @ $4,000) and could generate as much as $36 million in savings (1,500 x $30,000 x 80% effectiveness) annually to health care, criminal justice, and corrections services in the province.

Even using a much more conservative estimate of $15,000 for direct annual costs for an untreated IDU, the estimated net savings would be about $12 million annually ($18 million in savings minus $6 million in costs). The actual overall savings would be much greater, of course, because of more employment and productivity and less reliance on social assistance. The Ontario study found that 75% of IDUs were receiving social assistance. For British Columbia, this would amount to $67.5 million annually (75% of 15,000 IDUs receiving $6,000 per year in Income Assistance benefits).

Ultimately, the non-monetary aspects of such an endeavour should be considered: a number of suffering people will be provided with better social circumstances and better health, and a potential source of infection that threatens everyone will be removed.

What is being suggested is a small investment by government standards. It is not often that a health care intervention has so much potential for direct savings to government.

Expanded Detoxification, Residential, and Rehabilitative Counselling Services

The annual cost for an IDU going through the spectrum of care including detoxification, residential care, and rehabilitative counselling is about $20,000 (Table 3). Under optimal conditions, about 50% of clients will show substantial reductions in injection drug use and criminal activity.

In the first year, for a successful client going through the program, the direct costs and the savings probably are about the same and balance out. For subsequent years, provided the client stays "clean", the net annual savings could be in the range of $30,000 per client.

Table 3 Estimated Costs of Treatment, First-Year Costs per Injection Drug User, B.C., 1997

Treatment	Cost per IDU
Outreach	$400
Detoxification services	$1,500
Assessment	$250
Counselling	$1,350
Support recovery	$10,000
Residential care	$3,000
Day programs	$2,000
Follow-up counselling	$500
Methadone services	$2,000
Total	$21,000

Methadone services costs are based on Pharmacare and Medical Services Plan costs for methadone patients in 1996. All other costs are based on estimates prepared by Adult Addiction Services, B.C. Ministry for Children and Families, November 1997.

Priorities for Expanding Services

It is recommended that priority be given to:

- Investing $6 million in expanding comprehensive methadone services.
- Expanding detoxification, residential care, rehabilitative counselling services, and appropriate alternative therapies such as acupuncture.
- Improving data and information systems.

To adequately track the costs and savings will require close attention to government accounting procedures. If this is done in an appropriate and creative way, it should be possible to identify the savings accruing from expanding methadone availability in the first year. These savings could be applied to further expand methadone and other treatments as appropriate in subsequent years.

Recommendation 10: *Drug rehabilitation services should be incrementally increased, so that every injection drug user coming forward for intervention can receive prompt assessment and care. Specific actions should include the following:*

- *Increase methadone availability to serve an additional 1,500 addicted clients (1,000 in Vancouver and 500 elsewhere). Explore options other than physician-only delivery, e.g., nurses.*
- *Increase detoxification, residential care, counselling, and appropriate alternative therapies for non-heroin injection drug users by 50%.*
- *Improving data and information systems to allow for better accountability regarding the numbers of addicts served and the costs and outcomes of intervention.*
- *Incorporate cost-benefit analysis into decisions about policies, programs, and services related to injection drug use.*

5. Conclusion

The problems associated with injection drug use in British Columbia are not showing signs of improving. A cooperative approach is under way to address the problem in Vancouver. However, it is apparent that there is a need to do more province-wide.

Another 1,000 IDU treatment spots are needed in Vancouver, and 500 in other areas of the province. Primary prevention needs to be undertaken through concentrating on early child development and addressing the larger issues of poverty, unemployment, illiteracy, inadequate housing, mental illness, social isolation, abuse and neglect, discrimination, and crime.

The expansion of addiction services and early childhood interventions are cost-effective and have the potential to provide net savings to government. A comprehensive, coordinated provincial strategy as well as regional and community strategies need to be developed.

The problem of drug abuse will never be entirely eliminated, but certainly the harm and costs resulting from drug abuse can be greatly reduced through a cooperative effort to create and implement a well-designed and adequately funded plan.

Recommended Actions

1. A Substance Abuse Commission should be established, with responsibility to develop a strategic plan to include a vision, clear goals, objectives, measurable outcomes, and strategies for reducing substance abuse in British Columbia. Goals and objectives that could be included in the strategic plan include:

   - Reducing the number of drug overdose deaths to fewer than 50 per year.
   - Reducing the number of newly HIV-positive IDUs to fewer than 200 per year.
   - Reducing the prevalence and frequency of injection drug use.
   - Reducing criminal activity associated with injection drug use.
   - Reducing the occurrence of unprotected sexual contact among IDUs.

2. The Substance Abuse Commission should report annually to the Minister of Health on the inputs, outputs, and results of addiction services, as measured against the objectives and indicators in its strategic plan.

3. Adequate mental health services, health care, housing, and social support should be provided to IDUs at all stages of addiction and recovery.

4. Adopt the principle (modelled on the principles of medicare) that all children in British Columbia should have access to good quality child care without financial barriers.

5. Provide school and community education programs dealing with substance abuse, life skills, coping, and parenting.

6. Improve coordination between mental health and addiction services.

7. User fees for methadone therapy (clinic fees, pharmacy fees) should be discontinued.

8. Controlled legal availability of heroin, in a tightly controlled system of medical prescription, should be pilot tested as an option, as part of a comprehensive harm reduction program.

9. The drug courts process used in the United States should be pilot tested.

10. Drug rehabilitation services should be incrementally increased, so that every injection drug user coming forward for intervention can receive prompt assessment and care. Specific actions should include the following:

    - Increase methadone availability to serve an additional 1,500 addicted clients (1,000 in Vancouver and 500 elsewhere). Explore options other than physician-only delivery, e.g., nurses.
    - Increase detoxification, residential care, counselling, and appropriate alternative therapies for non-heroin injection drug users by 50%.
    - Improve data and information systems to allow for better accountability regarding the numbers of addicts served and the costs and outcomes of intervention.
    - Incorporate cost-benefit analysis into decisions about policies, programs, and services related to injection drug use.

APPENDIX A
Acknowledgements

Thanks are owed to Drs. John Anderson, Robin Hanvelt, Rick Hudson, Perry Kendall, David Patrick, Michael Rekart, and Elizabeth Whynot for their review and constructive comments on the first draft of this paper. Thanks are also owed to the following groups and agencies who kindly provided data for this report:

Addiction Research Foundation
Toronto, Ontario

Adult Addictions Services
B.C. Ministry for Children and Families

B.C. Coroners Service
B.C. Ministry of Attorney General

Department of Health Care and Epidemiology
University of British Columbia

Epidemiology Services
B.C. Centre for Disease Control Society

Health Planning Database
B.C. Ministry of Health

Information and Analysis Branch
Information Management Group
B.C. Ministry of Health

Information and Resource Management Branch
B.C. Vital Statistics Agency
B.C. Ministry of Health

Pharmacare
B.C. Ministry of Health

STD/AIDS Control
B.C. Centre for Disease Control Society

HIV/AIDS Drug Treatment Program
BC Centre for Excellence in HIV/AIDS

APPENDIX B
References

Albert, T., & Williams, G. (1997, November). The economic burden of HIV/AIDS in Canada: Summary of the findings and policy implications. *Reflexion*, Number 1, Canadian Policy Research Networks, Inc.

British Columbia. Ministry for Children and Families. (1997, May). *Review of alcohol and drug services in Vancouver*. Vancouver, BC: Vancouver Regional Office, Ministry for Children and Families and Vancouver/Richmond Health Board.

British Columbia. Ministry for Children and Families. (1997, September). *Measuring our success: A framework for evaluating population outcomes*. Victoria, BC: Ministry for Children and Families.

British Columbia. Ministry of Health. (1997, December). *Health goals for British Columbia*. Victoria, BC: Ministry of Health and Ministry Responsible for Seniors.

British Columbia. Ministry of Health. (1998). *Revitalizing and rebalancing British Columbia's mental health system: The 1998 mental health plan*. Victoria, BC: Ministry of Health and Ministry Responsible for Seniors.

British Columbia. Provincial Health Officer. (1992-) (Annual). *A report on the health of British Columbians: Provincial Health Officer's annual report*. Victoria, BC: Ministry of Health and Ministry Responsible for Seniors.

British Columbia Task Force into Illicit Narcotic Overdose Deaths in British Columbia. (1994). *Report of the Task Force into Illicit Narcotic Deaths in British Columbia*. Victoria, BC: Ministry of Health.

Cain, J.V. (1994). The Cain report. See British Columbia Task Force into Illicit Narcotic Overdose Deaths in British Columbia.

Canadian Centre on Substance Abuse. (1996). *The costs of substance abuse in Canada*. A cost estimation study by Eric Single, Lynda Robson, Xiaodi Xie, and Jurgen Rehm. Toronto, ON: Canadian Centre on Substance.

Canadian Centre on Substance Abuse National Working Group on Policy. (1997). Understanding harm reduction. *Addiction Research Foundation Journal*, *26*(4). Reprinted in PHERO (*Public Health and Epidemiology Report Ontario*, December 19, 1997, pp. 288-291.

Canadian Public Health Association. AIDS Program. (1997, July). *National HIV Prevention Program: Strategic plan for 1998-2003*. Ottawa, ON: Canadian Public Health Association.

Drug Courts Clearinghouse and Technical Assistance Project. (a program of the Drug Courts Program Office, Office of Justice Programs, U.S. Department of Justice) (1996, May). *Summary assessment of the drug court experience*. The American University.

Farrell, M., & Hall, W. (1998, February 28). The Swiss heroin trials: Testing alternative approaches. *British Medical Journal*, *316*, 639.

Fischer, B., & Rehm, J. (1997). The case for a heroin substitution treatment trial in Canada. *Canadian Journal of Public Health*, *88*(6), 367-370.

Fuddy, L.J. (1992). *Hawaii Healthy Start successful in preventing child abuse*. Mimeograph describing the Hawaii Healthy Start Program operated by the State of Hawaii Maternal and Child Health Branch, Department of Health. (Available from Loretta J. Fuddy, 741A Sunset Avenue, Honolulu 96816)

Health Officers Council of British Columbia (1998). *A comprehensive public health response to the problem of illicit injection drug use*. Recommendations developed by the Health Officers Council of British Columbia, May 1998.

Nadelmann, E.A. Commonsense drug policy. *Foreign Affairs*, *77*(1), 111-126.

National Association of Drug Court Professionals Drug Court Standards Committee (1997, January). *Defining drug courts: The key components*. U.S. Department of Justice. Office of Justice Programs. Drug Courts Program Office.

National Task Force on HIV, AIDS, and Injection Drug Use. (1997, May). *HIV, AIDS, and injection drug use: A national action plan*. Funded by the National AIDS Strategy, in collaboration with Canada's Drug Strategy, Health Canada.

Schweinhart, L.J., Barnes, H.V., & Weikart, D.P. (1993). *Significant benefits: The High/Scope Perry Preschool Study through Age 27*. High/Scope Educational Research Foundation Monograph Number 10. Ipsilante, MI: High/Scope Press.

"Something to eat, a place to sleep, and someone who gives a damn": HIV/AIDS and injection drug use in the DTES. (1997, September). Final project report to the Downtown Eastside community, Joy MacPhail, Minister of Health, and the Vancouver/Richmond Health Board.

Stewart, J. (1997, August 13). *The social cost of untreated opiate use*. Toronto, ON: Addiction Research Foundation.

Strathdee, S.A., Patrick, D.M., Currie, S., Cornelisse, P.G.A., Rekart, M.L., Montaner, J.S.G., Schechter, M.T., O'Shaughnessy, M.V. (1997, July). Needle exchange is not enough: Lessons from the Vancouver Injection Drug Use Study. *AIDS*, *11*(8), F59-F65.

Temporary Advisory Sub-Committee on Narcotics Harm Reduction. (1997, December). *No further harm: Report of the Temporary Advisory Sub-Committee on Narcotics Harm Reduction*. Submitted to the Professional Advisory Committee of the British Columbia Medical Association and the British Columbia Ministry of Health.

Uchtenhagen, A. (1997, July 10). *Summary of the synthesis report: Final report of the research representatives, Programme for a Medical Prescription of narcotics*. (Available from the Swiss Federal Office of Public Health in Berne and from the Addiction Research Institute in Zurich)

Whynot, E. (1996, May 6). *Health impact of injection drug use and HIV in Vancouver*. Report prepared by Dr. Elizabeth Whynot on request of Vancouver's Medical Health Officer Dr. John Blatherwick and Dr. Anne Vogel and presented to the Vancouver Health Board on May 23, 1996.

APPENDIX C
Health Region Data

New Positive HIV Tests

Table 4 Persons Testing Newly Positive for HIV, Health Regions, B.C., 1996

Health Region	New cases	Rate per 100,000
East Kootenay	2	2.5
West Kootenay	10	12.3
North Okanagan	4	3.3
South Okanagan	12	5.4
Thompson	5	3.9
Fraser Valley	17	7.4
South Fraser Valley	41	7.8
Simon Fraser	40	13.5
Coast Garibaldi	7	9.5
Central Vanc Island	14	5.9
Upper Island	8	6.7
Cariboo	3	4.0
North West	2	2.2
Peace Liard	1	1.5
Northern Interior	2	1.6
Vancouver	469	86.4
Burnaby	19	10.6
North Shore	13	7.5
Richmond	8	5.4
Capital	36	10.8
Subtotals		
Vancouver	469	86.4
Other metro*	116	10.2
Other areas	128	5.9
British Columbia total	713	18.5
Highest region		86.4
Lowest region		1.5

Number of newly positive HIV cases (persons who tested HIV positive for the first time) in 1996. Source: STD/AIDS Control, B.C. Centre for Disease Control Society. AIDS Update: First Quarter 1997.

Table 5 Persons Testing Newly Positive for HIV with Risk Category IDU, Health Regions, B.C., 1996

Health Region	New cases
East Kootenay	0
West Kootenay	1
North Okanagan	3
South Okanagan	4
Thompson	4
Fraser Valley	12
South Fraser Valley	31
Simon Fraser	24
Coast Garibaldi	4
Central Vanc Island	5
Upper Island	6
Cariboo	1
North West	2
Peace Liard	0
Northern Interior	1
Vancouver	258
Burnaby	8
North Shore	3
Richmond	3
Capital	17
Subtotals	
Vancouver	258
Other metro*	55
Other areas	74
British Columbia total	387

Number of newly positive HIV cases (persons who tested HIV positive for the first time) in 1996 with risk category injection drug use (IDU, MSM/IDU, or sex trade worker/IDU). Source: STD/AIDS Control, B.C. Centre for Disease Control Society. Unpublished tables, November 10, 1997.

** "Other metro" includes the Simon Fraser, Burnaby, Richmond, North Shore, and Capital health regions.*

Drug Treatment Participants

Table 6 Active Participants, HIV/AIDS Drug Treatment Program, Health Regions, B.C., November 1997

Health Region	Number	Rate per 100,000
East Kootenay	7	8.6
West Kootenay	17	20.6
North Okanagan	16	13.0
South Okanagan	57	25.2
Thompson	20	15.0
Fraser Valley	41	17.4
South Fraser Valley	173	32.1
Simon Fraser	148	48.2
Coast Garibaldi	35	45.7
Central Vanc Island	58	24.0
Upper Island	16	13.0
Cariboo	12	15.5
North West	8	8.5
Peace Liard	2	3.0
Northern Interior	20	15.2
Vancouver	1,439	259.8
Burnaby	90	49.4
North Shore	79	44.9
Richmond	40	26.3
Capital	165	48.8
Unspecified	14	
Subtotals		
Vancouver	1,439	259.8
Other metro*	522	45.2
Other areas	482	21.6
British Columbia total	2,457	62.3
Highest region		259.8
Lowest region		3.0

Number of total active participants in the HIV/AIDS Drug Treatment Program. Rate is per 100,000 total population in the specified region. Source: HIV/AIDS Drug Treatment Program, B.C. Centre for Excellence in HIV/AIDS.

Table 7 Participants Who Are Active Injection Drug Users (self-report), HIV/AIDS Drug Treatment Program, Health Regions, B.C., November 1997

Health Region	Number	Rate per 100,000
East Kootenay	<5	0.0
West Kootenay	<5	0.0
North Okanagan	<5	0.8
South Okanagan	8	3.5
Thompson	<5	1.5
Fraser Valley	7	3.0
South Fraser Valley	23	4.3
Simon Fraser	26	8.5
Coast Garibaldi	<5	2.6
Central Vanc Island	10	4.1
Upper Island	<5	2.4
Cariboo	<5	0.0
North West	<5	2.1
Peace Liard	<5	0.0
Northern Interior	<5	1.5
Vancouver	79	14.3
Burnaby	13	7.1
North Shore	<5	1.7
Richmond	<5	2.0
Capital	14	4.1
Unspecified	3	
Subtotals		
Vancouver	79	14.3
Other metro*	59	5.1
Other areas	60	2.7
British Columbia total	201	5.1
Highest region		14.3
Lowest region		0.0

Number of participants in the HIV/AIDS Drug Treatment Program who report that they are active injection drug users; regions with fewer than 5 are shown as "<5". Rate is per 100,000 total population in the specified region. Source: HIV/AIDS Drug Treatment Program, B.C. Centre for Excellence in HIV/AIDS.

** "Other metro" includes the Simon Fraser, Burnaby, Richmond, North Shore, and Capital health regions.*

Hepatitis C

Table 8 Reported Cases of Hepatitis C, Health Regions, B.C., 1997

Health Region	Cases	Rate per 100,000
East Kootenay	68	83.3
West Kootenay	140	169.7
North Okanagan	213	172.5
South Okanagan	375	165.8
Thompson	264	198.2
Fraser Valley	654	278.3
South Fraser Valley	921	170.8
Simon Fraser	600	195.5
Coast Garibaldi	189	246.8
Central Vanc Island	643	265.8
Upper Island	280	228.1
Cariboo	69	88.9
North West	106	112.1
Peace Liard	84	127.2
Northern Interior	197	149.7
Vancouver	1,997	360.5
Burnaby	408	224.0
North Shore	225	127.9
Richmond	140	92.1
Capital	628	185.8
Subtotals		
Vancouver	1,997	360.5
Other metro*	2,001	173.2
Other areas	4,203	188.3
British Columbia total	8,201	208.1
Highest region		360.5
Lowest region		83.3

Source: Epidemiology Services, B.C. Centre for Disease Control Society. Unpublished tables, June 1998.

** "Other metro" includes the Simon Fraser, Burnaby, Richmond, North Shore, and Capital health regions.*

Hepatitis B

Table 9 Reported Cases of Hepatitis B (Acute and Undetermined), Health Regions, B.C., 1997

Health Region	Cases	Rate per 100,000
East Kootenay	2	2.4
West Kootenay	6	7.3
North Okanagan	22	17.8
South Okanagan	15	6.6
Thompson	7	5.3
Fraser Valley	20	8.5
South Fraser Valley	42	7.8
Simon Fraser	45	14.7
Coast Garibaldi	7	9.1
Central Vanc Island	25	10.3
Upper Island	2	1.6
Cariboo	4	5.2
North West	5	5.3
Peace Liard	2	3.0
Northern Interior	2	1.5
Vancouver	146	26.4
Burnaby	37	20.3
North Shore	60	34.1
Richmond	266	175.0
Capital	36	10.7
Subtotals		
Vancouver	146	26.4
Other metro*	444	38.4
Other areas	161	7.2
British Columbia total	751	19.1
Highest region		175.0
Lowest region		1.5

Number of reported cases of hepatitis B, acute/undetermined. Acute cases are persons recently infected. Undetermined cases are persons in whom it could not be determined on the basis of the bloodwork at hand whether they were acute of chronically infected; some of these undetermined cases will ultimately be classified as chronic. Source: Epidemiology Services, B.C. Centre for Disease Control Society. Unpublished tables, June 1998.

Hepatitis B

Table 10 Reported Cases of Hepatitis B (Carrier/Chronic), Health Regions, B.C., 1997

Health Region	Cases	Rate per 100,000
East Kootenay	1	1.2
West Kootenay	1	1.2
North Okanagan	10	8.1
South Okanagan	10	4.4
Thompson	11	8.3
Fraser Valley	42	17.9
South Fraser Valley	165	30.6
Simon Fraser	172	56.0
Coast Garibaldi	1	1.3
Central Vanc Island	22	9.1
Upper Island	16	13.0
Cariboo	0	0.0
North West	1	1.1
Peace Liard	3	4.5
Northern Interior	3	2.3
Vancouver	1,350	243.7
Burnaby	232	127.4
North Shore	17	9.7
Richmond	153	100.6
Capital	42	12.4
Subtotals		
Vancouver	1,350	243.7
Other metro*	616	53.3
Other areas	286	12.8
British Columbia totals	2,252	57.1
Highest region		243.7
Lowest region		0.0

Number of reported cases of hepatitis B, carrier/chronic. Source: Epidemiology Services, B.C. Centre for Disease Control Society. Unpublished tables, June 1998.

** "Other metro" includes the Simon Fraser, Burnaby, Richmond, North Shore, and Capital health regions.*

Illicit Drug Deaths

Table 11 Illicit Drug Deaths, Health Regions, B.C., 1996

Health Region	Deaths	Rate per 100,000
East Kootenay	2	3.7
West Kootenay	0	0.0
North Okanagan	3	3.9
South Okanagan	4	2.9
Thompson	7	7.9
Fraser Valley	15	10.4
South Fraser Valley	29	8.3
Simon Fraser	17	8.2
Coast Garibaldi	5	10.1
Central Vanc Island	5	3.3
Upper Island	8	9.9
Cariboo	2	3.8
North West	3	4.7
Peace Liard	0	0.0
Northern Interior	6	6.7
Vancouver	135	33.4
Burnaby	14	10.2
North Shore	11	9.2
Richmond	4	3.8
Capital	20	9.1
Other/Unspecified	5	
Subtotals		
Vancouver	135	33.4
Other metro*	66	8.3
Other areas	89	6.2
British Columbia total	301	11.4
Highest region		33.4
Lowest region		0.0

Deaths due to illicit drugs, as determined by the B.C. Coroners Service, by health region of residence, 1996. B.C. total includes 6 deaths to out-of-province residents and 5 deaths of unspecified health region. Rate is per 100,000 population age 15-64. Source: Number of deaths from B.C. Coroners Service, Vancouver.

Table 12 Hospitalizations Due to HIV/AIDS by Region of Patient Residence, All Levels of Care, B.C., 1996/97

Health Region	Cases	Days
East Kootenay	6	75
West Kootenay	4	69
North Okanagan	1	31
South Okanagan	18	69
Thompson	1	23
Fraser Valley	15	173
South Fraser Valley	44	871
Simon Fraser	32	431
Coast Garibaldi	12	62
Central Vanc Island	23	126
Upper Island	5	22
Cariboo	2	23
North West	7	78
Peace Liard	1	7
Northern Interior	6	127
Vancouver	440	4,728
Burnaby	23	328
North Shore	25	316
Richmond	34	349
Capital	52	550
B.C. unspecified	3	54
Non-residents	6	48
Subtotals		
Vancouver	440	4,728
Other metro*	166	1,974
Other areas	145	1,756
British Columbia total	760	8,560

Source: Morbidity Database. Information and Analysis Branch, B.C. Ministry of Health.

** "Other metro" includes the Simon Fraser, Burnaby, Richmond, North Shore, and Capital health regions.*
