# Supplementary material for: Fighting addiction's death row: British Columbia Supreme Court Justice Ian Pitfield shows a measure of legal courage
Source: Harm Reduct J. 2008 Oct 28;5:31. doi: 10.1186/1477-7517-5-31 (PMC2611970; doi:10.1186/1477-7517-5-31)
Supplement: Additional file 6 — Junkies and drug pushers don't belong near children and families. This is a pamphlet mailed out compliments of Rob Merrifield, Member of Parliament, in August of 2008. [file 1477-7517-5-31-S6.pdf]

Junkies and drug pushers don't belong near children and families.

They should be in rehab or behind bars.

The Conservative Government will clean up drug crime.

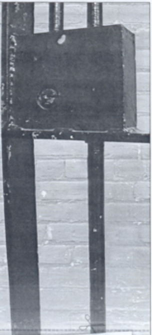

- ✓ Punish drug pushers with more jail time
- ✓ Keep junkies in rehab and off the streets
- ✓ Crack down on cross border drug smuggling

Who do you think is on the right track on crime?

Check one

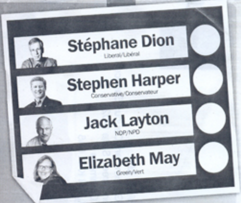

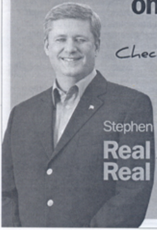

Stephen Harper

Real Action.
Real Results.

NAME

TELEPHONE

ADDRESS

EMAIL

CITY/TOWN

PROVINCE

POSTAL CODE

Compliments of Rob Merrifield, MP

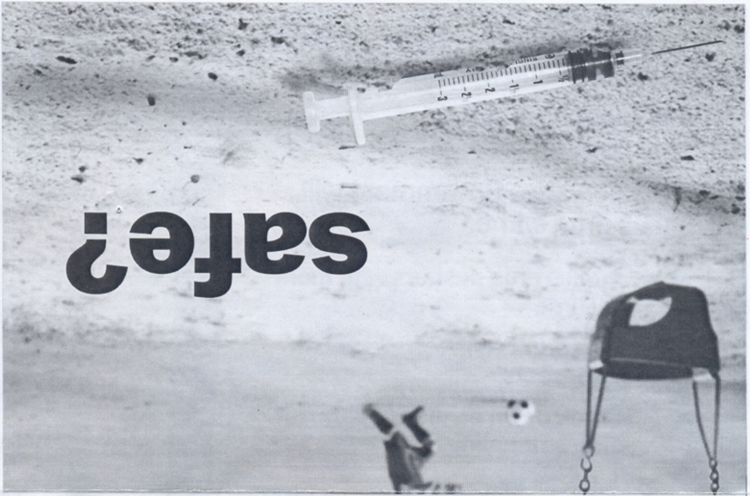

CUT

No Postage Required

Rob Merrifield, MP
c/o CRG-Government Caucus Services
131 Queen Street, Suite 8-02
House of Commons
OTTAWA, ON K1A 0A6

1002

FOLD

Canadians work hard and play by the rules. But where in the rules does it allow junkies and addicts to threaten or steal from innocent families? What rule says that drug pushers should be able to feed off the weak and sick in our society? What rule says that drug gangs can terrorize neighbourhoods? The answer is clear. Thugs, drug pushers, and others involved in the drug trade are writing their own rules. For too long, lax Liberal governments left gangs and drug pushers to make their own rules and set their own criminal agenda. Those days are over.

The Conservative Government is acting to stop this sickening trade now. We are punishing drug pushers, getting junkies and addicts off the streets, and cracking down on the flow of illegal drugs at the border. This is real action that we are taking for all Canadians who have been harmed, one way or another, by the illicit drug trade. We're putting the health and safety of law-abiding families first.

TAPE
